# Supplementary figures and images for: Diagnostic of fatty liver using radiomics and deep learning models on non-contrast abdominal CT
Source: PLoS One. 2025 Feb 13;20(2):e0310938. doi: 10.1371/journal.pone.0310938 (PMC11825062; doi:10.1371/journal.pone.0310938)

**S1 Fig. VB-net Structure**

**
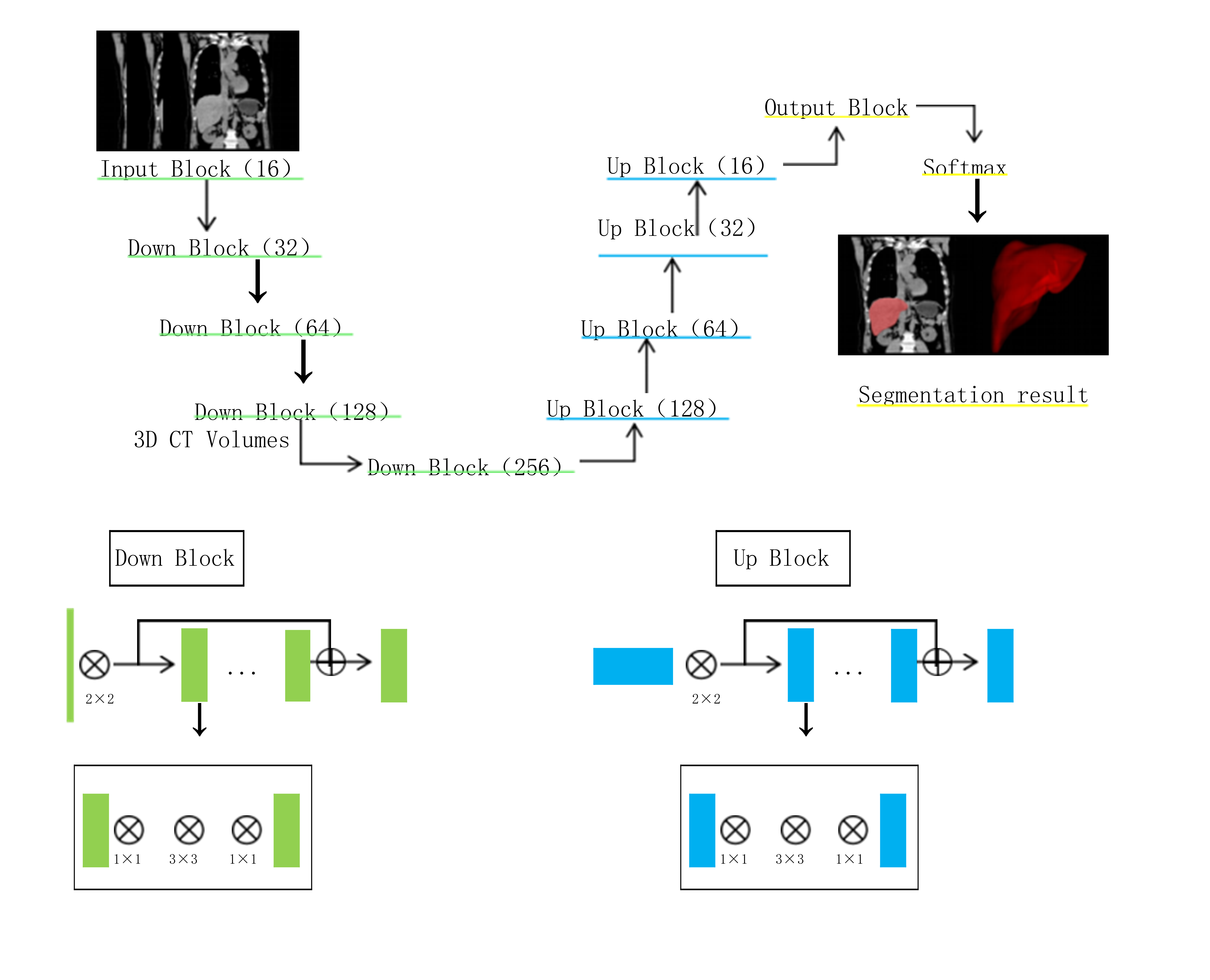
**

Supplement: S1 Fig — (DOCX) [file pone.0310938.s001.docx]

**S2 Fig.Visualisation of 2D model features**

**
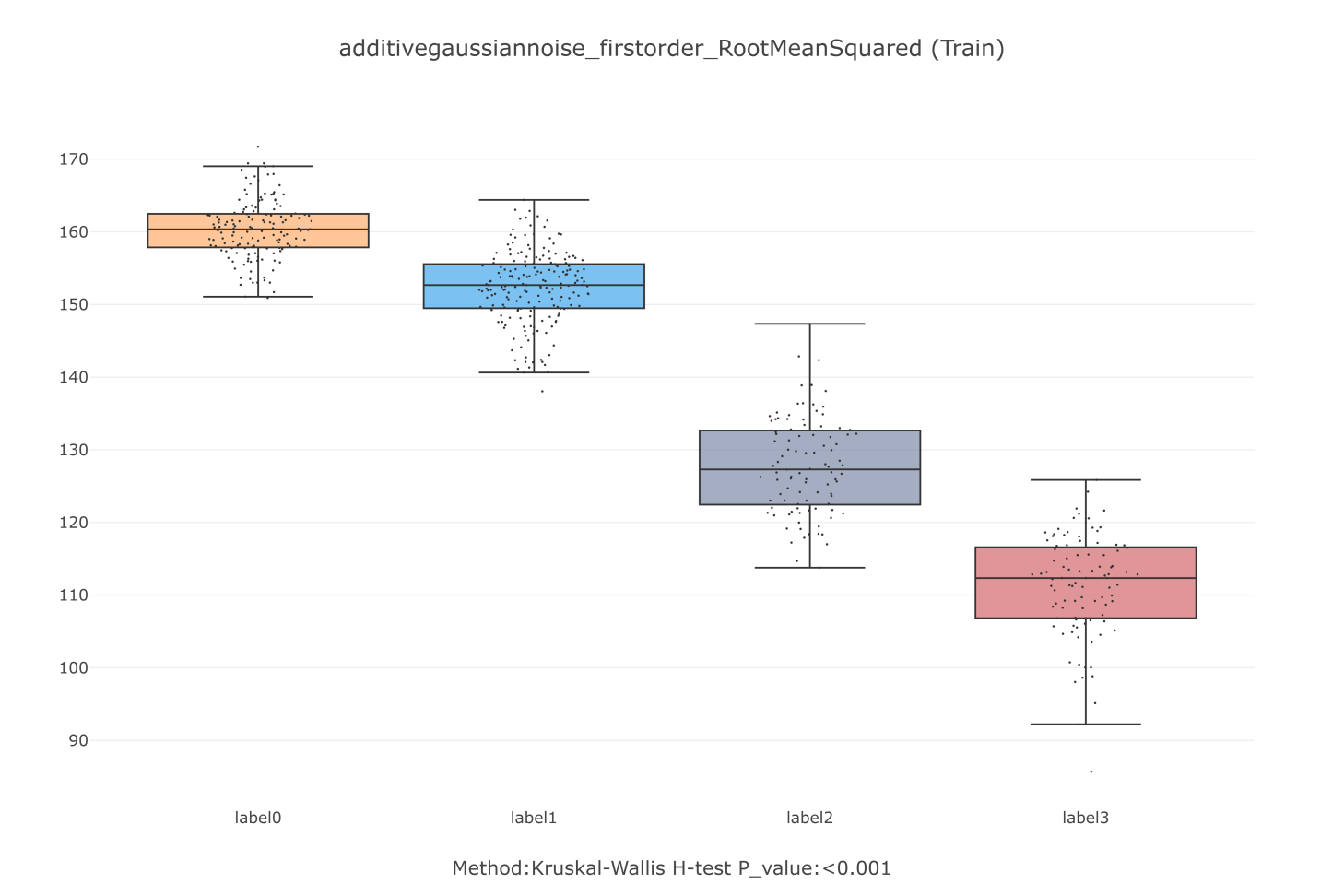
**

**
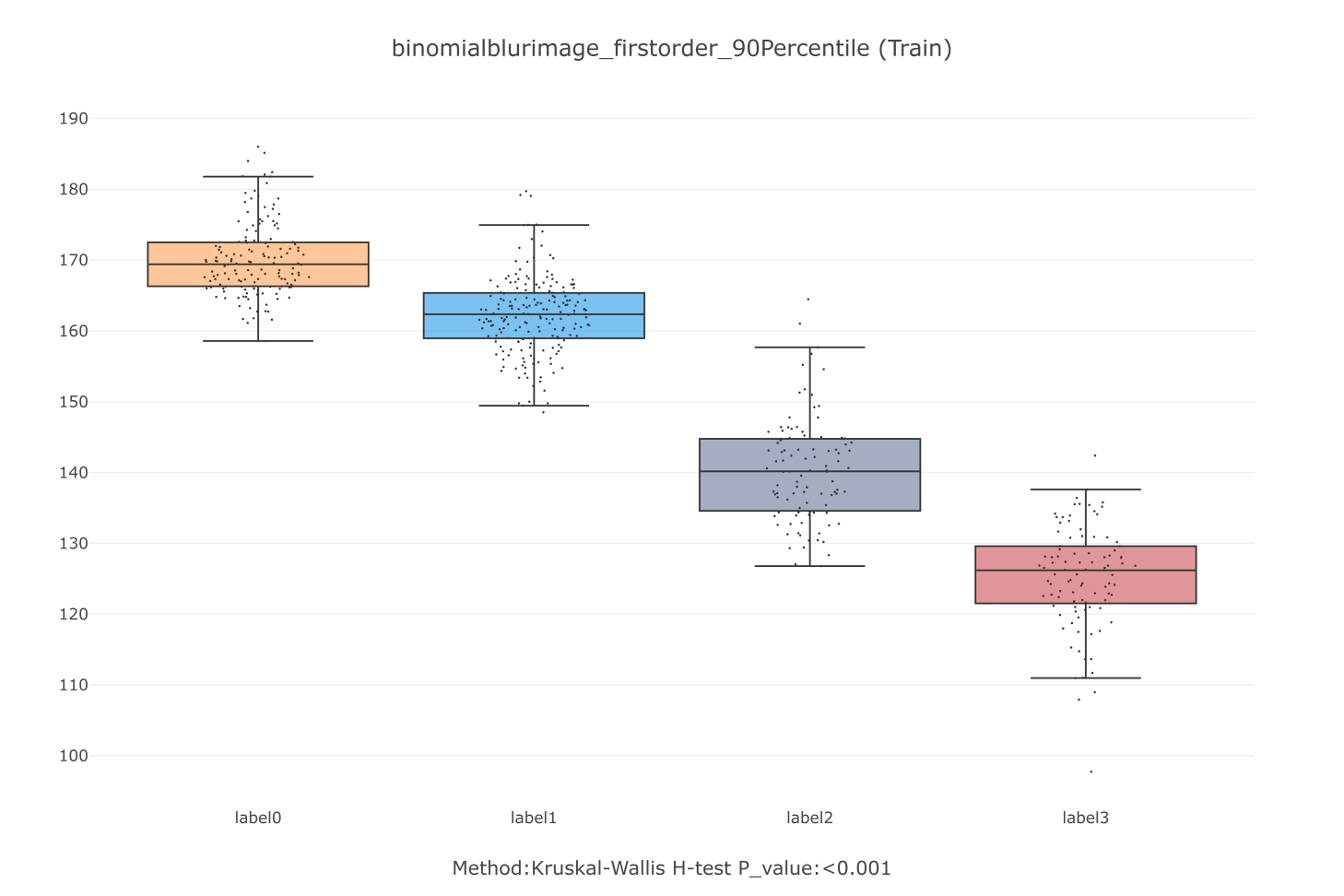
**

**
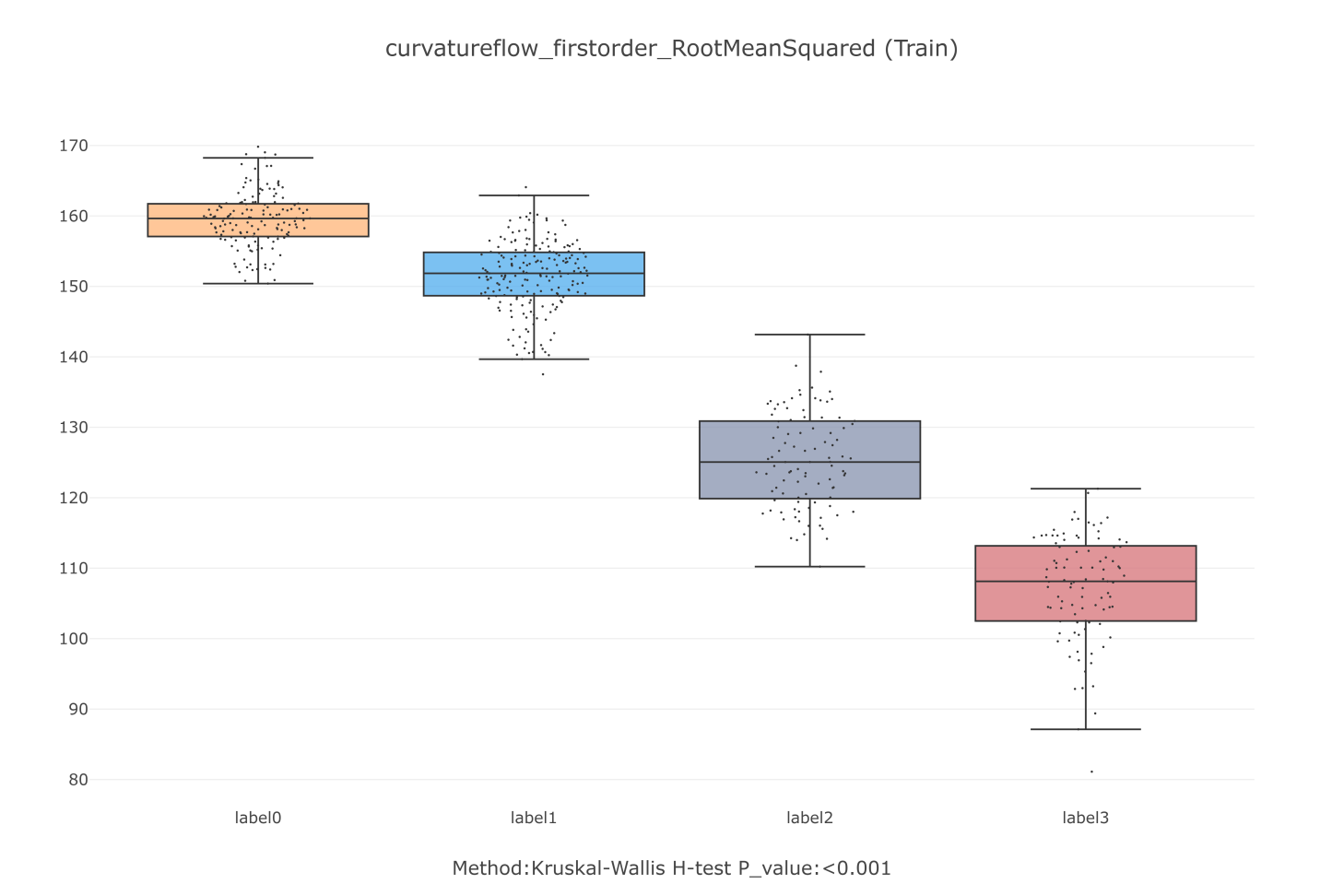
**

**
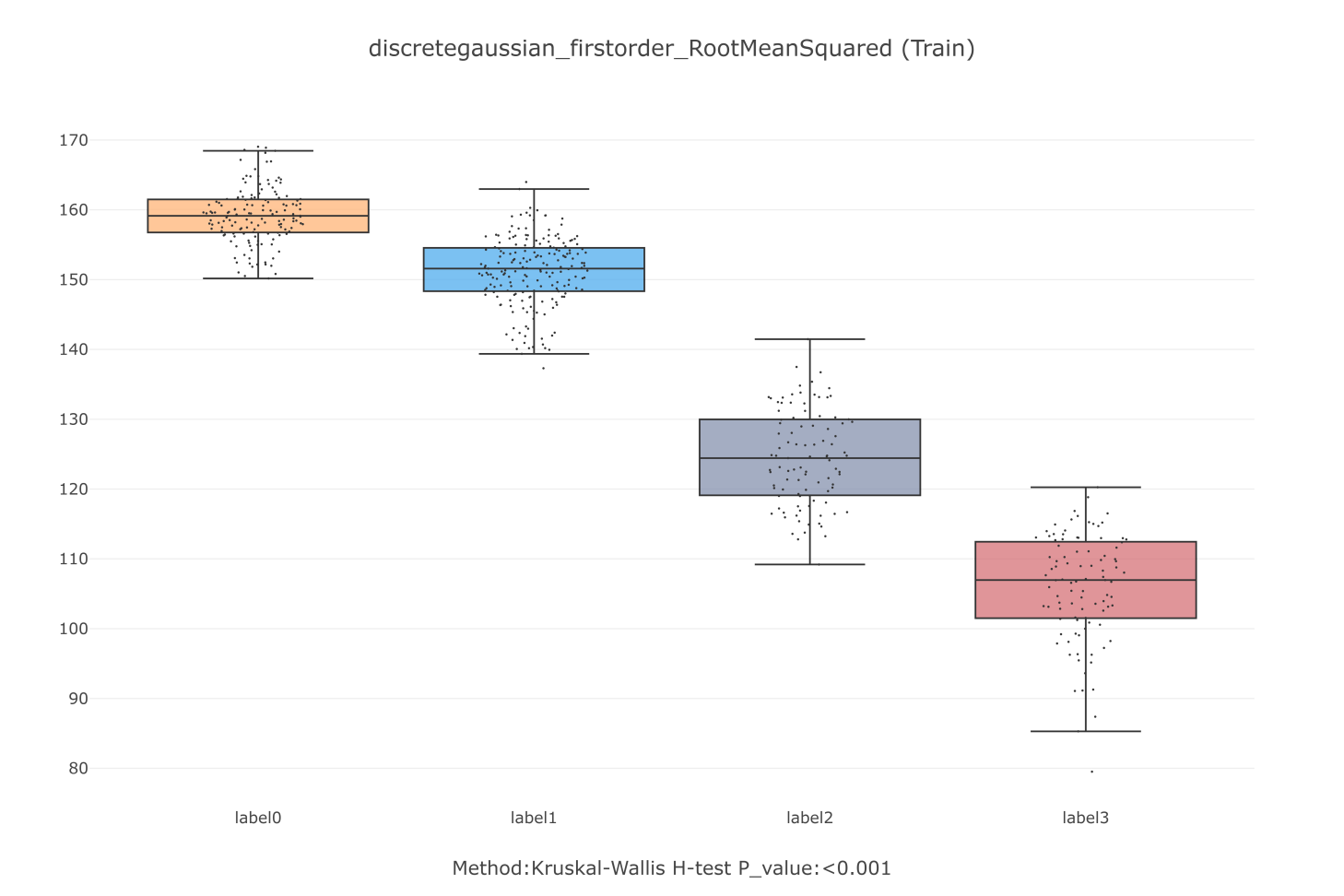
**

**
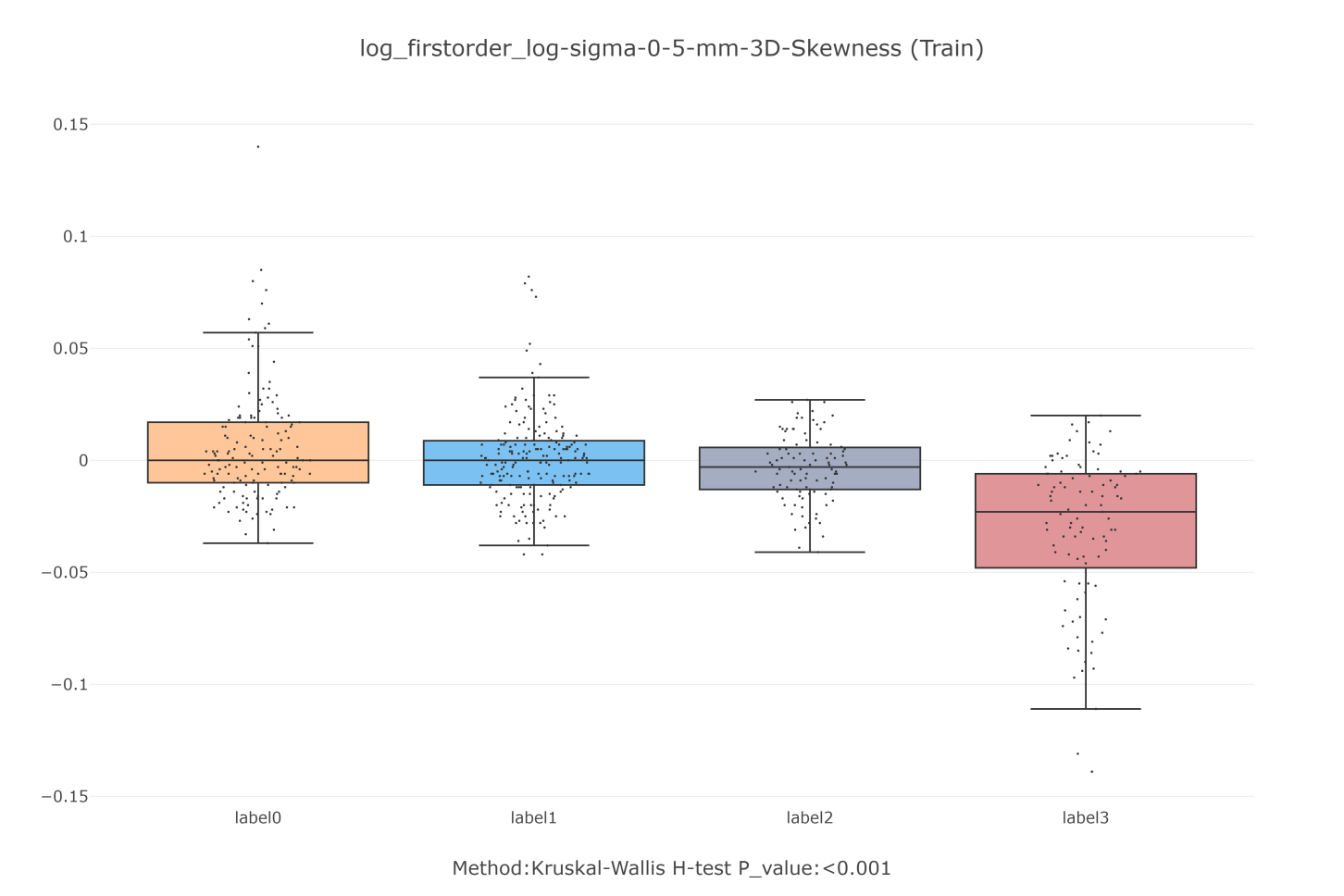
**

**
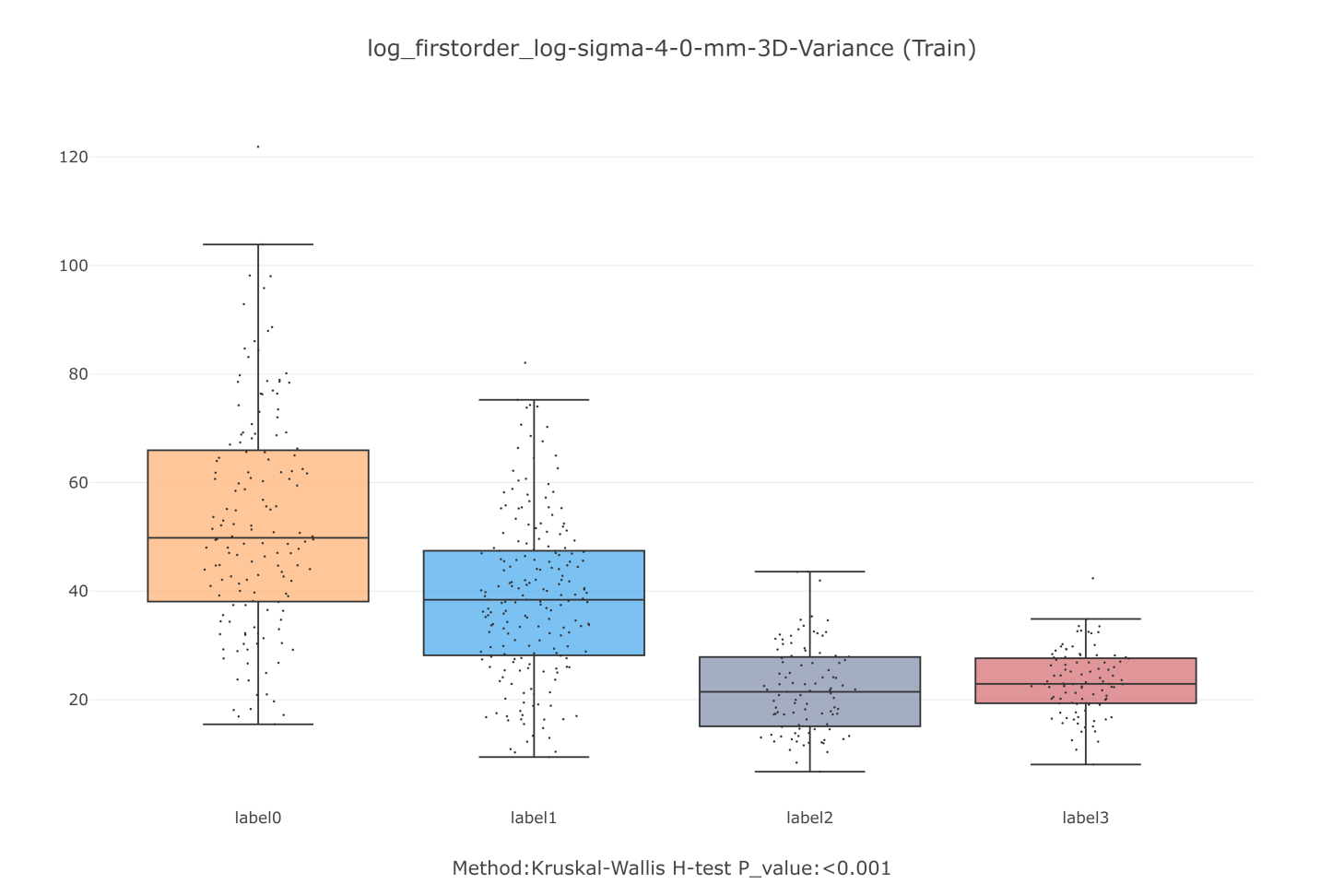
**

**
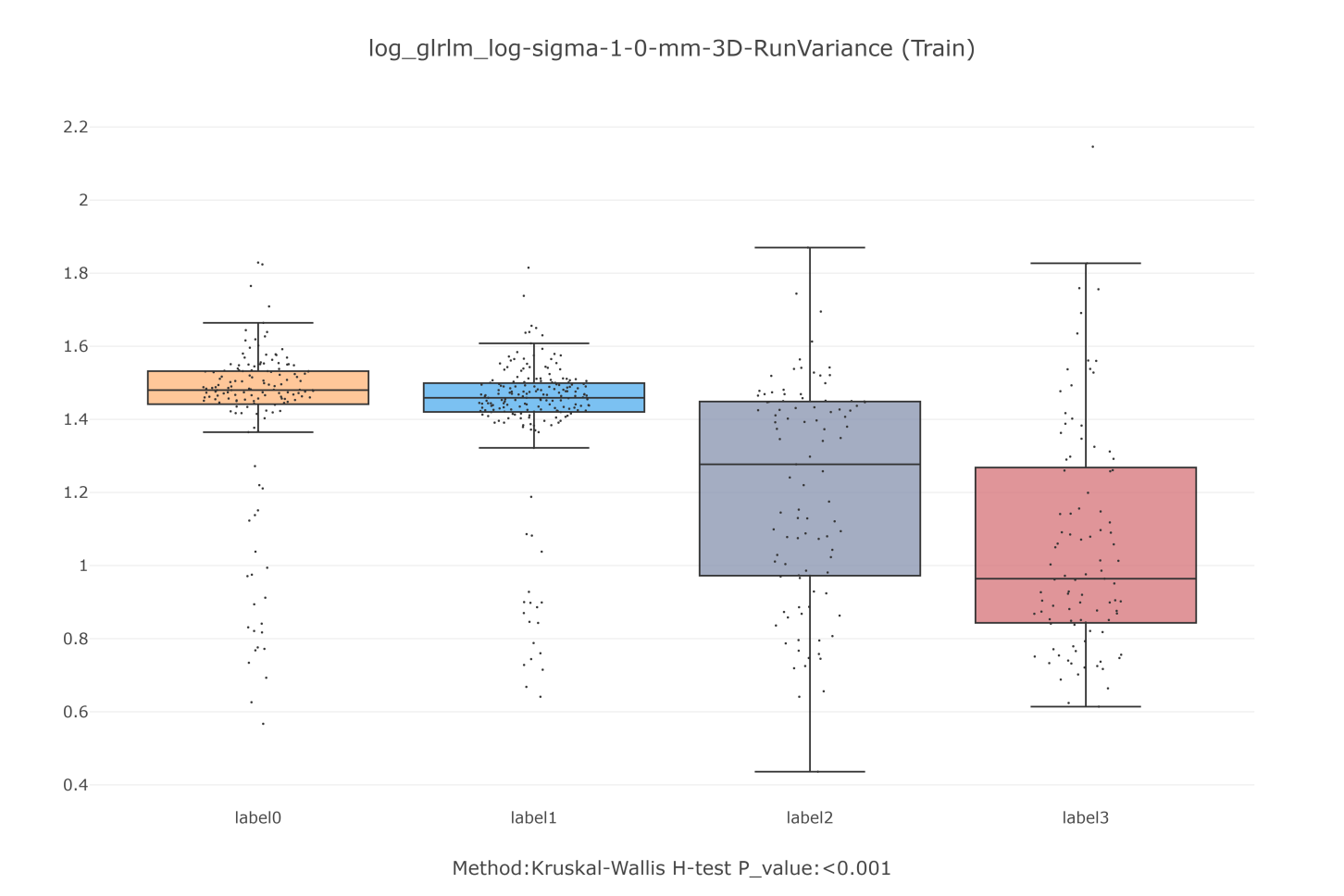
**

**
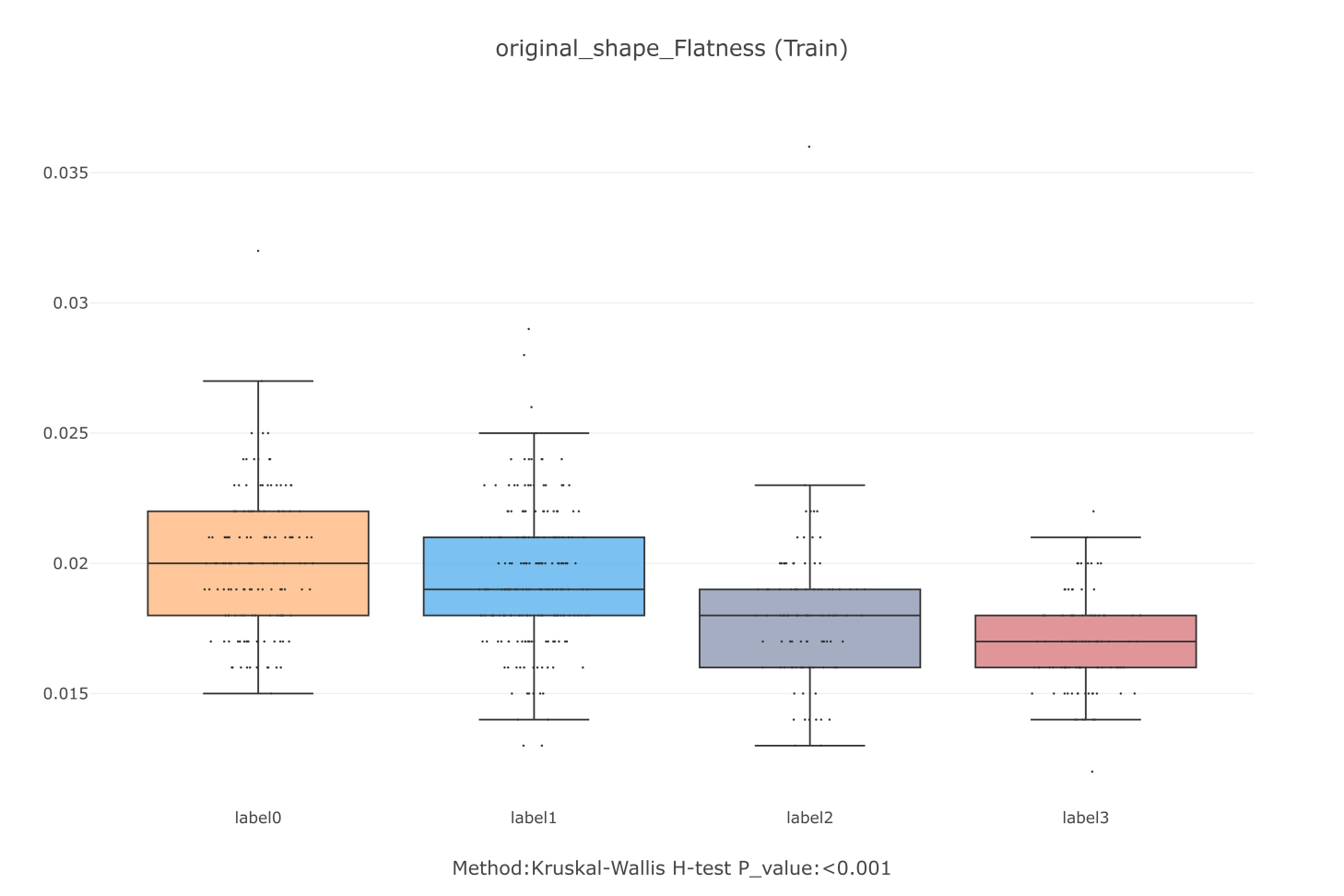
**

**
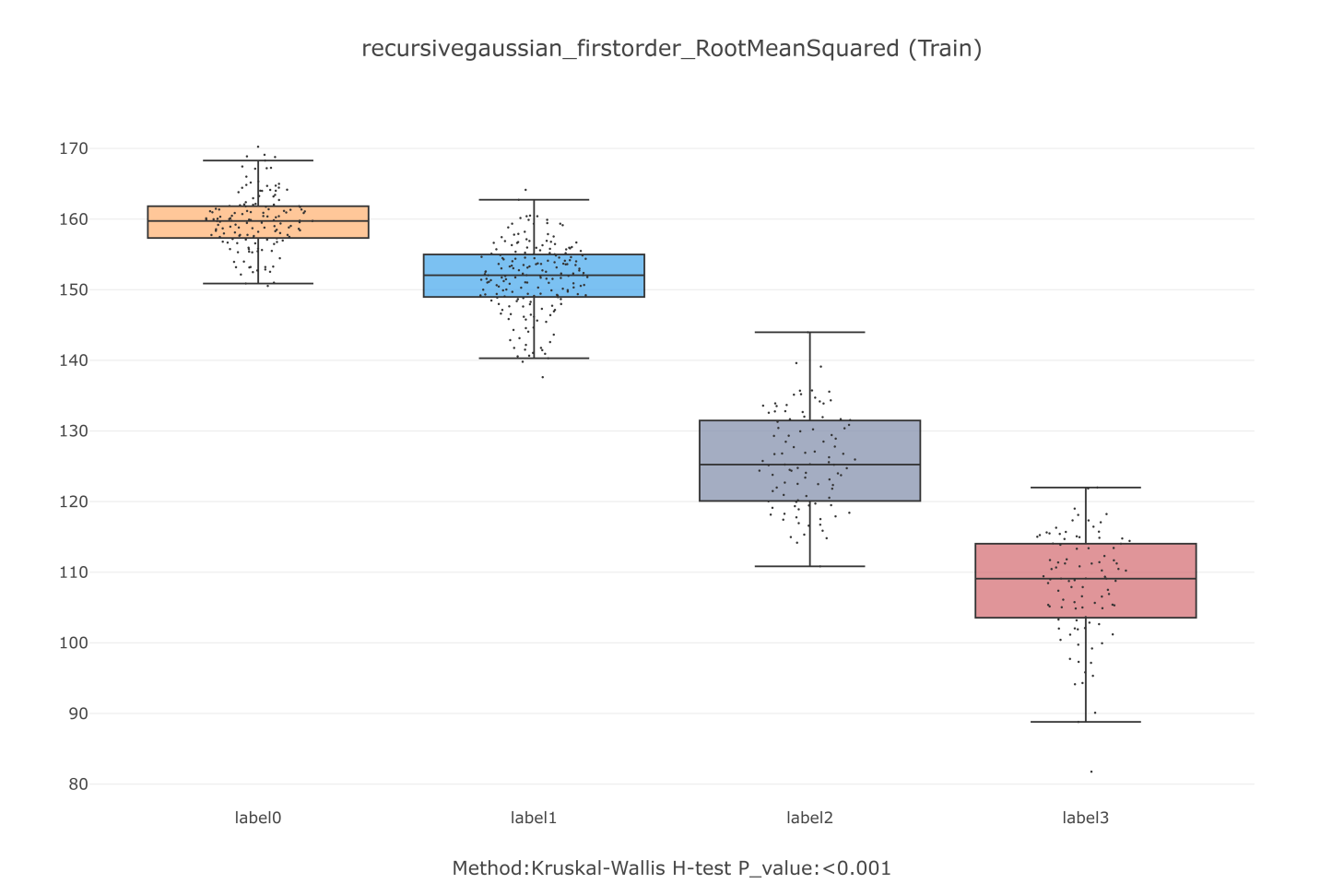
**

**
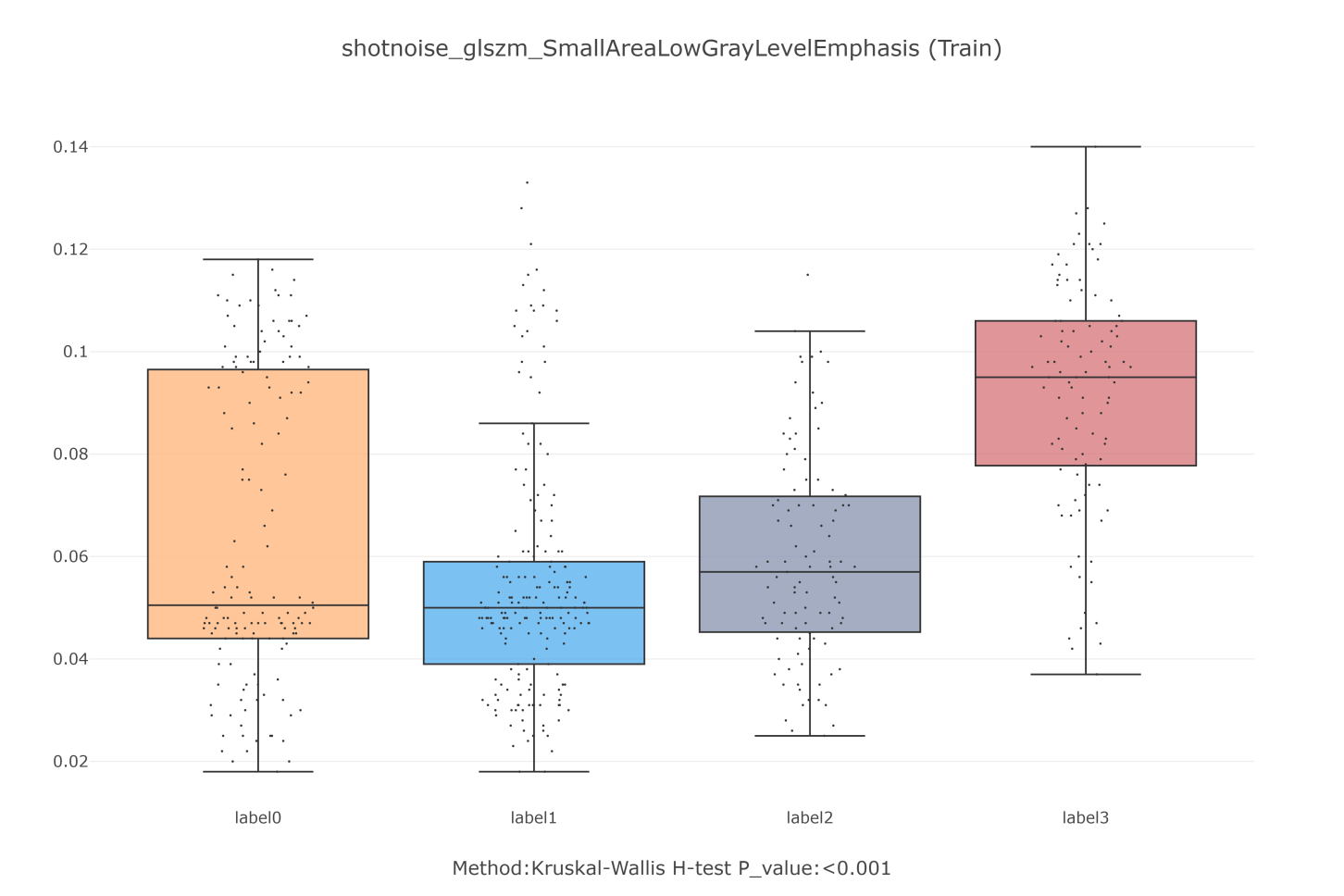
**

**
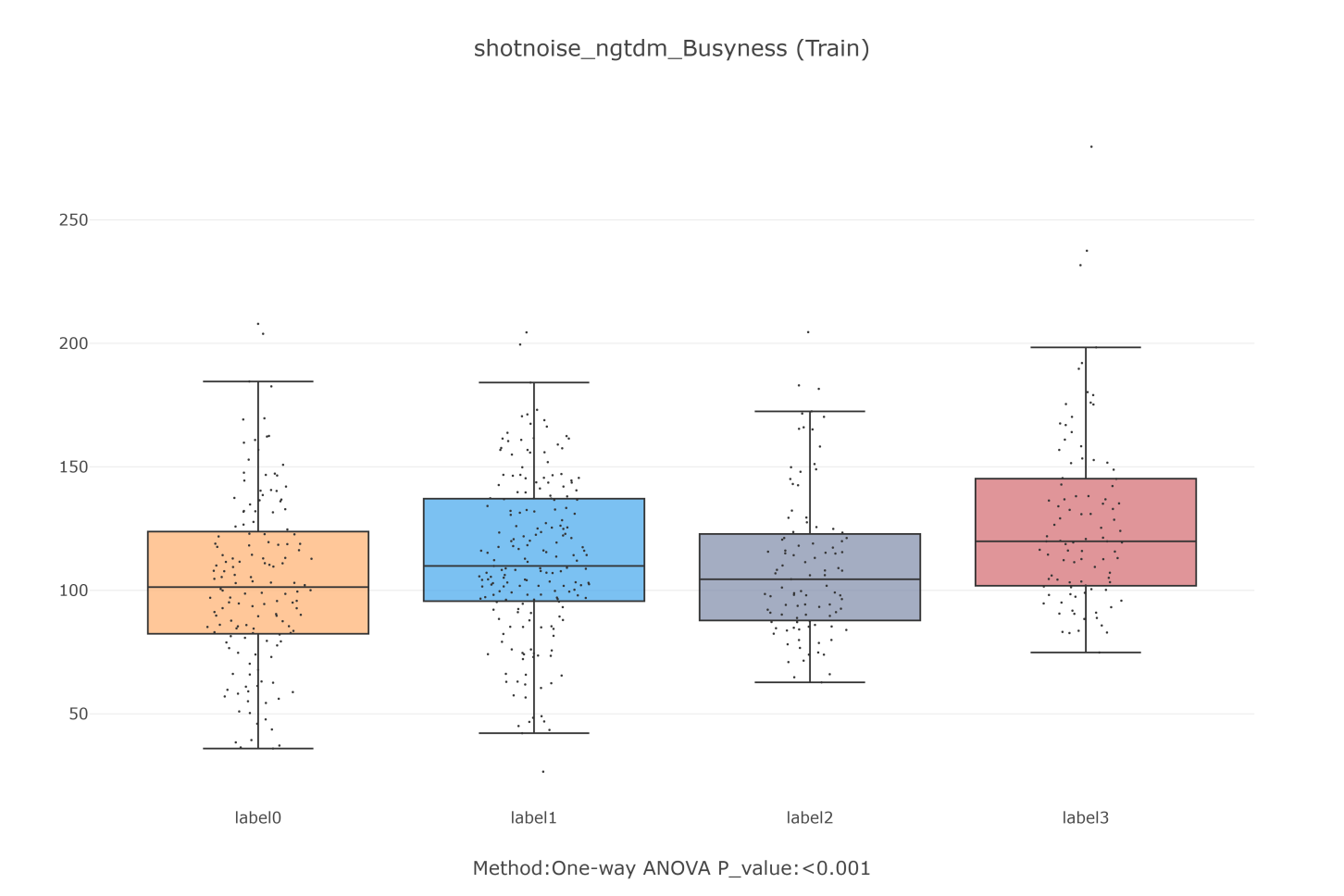
**

**
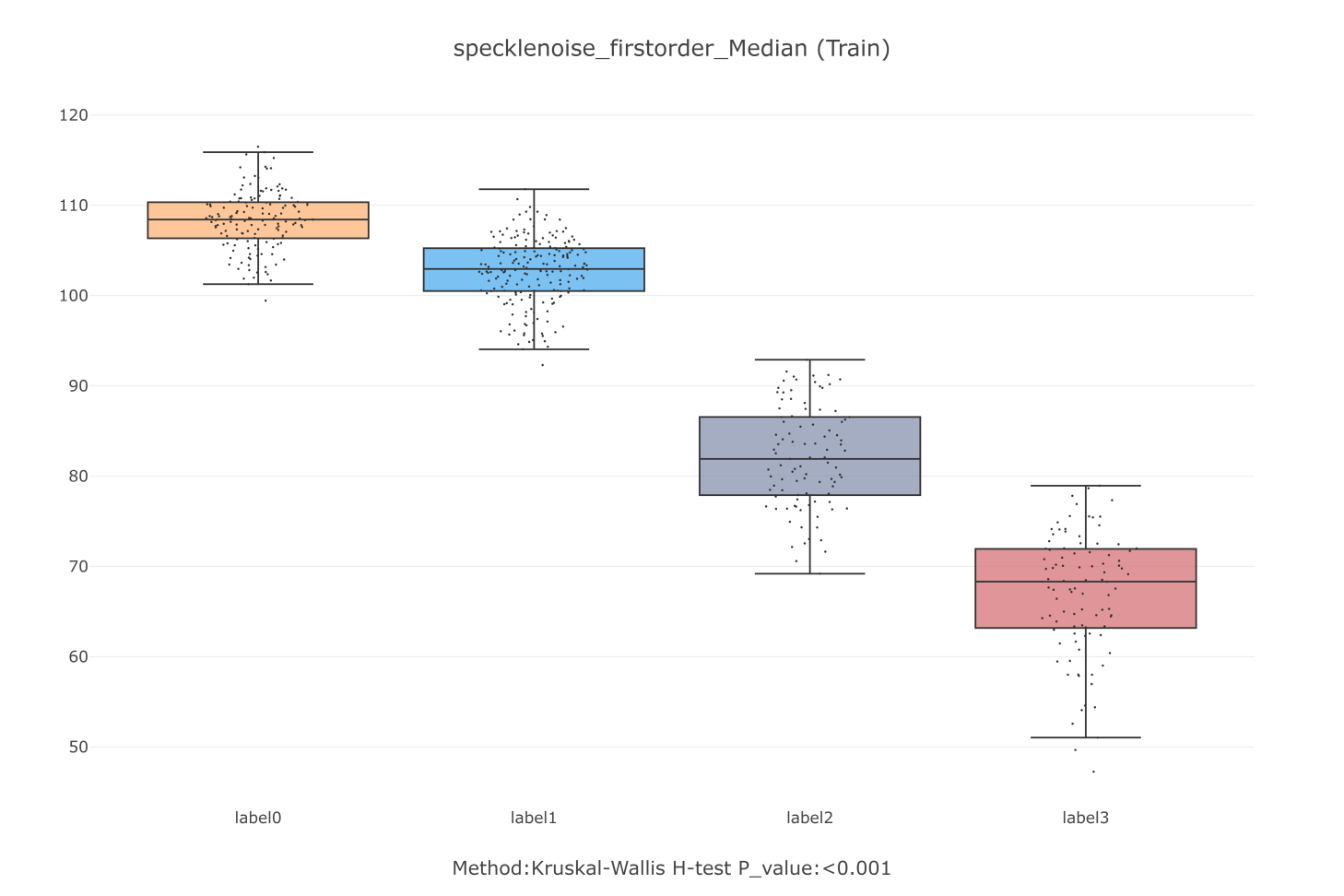
**

**
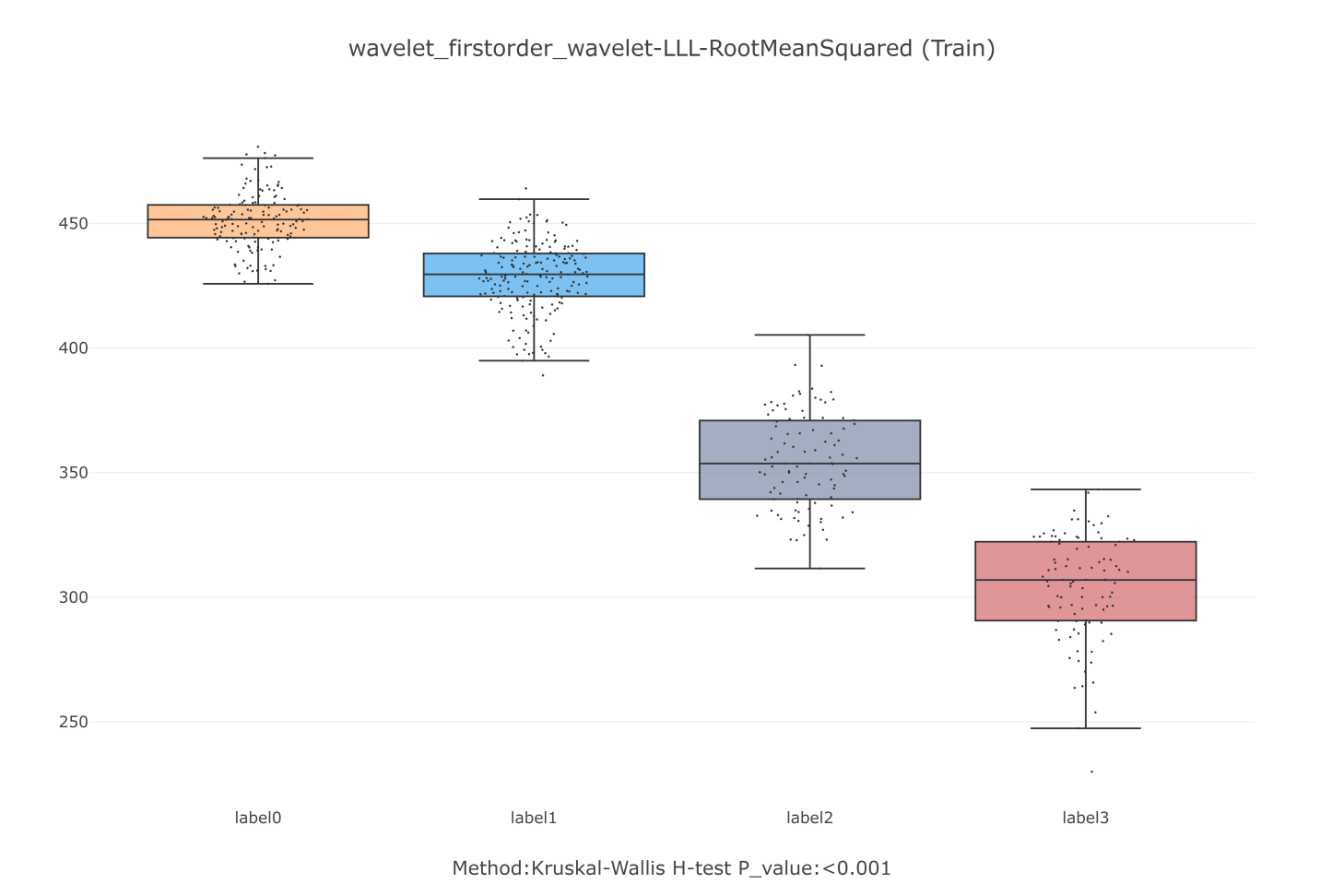
**

**
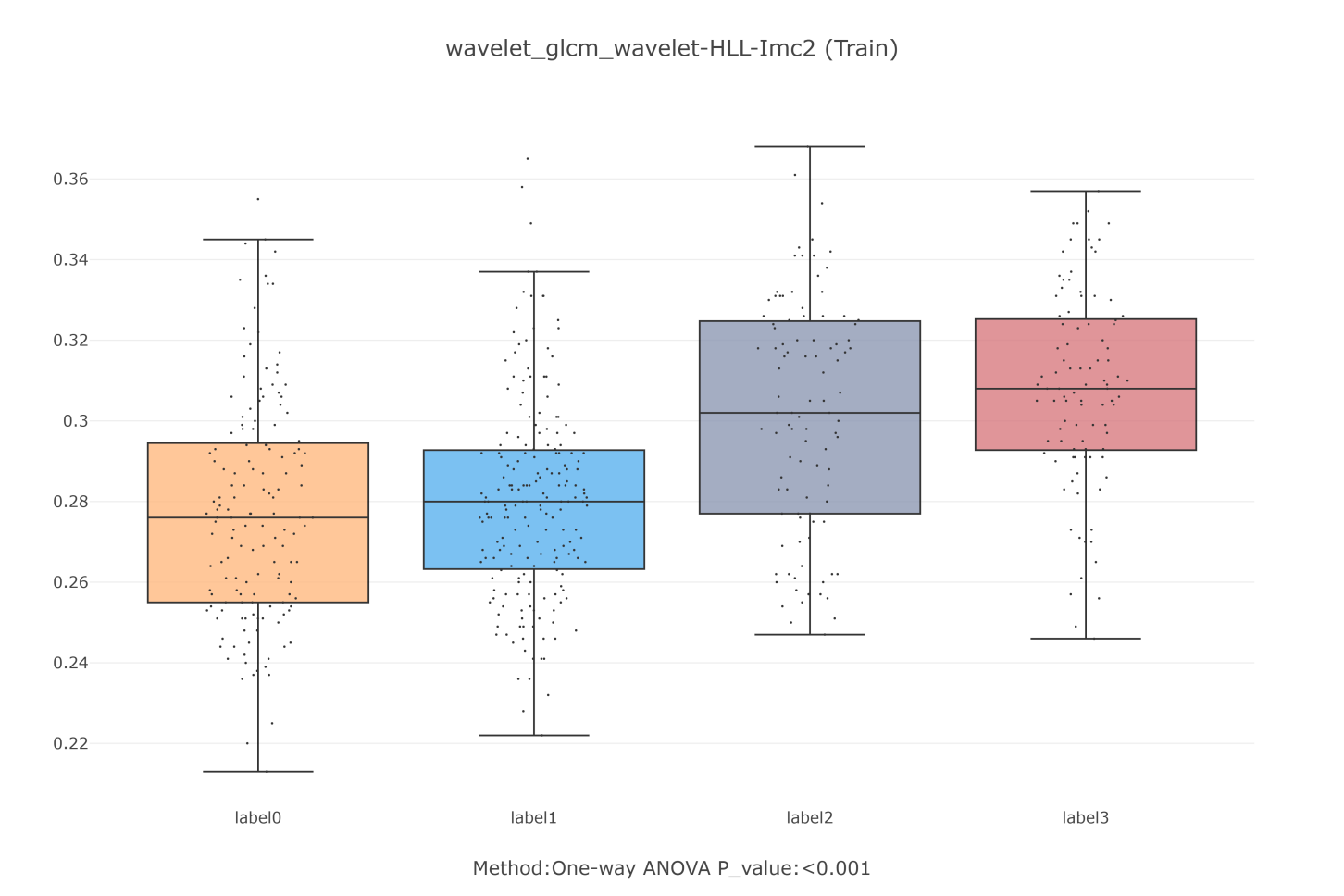
**

**
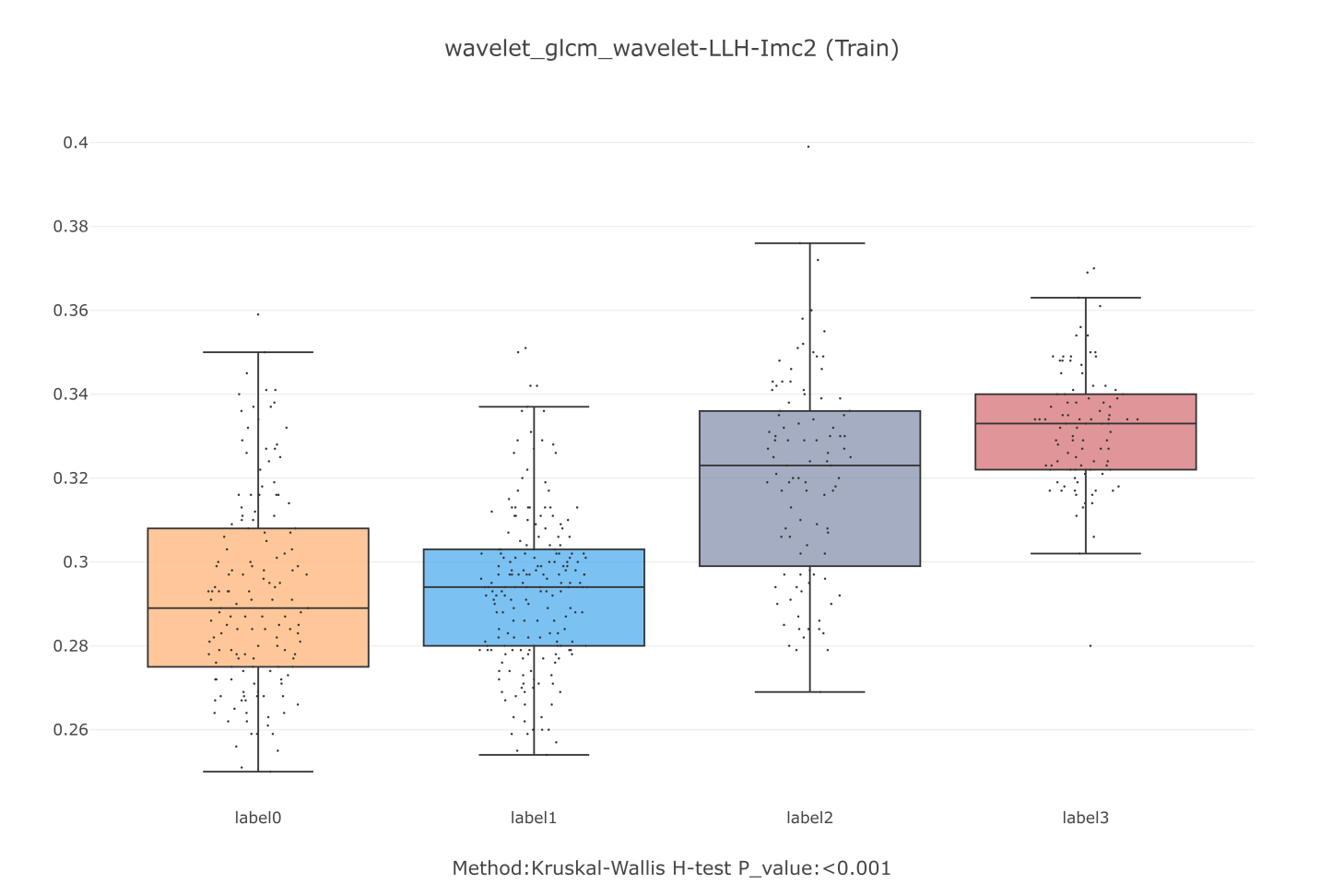
**

Supplement: S2 Fig — (DOCX) [file pone.0310938.s002.docx]
