# Supplementary material for: Diagnostic of fatty liver using radiomics and deep learning models on non-contrast abdominal CT
Source: PLoS One. 2025 Feb 13;20(2):e0310938. doi: 10.1371/journal.pone.0310938 (PMC11825062; doi:10.1371/journal.pone.0310938)
Supplement: S1 Table — (DOCX) [file pone.0310938.s003.docx]

**S1 Table. 2D radiomics model training and test set features**

| F1 | F2 | F3 | F4 | F5 | F6 | F7 | F8 | F9 | F10 | F11 | F12 | F13 | F14 | F15 | Label |  |
| --- | --- | --- | --- | --- | --- | --- | --- | --- | --- | --- | --- | --- | --- | --- | --- | --- |
| 0.016 | 160.266 | 168.656 | 159.740 | 0.039 | 50.855 | 1.506 | 451.718 | 0.295 | 0.277 | 159.576 | 109.724 | 159.836 | 0.102 | 203.864 | 0 | train |
| 0.022 | 169.035 | 175.469 | 168.719 | 0.000 | 69.244 | 1.474 | 477.321 | 0.268 | 0.244 | 168.586 | 116.484 | 168.783 | 0.054 | 84.604 | 0 | train |
| 0.017 | 157.688 | 167.594 | 156.595 | -0.005 | 28.097 | 1.384 | 443.182 | 0.308 | 0.266 | 156.234 | 106.457 | 156.919 | 0.028 | 72.160 | 1 | train |
| 0.020 | 152.316 | 162.359 | 151.535 | 0.012 | 62.185 | 1.497 | 428.663 | 0.291 | 0.256 | 151.164 | 102.666 | 151.736 | 0.031 | 82.340 | 1 | train |
| 0.017 | 127.368 | 137.000 | 126.656 | -0.003 | 28.598 | 1.448 | 358.287 | 0.302 | 0.280 | 126.402 | 86.549 | 126.807 | 0.048 | 143.055 | 2 | train |
| 0.021 | 163.600 | 171.578 | 163.174 | 0.044 | 88.659 | 1.476 | 461.523 | 0.265 | 0.220 | 162.933 | 110.416 | 163.261 | 0.044 | 59.100 | 0 | train |
| 0.017 | 160.890 | 170.250 | 160.182 | -0.012 | 52.264 | 1.473 | 453.399 | 0.293 | 0.261 | 159.934 | 109.292 | 160.402 | 0.046 | 130.555 | 1 | train |
| 0.015 | 157.960 | 170.359 | 156.929 | -0.021 | 36.522 | 1.377 | 444.241 | 0.301 | 0.328 | 156.590 | 107.564 | 157.097 | 0.054 | 136.058 | 0 | train |
| 0.016 | 166.619 | 181.825 | 163.822 | 0.059 | 39.064 | 0.831 | 463.319 | 0.327 | 0.300 | 162.638 | 107.877 | 164.451 | 0.025 | 50.961 | 0 | train |
| 0.018 | 152.684 | 163.562 | 151.855 | -0.007 | 17.505 | 1.405 | 429.709 | 0.303 | 0.290 | 151.583 | 103.654 | 152.054 | 0.056 | 116.046 | 1 | train |
| 0.020 | 158.672 | 167.703 | 158.140 | 0.025 | 65.008 | 1.480 | 447.057 | 0.284 | 0.297 | 157.942 | 108.574 | 158.197 | 0.045 | 79.330 | 0 | train |
| 0.017 | 147.339 | 164.462 | 143.162 | 0.026 | 41.959 | 0.656 | 405.258 | 0.342 | 0.316 | 141.468 | 90.716 | 143.972 | 0.052 | 85.368 | 2 | train |
| 0.018 | 167.900 | 176.203 | 167.356 | -0.003 | 39.531 | 1.824 | 473.620 | 0.316 | 0.257 | 167.154 | 114.204 | 167.444 | 0.036 | 98.573 | 0 | train |
| 0.019 | 156.187 | 170.442 | 155.103 | -0.010 | 27.591 | 1.476 | 439.119 | 0.318 | 0.309 | 154.768 | 104.426 | 155.385 | 0.063 | 140.616 | 0 | train |
| 0.022 | 150.733 | 160.297 | 150.259 | 0.005 | 40.306 | 1.543 | 424.820 | 0.269 | 0.284 | 150.181 | 101.889 | 150.320 | 0.048 | 108.886 | 1 | train |
| 0.017 | 147.599 | 158.969 | 146.740 | 0.007 | 21.358 | 1.426 | 415.157 | 0.297 | 0.293 | 146.409 | 99.532 | 146.934 | 0.035 | 106.220 | 1 | train |
| 0.023 | 161.335 | 169.828 | 160.760 | -0.010 | 95.838 | 1.452 | 454.885 | 0.283 | 0.292 | 160.618 | 110.753 | 160.883 | 0.099 | 94.454 | 0 | train |
| 0.017 | 160.511 | 169.391 | 159.748 | -0.009 | 80.142 | 1.365 | 452.166 | 0.308 | 0.290 | 159.471 | 107.954 | 159.906 | 0.048 | 105.384 | 0 | train |
| 0.016 | 155.910 | 165.562 | 155.084 | -0.020 | 18.276 | 1.486 | 438.630 | 0.319 | 0.294 | 154.827 | 105.247 | 155.289 | 0.052 | 118.891 | 0 | train |
| 0.020 | 156.855 | 165.953 | 156.341 | -0.006 | 78.561 | 1.829 | 442.483 | 0.269 | 0.241 | 156.167 | 107.336 | 156.472 | 0.049 | 112.890 | 0 | train |
| 0.021 | 165.464 | 174.259 | 165.050 | 0.011 | 49.581 | 1.486 | 467.073 | 0.293 | 0.268 | 164.870 | 113.057 | 165.169 | 0.075 | 116.246 | 0 | train |
| 0.020 | 168.550 | 177.219 | 168.246 | -0.001 | 98.037 | 1.520 | 476.221 | 0.291 | 0.253 | 168.163 | 114.062 | 168.284 | 0.062 | 94.675 | 0 | train |
| 0.020 | 161.567 | 169.719 | 160.984 | -0.009 | 55.633 | 1.467 | 455.515 | 0.303 | 0.251 | 160.739 | 110.079 | 161.144 | 0.044 | 100.549 | 0 | train |
| 0.022 | 162.223 | 170.594 | 161.801 | -0.023 | 44.704 | 1.664 | 457.501 | 0.311 | 0.279 | 161.535 | 109.734 | 161.831 | 0.097 | 146.501 | 0 | train |
| 0.016 | 162.461 | 171.453 | 161.953 | -0.003 | 44.712 | 1.491 | 457.719 | 0.298 | 0.295 | 161.873 | 111.096 | 161.957 | 0.098 | 152.910 | 0 | train |
| 0.016 | 124.703 | 137.078 | 123.491 | -0.006 | 19.372 | 1.542 | 349.431 | 0.306 | 0.251 | 123.139 | 82.825 | 123.786 | 0.035 | 113.135 | 2 | train |
| 0.032 | 161.367 | 169.500 | 160.930 | 0.023 | 121.894 | 1.516 | 455.693 | 0.267 | 0.306 | 160.659 | 110.003 | 160.962 | 0.109 | 100.051 | 0 | train |
| 0.017 | 138.051 | 149.766 | 137.523 | -0.001 | 38.045 | 1.455 | 389.001 | 0.272 | 0.257 | 137.301 | 94.045 | 137.612 | 0.084 | 166.296 | 1 | train |
| 0.018 | 145.059 | 156.547 | 143.575 | -0.009 | 28.869 | 1.379 | 406.209 | 0.313 | 0.273 | 143.039 | 97.120 | 144.055 | 0.043 | 105.107 | 1 | train |
| 0.023 | 163.037 | 170.688 | 162.915 | 0.005 | 50.711 | 1.510 | 459.744 | 0.274 | 0.268 | 162.973 | 110.674 | 162.732 | 0.086 | 114.293 | 1 | train |
| 0.023 | 167.632 | 175.734 | 167.084 | -0.008 | 79.776 | 1.551 | 472.588 | 0.295 | 0.248 | 166.894 | 114.103 | 167.255 | 0.073 | 103.030 | 0 | train |
| 0.016 | 154.861 | 164.922 | 154.267 | -0.023 | 21.977 | 1.500 | 436.309 | 0.300 | 0.273 | 154.076 | 105.253 | 154.385 | 0.051 | 131.416 | 1 | train |
| 0.018 | 149.460 | 159.875 | 148.753 | -0.011 | 55.438 | 1.566 | 420.708 | 0.308 | 0.253 | 148.526 | 101.242 | 148.946 | 0.060 | 145.519 | 1 | train |
| 0.018 | 169.433 | 178.688 | 168.764 | -0.014 | 62.495 | 1.530 | 477.702 | 0.312 | 0.284 | 168.451 | 115.878 | 168.879 | 0.082 | 142.011 | 0 | train |
| 0.021 | 159.235 | 167.375 | 158.729 | -0.015 | 75.247 | 1.565 | 449.452 | 0.292 | 0.282 | 158.507 | 108.412 | 158.819 | 0.018 | 46.912 | 1 | train |
| 0.017 | 153.710 | 163.688 | 153.213 | 0.009 | 49.519 | 1.532 | 433.037 | 0.284 | 0.276 | 153.038 | 103.159 | 153.208 | 0.052 | 107.733 | 0 | train |
| 0.019 | 169.435 | 177.844 | 169.031 | 0.006 | 68.721 | 1.644 | 478.271 | 0.274 | 0.244 | 168.896 | 115.638 | 169.101 | 0.058 | 144.435 | 0 | train |
| 0.019 | 148.182 | 158.500 | 147.676 | -0.008 | 55.295 | 1.483 | 417.550 | 0.286 | 0.267 | 147.467 | 100.109 | 147.727 | 0.058 | 140.477 | 1 | train |
| 0.019 | 160.702 | 168.016 | 160.398 | 0.027 | 39.360 | 1.537 | 453.577 | 0.259 | 0.249 | 160.284 | 109.808 | 160.402 | 0.096 | 122.496 | 1 | train |
| 0.016 | 149.159 | 160.781 | 148.049 | -0.015 | 44.301 | 1.470 | 419.040 | 0.298 | 0.284 | 147.552 | 99.980 | 148.379 | 0.036 | 101.547 | 1 | train |
| 0.016 | 161.897 | 172.750 | 160.902 | -0.011 | 74.255 | 1.502 | 455.491 | 0.310 | 0.284 | 160.708 | 108.408 | 161.110 | 0.053 | 127.704 | 0 | train |
| 0.018 | 153.900 | 161.953 | 153.508 | 0.024 | 60.363 | 1.516 | 434.179 | 0.260 | 0.301 | 153.283 | 104.791 | 153.497 | 0.048 | 161.458 | 1 | train |
| 0.018 | 157.062 | 164.469 | 156.529 | 0.018 | 49.136 | 1.435 | 442.699 | 0.290 | 0.287 | 156.337 | 106.820 | 156.591 | 0.111 | 184.555 | 0 | train |
| 0.017 | 156.885 | 166.547 | 156.411 | -0.013 | 70.251 | 1.552 | 442.912 | 0.301 | 0.260 | 156.190 | 106.640 | 156.462 | 0.018 | 74.491 | 1 | train |
| 0.018 | 163.360 | 172.250 | 162.812 | 0.028 | 60.670 | 1.508 | 460.530 | 0.298 | 0.251 | 162.594 | 110.863 | 162.949 | 0.099 | 182.579 | 0 | train |
| 0.015 | 146.976 | 156.062 | 145.935 | -0.009 | 20.188 | 1.383 | 412.875 | 0.313 | 0.307 | 145.528 | 99.239 | 146.170 | 0.034 | 99.632 | 1 | train |
| 0.021 | 162.313 | 170.953 | 161.862 | -0.037 | 50.731 | 1.563 | 458.413 | 0.266 | 0.274 | 161.646 | 110.305 | 162.023 | 0.087 | 140.349 | 0 | train |
| 0.022 | 151.080 | 161.125 | 150.899 | -0.004 | 72.023 | 1.534 | 427.204 | 0.251 | 0.248 | 150.814 | 103.633 | 150.877 | 0.110 | 112.774 | 0 | train |
| 0.020 | 152.076 | 163.000 | 151.214 | -0.036 | 33.685 | 1.554 | 427.921 | 0.293 | 0.294 | 150.910 | 102.359 | 151.484 | 0.054 | 132.528 | 1 | train |
| 0.020 | 156.327 | 164.312 | 155.960 | -0.006 | 51.889 | 1.487 | 440.820 | 0.267 | 0.316 | 155.833 | 107.430 | 155.937 | 0.044 | 104.448 | 1 | train |
| 0.017 | 125.520 | 136.500 | 124.547 | 0.026 | 31.084 | 1.481 | 352.428 | 0.292 | 0.289 | 124.122 | 84.378 | 124.725 | 0.031 | 94.282 | 2 | train |
| 0.017 | 152.675 | 162.344 | 151.813 | -0.023 | 33.407 | 1.415 | 429.586 | 0.300 | 0.292 | 151.516 | 102.530 | 152.046 | 0.054 | 142.016 | 1 | train |
| 0.021 | 157.460 | 165.297 | 157.074 | 0.029 | 78.937 | 1.462 | 444.135 | 0.267 | 0.269 | 156.889 | 106.884 | 157.082 | 0.050 | 93.586 | 0 | train |
| 0.024 | 152.600 | 161.062 | 152.160 | -0.007 | 64.532 | 1.508 | 430.433 | 0.254 | 0.253 | 151.975 | 103.517 | 152.207 | 0.046 | 98.174 | 1 | train |
| 0.020 | 159.565 | 168.672 | 159.060 | 0.015 | 23.418 | 1.455 | 450.051 | 0.280 | 0.279 | 158.746 | 108.407 | 159.131 | 0.046 | 103.266 | 1 | train |
| 0.016 | 160.463 | 169.344 | 159.834 | -0.004 | 63.978 | 1.423 | 452.179 | 0.297 | 0.336 | 159.623 | 108.196 | 159.861 | 0.048 | 99.501 | 0 | train |
| 0.021 | 155.712 | 166.062 | 155.167 | -0.028 | 47.941 | 1.500 | 439.033 | 0.279 | 0.291 | 155.018 | 104.927 | 155.195 | 0.047 | 96.490 | 1 | train |
| 0.020 | 158.815 | 167.297 | 158.251 | 0.010 | 67.403 | 1.569 | 447.693 | 0.297 | 0.253 | 158.032 | 107.699 | 158.409 | 0.047 | 106.249 | 0 | train |
| 0.016 | 126.306 | 137.281 | 125.500 | -0.024 | 43.549 | 1.519 | 355.238 | 0.286 | 0.275 | 125.231 | 84.706 | 125.752 | 0.028 | 99.687 | 2 | train |
| 0.018 | 118.384 | 128.328 | 117.755 | 0.018 | 43.588 | 1.469 | 332.939 | 0.284 | 0.295 | 117.525 | 79.681 | 117.771 | 0.035 | 89.649 | 2 | train |
| 0.018 | 149.149 | 157.109 | 148.462 | 0.005 | 29.391 | 1.507 | 419.957 | 0.294 | 0.264 | 148.204 | 100.483 | 148.667 | 0.029 | 121.181 | 1 | train |
| 0.021 | 157.768 | 167.578 | 156.904 | 0.005 | 48.837 | 1.488 | 443.979 | 0.298 | 0.261 | 156.592 | 107.143 | 157.182 | 0.029 | 59.730 | 0 | train |
| 0.018 | 155.992 | 166.562 | 155.309 | -0.001 | 36.091 | 1.468 | 439.545 | 0.295 | 0.287 | 155.076 | 105.921 | 155.485 | 0.052 | 147.088 | 1 | train |
| 0.022 | 157.045 | 166.281 | 156.619 | 0.004 | 64.247 | 1.505 | 442.877 | 0.264 | 0.251 | 156.455 | 105.625 | 156.667 | 0.048 | 76.615 | 0 | train |
| 0.014 | 136.359 | 145.891 | 135.632 | -0.004 | 32.456 | 1.380 | 383.712 | 0.294 | 0.316 | 135.372 | 92.887 | 135.736 | 0.025 | 73.964 | 2 | train |
| 0.023 | 155.874 | 164.509 | 155.208 | -0.001 | 78.724 | 1.457 | 439.045 | 0.291 | 0.305 | 155.044 | 105.360 | 155.305 | 0.051 | 83.579 | 0 | train |
| 0.018 | 152.430 | 162.844 | 151.427 | 0.003 | 58.208 | 1.650 | 428.636 | 0.300 | 0.292 | 151.012 | 102.605 | 151.662 | 0.031 | 117.453 | 1 | train |
| 0.016 | 164.428 | 172.547 | 163.878 | 0.019 | 46.664 | 1.482 | 463.674 | 0.299 | 0.258 | 163.698 | 112.078 | 164.012 | 0.092 | 147.249 | 0 | train |
| 0.017 | 156.564 | 167.875 | 155.372 | 0.015 | 35.576 | 1.490 | 439.561 | 0.316 | 0.255 | 154.990 | 105.823 | 155.621 | 0.033 | 80.770 | 0 | train |
| 0.020 | 165.253 | 176.781 | 163.685 | -0.006 | 60.674 | 1.211 | 463.191 | 0.316 | 0.313 | 163.184 | 109.085 | 163.993 | 0.029 | 45.997 | 0 | train |
| 0.017 | 161.964 | 179.188 | 158.409 | 0.082 | 50.497 | 0.788 | 448.323 | 0.326 | 0.284 | 156.856 | 102.715 | 159.345 | 0.037 | 81.585 | 1 | train |
| 0.016 | 159.572 | 168.219 | 159.073 | -0.008 | 16.903 | 1.457 | 450.016 | 0.282 | 0.252 | 158.900 | 109.288 | 159.211 | 0.045 | 109.442 | 0 | train |
| 0.024 | 162.093 | 175.141 | 160.154 | -0.002 | 103.876 | 1.151 | 453.465 | 0.329 | 0.345 | 159.567 | 106.640 | 160.405 | 0.020 | 37.164 | 0 | train |
| 0.018 | 161.676 | 170.312 | 161.204 | -0.014 | 41.382 | 1.455 | 456.397 | 0.275 | 0.283 | 161.003 | 110.304 | 161.313 | 0.093 | 111.575 | 0 | train |
| 0.016 | 154.677 | 163.441 | 153.946 | 0.001 | 40.145 | 1.421 | 435.711 | 0.301 | 0.284 | 153.690 | 105.106 | 154.098 | 0.050 | 120.706 | 1 | train |
| 0.013 | 150.408 | 158.656 | 149.831 | 0.001 | 55.428 | 1.482 | 423.697 | 0.290 | 0.287 | 149.633 | 102.633 | 149.904 | 0.053 | 204.438 | 1 | train |
| 0.022 | 158.265 | 166.531 | 157.677 | -0.001 | 50.941 | 1.503 | 446.482 | 0.270 | 0.276 | 157.314 | 107.468 | 157.867 | 0.026 | 63.046 | 1 | train |
| 0.014 | 127.297 | 144.562 | 126.662 | 0.007 | 22.971 | 1.433 | 358.391 | 0.290 | 0.298 | 126.416 | 83.953 | 126.725 | 0.057 | 129.365 | 2 | train |
| 0.017 | 132.992 | 143.219 | 132.435 | -0.006 | 10.336 | 1.447 | 374.775 | 0.291 | 0.275 | 132.262 | 89.271 | 132.579 | 0.098 | 151.132 | 2 | train |
| 0.020 | 159.145 | 171.078 | 158.737 | 0.004 | 60.846 | 1.522 | 449.104 | 0.279 | 0.282 | 158.612 | 109.144 | 158.813 | 0.091 | 131.579 | 0 | train |
| 0.026 | 164.404 | 171.719 | 164.085 | -0.012 | 70.673 | 1.489 | 464.070 | 0.271 | 0.257 | 163.983 | 111.777 | 164.137 | 0.077 | 104.357 | 1 | train |
| 0.016 | 148.486 | 158.812 | 147.411 | -0.007 | 36.357 | 1.391 | 416.916 | 0.299 | 0.263 | 147.039 | 100.491 | 147.645 | 0.036 | 112.011 | 1 | train |
| 0.022 | 156.028 | 164.797 | 155.658 | 0.013 | 65.652 | 1.545 | 440.442 | 0.268 | 0.278 | 155.486 | 105.687 | 155.734 | 0.106 | 160.876 | 0 | train |
| 0.017 | 165.794 | 175.172 | 165.168 | -0.015 | 51.479 | 1.505 | 467.304 | 0.287 | 0.281 | 164.822 | 114.273 | 165.322 | 0.086 | 113.128 | 0 | train |
| 0.020 | 162.521 | 171.750 | 161.980 | 0.020 | 68.855 | 1.450 | 458.241 | 0.293 | 0.269 | 161.771 | 111.040 | 162.079 | 0.042 | 118.175 | 0 | train |
| 0.022 | 160.126 | 168.061 | 159.657 | 0.012 | 78.609 | 1.531 | 451.945 | 0.275 | 0.242 | 159.382 | 108.040 | 159.773 | 0.096 | 132.123 | 0 | train |
| 0.020 | 154.605 | 165.250 | 154.090 | -0.014 | 45.411 | 1.490 | 436.250 | 0.262 | 0.276 | 153.901 | 104.463 | 154.180 | 0.048 | 105.479 | 1 | train |
| 0.021 | 155.679 | 163.969 | 155.130 | -0.012 | 47.434 | 1.533 | 438.620 | 0.288 | 0.289 | 154.906 | 105.384 | 155.239 | 0.048 | 102.484 | 1 | train |
| 0.023 | 171.724 | 185.141 | 169.827 | 0.019 | 92.888 | 1.138 | 480.802 | 0.337 | 0.307 | 169.038 | 112.361 | 170.244 | 0.018 | 35.954 | 0 | train |
| 0.021 | 151.208 | 160.375 | 150.196 | -0.028 | 28.824 | 1.396 | 425.077 | 0.301 | 0.282 | 149.891 | 101.745 | 150.450 | 0.031 | 61.880 | 1 | train |
| 0.023 | 151.894 | 166.031 | 148.986 | 0.028 | 47.024 | 0.898 | 421.592 | 0.313 | 0.287 | 147.832 | 95.939 | 149.904 | 0.032 | 42.156 | 1 | train |
| 0.019 | 164.354 | 175.469 | 162.739 | -0.003 | 56.837 | 1.272 | 460.623 | 0.338 | 0.309 | 162.223 | 110.820 | 163.200 | 0.058 | 78.903 | 0 | train |
| 0.019 | 155.976 | 170.109 | 153.120 | 0.022 | 84.706 | 0.894 | 433.534 | 0.316 | 0.304 | 151.982 | 101.261 | 153.939 | 0.030 | 50.315 | 0 | train |
| 0.029 | 159.119 | 172.031 | 157.024 | 0.011 | 46.913 | 0.668 | 443.203 | 0.351 | 0.311 | 155.897 | 103.471 | 157.141 | 0.024 | 73.002 | 1 | train |
| 0.019 | 159.199 | 166.531 | 158.785 | 0.020 | 51.353 | 1.619 | 449.711 | 0.262 | 0.255 | 158.583 | 109.045 | 158.927 | 0.106 | 169.649 | 0 | train |
| 0.020 | 142.035 | 154.062 | 141.203 | 0.001 | 32.399 | 1.495 | 399.295 | 0.293 | 0.264 | 140.934 | 96.048 | 141.439 | 0.040 | 87.846 | 1 | train |
| 0.016 | 132.216 | 140.156 | 131.439 | 0.001 | 15.470 | 1.451 | 371.897 | 0.282 | 0.250 | 131.194 | 89.771 | 131.660 | 0.045 | 127.533 | 2 | train |
| 0.020 | 163.109 | 171.984 | 162.572 | -0.010 | 84.336 | 1.576 | 459.789 | 0.309 | 0.253 | 162.342 | 111.200 | 162.703 | 0.043 | 84.505 | 0 | train |
| 0.018 | 154.493 | 162.266 | 153.987 | -0.028 | 41.673 | 1.476 | 436.005 | 0.272 | 0.285 | 153.798 | 105.463 | 154.104 | 0.046 | 114.235 | 1 | train |
| 0.018 | 150.814 | 163.031 | 150.171 | -0.007 | 44.055 | 1.536 | 425.007 | 0.302 | 0.311 | 149.951 | 102.702 | 150.303 | 0.058 | 146.397 | 1 | train |
| 0.021 | 158.083 | 167.312 | 157.675 | -0.006 | 44.798 | 1.534 | 446.005 | 0.278 | 0.238 | 157.493 | 107.799 | 157.745 | 0.046 | 83.040 | 0 | train |
| 0.017 | 157.114 | 165.438 | 156.705 | 0.043 | 49.327 | 1.469 | 443.556 | 0.269 | 0.292 | 156.427 | 106.669 | 156.742 | 0.047 | 95.423 | 1 | train |
| 0.018 | 142.336 | 157.234 | 141.132 | -0.001 | 35.452 | 1.452 | 399.297 | 0.301 | 0.269 | 140.708 | 94.936 | 141.511 | 0.045 | 117.908 | 1 | train |
| 0.022 | 143.047 | 152.844 | 142.393 | -0.015 | 48.784 | 1.492 | 402.890 | 0.277 | 0.247 | 142.154 | 96.951 | 142.585 | 0.077 | 136.623 | 1 | train |
| 0.019 | 146.368 | 156.125 | 145.655 | 0.008 | 36.877 | 1.512 | 412.018 | 0.280 | 0.260 | 145.341 | 99.800 | 145.845 | 0.072 | 143.723 | 1 | train |
| 0.016 | 153.015 | 164.797 | 152.396 | 0.005 | 52.075 | 1.578 | 431.086 | 0.285 | 0.255 | 152.166 | 102.816 | 152.485 | 0.053 | 103.133 | 0 | train |
| 0.017 | 153.011 | 163.203 | 152.445 | -0.006 | 59.840 | 1.577 | 430.951 | 0.294 | 0.260 | 152.310 | 103.436 | 152.535 | 0.056 | 122.668 | 0 | train |
| 0.018 | 155.718 | 166.322 | 154.977 | 0.001 | 37.983 | 1.497 | 438.398 | 0.295 | 0.271 | 154.647 | 106.739 | 155.170 | 0.051 | 128.378 | 1 | train |
| 0.020 | 162.231 | 169.783 | 161.698 | -0.019 | 66.263 | 1.473 | 457.425 | 0.287 | 0.272 | 161.540 | 112.199 | 161.808 | 0.104 | 118.439 | 0 | train |
| 0.020 | 134.641 | 142.203 | 134.006 | -0.031 | 32.234 | 1.425 | 379.358 | 0.287 | 0.257 | 133.819 | 91.131 | 134.180 | 0.089 | 115.465 | 2 | train |
| 0.019 | 153.217 | 161.663 | 152.624 | 0.007 | 37.531 | 1.473 | 431.432 | 0.311 | 0.293 | 152.478 | 103.571 | 152.733 | 0.049 | 133.351 | 1 | train |
| 0.020 | 157.139 | 166.906 | 156.563 | -0.020 | 66.387 | 1.453 | 443.015 | 0.291 | 0.294 | 156.345 | 106.965 | 156.682 | 0.106 | 199.517 | 1 | train |
| 0.020 | 150.917 | 158.562 | 150.402 | -0.009 | 45.445 | 1.422 | 425.776 | 0.268 | 0.262 | 150.173 | 102.555 | 150.535 | 0.118 | 162.261 | 0 | train |
| 0.022 | 153.009 | 161.578 | 152.618 | -0.017 | 73.038 | 1.709 | 431.689 | 0.261 | 0.213 | 152.450 | 104.579 | 152.722 | 0.044 | 85.473 | 0 | train |
| 0.022 | 154.141 | 162.906 | 153.532 | 0.037 | 12.939 | 1.411 | 434.280 | 0.299 | 0.266 | 153.328 | 104.008 | 153.645 | 0.050 | 85.016 | 1 | train |
| 0.018 | 148.375 | 158.234 | 147.779 | 0.005 | 38.608 | 1.543 | 418.061 | 0.280 | 0.255 | 147.557 | 100.609 | 147.938 | 0.056 | 146.764 | 1 | train |
| 0.028 | 161.558 | 174.936 | 159.661 | 0.011 | 42.009 | 1.086 | 451.293 | 0.328 | 0.331 | 159.063 | 105.931 | 159.897 | 0.022 | 26.579 | 1 | train |
| 0.017 | 152.532 | 166.598 | 149.516 | 0.025 | 34.003 | 0.886 | 422.688 | 0.350 | 0.322 | 148.413 | 98.112 | 149.996 | 0.035 | 62.960 | 1 | train |
| 0.020 | 153.672 | 162.719 | 153.188 | -0.003 | 40.955 | 1.460 | 433.218 | 0.271 | 0.269 | 153.082 | 104.593 | 153.272 | 0.111 | 136.752 | 0 | train |
| 0.019 | 163.109 | 179.791 | 159.747 | 0.054 | 64.570 | 0.734 | 452.167 | 0.334 | 0.355 | 158.393 | 104.966 | 160.103 | 0.032 | 62.602 | 0 | train |
| 0.023 | 154.546 | 166.250 | 152.782 | -0.023 | 69.004 | 1.220 | 432.860 | 0.324 | 0.323 | 152.176 | 103.428 | 153.154 | 0.022 | 47.746 | 0 | train |
| 0.021 | 154.761 | 163.656 | 154.211 | -0.001 | 47.942 | 1.572 | 436.400 | 0.266 | 0.243 | 153.872 | 106.376 | 154.374 | 0.048 | 104.097 | 1 | train |
| 0.019 | 164.751 | 173.203 | 164.368 | 0.017 | 61.818 | 1.553 | 465.082 | 0.272 | 0.266 | 164.168 | 111.605 | 164.428 | 0.084 | 136.446 | 0 | train |
| 0.019 | 157.631 | 167.750 | 156.724 | 0.001 | 32.457 | 1.424 | 443.283 | 0.296 | 0.283 | 156.284 | 107.151 | 156.901 | 0.028 | 73.487 | 1 | train |
| 0.018 | 151.671 | 163.094 | 150.736 | -0.025 | 26.928 | 1.432 | 426.483 | 0.290 | 0.265 | 150.430 | 102.239 | 151.013 | 0.064 | 109.859 | 1 | train |
| 0.024 | 158.153 | 166.984 | 157.548 | -0.021 | 98.159 | 1.548 | 445.709 | 0.288 | 0.306 | 157.354 | 106.911 | 157.635 | 0.049 | 102.929 | 0 | train |
| 0.017 | 149.872 | 158.484 | 149.416 | 0.003 | 28.390 | 1.410 | 422.405 | 0.282 | 0.298 | 149.265 | 101.432 | 149.436 | 0.048 | 161.601 | 1 | train |
| 0.019 | 158.049 | 166.312 | 157.482 | -0.019 | 38.158 | 1.466 | 445.693 | 0.285 | 0.299 | 157.290 | 107.058 | 157.604 | 0.111 | 207.890 | 0 | train |
| 0.016 | 158.514 | 167.734 | 157.860 | -0.022 | 36.460 | 1.424 | 446.384 | 0.292 | 0.319 | 157.665 | 107.076 | 157.901 | 0.048 | 99.756 | 1 | train |
| 0.017 | 158.139 | 166.500 | 157.681 | 0.015 | 31.936 | 1.596 | 445.672 | 0.285 | 0.261 | 157.531 | 107.587 | 157.760 | 0.048 | 95.678 | 0 | train |
| 0.027 | 159.013 | 168.375 | 158.393 | -0.001 | 32.061 | 1.484 | 448.256 | 0.300 | 0.283 | 158.165 | 108.824 | 158.554 | 0.112 | 112.918 | 0 | train |
| 0.021 | 158.569 | 166.227 | 158.298 | 0.017 | 34.435 | 1.538 | 447.513 | 0.262 | 0.273 | 158.175 | 107.946 | 158.290 | 0.094 | 150.823 | 0 | train |
| 0.022 | 151.457 | 159.312 | 151.024 | -0.025 | 27.914 | 1.444 | 426.804 | 0.278 | 0.296 | 150.970 | 102.806 | 151.015 | 0.121 | 160.426 | 1 | train |
| 0.020 | 152.714 | 161.781 | 152.040 | -0.023 | 20.969 | 1.434 | 429.957 | 0.289 | 0.292 | 151.841 | 105.558 | 152.147 | 0.052 | 90.091 | 0 | train |
| 0.020 | 155.155 | 164.266 | 154.414 | -0.006 | 41.492 | 1.387 | 436.649 | 0.294 | 0.318 | 154.148 | 105.040 | 154.458 | 0.050 | 131.923 | 1 | train |
| 0.018 | 153.227 | 163.016 | 152.645 | 0.007 | 28.601 | 1.427 | 431.706 | 0.307 | 0.280 | 152.412 | 103.834 | 152.718 | 0.128 | 171.228 | 1 | train |
| 0.023 | 159.741 | 166.578 | 159.352 | 0.010 | 55.258 | 1.441 | 450.877 | 0.284 | 0.252 | 159.205 | 109.290 | 159.430 | 0.045 | 74.102 | 1 | train |
| 0.021 | 158.082 | 166.172 | 157.665 | -0.016 | 42.712 | 1.466 | 446.194 | 0.272 | 0.254 | 157.471 | 109.021 | 157.756 | 0.047 | 82.674 | 0 | train |
| 0.019 | 153.159 | 163.155 | 152.067 | 0.008 | 26.529 | 1.443 | 430.410 | 0.302 | 0.271 | 151.683 | 103.442 | 152.368 | 0.032 | 85.180 | 1 | train |
| 0.017 | 155.391 | 166.281 | 154.275 | 0.004 | 14.758 | 1.372 | 436.626 | 0.297 | 0.290 | 153.879 | 104.563 | 154.549 | 0.032 | 101.582 | 1 | train |
| 0.023 | 155.795 | 162.719 | 155.416 | 0.005 | 34.354 | 1.472 | 439.570 | 0.256 | 0.251 | 155.274 | 106.302 | 155.492 | 0.109 | 131.840 | 0 | train |
| 0.022 | 161.460 | 169.141 | 160.890 | 0.015 | 30.422 | 1.525 | 454.823 | 0.310 | 0.278 | 160.781 | 108.557 | 160.988 | 0.047 | 67.794 | 0 | train |
| 0.018 | 156.724 | 164.516 | 156.159 | -0.007 | 53.335 | 1.430 | 442.135 | 0.278 | 0.280 | 155.905 | 107.135 | 156.294 | 0.108 | 144.479 | 1 | train |
| 0.019 | 160.507 | 169.891 | 159.938 | -0.012 | 47.047 | 1.417 | 452.542 | 0.299 | 0.265 | 159.696 | 108.779 | 160.052 | 0.104 | 159.775 | 0 | train |
| 0.017 | 158.310 | 167.688 | 157.693 | 0.026 | 26.663 | 1.424 | 445.935 | 0.291 | 0.312 | 157.400 | 107.163 | 157.759 | 0.050 | 90.397 | 0 | train |
| 0.022 | 157.088 | 165.281 | 156.737 | 0.005 | 47.034 | 1.550 | 443.125 | 0.259 | 0.272 | 156.601 | 107.322 | 156.754 | 0.105 | 118.818 | 0 | train |
| 0.023 | 156.996 | 165.750 | 156.489 | -0.006 | 43.833 | 1.464 | 442.758 | 0.280 | 0.273 | 156.286 | 107.311 | 156.633 | 0.113 | 132.709 | 1 | train |
| 0.019 | 167.964 | 183.984 | 165.378 | 0.051 | 46.397 | 0.841 | 467.997 | 0.327 | 0.306 | 164.270 | 108.801 | 166.008 | 0.025 | 36.392 | 0 | train |
| 0.017 | 153.195 | 163.938 | 152.296 | 0.005 | 29.935 | 1.490 | 430.811 | 0.296 | 0.261 | 151.990 | 103.055 | 152.542 | 0.061 | 136.706 | 1 | train |
| 0.018 | 162.614 | 171.469 | 161.960 | -0.006 | 61.912 | 1.602 | 458.262 | 0.280 | 0.237 | 161.713 | 110.043 | 162.152 | 0.044 | 98.996 | 0 | train |
| 0.019 | 156.284 | 164.438 | 155.663 | -0.020 | 55.796 | 1.512 | 440.431 | 0.279 | 0.232 | 155.439 | 105.315 | 155.855 | 0.048 | 100.243 | 1 | train |
| 0.020 | 152.679 | 161.363 | 152.223 | -0.011 | 25.405 | 1.494 | 430.441 | 0.274 | 0.251 | 152.102 | 103.244 | 152.277 | 0.047 | 119.342 | 1 | train |
| 0.023 | 154.848 | 162.422 | 154.367 | 0.003 | 54.047 | 1.500 | 436.649 | 0.291 | 0.249 | 154.194 | 105.688 | 154.468 | 0.109 | 163.778 | 1 | train |
| 0.025 | 160.198 | 167.156 | 159.798 | 0.024 | 54.875 | 1.480 | 452.692 | 0.259 | 0.257 | 159.616 | 110.372 | 159.927 | 0.097 | 117.593 | 0 | train |
| 0.020 | 161.495 | 169.406 | 161.019 | 0.008 | 48.737 | 1.446 | 455.558 | 0.284 | 0.244 | 160.817 | 109.896 | 161.154 | 0.099 | 95.135 | 0 | train |
| 0.021 | 156.421 | 164.656 | 156.001 | 0.052 | 34.879 | 1.541 | 440.984 | 0.284 | 0.281 | 155.804 | 107.001 | 156.037 | 0.046 | 95.273 | 1 | train |
| 0.018 | 146.101 | 153.969 | 145.470 | 0.022 | 16.980 | 1.441 | 411.474 | 0.297 | 0.241 | 145.270 | 97.719 | 145.634 | 0.031 | 85.447 | 1 | train |
| 0.019 | 152.400 | 161.094 | 151.545 | -0.005 | 49.624 | 1.439 | 428.640 | 0.309 | 0.269 | 151.285 | 103.184 | 151.784 | 0.056 | 142.637 | 1 | train |
| 0.019 | 155.252 | 165.422 | 154.372 | -0.002 | 30.931 | 1.511 | 436.699 | 0.283 | 0.268 | 154.108 | 104.807 | 154.612 | 0.055 | 127.211 | 1 | train |
| 0.019 | 160.676 | 168.844 | 160.223 | -0.012 | 32.193 | 1.495 | 453.566 | 0.277 | 0.265 | 160.033 | 110.111 | 160.332 | 0.098 | 134.774 | 0 | train |
| 0.017 | 146.872 | 155.609 | 146.401 | 0.012 | 21.221 | 1.365 | 414.187 | 0.288 | 0.269 | 146.204 | 98.510 | 146.457 | 0.055 | 116.148 | 1 | train |
| 0.024 | 159.213 | 167.031 | 158.702 | 0.032 | 52.344 | 1.403 | 449.025 | 0.293 | 0.287 | 158.449 | 108.425 | 158.791 | 0.047 | 82.128 | 0 | train |
| 0.021 | 158.917 | 168.047 | 158.261 | 0.003 | 43.964 | 1.443 | 447.455 | 0.293 | 0.293 | 158.067 | 108.149 | 158.340 | 0.049 | 85.171 | 0 | train |
| 0.015 | 144.113 | 155.562 | 142.830 | 0.005 | 24.996 | 1.391 | 403.968 | 0.310 | 0.277 | 142.362 | 96.852 | 143.136 | 0.025 | 91.153 | 1 | train |
| 0.023 | 149.598 | 157.125 | 149.145 | -0.020 | 49.188 | 1.489 | 421.848 | 0.255 | 0.249 | 149.046 | 101.000 | 149.269 | 0.047 | 107.145 | 1 | train |
| 0.019 | 148.127 | 159.938 | 147.425 | -0.027 | 37.446 | 1.468 | 417.448 | 0.302 | 0.294 | 147.220 | 100.528 | 147.620 | 0.067 | 125.996 | 1 | train |
| 0.018 | 147.020 | 155.105 | 146.535 | 0.000 | 82.078 | 1.440 | 414.268 | 0.278 | 0.299 | 146.360 | 100.377 | 146.542 | 0.056 | 141.230 | 1 | train |
| 0.017 | 155.547 | 164.062 | 154.958 | -0.002 | 47.242 | 1.477 | 438.083 | 0.298 | 0.265 | 154.764 | 105.580 | 155.049 | 0.051 | 123.585 | 1 | train |
| 0.016 | 150.881 | 160.875 | 150.129 | 0.009 | 24.117 | 1.416 | 424.949 | 0.289 | 0.304 | 149.850 | 102.180 | 150.286 | 0.030 | 88.412 | 1 | train |
| 0.020 | 152.547 | 167.312 | 149.456 | 0.049 | 38.406 | 0.846 | 422.846 | 0.315 | 0.331 | 148.317 | 97.891 | 150.025 | 0.035 | 56.619 | 1 | train |
| 0.019 | 158.962 | 171.588 | 156.985 | 0.003 | 48.009 | 1.123 | 443.888 | 0.336 | 0.317 | 156.186 | 106.241 | 157.466 | 0.022 | 43.670 | 0 | train |
| 0.020 | 156.563 | 174.031 | 153.031 | 0.073 | 47.436 | 0.744 | 432.967 | 0.327 | 0.365 | 151.622 | 99.152 | 153.129 | 0.038 | 57.473 | 1 | train |
| 0.020 | 159.676 | 167.391 | 159.341 | -0.008 | 52.451 | 1.476 | 450.488 | 0.277 | 0.280 | 159.205 | 108.449 | 159.343 | 0.095 | 160.914 | 1 | train |
| 0.020 | 161.312 | 170.750 | 160.784 | 0.002 | 23.542 | 1.448 | 454.740 | 0.280 | 0.281 | 160.604 | 109.710 | 160.856 | 0.044 | 81.440 | 0 | train |
| 0.021 | 156.307 | 165.219 | 155.817 | -0.017 | 59.458 | 1.569 | 440.984 | 0.272 | 0.240 | 155.596 | 105.783 | 155.947 | 0.046 | 79.539 | 0 | train |
| 0.017 | 141.148 | 153.375 | 140.309 | 0.009 | 23.672 | 1.416 | 397.451 | 0.294 | 0.283 | 139.952 | 95.527 | 140.561 | 0.082 | 168.884 | 1 | train |
| 0.023 | 160.372 | 168.062 | 159.910 | -0.024 | 29.218 | 1.456 | 452.205 | 0.278 | 0.284 | 159.742 | 108.300 | 160.004 | 0.101 | 122.909 | 0 | train |
| 0.020 | 141.681 | 151.562 | 140.692 | -0.007 | 59.717 | 1.584 | 397.935 | 0.303 | 0.263 | 140.343 | 95.616 | 141.052 | 0.039 | 84.394 | 1 | train |
| 0.022 | 133.233 | 143.031 | 132.614 | 0.014 | 35.343 | 1.478 | 374.993 | 0.283 | 0.291 | 132.374 | 89.779 | 132.679 | 0.100 | 181.568 | 2 | train |
| 0.021 | 164.426 | 172.391 | 164.077 | -0.016 | 67.001 | 1.511 | 464.230 | 0.275 | 0.260 | 163.929 | 112.317 | 164.160 | 0.075 | 110.979 | 0 | train |
| 0.017 | 162.757 | 179.469 | 159.270 | 0.063 | 30.310 | 0.776 | 450.559 | 0.340 | 0.344 | 157.886 | 103.362 | 159.555 | 0.032 | 65.916 | 0 | train |
| 0.020 | 154.177 | 163.075 | 153.801 | -0.002 | 44.753 | 1.444 | 435.199 | 0.271 | 0.257 | 153.661 | 106.050 | 153.882 | 0.045 | 101.818 | 1 | train |
| 0.019 | 153.508 | 163.500 | 153.109 | -0.017 | 39.737 | 1.580 | 432.945 | 0.268 | 0.257 | 153.037 | 104.591 | 153.186 | 0.047 | 103.647 | 0 | train |
| 0.019 | 134.357 | 146.167 | 133.714 | 0.008 | 31.794 | 1.538 | 378.336 | 0.295 | 0.255 | 133.525 | 91.021 | 133.887 | 0.041 | 111.412 | 2 | train |
| 0.017 | 158.175 | 167.125 | 157.394 | 0.004 | 25.178 | 1.429 | 445.234 | 0.298 | 0.299 | 157.141 | 107.681 | 157.534 | 0.050 | 116.047 | 1 | train |
| 0.019 | 161.297 | 168.578 | 160.844 | 0.019 | 47.796 | 1.457 | 455.167 | 0.284 | 0.265 | 160.662 | 110.272 | 160.933 | 0.097 | 162.499 | 0 | train |
| 0.017 | 161.118 | 167.891 | 160.840 | -0.005 | 60.275 | 1.531 | 454.756 | 0.269 | 0.255 | 160.871 | 110.783 | 160.858 | 0.090 | 104.653 | 0 | train |
| 0.021 | 154.544 | 163.875 | 153.883 | 0.002 | 41.519 | 1.458 | 435.218 | 0.297 | 0.288 | 153.688 | 104.435 | 154.032 | 0.048 | 111.544 | 1 | train |
| 0.019 | 156.687 | 165.281 | 156.290 | -0.002 | 46.965 | 1.467 | 441.921 | 0.283 | 0.270 | 156.113 | 106.360 | 156.312 | 0.046 | 131.004 | 1 | train |
| 0.020 | 152.030 | 160.094 | 151.375 | 0.007 | 45.768 | 1.496 | 428.076 | 0.306 | 0.257 | 151.132 | 103.473 | 151.531 | 0.027 | 97.238 | 1 | train |
| 0.015 | 151.050 | 163.531 | 149.753 | 0.005 | 30.976 | 1.396 | 423.954 | 0.294 | 0.279 | 149.296 | 100.791 | 150.140 | 0.037 | 105.657 | 1 | train |
| 0.019 | 156.576 | 164.938 | 155.830 | -0.011 | 33.278 | 1.420 | 440.776 | 0.306 | 0.280 | 155.596 | 105.946 | 155.968 | 0.053 | 108.261 | 1 | train |
| 0.024 | 149.667 | 159.245 | 149.273 | -0.014 | 51.161 | 1.565 | 422.172 | 0.260 | 0.282 | 149.094 | 100.558 | 149.328 | 0.049 | 107.008 | 1 | train |
| 0.022 | 162.393 | 170.344 | 161.968 | 0.010 | 73.483 | 1.494 | 458.483 | 0.286 | 0.264 | 161.767 | 111.540 | 162.043 | 0.095 | 140.231 | 0 | train |
| 0.023 | 165.158 | 172.453 | 164.750 | -0.031 | 49.440 | 1.504 | 466.015 | 0.278 | 0.265 | 164.609 | 111.879 | 164.835 | 0.077 | 95.750 | 0 | train |
| 0.021 | 148.303 | 156.669 | 147.958 | 0.021 | 60.695 | 1.493 | 418.328 | 0.263 | 0.250 | 147.822 | 100.968 | 147.970 | 0.052 | 94.554 | 1 | train |
| 0.020 | 142.731 | 153.375 | 142.053 | 0.023 | 25.357 | 1.511 | 401.685 | 0.298 | 0.284 | 141.902 | 96.703 | 142.184 | 0.080 | 138.319 | 1 | train |
| 0.022 | 157.222 | 166.828 | 156.646 | -0.006 | 74.298 | 1.553 | 443.381 | 0.279 | 0.286 | 156.390 | 105.814 | 156.785 | 0.047 | 107.805 | 1 | train |
| 0.024 | 159.090 | 165.828 | 158.799 | -0.027 | 42.965 | 1.496 | 448.817 | 0.281 | 0.252 | 158.781 | 109.340 | 158.801 | 0.093 | 114.397 | 0 | train |
| 0.021 | 152.797 | 161.695 | 152.147 | -0.004 | 73.807 | 1.464 | 430.389 | 0.288 | 0.246 | 151.934 | 103.137 | 152.294 | 0.052 | 99.474 | 1 | train |
| 0.021 | 160.462 | 170.828 | 159.633 | -0.003 | 39.197 | 1.532 | 451.695 | 0.308 | 0.288 | 159.385 | 108.354 | 159.760 | 0.050 | 122.748 | 0 | train |
| 0.022 | 159.945 | 168.125 | 159.393 | 0.004 | 40.038 | 1.488 | 450.916 | 0.278 | 0.298 | 159.146 | 108.552 | 159.481 | 0.045 | 89.705 | 0 | train |
| 0.018 | 151.022 | 160.719 | 150.379 | 0.032 | 22.904 | 1.637 | 425.490 | 0.280 | 0.236 | 150.116 | 102.944 | 150.542 | 0.057 | 122.205 | 1 | train |
| 0.019 | 147.623 | 157.250 | 146.968 | 0.007 | 41.154 | 1.575 | 415.779 | 0.279 | 0.266 | 146.753 | 100.063 | 147.136 | 0.030 | 102.897 | 1 | train |
| 0.019 | 161.025 | 171.328 | 160.348 | -0.004 | 15.455 | 1.445 | 453.704 | 0.287 | 0.303 | 160.098 | 107.791 | 160.467 | 0.048 | 99.918 | 0 | train |
| 0.019 | 149.245 | 157.656 | 148.649 | 0.027 | 42.824 | 1.502 | 420.441 | 0.283 | 0.245 | 148.428 | 101.266 | 148.815 | 0.056 | 99.148 | 1 | train |
| 0.017 | 162.264 | 174.469 | 161.624 | 0.010 | 20.867 | 1.556 | 457.322 | 0.293 | 0.293 | 161.402 | 109.847 | 161.749 | 0.025 | 91.225 | 0 | train |
| 0.018 | 153.280 | 162.969 | 152.582 | 0.006 | 27.633 | 1.460 | 431.762 | 0.292 | 0.278 | 152.362 | 104.466 | 152.757 | 0.054 | 107.107 | 1 | train |
| 0.018 | 149.924 | 160.422 | 149.194 | -0.025 | 35.542 | 1.465 | 422.247 | 0.313 | 0.281 | 148.961 | 101.932 | 149.368 | 0.059 | 125.397 | 1 | train |
| 0.021 | 154.030 | 163.531 | 153.474 | -0.035 | 9.420 | 1.512 | 434.215 | 0.295 | 0.291 | 153.301 | 104.893 | 153.618 | 0.050 | 103.040 | 1 | train |
| 0.019 | 155.486 | 168.406 | 153.203 | 0.003 | 57.778 | 1.038 | 433.891 | 0.319 | 0.323 | 152.373 | 100.534 | 153.688 | 0.025 | 43.476 | 1 | train |
| 0.019 | 157.326 | 166.156 | 156.745 | 0.009 | 29.191 | 1.627 | 443.606 | 0.287 | 0.275 | 156.547 | 107.815 | 156.800 | 0.047 | 102.102 | 0 | train |
| 0.024 | 153.356 | 160.930 | 152.809 | -0.022 | 38.064 | 1.738 | 432.450 | 0.300 | 0.255 | 152.588 | 104.515 | 152.983 | 0.116 | 159.028 | 1 | train |
| 0.017 | 165.140 | 174.906 | 164.542 | -0.017 | 32.246 | 1.533 | 465.277 | 0.302 | 0.262 | 164.358 | 111.742 | 164.695 | 0.024 | 89.775 | 0 | train |
| 0.022 | 152.971 | 161.562 | 152.542 | 0.004 | 51.606 | 1.505 | 431.183 | 0.270 | 0.276 | 152.364 | 104.604 | 152.586 | 0.048 | 107.730 | 1 | train |
| 0.019 | 152.525 | 161.219 | 152.019 | 0.006 | 45.516 | 1.639 | 430.016 | 0.288 | 0.284 | 151.804 | 103.528 | 152.053 | 0.047 | 123.401 | 1 | train |
| 0.023 | 153.534 | 161.875 | 152.943 | 0.010 | 39.277 | 1.463 | 432.516 | 0.280 | 0.279 | 152.767 | 104.182 | 153.034 | 0.050 | 74.146 | 1 | train |
| 0.017 | 152.195 | 161.000 | 151.432 | -0.010 | 17.150 | 1.656 | 428.396 | 0.293 | 0.228 | 151.165 | 102.875 | 151.697 | 0.054 | 124.876 | 1 | train |
| 0.020 | 135.130 | 142.906 | 134.628 | 0.005 | 22.527 | 1.469 | 380.911 | 0.284 | 0.277 | 134.450 | 91.581 | 134.714 | 0.087 | 170.243 | 2 | train |
| 0.020 | 159.600 | 174.969 | 156.815 | 0.001 | 39.215 | 0.928 | 444.201 | 0.337 | 0.331 | 155.899 | 104.362 | 157.439 | 0.026 | 49.041 | 1 | train |
| 0.016 | 151.502 | 161.906 | 150.670 | -0.025 | 13.337 | 1.391 | 426.373 | 0.295 | 0.309 | 150.456 | 101.797 | 150.842 | 0.030 | 87.981 | 1 | train |
| 0.018 | 153.898 | 164.094 | 153.084 | 0.005 | 32.179 | 1.423 | 433.111 | 0.302 | 0.280 | 152.844 | 104.586 | 153.284 | 0.030 | 98.226 | 1 | train |
| 0.014 | 151.945 | 161.594 | 150.944 | 0.001 | 27.486 | 1.407 | 427.047 | 0.317 | 0.270 | 150.617 | 102.656 | 151.219 | 0.065 | 155.781 | 1 | train |
| 0.019 | 154.593 | 169.750 | 151.489 | 0.012 | 42.072 | 0.843 | 428.720 | 0.331 | 0.337 | 150.427 | 99.863 | 151.938 | 0.033 | 48.409 | 1 | train |
| 0.017 | 145.276 | 154.906 | 144.624 | 0.016 | 16.186 | 1.413 | 408.833 | 0.286 | 0.297 | 144.387 | 98.255 | 144.640 | 0.072 | 162.449 | 1 | train |
| 0.018 | 154.697 | 164.609 | 153.813 | -0.002 | 18.095 | 1.415 | 435.028 | 0.332 | 0.276 | 153.534 | 104.047 | 153.981 | 0.030 | 66.169 | 0 | train |
| 0.018 | 160.691 | 169.812 | 160.180 | 0.000 | 19.692 | 1.474 | 452.887 | 0.267 | 0.271 | 160.043 | 109.967 | 160.252 | 0.047 | 97.071 | 0 | train |
| 0.022 | 160.334 | 167.938 | 159.770 | -0.006 | 68.592 | 1.427 | 451.905 | 0.294 | 0.276 | 159.586 | 108.982 | 159.887 | 0.108 | 138.035 | 1 | train |
| 0.015 | 132.169 | 145.750 | 129.892 | -0.017 | 15.007 | 1.129 | 367.685 | 0.317 | 0.307 | 129.070 | 85.052 | 130.424 | 0.040 | 106.141 | 2 | train |
| 0.017 | 123.652 | 135.703 | 122.458 | -0.002 | 13.217 | 1.420 | 346.232 | 0.297 | 0.283 | 122.106 | 81.497 | 122.687 | 0.032 | 96.446 | 2 | train |
| 0.018 | 147.755 | 155.938 | 147.157 | -0.016 | 16.940 | 1.403 | 416.241 | 0.295 | 0.276 | 146.971 | 99.036 | 147.291 | 0.058 | 146.604 | 1 | train |
| 0.024 | 148.460 | 158.906 | 147.761 | 0.013 | 42.094 | 1.630 | 417.950 | 0.279 | 0.246 | 147.542 | 99.198 | 147.973 | 0.033 | 62.386 | 1 | train |
| 0.018 | 155.047 | 163.062 | 154.522 | 0.002 | 16.780 | 1.437 | 437.179 | 0.276 | 0.283 | 154.295 | 104.910 | 154.648 | 0.115 | 157.503 | 1 | train |
| 0.018 | 144.369 | 154.344 | 143.364 | 0.006 | 39.803 | 1.453 | 405.637 | 0.294 | 0.265 | 142.973 | 96.806 | 143.634 | 0.038 | 85.248 | 1 | train |
| 0.015 | 140.638 | 149.438 | 139.668 | 0.002 | 15.497 | 1.435 | 394.914 | 0.312 | 0.311 | 139.367 | 94.335 | 139.798 | 0.042 | 113.655 | 1 | train |
| 0.022 | 159.662 | 168.562 | 159.237 | 0.000 | 78.412 | 1.532 | 450.874 | 0.255 | 0.251 | 159.126 | 108.162 | 159.373 | 0.107 | 121.807 | 0 | train |
| 0.024 | 161.587 | 168.156 | 161.234 | -0.026 | 53.652 | 1.554 | 456.376 | 0.250 | 0.245 | 161.098 | 110.591 | 161.352 | 0.088 | 85.431 | 0 | train |
| 0.018 | 149.449 | 157.359 | 148.937 | -0.010 | 35.124 | 1.435 | 421.388 | 0.282 | 0.254 | 148.777 | 102.448 | 149.045 | 0.051 | 109.142 | 1 | train |
| 0.022 | 153.929 | 160.812 | 153.607 | -0.006 | 57.238 | 1.495 | 434.247 | 0.259 | 0.256 | 153.418 | 104.381 | 153.585 | 0.098 | 125.037 | 1 | train |
| 0.018 | 129.116 | 137.922 | 128.210 | 0.015 | 13.854 | 1.458 | 362.862 | 0.285 | 0.257 | 127.942 | 87.356 | 128.472 | 0.049 | 132.285 | 2 | train |
| 0.024 | 153.808 | 164.336 | 152.599 | -0.042 | 27.405 | 1.405 | 431.825 | 0.303 | 0.258 | 152.222 | 103.351 | 153.016 | 0.031 | 66.189 | 1 | train |
| 0.013 | 135.944 | 147.781 | 133.822 | 0.006 | 25.447 | 1.437 | 379.259 | 0.330 | 0.260 | 133.174 | 88.502 | 134.334 | 0.034 | 97.610 | 2 | train |
| 0.016 | 149.456 | 159.281 | 148.638 | -0.017 | 56.575 | 1.579 | 420.857 | 0.311 | 0.279 | 148.409 | 101.321 | 148.881 | 0.061 | 156.844 | 1 | train |
| 0.018 | 160.994 | 170.858 | 160.222 | -0.022 | 50.072 | 1.462 | 453.377 | 0.303 | 0.288 | 159.946 | 110.104 | 160.426 | 0.047 | 119.515 | 0 | train |
| 0.019 | 152.031 | 160.531 | 151.324 | 0.029 | 41.282 | 1.425 | 427.907 | 0.301 | 0.292 | 150.984 | 102.695 | 151.410 | 0.055 | 108.920 | 1 | train |
| 0.018 | 120.979 | 132.594 | 120.017 | 0.009 | 21.184 | 1.450 | 339.306 | 0.294 | 0.283 | 119.704 | 80.956 | 120.177 | 0.075 | 123.623 | 2 | train |
| 0.016 | 155.927 | 167.328 | 154.748 | 0.019 | 74.028 | 1.468 | 437.318 | 0.313 | 0.248 | 154.377 | 104.379 | 155.100 | 0.031 | 74.653 | 1 | train |
| 0.021 | 160.786 | 169.531 | 160.257 | 0.002 | 70.767 | 1.765 | 453.470 | 0.306 | 0.246 | 160.075 | 109.653 | 160.370 | 0.047 | 110.334 | 0 | train |
| 0.022 | 163.548 | 180.863 | 160.428 | 0.061 | 55.136 | 1.463 | 455.341 | 0.359 | 0.241 | 159.332 | 105.567 | 161.396 | 0.027 | 57.032 | 0 | train |
| 0.013 | 152.334 | 164.062 | 151.258 | 0.017 | 37.937 | 1.439 | 427.963 | 0.322 | 0.259 | 150.863 | 102.577 | 151.543 | 0.074 | 173.080 | 1 | train |
| 0.017 | 162.253 | 179.031 | 158.700 | 0.080 | 55.029 | 0.693 | 448.889 | 0.341 | 0.342 | 157.208 | 102.611 | 159.182 | 0.035 | 63.099 | 0 | train |
| 0.020 | 150.948 | 166.875 | 147.314 | 0.039 | 18.854 | 0.760 | 416.640 | 0.342 | 0.328 | 145.963 | 94.864 | 147.978 | 0.045 | 73.597 | 1 | train |
| 0.020 | 154.351 | 164.438 | 153.359 | -0.021 | 64.975 | 1.423 | 434.097 | 0.289 | 0.262 | 153.012 | 104.271 | 153.647 | 0.030 | 79.155 | 1 | train |
| 0.023 | 158.447 | 165.969 | 158.105 | 0.020 | 76.276 | 1.480 | 447.024 | 0.264 | 0.276 | 157.962 | 108.365 | 158.104 | 0.107 | 126.610 | 0 | train |
| 0.020 | 160.356 | 167.938 | 160.010 | -0.014 | 23.712 | 1.451 | 452.214 | 0.279 | 0.293 | 159.894 | 108.798 | 159.975 | 0.105 | 110.938 | 0 | train |
| 0.017 | 114.695 | 129.312 | 113.992 | -0.018 | 22.077 | 1.528 | 322.862 | 0.280 | 0.270 | 113.758 | 76.371 | 114.177 | 0.071 | 166.001 | 2 | train |
| 0.020 | 158.365 | 166.234 | 158.006 | -0.017 | 37.432 | 1.616 | 446.560 | 0.289 | 0.254 | 157.771 | 108.696 | 158.019 | 0.046 | 87.492 | 0 | train |
| 0.022 | 142.098 | 149.766 | 141.668 | 0.007 | 35.860 | 1.456 | 400.525 | 0.274 | 0.247 | 141.538 | 96.556 | 141.756 | 0.071 | 134.142 | 1 | train |
| 0.018 | 157.801 | 172.969 | 154.864 | 0.029 | 45.733 | 0.900 | 438.043 | 0.329 | 0.310 | 153.749 | 101.865 | 155.579 | 0.031 | 76.063 | 1 | train |
| 0.021 | 162.383 | 170.625 | 161.760 | 0.001 | 36.370 | 1.639 | 457.473 | 0.313 | 0.271 | 161.593 | 111.705 | 161.857 | 0.106 | 125.832 | 0 | train |
| 0.021 | 149.689 | 159.781 | 149.021 | -0.017 | 25.726 | 1.433 | 421.664 | 0.281 | 0.284 | 148.771 | 102.194 | 149.117 | 0.061 | 101.826 | 1 | train |
| 0.021 | 157.725 | 166.000 | 157.208 | 0.016 | 26.793 | 1.524 | 444.752 | 0.281 | 0.236 | 156.956 | 108.691 | 157.368 | 0.115 | 109.613 | 0 | train |
| 0.018 | 153.646 | 161.797 | 152.996 | -0.016 | 33.553 | 1.466 | 432.727 | 0.304 | 0.258 | 152.788 | 103.615 | 153.154 | 0.047 | 146.748 | 1 | train |
| 0.020 | 147.741 | 156.500 | 147.067 | 0.003 | 44.513 | 1.445 | 415.895 | 0.292 | 0.297 | 146.851 | 100.336 | 147.135 | 0.061 | 126.181 | 1 | train |
| 0.019 | 129.809 | 137.984 | 129.185 | 0.000 | 18.208 | 1.431 | 365.492 | 0.290 | 0.269 | 128.970 | 88.104 | 129.326 | 0.104 | 204.542 | 2 | train |
| 0.022 | 155.576 | 165.375 | 154.905 | -0.003 | 47.630 | 1.480 | 438.026 | 0.289 | 0.275 | 154.641 | 105.148 | 155.017 | 0.051 | 104.216 | 1 | train |
| 0.021 | 153.785 | 162.438 | 153.277 | 0.026 | 40.923 | 1.608 | 433.424 | 0.278 | 0.253 | 153.046 | 104.769 | 153.403 | 0.052 | 101.809 | 1 | train |
| 0.016 | 138.866 | 156.750 | 134.127 | 0.004 | 19.991 | 0.641 | 380.041 | 0.325 | 0.317 | 132.352 | 83.504 | 135.135 | 0.069 | 123.357 | 2 | train |
| 0.019 | 136.414 | 149.219 | 135.266 | 0.019 | 27.301 | 1.393 | 382.586 | 0.297 | 0.264 | 134.816 | 91.204 | 135.563 | 0.026 | 76.683 | 2 | train |
| 0.023 | 157.060 | 164.312 | 156.482 | -0.025 | 41.799 | 1.409 | 442.673 | 0.302 | 0.262 | 156.322 | 108.434 | 156.626 | 0.109 | 137.194 | 1 | train |
| 0.023 | 155.258 | 162.406 | 154.885 | 0.009 | 67.604 | 1.815 | 438.104 | 0.287 | 0.241 | 154.765 | 106.177 | 154.908 | 0.101 | 155.893 | 1 | train |
| 0.025 | 167.451 | 174.891 | 167.107 | 0.000 | 69.231 | 1.539 | 472.851 | 0.264 | 0.250 | 166.933 | 115.240 | 167.200 | 0.066 | 110.047 | 0 | train |
| 0.018 | 166.424 | 182.062 | 163.263 | 0.085 | 42.089 | 0.768 | 461.035 | 0.350 | 0.322 | 161.969 | 106.027 | 163.806 | 0.031 | 58.800 | 0 | train |
| 0.018 | 162.102 | 169.562 | 161.559 | -0.006 | 42.688 | 1.484 | 457.180 | 0.299 | 0.257 | 161.437 | 111.862 | 161.719 | 0.098 | 146.740 | 0 | train |
| 0.013 | 134.889 | 144.812 | 133.636 | 0.003 | 11.581 | 1.373 | 378.164 | 0.319 | 0.277 | 133.172 | 89.515 | 133.902 | 0.055 | 158.221 | 2 | train |
| 0.016 | 153.330 | 161.672 | 152.703 | -0.021 | 34.722 | 1.515 | 432.074 | 0.291 | 0.276 | 152.466 | 103.989 | 152.860 | 0.047 | 107.910 | 0 | train |
| 0.017 | 150.483 | 158.031 | 149.816 | -0.010 | 26.057 | 1.403 | 423.776 | 0.299 | 0.286 | 149.607 | 101.704 | 149.939 | 0.050 | 121.862 | 1 | train |
| 0.021 | 155.485 | 171.219 | 152.319 | 0.032 | 44.765 | 0.821 | 430.891 | 0.314 | 0.302 | 151.030 | 99.434 | 153.212 | 0.035 | 56.102 | 0 | train |
| 0.023 | 158.898 | 165.969 | 158.573 | 0.019 | 68.151 | 1.551 | 448.535 | 0.268 | 0.225 | 158.383 | 108.477 | 158.618 | 0.093 | 114.248 | 0 | train |
| 0.018 | 157.762 | 166.156 | 157.325 | -0.014 | 29.260 | 1.550 | 444.877 | 0.293 | 0.261 | 157.193 | 107.862 | 157.411 | 0.110 | 169.172 | 0 | train |
| 0.016 | 151.625 | 161.375 | 150.900 | 0.005 | 33.905 | 1.490 | 426.991 | 0.298 | 0.266 | 150.662 | 102.094 | 151.119 | 0.054 | 139.638 | 1 | train |
| 0.021 | 151.281 | 158.438 | 150.810 | -0.009 | 10.401 | 1.441 | 426.549 | 0.269 | 0.273 | 150.733 | 102.888 | 150.905 | 0.047 | 96.913 | 1 | train |
| 0.015 | 146.792 | 156.750 | 146.113 | 0.023 | 16.377 | 1.463 | 413.108 | 0.297 | 0.292 | 145.890 | 99.087 | 146.170 | 0.069 | 145.567 | 1 | train |
| 0.020 | 155.753 | 163.688 | 155.318 | 0.014 | 40.342 | 1.453 | 439.364 | 0.279 | 0.283 | 155.130 | 106.032 | 155.371 | 0.108 | 184.115 | 1 | train |
| 0.021 | 160.108 | 167.344 | 159.661 | 0.002 | 41.895 | 1.450 | 451.678 | 0.276 | 0.256 | 159.504 | 108.533 | 159.747 | 0.046 | 95.042 | 0 | train |
| 0.019 | 150.253 | 160.531 | 149.484 | -0.006 | 55.235 | 1.593 | 422.925 | 0.282 | 0.254 | 149.193 | 101.629 | 149.727 | 0.031 | 102.323 | 1 | train |
| 0.016 | 151.070 | 159.000 | 150.448 | -0.020 | 39.682 | 1.480 | 425.569 | 0.279 | 0.249 | 150.279 | 102.877 | 150.637 | 0.050 | 120.088 | 1 | train |
| 0.022 | 140.787 | 148.500 | 140.240 | -0.006 | 40.510 | 1.451 | 396.577 | 0.268 | 0.317 | 140.063 | 95.064 | 140.294 | 0.074 | 125.473 | 1 | train |
| 0.018 | 154.940 | 164.672 | 154.424 | 0.006 | 76.967 | 1.481 | 436.610 | 0.288 | 0.314 | 154.215 | 105.150 | 154.453 | 0.047 | 137.411 | 0 | train |
| 0.021 | 157.924 | 165.734 | 157.424 | 0.021 | 50.070 | 1.417 | 445.396 | 0.277 | 0.292 | 157.220 | 106.869 | 157.489 | 0.114 | 138.580 | 0 | train |
| 0.018 | 152.352 | 160.344 | 151.932 | 0.029 | 48.235 | 1.452 | 429.534 | 0.279 | 0.268 | 151.777 | 104.239 | 151.950 | 0.048 | 115.206 | 1 | train |
| 0.021 | 157.805 | 166.844 | 157.093 | 0.015 | 48.701 | 1.472 | 444.340 | 0.279 | 0.281 | 156.716 | 107.263 | 157.269 | 0.052 | 82.749 | 0 | train |
| 0.023 | 146.376 | 154.672 | 145.577 | -0.001 | 34.298 | 1.443 | 411.771 | 0.297 | 0.270 | 145.355 | 99.532 | 145.773 | 0.067 | 117.562 | 1 | train |
| 0.019 | 142.382 | 152.211 | 141.593 | -0.006 | 44.468 | 1.505 | 400.394 | 0.297 | 0.278 | 141.364 | 96.125 | 141.761 | 0.082 | 170.482 | 1 | train |
| 0.020 | 154.129 | 163.500 | 153.486 | -0.019 | 33.120 | 1.426 | 434.401 | 0.294 | 0.292 | 153.246 | 104.931 | 153.610 | 0.048 | 102.075 | 1 | train |
| 0.021 | 151.437 | 160.219 | 150.964 | 0.015 | 29.681 | 1.575 | 426.724 | 0.281 | 0.256 | 150.748 | 102.656 | 150.975 | 0.049 | 122.863 | 1 | train |
| 0.019 | 163.898 | 182.400 | 160.193 | 0.070 | 53.004 | 0.626 | 453.082 | 0.325 | 0.298 | 158.497 | 102.957 | 161.085 | 0.037 | 55.087 | 0 | train |
| 0.016 | 161.803 | 179.062 | 158.015 | 0.076 | 38.202 | 0.641 | 447.163 | 0.326 | 0.358 | 156.352 | 101.510 | 158.202 | 0.039 | 65.830 | 1 | train |
| 0.018 | 154.405 | 169.842 | 151.464 | 0.024 | 38.440 | 0.870 | 428.901 | 0.329 | 0.349 | 150.413 | 98.174 | 151.966 | 0.033 | 60.472 | 1 | train |
| 0.018 | 147.615 | 163.719 | 143.830 | 0.025 | 46.658 | 0.728 | 406.955 | 0.320 | 0.332 | 142.401 | 92.302 | 144.558 | 0.049 | 96.182 | 1 | train |
| 0.017 | 147.139 | 155.344 | 146.574 | -0.007 | 37.833 | 1.485 | 414.485 | 0.286 | 0.315 | 146.390 | 99.663 | 146.615 | 0.058 | 167.436 | 1 | train |
| 0.021 | 151.649 | 166.125 | 148.576 | 0.017 | 52.481 | 0.899 | 420.559 | 0.342 | 0.337 | 147.554 | 97.406 | 149.195 | 0.035 | 65.494 | 1 | train |
| 0.019 | 157.647 | 171.906 | 154.984 | 0.030 | 61.696 | 0.912 | 438.232 | 0.328 | 0.334 | 154.015 | 101.980 | 155.437 | 0.029 | 61.289 | 0 | train |
| 0.021 | 153.773 | 161.688 | 153.377 | -0.042 | 58.834 | 1.544 | 433.875 | 0.260 | 0.222 | 153.244 | 106.186 | 153.492 | 0.112 | 149.827 | 1 | train |
| 0.019 | 162.101 | 178.172 | 159.056 | 0.057 | 48.010 | 0.772 | 449.931 | 0.332 | 0.311 | 157.737 | 104.195 | 159.716 | 0.032 | 54.396 | 0 | train |
| 0.022 | 162.879 | 179.719 | 159.453 | 0.079 | 46.462 | 0.715 | 450.783 | 0.336 | 0.311 | 157.876 | 102.921 | 160.151 | 0.034 | 45.063 | 1 | train |
| 0.021 | 158.869 | 166.703 | 158.394 | -0.033 | 76.405 | 1.478 | 448.047 | 0.275 | 0.289 | 158.218 | 107.828 | 158.484 | 0.098 | 132.002 | 0 | train |
| 0.021 | 146.016 | 154.812 | 145.280 | -0.012 | 25.807 | 1.434 | 411.116 | 0.305 | 0.276 | 145.019 | 99.025 | 145.448 | 0.061 | 112.805 | 1 | train |
| 0.023 | 152.149 | 160.219 | 151.617 | -0.007 | 43.938 | 1.425 | 429.016 | 0.277 | 0.266 | 151.392 | 103.554 | 151.722 | 0.049 | 112.214 | 1 | train |
| 0.019 | 159.653 | 167.219 | 159.377 | 0.007 | 40.043 | 1.470 | 450.330 | 0.271 | 0.268 | 159.302 | 108.920 | 159.338 | 0.098 | 137.279 | 1 | train |
| 0.021 | 152.026 | 161.188 | 151.500 | 0.010 | 18.879 | 1.459 | 428.445 | 0.299 | 0.288 | 151.321 | 103.218 | 151.560 | 0.133 | 154.960 | 1 | train |
| 0.019 | 158.388 | 170.375 | 157.660 | 0.017 | 17.914 | 1.459 | 446.136 | 0.304 | 0.288 | 157.353 | 107.111 | 157.792 | 0.051 | 119.927 | 1 | train |
| 0.020 | 132.047 | 141.953 | 131.386 | -0.028 | 21.632 | 1.424 | 371.512 | 0.284 | 0.262 | 131.210 | 89.297 | 131.538 | 0.048 | 115.223 | 2 | train |
| 0.019 | 134.220 | 145.212 | 133.347 | -0.016 | 34.618 | 1.402 | 377.308 | 0.308 | 0.271 | 133.102 | 90.423 | 133.524 | 0.115 | 172.460 | 2 | train |
| 0.015 | 145.691 | 157.609 | 143.910 | 0.007 | 10.273 | 1.188 | 407.018 | 0.313 | 0.294 | 143.288 | 95.783 | 144.304 | 0.027 | 76.082 | 1 | train |
| 0.019 | 151.746 | 161.656 | 150.960 | 0.003 | 36.215 | 1.421 | 426.859 | 0.302 | 0.323 | 150.730 | 102.823 | 151.042 | 0.031 | 90.834 | 1 | train |
| 0.021 | 151.223 | 161.344 | 150.495 | -0.012 | 44.097 | 1.432 | 426.091 | 0.300 | 0.281 | 150.279 | 102.416 | 150.667 | 0.056 | 116.742 | 1 | train |
| 0.022 | 159.485 | 167.250 | 158.895 | -0.011 | 76.396 | 1.419 | 449.549 | 0.307 | 0.274 | 158.704 | 109.910 | 159.031 | 0.045 | 77.696 | 0 | train |
| 0.017 | 163.385 | 177.469 | 160.868 | 0.019 | 37.427 | 0.975 | 455.576 | 0.337 | 0.316 | 159.930 | 106.605 | 161.458 | 0.024 | 58.160 | 0 | train |
| 0.020 | 156.509 | 165.375 | 155.798 | -0.001 | 12.240 | 1.437 | 440.786 | 0.294 | 0.289 | 155.675 | 106.523 | 155.943 | 0.052 | 92.163 | 1 | train |
| 0.020 | 134.237 | 143.109 | 133.262 | -0.034 | 10.735 | 1.695 | 376.974 | 0.298 | 0.256 | 133.005 | 89.943 | 133.581 | 0.027 | 108.301 | 2 | train |
| 0.019 | 127.673 | 138.688 | 126.942 | 0.016 | 22.608 | 1.450 | 358.994 | 0.304 | 0.260 | 126.696 | 85.705 | 127.081 | 0.049 | 108.040 | 2 | train |
| 0.018 | 153.823 | 163.094 | 153.202 | 0.019 | 33.494 | 1.470 | 433.282 | 0.303 | 0.284 | 152.935 | 104.313 | 153.300 | 0.052 | 143.620 | 1 | train |
| 0.020 | 159.945 | 176.497 | 156.356 | 0.051 | 58.765 | 0.971 | 442.916 | 0.341 | 0.334 | 155.073 | 102.361 | 156.565 | 0.034 | 74.021 | 0 | train |
| 0.020 | 161.054 | 170.000 | 160.714 | 0.004 | 42.089 | 1.465 | 454.351 | 0.277 | 0.277 | 160.605 | 110.754 | 160.710 | 0.103 | 85.944 | 0 | train |
| 0.019 | 165.199 | 174.094 | 164.655 | -0.007 | 28.943 | 1.517 | 465.573 | 0.283 | 0.301 | 164.449 | 113.079 | 164.734 | 0.042 | 92.750 | 0 | train |
| 0.020 | 158.346 | 167.312 | 157.992 | -0.004 | 83.142 | 1.499 | 446.851 | 0.263 | 0.291 | 157.866 | 107.465 | 157.963 | 0.046 | 86.052 | 0 | train |
| 0.023 | 159.503 | 167.594 | 158.789 | -0.021 | 61.886 | 1.454 | 449.665 | 0.297 | 0.280 | 158.524 | 108.594 | 159.001 | 0.045 | 89.520 | 0 | train |
| 0.022 | 164.331 | 172.734 | 163.858 | -0.010 | 86.061 | 1.551 | 463.712 | 0.289 | 0.237 | 163.683 | 111.040 | 164.014 | 0.085 | 105.356 | 0 | train |
| 0.021 | 168.968 | 181.781 | 166.708 | 0.027 | 58.474 | 1.038 | 471.792 | 0.345 | 0.319 | 165.824 | 111.602 | 167.130 | 0.020 | 38.437 | 0 | train |
| 0.019 | 149.624 | 157.656 | 148.987 | 0.010 | 29.818 | 1.524 | 421.551 | 0.313 | 0.251 | 148.737 | 101.640 | 149.180 | 0.052 | 151.904 | 1 | train |
| 0.014 | 125.938 | 134.359 | 125.081 | 0.012 | 6.726 | 1.447 | 353.768 | 0.310 | 0.261 | 124.776 | 84.533 | 125.279 | 0.053 | 149.844 | 2 | train |
| 0.017 | 151.708 | 164.688 | 150.801 | 0.000 | 17.167 | 1.468 | 426.586 | 0.307 | 0.294 | 150.515 | 101.872 | 150.995 | 0.069 | 124.645 | 0 | train |
| 0.014 | 149.335 | 159.156 | 148.297 | 0.004 | 16.335 | 1.396 | 419.428 | 0.317 | 0.297 | 147.959 | 100.519 | 148.470 | 0.034 | 118.275 | 1 | train |
| 0.022 | 157.366 | 166.797 | 156.746 | -0.027 | 27.681 | 1.485 | 443.257 | 0.293 | 0.267 | 156.521 | 106.420 | 156.882 | 0.044 | 92.444 | 1 | train |
| 0.024 | 154.809 | 163.328 | 154.033 | -0.038 | 36.775 | 1.507 | 435.882 | 0.310 | 0.267 | 153.795 | 103.872 | 154.294 | 0.048 | 96.239 | 1 | train |
| 0.021 | 154.803 | 162.016 | 154.421 | -0.005 | 45.597 | 1.539 | 436.971 | 0.257 | 0.236 | 154.291 | 104.771 | 154.538 | 0.105 | 136.929 | 1 | train |
| 0.022 | 161.669 | 170.531 | 161.400 | -0.007 | 62.111 | 1.481 | 456.300 | 0.259 | 0.277 | 161.321 | 110.706 | 161.353 | 0.092 | 147.629 | 0 | train |
| 0.018 | 150.520 | 160.078 | 149.555 | -0.007 | 21.014 | 1.370 | 422.939 | 0.297 | 0.280 | 149.179 | 100.765 | 149.695 | 0.033 | 89.389 | 1 | train |
| 0.024 | 149.884 | 159.406 | 148.937 | -0.028 | 40.536 | 1.451 | 421.655 | 0.282 | 0.285 | 148.564 | 100.576 | 149.218 | 0.062 | 103.166 | 1 | train |
| 0.018 | 151.829 | 160.516 | 151.306 | -0.020 | 45.881 | 1.431 | 427.753 | 0.279 | 0.301 | 151.192 | 103.314 | 151.344 | 0.048 | 139.743 | 1 | train |
| 0.023 | 162.843 | 171.828 | 162.267 | -0.022 | 55.620 | 1.453 | 459.122 | 0.300 | 0.287 | 162.120 | 110.397 | 162.387 | 0.101 | 118.700 | 0 | train |
| 0.020 | 159.897 | 168.531 | 159.285 | 0.035 | 32.980 | 1.460 | 450.852 | 0.294 | 0.277 | 159.013 | 108.407 | 159.448 | 0.116 | 156.893 | 0 | train |
| 0.023 | 156.231 | 164.891 | 155.567 | 0.006 | 34.593 | 1.475 | 439.868 | 0.297 | 0.281 | 155.239 | 107.051 | 155.709 | 0.050 | 75.666 | 1 | train |
| 0.022 | 149.395 | 159.328 | 148.485 | -0.023 | 45.419 | 1.384 | 420.216 | 0.302 | 0.306 | 148.214 | 100.346 | 148.661 | 0.070 | 108.268 | 1 | train |
| 0.021 | 160.011 | 167.156 | 159.655 | 0.017 | 44.048 | 1.548 | 451.439 | 0.271 | 0.239 | 159.496 | 109.258 | 159.720 | 0.100 | 87.714 | 0 | train |
| 0.017 | 161.609 | 178.688 | 157.820 | 0.076 | 31.311 | 0.817 | 447.231 | 0.316 | 0.290 | 156.365 | 101.664 | 158.707 | 0.035 | 73.026 | 0 | train |
| 0.018 | 164.440 | 172.969 | 164.060 | 0.004 | 52.129 | 1.592 | 464.245 | 0.273 | 0.258 | 163.880 | 113.249 | 164.129 | 0.039 | 96.991 | 0 | train |
| 0.017 | 160.620 | 169.766 | 159.964 | -0.006 | 33.332 | 1.474 | 452.643 | 0.305 | 0.273 | 159.700 | 108.259 | 160.121 | 0.047 | 111.505 | 0 | train |
| 0.019 | 167.100 | 186.000 | 163.164 | 0.140 | 30.270 | 0.567 | 461.120 | 0.326 | 0.335 | 161.158 | 104.151 | 163.423 | 0.039 | 60.988 | 0 | train |
| 0.024 | 156.041 | 162.781 | 155.812 | 0.003 | 87.946 | 1.474 | 440.399 | 0.272 | 0.276 | 155.771 | 106.380 | 155.749 | 0.099 | 132.835 | 0 | train |
| 0.018 | 123.898 | 132.750 | 123.043 | -0.001 | 28.088 | 1.426 | 348.012 | 0.293 | 0.302 | 122.795 | 83.610 | 123.133 | 0.059 | 145.074 | 2 | train |
| 0.019 | 143.707 | 154.734 | 142.425 | -0.030 | 19.121 | 1.438 | 403.014 | 0.311 | 0.280 | 141.985 | 95.685 | 142.857 | 0.043 | 96.638 | 1 | train |
| 0.025 | 162.149 | 175.000 | 159.900 | 0.007 | 25.989 | 1.082 | 452.422 | 0.336 | 0.320 | 159.185 | 105.307 | 160.493 | 0.023 | 46.739 | 1 | train |
| 0.020 | 125.642 | 140.109 | 125.072 | 0.014 | 23.078 | 1.520 | 353.695 | 0.269 | 0.262 | 124.860 | 84.075 | 125.219 | 0.058 | 115.645 | 2 | train |
| 0.021 | 159.110 | 167.281 | 158.552 | -0.009 | 46.988 | 1.529 | 448.509 | 0.296 | 0.245 | 158.363 | 109.084 | 158.684 | 0.049 | 98.675 | 0 | train |
| 0.018 | 156.413 | 164.844 | 155.713 | -0.024 | 43.543 | 1.440 | 440.447 | 0.311 | 0.274 | 155.535 | 106.397 | 155.820 | 0.046 | 92.783 | 0 | train |
| 0.018 | 146.936 | 155.344 | 146.349 | 0.011 | 29.876 | 1.481 | 413.726 | 0.294 | 0.292 | 146.108 | 99.871 | 146.400 | 0.060 | 137.168 | 1 | train |
| 0.020 | 149.809 | 157.766 | 149.276 | 0.008 | 58.288 | 1.561 | 422.368 | 0.266 | 0.241 | 149.037 | 101.144 | 149.417 | 0.052 | 98.267 | 1 | train |
| 0.019 | 134.176 | 144.906 | 133.565 | 0.014 | 29.474 | 1.449 | 377.639 | 0.279 | 0.247 | 133.338 | 90.696 | 133.676 | 0.099 | 182.983 | 2 | train |
| 0.019 | 155.355 | 165.609 | 154.675 | -0.012 | 33.762 | 1.477 | 437.797 | 0.309 | 0.274 | 154.473 | 105.023 | 154.847 | 0.050 | 132.172 | 1 | train |
| 0.017 | 141.334 | 150.000 | 140.540 | 0.002 | 10.885 | 1.449 | 397.543 | 0.304 | 0.301 | 140.185 | 95.818 | 140.654 | 0.092 | 161.437 | 1 | train |
| 0.023 | 156.105 | 164.750 | 155.713 | -0.003 | 62.635 | 1.444 | 440.690 | 0.273 | 0.278 | 155.563 | 106.540 | 155.788 | 0.103 | 156.852 | 1 | train |
| 0.021 | 156.198 | 164.281 | 155.599 | -0.015 | 40.889 | 1.420 | 440.582 | 0.281 | 0.308 | 155.310 | 105.696 | 155.722 | 0.046 | 103.558 | 1 | train |
| 0.021 | 148.718 | 156.125 | 148.201 | 0.000 | 39.082 | 1.413 | 419.142 | 0.276 | 0.302 | 148.020 | 102.293 | 148.273 | 0.051 | 115.475 | 1 | train |
| 0.016 | 133.425 | 143.219 | 132.713 | -0.006 | 17.608 | 1.392 | 375.493 | 0.296 | 0.318 | 132.457 | 90.179 | 132.798 | 0.043 | 125.610 | 2 | train |
| 0.018 | 152.836 | 163.641 | 152.119 | -0.009 | 31.845 | 1.468 | 430.579 | 0.297 | 0.276 | 151.762 | 103.535 | 152.294 | 0.055 | 108.729 | 1 | train |
| 0.023 | 152.869 | 161.766 | 152.507 | -0.011 | 35.222 | 1.490 | 430.858 | 0.273 | 0.277 | 152.387 | 104.039 | 152.530 | 0.104 | 145.367 | 1 | train |
| 0.025 | 165.286 | 172.531 | 164.917 | 0.001 | 38.006 | 1.471 | 466.659 | 0.282 | 0.254 | 164.788 | 111.729 | 164.987 | 0.076 | 74.719 | 0 | train |
| 0.018 | 149.955 | 158.938 | 149.327 | 0.004 | 16.400 | 1.477 | 422.133 | 0.286 | 0.313 | 149.067 | 100.252 | 149.396 | 0.059 | 157.641 | 1 | train |
| 0.019 | 154.549 | 166.156 | 153.876 | 0.001 | 32.242 | 1.501 | 435.191 | 0.306 | 0.288 | 153.694 | 105.419 | 154.013 | 0.052 | 143.616 | 1 | train |
| 0.018 | 130.553 | 140.594 | 129.847 | -0.014 | 8.391 | 1.469 | 367.061 | 0.279 | 0.305 | 129.629 | 88.551 | 129.950 | 0.047 | 119.889 | 2 | train |
| 0.020 | 154.086 | 162.656 | 153.658 | 0.013 | 26.020 | 1.501 | 434.450 | 0.263 | 0.246 | 153.456 | 105.215 | 153.728 | 0.048 | 97.055 | 1 | train |
| 0.019 | 142.092 | 153.438 | 140.673 | -0.001 | 28.539 | 1.322 | 398.053 | 0.311 | 0.325 | 140.173 | 94.608 | 140.921 | 0.049 | 97.972 | 1 | train |
| 0.020 | 155.298 | 163.616 | 154.748 | -0.020 | 19.464 | 1.476 | 437.810 | 0.299 | 0.297 | 154.580 | 106.865 | 154.858 | 0.048 | 93.193 | 1 | train |
| 0.018 | 157.367 | 165.125 | 156.940 | -0.006 | 24.916 | 1.368 | 443.811 | 0.274 | 0.308 | 156.798 | 107.677 | 156.962 | 0.046 | 70.283 | 0 | train |
| 0.021 | 161.316 | 175.500 | 158.981 | 0.011 | 65.591 | 0.994 | 449.823 | 0.322 | 0.299 | 158.137 | 105.916 | 159.624 | 0.025 | 39.371 | 0 | train |
| 0.020 | 111.124 | 122.925 | 110.014 | -0.011 | 42.369 | 1.477 | 310.980 | 0.306 | 0.285 | 109.670 | 74.132 | 110.229 | 0.044 | 82.917 | 3 | train |
| 0.017 | 118.125 | 131.016 | 114.645 | -0.022 | 19.046 | 0.961 | 324.566 | 0.345 | 0.313 | 113.522 | 72.791 | 115.420 | 0.080 | 83.165 | 3 | train |
| 0.017 | 118.271 | 132.906 | 114.360 | -0.006 | 25.162 | 0.876 | 323.828 | 0.329 | 0.349 | 113.060 | 71.987 | 114.870 | 0.091 | 104.337 | 3 | train |
| 0.015 | 109.168 | 123.250 | 104.754 | -0.028 | 22.870 | 0.844 | 296.824 | 0.321 | 0.349 | 103.232 | 64.555 | 105.312 | 0.115 | 118.739 | 3 | train |
| 0.018 | 133.997 | 147.766 | 131.366 | -0.009 | 16.369 | 1.073 | 371.950 | 0.339 | 0.312 | 130.446 | 86.262 | 131.957 | 0.044 | 83.959 | 2 | train |
| 0.014 | 100.034 | 113.609 | 95.327 | -0.043 | 23.596 | 0.841 | 270.096 | 0.322 | 0.345 | 93.613 | 56.967 | 95.826 | 0.128 | 158.335 | 3 | train |
| 0.017 | 109.940 | 125.603 | 105.925 | -0.014 | 29.332 | 0.881 | 300.011 | 0.327 | 0.312 | 104.468 | 65.294 | 106.915 | 0.102 | 107.125 | 3 | train |
| 0.019 | 105.132 | 117.625 | 101.348 | -0.009 | 19.213 | 0.972 | 287.070 | 0.323 | 0.331 | 99.992 | 63.360 | 102.022 | 0.118 | 103.231 | 3 | train |
| 0.016 | 104.534 | 117.484 | 102.311 | -0.026 | 19.984 | 1.325 | 289.760 | 0.317 | 0.336 | 101.592 | 66.415 | 102.646 | 0.082 | 120.034 | 3 | train |
| 0.020 | 119.100 | 130.438 | 117.151 | 0.001 | 33.617 | 1.374 | 331.747 | 0.318 | 0.332 | 116.468 | 78.092 | 117.523 | 0.047 | 93.299 | 2 | train |
| 0.016 | 116.772 | 135.766 | 113.687 | 0.004 | 17.982 | 1.560 | 322.972 | 0.354 | 0.291 | 112.812 | 72.523 | 114.428 | 0.074 | 180.253 | 3 | train |
| 0.017 | 113.825 | 131.656 | 110.058 | -0.036 | 20.729 | 1.148 | 312.472 | 0.334 | 0.273 | 109.004 | 69.733 | 111.239 | 0.088 | 90.489 | 3 | train |
| 0.019 | 112.354 | 126.188 | 108.462 | -0.011 | 29.808 | 0.890 | 306.984 | 0.313 | 0.306 | 107.037 | 67.657 | 109.426 | 0.099 | 112.214 | 3 | train |
| 0.016 | 104.668 | 120.602 | 97.855 | -0.131 | 32.232 | 0.614 | 278.101 | 0.322 | 0.297 | 95.456 | 54.398 | 99.737 | 0.098 | 166.919 | 3 | train |
| 0.016 | 125.857 | 142.375 | 120.677 | -0.023 | 22.574 | 0.688 | 341.941 | 0.340 | 0.310 | 118.830 | 74.146 | 121.991 | 0.091 | 98.436 | 3 | train |
| 0.015 | 111.673 | 126.250 | 105.948 | -0.074 | 34.872 | 0.664 | 300.424 | 0.323 | 0.342 | 103.945 | 63.245 | 106.590 | 0.103 | 132.574 | 3 | train |
| 0.014 | 98.628 | 111.703 | 92.973 | -0.081 | 28.387 | 0.923 | 264.271 | 0.331 | 0.305 | 91.272 | 54.079 | 94.139 | 0.120 | 237.488 | 3 | train |
| 0.015 | 112.874 | 129.000 | 107.278 | -0.077 | 26.843 | 0.702 | 304.471 | 0.327 | 0.357 | 105.423 | 63.898 | 107.892 | 0.100 | 119.836 | 3 | train |
| 0.016 | 111.361 | 122.734 | 108.031 | -0.038 | 22.856 | 1.079 | 306.223 | 0.333 | 0.290 | 106.961 | 68.470 | 109.091 | 0.090 | 116.107 | 3 | train |
| 0.015 | 106.826 | 118.719 | 104.311 | -0.012 | 26.428 | 1.292 | 295.382 | 0.329 | 0.291 | 103.566 | 67.446 | 105.008 | 0.079 | 142.225 | 3 | train |
| 0.018 | 121.950 | 137.250 | 118.008 | -0.011 | 21.825 | 0.836 | 334.187 | 0.320 | 0.290 | 116.614 | 74.328 | 119.117 | 0.084 | 85.289 | 2 | train |
| 0.016 | 106.637 | 121.984 | 100.169 | -0.093 | 32.490 | 0.766 | 285.257 | 0.341 | 0.335 | 98.229 | 58.003 | 101.160 | 0.106 | 175.234 | 3 | train |
| 0.020 | 128.025 | 141.688 | 125.582 | -0.010 | 31.989 | 1.145 | 355.787 | 0.323 | 0.343 | 124.686 | 82.524 | 125.958 | 0.047 | 87.128 | 2 | train |
| 0.015 | 112.207 | 126.844 | 105.794 | -0.084 | 22.430 | 0.624 | 300.211 | 0.334 | 0.286 | 103.615 | 61.461 | 107.379 | 0.105 | 124.063 | 3 | train |
| 0.016 | 121.913 | 134.312 | 119.652 | 0.003 | 12.732 | 1.744 | 339.293 | 0.358 | 0.262 | 119.043 | 78.870 | 120.051 | 0.046 | 142.542 | 2 | train |
| 0.018 | 117.468 | 130.844 | 114.075 | -0.030 | 23.375 | 1.142 | 323.486 | 0.334 | 0.304 | 113.057 | 72.562 | 114.776 | 0.077 | 93.174 | 3 | train |
| 0.016 | 100.740 | 113.641 | 97.417 | -0.006 | 32.720 | 1.050 | 275.571 | 0.319 | 0.343 | 96.263 | 60.783 | 97.726 | 0.125 | 151.495 | 3 | train |
| 0.019 | 126.799 | 139.547 | 123.602 | 0.021 | 19.223 | 1.153 | 350.149 | 0.351 | 0.300 | 122.594 | 80.488 | 124.368 | 0.060 | 99.117 | 2 | train |
| 0.018 | 115.497 | 133.688 | 113.540 | 0.013 | 24.421 | 1.635 | 322.261 | 0.340 | 0.295 | 112.961 | 74.883 | 113.890 | 0.056 | 111.386 | 3 | train |
| 0.020 | 123.004 | 133.828 | 121.397 | -0.009 | 26.748 | 1.499 | 343.620 | 0.320 | 0.281 | 120.953 | 81.202 | 121.824 | 0.037 | 106.881 | 2 | train |
| 0.019 | 109.698 | 121.797 | 107.329 | -0.012 | 21.235 | 1.493 | 304.317 | 0.329 | 0.313 | 106.566 | 70.300 | 107.898 | 0.067 | 152.758 | 3 | train |
| 0.014 | 113.337 | 124.625 | 111.048 | -0.005 | 20.173 | 1.538 | 315.011 | 0.334 | 0.295 | 110.429 | 72.454 | 111.419 | 0.058 | 112.654 | 3 | train |
| 0.019 | 108.254 | 120.844 | 104.135 | -0.041 | 30.073 | 0.920 | 294.991 | 0.332 | 0.320 | 102.806 | 64.461 | 104.863 | 0.111 | 119.843 | 3 | train |
| 0.016 | 121.328 | 134.438 | 117.235 | -0.039 | 17.458 | 0.887 | 331.958 | 0.343 | 0.326 | 115.943 | 74.311 | 118.272 | 0.083 | 101.847 | 2 | train |
| 0.017 | 116.532 | 127.250 | 114.633 | 0.016 | 16.346 | 1.312 | 324.257 | 0.334 | 0.308 | 113.969 | 75.518 | 115.017 | 0.055 | 130.833 | 3 | train |
| 0.017 | 112.849 | 126.531 | 108.302 | -0.016 | 28.154 | 0.868 | 306.903 | 0.349 | 0.310 | 106.753 | 66.826 | 109.357 | 0.104 | 115.701 | 3 | train |
| 0.022 | 109.199 | 122.562 | 105.941 | 0.002 | 33.563 | 1.085 | 300.056 | 0.333 | 0.324 | 104.824 | 67.174 | 106.553 | 0.098 | 82.707 | 3 | train |
| 0.017 | 113.775 | 126.781 | 110.223 | -0.020 | 19.775 | 0.966 | 311.543 | 0.335 | 0.324 | 109.190 | 69.191 | 110.830 | 0.092 | 118.947 | 2 | train |
| 0.016 | 121.643 | 136.406 | 116.904 | -0.026 | 15.590 | 0.732 | 331.211 | 0.322 | 0.293 | 115.198 | 71.966 | 118.233 | 0.095 | 101.256 | 3 | train |
| 0.018 | 125.877 | 140.125 | 121.475 | -0.024 | 17.274 | 0.981 | 344.961 | 0.330 | 0.331 | 120.139 | 76.707 | 122.470 | 0.080 | 121.193 | 2 | train |
| 0.016 | 132.099 | 146.389 | 127.766 | -0.020 | 26.328 | 0.795 | 361.671 | 0.348 | 0.315 | 126.272 | 79.885 | 128.909 | 0.075 | 114.216 | 2 | train |
| 0.018 | 106.061 | 121.000 | 100.845 | -0.070 | 29.799 | 0.964 | 286.836 | 0.334 | 0.324 | 99.282 | 60.400 | 101.912 | 0.117 | 142.824 | 3 | train |
| 0.016 | 116.316 | 127.969 | 114.628 | 0.003 | 15.058 | 1.402 | 324.358 | 0.316 | 0.249 | 114.062 | 75.430 | 115.256 | 0.047 | 74.831 | 3 | train |
| 0.020 | 119.445 | 131.119 | 116.931 | -0.014 | 34.726 | 1.258 | 331.441 | 0.330 | 0.341 | 116.181 | 76.623 | 117.437 | 0.054 | 87.439 | 2 | train |
| 0.021 | 126.055 | 135.391 | 125.067 | -0.002 | 29.236 | 1.397 | 353.649 | 0.306 | 0.331 | 124.778 | 85.480 | 125.157 | 0.059 | 92.156 | 2 | train |
| 0.017 | 119.982 | 131.272 | 117.509 | -0.012 | 24.096 | 1.175 | 332.684 | 0.309 | 0.298 | 116.688 | 76.318 | 118.151 | 0.053 | 98.177 | 2 | train |
| 0.015 | 118.050 | 130.906 | 116.126 | 0.003 | 26.850 | 1.561 | 328.933 | 0.326 | 0.292 | 115.642 | 76.907 | 116.445 | 0.049 | 126.534 | 3 | train |
| 0.014 | 122.530 | 134.375 | 120.526 | 0.002 | 13.295 | 1.241 | 340.905 | 0.317 | 0.328 | 119.937 | 80.168 | 120.788 | 0.046 | 117.296 | 2 | train |
| 0.017 | 132.795 | 146.420 | 129.190 | 0.003 | 24.005 | 0.924 | 365.754 | 0.343 | 0.298 | 128.040 | 83.799 | 130.227 | 0.059 | 73.955 | 2 | train |
| 0.021 | 142.353 | 157.672 | 138.739 | -0.009 | 27.921 | 0.858 | 392.889 | 0.352 | 0.320 | 137.491 | 90.575 | 139.609 | 0.049 | 71.527 | 2 | train |
| 0.017 | 105.700 | 121.625 | 99.721 | -0.079 | 27.584 | 0.733 | 282.890 | 0.338 | 0.318 | 97.877 | 58.016 | 101.197 | 0.112 | 136.352 | 3 | train |
| 0.019 | 126.252 | 141.578 | 122.615 | -0.013 | 30.438 | 0.873 | 347.223 | 0.308 | 0.341 | 121.296 | 78.430 | 123.395 | 0.073 | 90.189 | 2 | train |
| 0.018 | 113.886 | 128.156 | 109.844 | -0.016 | 22.323 | 0.899 | 310.710 | 0.334 | 0.311 | 108.553 | 68.522 | 110.833 | 0.098 | 121.315 | 3 | train |
| 0.018 | 109.188 | 123.083 | 104.787 | -0.046 | 28.422 | 0.869 | 296.908 | 0.335 | 0.337 | 103.325 | 65.199 | 105.404 | 0.114 | 90.961 | 3 | train |
| 0.015 | 115.504 | 128.594 | 111.248 | -0.028 | 16.502 | 0.853 | 315.111 | 0.319 | 0.295 | 109.830 | 69.137 | 112.297 | 0.098 | 119.399 | 3 | train |
| 0.019 | 131.906 | 143.125 | 130.463 | -0.008 | 29.058 | 1.613 | 369.552 | 0.343 | 0.276 | 129.987 | 87.443 | 130.858 | 0.058 | 147.999 | 2 | train |
| 0.019 | 122.029 | 137.036 | 120.609 | -0.004 | 17.696 | 1.541 | 341.618 | 0.346 | 0.306 | 120.207 | 80.725 | 120.941 | 0.035 | 94.106 | 2 | train |
| 0.018 | 119.159 | 132.531 | 116.038 | 0.000 | 18.535 | 1.043 | 328.707 | 0.324 | 0.313 | 114.912 | 74.947 | 116.727 | 0.070 | 91.164 | 2 | train |
| 0.018 | 112.951 | 125.531 | 109.805 | -0.014 | 22.471 | 1.071 | 310.839 | 0.317 | 0.309 | 108.772 | 70.623 | 110.464 | 0.084 | 109.473 | 3 | train |
| 0.016 | 113.918 | 128.031 | 110.996 | -0.003 | 16.061 | 1.827 | 315.329 | 0.370 | 0.270 | 110.259 | 72.006 | 111.682 | 0.072 | 175.415 | 3 | train |
| 0.019 | 112.356 | 127.281 | 107.172 | -0.054 | 27.016 | 0.740 | 303.597 | 0.318 | 0.291 | 105.385 | 64.735 | 108.776 | 0.101 | 112.606 | 3 | train |
| 0.020 | 119.283 | 135.172 | 114.622 | -0.018 | 28.216 | 0.721 | 324.431 | 0.325 | 0.308 | 112.779 | 69.900 | 115.740 | 0.097 | 85.772 | 3 | train |
| 0.016 | 129.544 | 146.406 | 124.069 | -0.025 | 14.654 | 0.886 | 353.441 | 0.349 | 0.305 | 122.482 | 76.640 | 125.240 | 0.079 | 165.160 | 2 | train |
| 0.018 | 119.114 | 134.094 | 114.923 | -0.020 | 33.509 | 0.902 | 325.654 | 0.356 | 0.282 | 113.550 | 71.436 | 116.295 | 0.088 | 88.402 | 3 | train |
| 0.017 | 127.870 | 141.594 | 123.194 | -0.012 | 20.839 | 0.745 | 348.662 | 0.341 | 0.316 | 121.562 | 76.393 | 124.379 | 0.081 | 98.514 | 2 | train |
| 0.019 | 121.715 | 138.200 | 118.367 | -0.013 | 24.090 | 0.972 | 334.856 | 0.332 | 0.323 | 117.219 | 75.495 | 118.904 | 0.077 | 90.135 | 2 | train |
| 0.015 | 104.195 | 120.359 | 99.615 | -0.072 | 19.073 | 0.874 | 282.369 | 0.323 | 0.327 | 98.110 | 59.535 | 100.422 | 0.119 | 136.845 | 3 | train |
| 0.018 | 112.694 | 126.281 | 108.417 | -0.035 | 29.268 | 0.838 | 307.048 | 0.317 | 0.345 | 106.943 | 67.562 | 109.090 | 0.107 | 97.411 | 3 | train |
| 0.016 | 109.697 | 124.125 | 104.490 | -0.071 | 23.207 | 0.747 | 296.312 | 0.324 | 0.287 | 102.582 | 62.987 | 106.110 | 0.104 | 138.101 | 3 | train |
| 0.019 | 132.732 | 143.969 | 130.896 | -0.005 | 21.727 | 1.407 | 370.507 | 0.336 | 0.302 | 130.265 | 87.210 | 131.297 | 0.031 | 83.730 | 2 | train |
| 0.016 | 123.951 | 134.297 | 122.008 | 0.001 | 13.518 | 1.277 | 345.322 | 0.320 | 0.318 | 121.360 | 81.086 | 122.344 | 0.042 | 110.061 | 2 | train |
| 0.018 | 131.177 | 145.745 | 127.901 | -0.017 | 21.881 | 1.094 | 362.434 | 0.341 | 0.318 | 126.906 | 82.056 | 128.497 | 0.055 | 84.659 | 2 | train |
| 0.017 | 100.035 | 115.281 | 93.235 | -0.139 | 25.529 | 0.725 | 265.738 | 0.324 | 0.293 | 91.074 | 52.578 | 95.332 | 0.105 | 156.845 | 3 | train |
| 0.017 | 119.326 | 134.531 | 114.706 | -0.004 | 21.735 | 0.851 | 325.620 | 0.337 | 0.349 | 113.017 | 70.798 | 115.081 | 0.093 | 106.001 | 3 | train |
| 0.016 | 95.124 | 108.969 | 89.387 | -0.111 | 22.904 | 0.771 | 253.765 | 0.335 | 0.352 | 87.385 | 51.040 | 90.099 | 0.121 | 167.551 | 3 | train |
| 0.018 | 120.564 | 134.188 | 116.489 | -0.030 | 25.512 | 1.014 | 330.442 | 0.348 | 0.305 | 115.254 | 74.092 | 117.332 | 0.085 | 104.599 | 3 | train |
| 0.019 | 128.333 | 140.281 | 125.661 | 0.020 | 32.542 | 1.130 | 356.013 | 0.338 | 0.318 | 124.786 | 82.938 | 126.274 | 0.049 | 78.150 | 2 | train |
| 0.018 | 108.198 | 122.406 | 105.296 | 0.009 | 26.307 | 2.146 | 299.983 | 0.369 | 0.261 | 104.554 | 68.313 | 105.760 | 0.081 | 231.643 | 3 | train |
| 0.016 | 118.389 | 133.922 | 112.976 | -0.034 | 15.673 | 1.097 | 322.285 | 0.354 | 0.299 | 111.606 | 70.068 | 114.033 | 0.094 | 198.361 | 3 | train |
| 0.015 | 105.549 | 122.953 | 98.818 | -0.094 | 24.634 | 1.141 | 283.994 | 0.361 | 0.291 | 97.239 | 57.879 | 99.939 | 0.101 | 279.605 | 3 | train |
| 0.017 | 121.203 | 135.406 | 116.399 | -0.006 | 26.836 | 0.751 | 329.667 | 0.338 | 0.342 | 114.700 | 71.843 | 117.070 | 0.097 | 125.325 | 3 | train |
| 0.016 | 115.055 | 128.531 | 110.080 | -0.044 | 20.192 | 0.779 | 311.833 | 0.349 | 0.323 | 108.308 | 67.412 | 111.227 | 0.106 | 134.024 | 3 | train |
| 0.016 | 121.472 | 137.545 | 116.006 | -0.041 | 13.814 | 1.004 | 330.636 | 0.350 | 0.332 | 114.627 | 71.633 | 116.588 | 0.090 | 171.575 | 2 | train |
| 0.019 | 113.518 | 129.609 | 108.935 | -0.008 | 32.618 | 1.298 | 310.185 | 0.348 | 0.270 | 107.653 | 67.543 | 110.246 | 0.096 | 151.682 | 3 | train |
| 0.017 | 138.099 | 155.194 | 134.126 | -0.015 | 13.291 | 1.349 | 381.632 | 0.376 | 0.284 | 133.142 | 86.008 | 135.206 | 0.050 | 116.098 | 2 | train |
| 0.018 | 123.017 | 138.750 | 118.184 | -0.028 | 24.787 | 0.747 | 334.737 | 0.325 | 0.319 | 116.446 | 72.552 | 119.493 | 0.094 | 93.713 | 2 | train |
| 0.020 | 113.281 | 128.039 | 110.728 | -0.016 | 30.220 | 1.258 | 313.861 | 0.333 | 0.306 | 109.959 | 71.574 | 111.380 | 0.069 | 98.993 | 3 | train |
| 0.015 | 106.386 | 119.875 | 102.453 | -0.031 | 20.180 | 0.986 | 290.405 | 0.341 | 0.298 | 101.239 | 63.470 | 103.297 | 0.117 | 135.071 | 3 | train |
| 0.019 | 120.626 | 133.703 | 117.184 | -0.011 | 22.204 | 1.118 | 332.464 | 0.350 | 0.291 | 116.143 | 75.575 | 118.109 | 0.071 | 83.617 | 3 | train |
| 0.017 | 109.238 | 124.250 | 103.467 | -0.062 | 24.410 | 0.737 | 293.280 | 0.345 | 0.319 | 101.537 | 61.665 | 105.160 | 0.110 | 130.934 | 3 | train |
| 0.016 | 113.171 | 127.306 | 107.798 | -0.055 | 27.339 | 0.756 | 305.687 | 0.349 | 0.315 | 105.948 | 65.236 | 109.111 | 0.110 | 128.580 | 3 | train |
| 0.016 | 118.349 | 131.406 | 114.172 | -0.030 | 13.023 | 0.868 | 323.085 | 0.335 | 0.342 | 112.797 | 72.157 | 114.812 | 0.099 | 124.874 | 2 | train |
| 0.016 | 106.517 | 121.172 | 102.085 | -0.034 | 27.809 | 0.899 | 289.050 | 0.324 | 0.332 | 100.564 | 62.561 | 102.861 | 0.121 | 161.014 | 3 | train |
| 0.017 | 118.675 | 133.161 | 114.331 | -0.010 | 22.852 | 0.818 | 323.666 | 0.328 | 0.309 | 112.676 | 71.251 | 115.631 | 0.091 | 114.464 | 3 | train |
| 0.015 | 103.597 | 117.969 | 98.137 | -0.085 | 23.353 | 0.793 | 278.310 | 0.342 | 0.326 | 96.341 | 58.008 | 99.202 | 0.121 | 138.150 | 3 | train |
| 0.016 | 121.922 | 135.516 | 117.976 | -0.020 | 16.366 | 1.091 | 334.789 | 0.349 | 0.293 | 116.859 | 75.536 | 119.002 | 0.078 | 121.887 | 3 | train |
| 0.014 | 126.705 | 140.906 | 122.269 | -0.005 | 14.977 | 0.725 | 346.225 | 0.331 | 0.368 | 120.653 | 77.140 | 122.509 | 0.084 | 98.855 | 2 | train |
| 0.020 | 117.540 | 130.172 | 114.223 | -0.002 | 22.298 | 1.261 | 324.257 | 0.348 | 0.313 | 113.262 | 73.558 | 114.961 | 0.074 | 88.821 | 3 | train |
| 0.022 | 134.779 | 149.391 | 131.030 | 0.003 | 31.776 | 1.298 | 372.045 | 0.372 | 0.286 | 129.938 | 84.584 | 132.023 | 0.056 | 104.502 | 2 | train |
| 0.015 | 118.831 | 135.562 | 112.311 | -0.059 | 16.783 | 0.717 | 320.224 | 0.337 | 0.331 | 110.252 | 66.977 | 113.396 | 0.097 | 189.694 | 3 | train |
| 0.019 | 129.951 | 144.172 | 125.774 | -0.012 | 17.402 | 1.121 | 357.194 | 0.360 | 0.293 | 124.644 | 79.468 | 126.910 | 0.066 | 109.006 | 2 | train |
| 0.018 | 124.233 | 137.594 | 121.283 | 0.001 | 28.193 | 1.060 | 343.282 | 0.339 | 0.315 | 120.258 | 78.942 | 121.857 | 0.060 | 103.293 | 3 | train |
| 0.016 | 132.435 | 145.000 | 130.010 | -0.008 | 11.960 | 1.474 | 368.640 | 0.340 | 0.324 | 129.434 | 86.545 | 130.377 | 0.039 | 101.126 | 2 | train |
| 0.018 | 121.111 | 132.719 | 118.817 | 0.009 | 20.324 | 1.427 | 336.753 | 0.313 | 0.299 | 118.058 | 77.743 | 119.455 | 0.051 | 115.002 | 2 | train |
| 0.018 | 142.866 | 161.016 | 137.886 | 0.022 | 32.965 | 1.346 | 393.224 | 0.399 | 0.258 | 136.727 | 87.501 | 139.114 | 0.059 | 120.526 | 2 | train |
| 0.021 | 130.772 | 142.369 | 129.042 | 0.017 | 24.130 | 1.870 | 365.797 | 0.333 | 0.254 | 128.607 | 86.628 | 129.419 | 0.032 | 90.948 | 2 | train |
| 0.017 | 135.358 | 151.281 | 130.852 | 0.007 | 17.869 | 0.863 | 371.011 | 0.355 | 0.325 | 129.409 | 82.058 | 131.672 | 0.070 | 129.477 | 2 | train |
| 0.018 | 107.253 | 126.859 | 100.538 | -0.086 | 25.181 | 1.290 | 289.829 | 0.363 | 0.305 | 99.047 | 59.012 | 102.095 | 0.087 | 153.389 | 3 | train |
| 0.016 | 124.150 | 137.312 | 120.931 | -0.020 | 22.854 | 0.973 | 342.110 | 0.323 | 0.354 | 119.888 | 77.891 | 121.489 | 0.064 | 97.961 | 2 | train |
| 0.019 | 118.426 | 131.422 | 114.808 | -0.004 | 12.286 | 0.929 | 324.931 | 0.326 | 0.341 | 113.581 | 72.892 | 115.322 | 0.085 | 84.843 | 2 | train |
| 0.016 | 92.222 | 107.938 | 87.126 | -0.097 | 32.436 | 0.849 | 247.375 | 0.317 | 0.311 | 85.291 | 49.670 | 88.803 | 0.114 | 129.139 | 3 | train |
| 0.016 | 113.176 | 128.125 | 110.092 | -0.014 | 27.776 | 1.090 | 311.669 | 0.323 | 0.333 | 108.956 | 70.090 | 110.449 | 0.083 | 100.177 | 3 | train |
| 0.015 | 136.251 | 151.750 | 131.793 | 0.005 | 17.272 | 0.767 | 373.332 | 0.349 | 0.320 | 130.195 | 82.920 | 132.769 | 0.067 | 121.181 | 2 | train |
| 0.018 | 123.018 | 136.812 | 120.033 | -0.001 | 21.447 | 1.023 | 340.097 | 0.327 | 0.326 | 119.010 | 77.424 | 120.546 | 0.062 | 78.694 | 2 | train |
| 0.015 | 131.760 | 150.969 | 127.456 | -0.014 | 26.913 | 0.719 | 361.045 | 0.317 | 0.361 | 125.872 | 79.654 | 127.710 | 0.070 | 94.274 | 2 | train |
| 0.022 | 131.302 | 144.859 | 128.498 | 0.027 | 30.243 | 1.088 | 363.669 | 0.339 | 0.288 | 127.574 | 83.538 | 129.299 | 0.047 | 62.735 | 2 | train |
| 0.023 | 130.019 | 142.719 | 127.252 | -0.013 | 22.843 | 1.029 | 360.361 | 0.329 | 0.319 | 126.364 | 83.563 | 127.788 | 0.051 | 66.031 | 2 | train |
| 0.019 | 125.232 | 134.984 | 123.454 | 0.000 | 15.309 | 1.341 | 349.458 | 0.334 | 0.277 | 122.902 | 81.906 | 123.995 | 0.038 | 106.098 | 2 | train |
| 0.018 | 126.914 | 140.250 | 123.609 | 0.014 | 18.291 | 0.986 | 349.977 | 0.329 | 0.326 | 122.430 | 79.352 | 124.074 | 0.067 | 85.994 | 2 | train |
| 0.016 | 108.411 | 122.719 | 104.458 | -0.043 | 17.601 | 0.929 | 296.091 | 0.321 | 0.335 | 103.150 | 64.581 | 105.038 | 0.102 | 98.124 | 3 | train |
| 0.018 | 128.497 | 143.094 | 124.507 | -0.002 | 12.277 | 0.787 | 352.440 | 0.319 | 0.301 | 123.086 | 78.483 | 125.577 | 0.070 | 70.955 | 2 | train |
| 0.014 | 106.887 | 120.984 | 102.300 | -0.031 | 22.373 | 1.003 | 290.672 | 0.335 | 0.299 | 100.870 | 62.389 | 103.188 | 0.114 | 170.231 | 3 | train |
| 0.018 | 124.179 | 137.000 | 121.423 | -0.015 | 12.130 | 1.075 | 343.823 | 0.330 | 0.331 | 120.518 | 78.951 | 121.987 | 0.056 | 88.813 | 2 | train |
| 0.036 | 122.419 | 136.913 | 117.916 | -0.026 | 24.923 | 0.436 | 331.389 | 0.346 | 0.320 | 115.386 | 70.585 | 117.909 | 0.098 | 118.062 | 2 | train |
| 0.021 | 126.877 | 140.641 | 123.772 | 0.019 | 31.217 | 1.220 | 350.741 | 0.336 | 0.326 | 122.731 | 79.752 | 124.495 | 0.058 | 87.185 | 2 | train |
| 0.016 | 85.694 | 97.750 | 81.113 | -0.067 | 19.348 | 0.962 | 229.970 | 0.318 | 0.330 | 79.512 | 47.264 | 81.757 | 0.140 | 179.048 | 3 | train |
| 0.018 | 138.919 | 154.578 | 135.078 | 0.003 | 20.837 | 0.758 | 382.344 | 0.324 | 0.332 | 133.564 | 86.018 | 135.701 | 0.058 | 84.173 | 2 | train |
| 0.022 | 129.611 | 143.266 | 125.852 | 0.014 | 14.560 | 0.796 | 356.133 | 0.336 | 0.302 | 124.431 | 79.971 | 126.765 | 0.070 | 64.809 | 2 | train |
| 0.016 | 111.267 | 127.156 | 106.471 | -0.042 | 21.301 | 0.754 | 301.895 | 0.316 | 0.315 | 104.666 | 64.266 | 107.507 | 0.106 | 116.420 | 3 | train |
| 0.020 | 121.308 | 134.031 | 118.556 | -0.002 | 14.537 | 1.078 | 335.456 | 0.316 | 0.297 | 117.563 | 76.785 | 119.352 | 0.061 | 79.878 | 2 | train |
| 0.018 | 117.229 | 130.406 | 114.257 | -0.012 | 27.670 | 1.080 | 323.142 | 0.319 | 0.297 | 113.240 | 73.026 | 114.976 | 0.069 | 82.439 | 2 | train |
| 0.017 | 121.243 | 133.938 | 118.039 | 0.013 | 17.282 | 1.011 | 334.083 | 0.329 | 0.345 | 116.910 | 76.235 | 118.415 | 0.072 | 107.970 | 2 | train |
| 0.019 | 127.791 | 144.219 | 123.763 | -0.005 | 28.051 | 0.807 | 350.544 | 0.327 | 0.325 | 122.120 | 77.907 | 124.762 | 0.073 | 74.839 | 2 | train |
| 0.019 | 122.566 | 136.156 | 119.335 | -0.021 | 18.595 | 0.970 | 338.095 | 0.321 | 0.336 | 118.331 | 76.417 | 119.958 | 0.066 | 92.567 | 2 | train |
| 0.014 | 117.190 | 129.594 | 114.017 | -0.007 | 14.095 | 1.058 | 322.903 | 0.334 | 0.304 | 112.972 | 73.339 | 114.701 | 0.076 | 132.944 | 3 | train |
| 0.017 | 116.853 | 126.531 | 115.449 | -0.017 | 14.879 | 1.388 | 326.827 | 0.311 | 0.307 | 115.006 | 77.335 | 115.704 | 0.043 | 91.771 | 3 | train |
| 0.019 | 125.957 | 137.250 | 123.395 | -0.006 | 17.412 | 1.099 | 349.312 | 0.329 | 0.338 | 122.456 | 80.799 | 123.734 | 0.054 | 79.908 | 2 | train |
| 0.016 | 105.779 | 119.531 | 102.522 | -0.024 | 25.988 | 1.199 | 290.427 | 0.320 | 0.256 | 101.399 | 64.602 | 103.635 | 0.096 | 145.124 | 3 | train |
| 0.018 | 115.583 | 128.047 | 113.054 | 0.007 | 14.152 | 1.756 | 320.972 | 0.339 | 0.283 | 112.374 | 73.866 | 113.431 | 0.059 | 136.835 | 3 | train |
| 0.016 | 104.904 | 117.172 | 100.755 | -0.034 | 17.656 | 0.905 | 285.428 | 0.322 | 0.273 | 99.205 | 62.306 | 102.043 | 0.113 | 90.575 | 3 | train |
| 0.021 | 113.996 | 129.156 | 110.238 | -0.040 | 24.472 | 0.904 | 312.467 | 0.317 | 0.299 | 108.901 | 69.344 | 111.241 | 0.093 | 105.067 | 3 | train |
| 0.015 | 114.744 | 126.834 | 110.967 | -0.007 | 10.782 | 0.927 | 314.078 | 0.350 | 0.325 | 109.660 | 69.776 | 111.717 | 0.095 | 115.926 | 3 | train |
| 0.016 | 111.244 | 123.859 | 108.719 | -0.005 | 16.756 | 1.537 | 308.227 | 0.338 | 0.308 | 108.028 | 70.972 | 108.989 | 0.070 | 135.199 | 3 | train |
| 0.016 | 110.654 | 124.031 | 108.092 | 0.002 | 12.259 | 1.759 | 307.008 | 0.340 | 0.265 | 107.412 | 70.189 | 108.750 | 0.068 | 145.436 | 3 | train |
| 0.019 | 112.841 | 124.344 | 111.525 | 0.020 | 29.042 | 1.417 | 315.202 | 0.302 | 0.309 | 111.080 | 74.555 | 111.824 | 0.046 | 95.806 | 3 | train |
| 0.018 | 111.027 | 124.688 | 108.130 | -0.005 | 19.407 | 1.156 | 306.403 | 0.330 | 0.318 | 107.116 | 69.829 | 108.872 | 0.083 | 113.063 | 3 | train |
| 0.017 | 100.425 | 114.750 | 96.518 | -0.032 | 27.850 | 1.013 | 273.777 | 0.329 | 0.326 | 95.152 | 59.466 | 97.307 | 0.123 | 120.787 | 3 | train |
| 0.014 | 98.040 | 111.031 | 92.860 | -0.090 | 22.482 | 0.821 | 263.593 | 0.314 | 0.283 | 91.136 | 54.585 | 94.313 | 0.127 | 148.848 | 3 | train |
| 0.018 | 116.870 | 131.984 | 112.456 | -0.031 | 25.680 | 0.951 | 319.408 | 0.333 | 0.304 | 111.029 | 70.011 | 113.321 | 0.095 | 101.546 | 3 | train |
| 0.015 | 106.820 | 118.844 | 104.383 | 0.002 | 12.516 | 1.691 | 296.568 | 0.336 | 0.271 | 103.724 | 68.387 | 104.905 | 0.069 | 164.069 | 3 | train |
| 0.020 | 118.630 | 128.281 | 117.002 | 0.000 | 19.300 | 1.363 | 331.297 | 0.322 | 0.305 | 116.510 | 78.654 | 117.338 | 0.042 | 94.690 | 3 | train |
| 0.012 | 116.928 | 130.781 | 113.012 | -0.012 | 8.049 | 1.347 | 321.471 | 0.347 | 0.257 | 111.881 | 71.931 | 114.056 | 0.082 | 192.024 | 3 | train |
| 0.020 | 108.843 | 127.383 | 104.321 | -0.056 | 24.011 | 0.877 | 295.863 | 0.332 | 0.331 | 102.849 | 62.567 | 105.372 | 0.103 | 101.942 | 3 | train |
| 0.018 | 98.823 | 110.969 | 96.927 | -0.003 | 22.984 | 1.383 | 274.369 | 0.314 | 0.324 | 96.266 | 63.348 | 97.167 | 0.079 | 176.002 | 3 | train |
| 0.014 | 121.660 | 132.875 | 119.831 | 0.001 | 26.765 | 1.564 | 339.398 | 0.332 | 0.317 | 119.286 | 79.351 | 120.038 | 0.044 | 165.394 | 2 | train |
| 0.017 | 112.345 | 124.344 | 110.074 | 0.017 | 20.987 | 1.260 | 311.308 | 0.342 | 0.300 | 109.357 | 71.721 | 110.669 | 0.068 | 103.567 | 3 | train |
| 0.018 | 111.427 | 124.828 | 107.976 | 0.008 | 16.602 | 0.976 | 305.510 | 0.327 | 0.345 | 106.710 | 68.587 | 108.460 | 0.094 | 95.056 | 3 | train |
| 0.019 | 117.886 | 129.409 | 116.662 | -0.001 | 12.561 | 1.465 | 330.139 | 0.302 | 0.296 | 116.197 | 78.050 | 116.944 | 0.038 | 114.893 | 2 | train |
| 0.018 | 116.999 | 127.031 | 115.583 | 0.005 | 12.061 | 1.411 | 327.044 | 0.297 | 0.330 | 115.047 | 77.181 | 115.876 | 0.044 | 97.999 | 2 | train |
| 0.020 | 116.106 | 125.609 | 115.235 | 0.013 | 20.139 | 1.528 | 326.062 | 0.280 | 0.246 | 114.923 | 77.819 | 115.494 | 0.037 | 100.471 | 3 | train |
| 0.016 | 120.635 | 130.188 | 119.408 | -0.006 | 12.702 | 1.521 | 337.833 | 0.307 | 0.262 | 118.998 | 80.217 | 119.718 | 0.037 | 149.000 | 2 | train |
| 0.015 | 108.684 | 121.969 | 104.537 | -0.055 | 20.496 | 0.851 | 296.549 | 0.322 | 0.305 | 103.136 | 64.992 | 105.658 | 0.104 | 119.398 | 3 | train |
| 0.029 | 160.678 | 169.511 | 160.163 | 0.019 | 66.193 | 1.545 | 452.888 | 0.285 | 0.259 | 159.921 | 109.390 | 160.248 | 0.049 | 64.578 | 0 | test |
| 0.019 | 160.164 | 168.406 | 159.689 | 0.003 | 43.565 | 1.481 | 451.681 | 0.289 | 0.229 | 159.469 | 109.622 | 159.805 | 0.044 | 71.624 | 1 | test |
| 0.022 | 156.305 | 164.628 | 155.806 | 0.001 | 53.995 | 1.588 | 440.635 | 0.280 | 0.288 | 155.719 | 106.082 | 155.886 | 0.046 | 110.422 | 0 | test |
| 0.017 | 127.347 | 137.250 | 126.268 | -0.002 | 29.751 | 1.450 | 357.165 | 0.302 | 0.316 | 125.942 | 85.400 | 126.412 | 0.029 | 119.319 | 2 | test |
| 0.020 | 167.763 | 177.469 | 167.103 | 0.004 | 49.957 | 1.478 | 472.896 | 0.289 | 0.285 | 166.837 | 115.308 | 167.230 | 0.077 | 110.174 | 0 | test |
| 0.017 | 159.629 | 169.922 | 158.821 | -0.008 | 97.488 | 1.451 | 449.894 | 0.308 | 0.267 | 158.438 | 108.693 | 159.007 | 0.019 | 79.172 | 1 | test |
| 0.017 | 130.243 | 145.156 | 129.194 | 0.008 | 27.975 | 1.502 | 365.714 | 0.321 | 0.258 | 128.788 | 86.399 | 129.503 | 0.056 | 123.617 | 2 | test |
| 0.016 | 153.144 | 166.766 | 152.480 | -0.011 | 27.941 | 1.444 | 431.545 | 0.293 | 0.311 | 152.212 | 103.772 | 152.611 | 0.054 | 98.901 | 1 | test |
| 0.017 | 161.495 | 178.594 | 158.461 | 0.056 | 45.036 | 0.796 | 448.397 | 0.329 | 0.353 | 157.229 | 103.440 | 158.735 | 0.030 | 60.610 | 1 | test |
| 0.022 | 163.070 | 170.969 | 162.604 | -0.029 | 37.521 | 1.460 | 459.905 | 0.275 | 0.273 | 162.477 | 110.373 | 162.725 | 0.096 | 100.293 | 0 | test |
| 0.018 | 161.101 | 170.328 | 160.446 | -0.007 | 65.695 | 1.460 | 453.722 | 0.295 | 0.292 | 160.225 | 111.160 | 160.517 | 0.046 | 99.428 | 0 | test |
| 0.020 | 145.839 | 160.391 | 142.194 | 0.025 | 59.290 | 0.781 | 402.172 | 0.318 | 0.326 | 140.868 | 92.407 | 142.841 | 0.049 | 50.952 | 1 | test |
| 0.020 | 160.363 | 170.250 | 159.914 | 0.001 | 59.961 | 1.476 | 452.428 | 0.278 | 0.284 | 159.757 | 108.958 | 159.967 | 0.094 | 176.563 | 0 | test |
| 0.021 | 157.098 | 165.219 | 156.645 | -0.007 | 55.998 | 1.516 | 442.939 | 0.288 | 0.279 | 156.522 | 107.125 | 156.673 | 0.046 | 101.954 | 0 | test |
| 0.018 | 157.762 | 166.422 | 157.217 | -0.028 | 35.369 | 1.460 | 444.709 | 0.300 | 0.271 | 157.031 | 107.136 | 157.313 | 0.106 | 164.214 | 0 | test |
| 0.020 | 163.116 | 171.531 | 162.413 | -0.008 | 81.023 | 1.443 | 459.755 | 0.282 | 0.294 | 162.187 | 109.925 | 162.558 | 0.044 | 86.494 | 0 | test |
| 0.017 | 156.118 | 164.156 | 155.648 | 0.002 | 43.866 | 1.596 | 440.301 | 0.284 | 0.219 | 155.423 | 106.546 | 155.784 | 0.113 | 109.051 | 1 | test |
| 0.017 | 155.527 | 165.062 | 154.715 | -0.016 | 32.085 | 1.394 | 437.833 | 0.292 | 0.279 | 154.335 | 104.763 | 154.862 | 0.027 | 82.084 | 1 | test |
| 0.021 | 156.869 | 164.375 | 156.536 | -0.018 | 63.309 | 1.464 | 442.544 | 0.260 | 0.286 | 156.443 | 107.557 | 156.486 | 0.046 | 105.399 | 1 | test |
| 0.019 | 154.311 | 164.391 | 153.502 | 0.009 | 33.629 | 1.491 | 434.132 | 0.291 | 0.262 | 153.210 | 104.162 | 153.730 | 0.030 | 73.706 | 1 | test |
| 0.019 | 156.934 | 166.656 | 156.432 | -0.006 | 63.469 | 1.456 | 442.945 | 0.282 | 0.269 | 156.155 | 107.569 | 156.551 | 0.046 | 97.271 | 1 | test |
| 0.020 | 156.642 | 165.250 | 156.131 | 0.029 | 52.924 | 1.469 | 442.221 | 0.282 | 0.284 | 155.891 | 107.425 | 156.234 | 0.049 | 108.089 | 0 | test |
| 0.016 | 144.985 | 154.547 | 144.493 | 0.001 | 46.518 | 1.430 | 408.698 | 0.291 | 0.266 | 144.296 | 98.156 | 144.567 | 0.033 | 74.097 | 1 | test |
| 0.018 | 155.632 | 166.328 | 154.768 | 0.008 | 33.639 | 1.546 | 437.837 | 0.299 | 0.295 | 154.430 | 105.012 | 154.959 | 0.031 | 97.217 | 0 | test |
| 0.022 | 154.428 | 162.156 | 154.010 | -0.007 | 39.592 | 1.473 | 435.527 | 0.265 | 0.274 | 153.818 | 105.767 | 154.065 | 0.048 | 94.330 | 1 | test |
| 0.021 | 162.951 | 176.125 | 160.695 | -0.005 | 94.898 | 1.088 | 454.941 | 0.341 | 0.324 | 159.990 | 107.704 | 161.161 | 0.040 | 63.398 | 0 | test |
| 0.022 | 166.286 | 174.266 | 165.812 | -0.020 | 66.594 | 1.491 | 469.150 | 0.292 | 0.236 | 165.623 | 113.300 | 165.933 | 0.078 | 145.897 | 0 | test |
| 0.021 | 155.312 | 162.547 | 154.838 | 0.006 | 58.683 | 1.458 | 437.992 | 0.272 | 0.245 | 154.647 | 105.708 | 154.926 | 0.112 | 127.883 | 0 | test |
| 0.018 | 159.594 | 170.125 | 159.051 | -0.004 | 36.094 | 1.461 | 449.854 | 0.287 | 0.299 | 158.869 | 108.081 | 159.121 | 0.045 | 87.564 | 0 | test |
| 0.017 | 134.928 | 144.572 | 134.227 | 0.019 | 27.728 | 1.469 | 379.478 | 0.293 | 0.282 | 133.929 | 91.830 | 134.322 | 0.045 | 116.945 | 2 | test |
| 0.017 | 158.457 | 166.981 | 157.900 | 0.012 | 37.464 | 1.454 | 446.848 | 0.285 | 0.269 | 157.627 | 108.308 | 157.990 | 0.047 | 102.967 | 0 | test |
| 0.019 | 159.615 | 169.078 | 158.857 | 0.002 | 48.011 | 1.484 | 449.456 | 0.289 | 0.276 | 158.543 | 109.046 | 159.063 | 0.048 | 86.303 | 0 | test |
| 0.017 | 147.713 | 159.969 | 147.012 | 0.016 | 35.006 | 1.416 | 415.927 | 0.292 | 0.311 | 146.823 | 99.827 | 147.115 | 0.066 | 154.268 | 1 | test |
| 0.019 | 134.993 | 143.562 | 134.419 | 0.007 | 28.456 | 1.419 | 380.424 | 0.285 | 0.269 | 134.190 | 91.443 | 134.533 | 0.092 | 180.871 | 2 | test |
| 0.023 | 153.880 | 161.859 | 153.353 | -0.009 | 58.405 | 1.447 | 433.803 | 0.272 | 0.291 | 153.229 | 104.851 | 153.470 | 0.048 | 82.242 | 0 | test |
| 0.018 | 152.461 | 162.078 | 151.587 | 0.004 | 45.244 | 1.419 | 428.645 | 0.309 | 0.316 | 151.300 | 102.751 | 151.726 | 0.059 | 158.139 | 1 | test |
| 0.022 | 156.470 | 165.562 | 155.782 | -0.024 | 63.920 | 1.550 | 440.655 | 0.296 | 0.281 | 155.542 | 105.929 | 155.942 | 0.050 | 80.763 | 0 | test |
| 0.016 | 151.399 | 161.562 | 150.554 | 0.016 | 45.173 | 1.495 | 425.841 | 0.282 | 0.283 | 150.249 | 101.896 | 150.717 | 0.030 | 107.183 | 1 | test |
| 0.017 | 154.411 | 163.594 | 153.689 | -0.022 | 35.613 | 1.547 | 434.921 | 0.304 | 0.292 | 153.469 | 104.797 | 153.826 | 0.052 | 143.571 | 0 | test |
| 0.021 | 162.421 | 170.375 | 162.103 | 0.019 | 73.331 | 1.608 | 458.353 | 0.265 | 0.271 | 161.966 | 110.232 | 162.126 | 0.085 | 131.205 | 0 | test |
| 0.018 | 163.066 | 171.438 | 162.499 | 0.013 | 16.224 | 1.548 | 459.945 | 0.295 | 0.263 | 162.201 | 110.050 | 162.631 | 0.099 | 95.066 | 0 | test |
| 0.020 | 150.749 | 158.734 | 150.160 | -0.015 | 58.096 | 1.424 | 424.680 | 0.283 | 0.304 | 149.946 | 102.460 | 150.224 | 0.018 | 72.648 | 1 | test |
| 0.017 | 156.074 | 164.453 | 155.405 | 0.000 | 36.504 | 1.382 | 439.510 | 0.297 | 0.301 | 155.201 | 106.912 | 155.527 | 0.047 | 110.368 | 1 | test |
| 0.016 | 152.417 | 161.125 | 151.804 | -0.008 | 30.015 | 1.482 | 429.301 | 0.303 | 0.279 | 151.558 | 102.360 | 151.939 | 0.019 | 62.342 | 1 | test |
| 0.020 | 165.138 | 172.375 | 164.732 | 0.021 | 64.378 | 1.525 | 465.925 | 0.285 | 0.235 | 164.520 | 112.607 | 164.846 | 0.093 | 123.519 | 0 | test |
| 0.020 | 153.997 | 163.250 | 153.423 | 0.039 | 52.925 | 1.454 | 433.291 | 0.304 | 0.300 | 153.154 | 104.639 | 153.451 | 0.050 | 135.632 | 1 | test |
| 0.018 | 157.055 | 166.781 | 156.530 | -0.004 | 47.143 | 1.454 | 443.019 | 0.286 | 0.276 | 156.247 | 107.661 | 156.639 | 0.017 | 63.456 | 1 | test |
| 0.017 | 150.185 | 160.219 | 149.341 | 0.015 | 89.973 | 1.628 | 422.624 | 0.295 | 0.243 | 148.971 | 101.206 | 149.619 | 0.068 | 134.978 | 1 | test |
| 0.021 | 156.659 | 164.578 | 156.331 | 0.013 | 55.511 | 1.541 | 442.061 | 0.268 | 0.253 | 156.177 | 107.612 | 156.353 | 0.107 | 155.067 | 0 | test |
| 0.021 | 160.812 | 170.984 | 160.197 | -0.031 | 78.792 | 1.455 | 452.821 | 0.296 | 0.291 | 160.064 | 108.449 | 160.228 | 0.046 | 100.460 | 1 | test |
| 0.020 | 161.234 | 169.906 | 160.691 | -0.027 | 65.018 | 1.452 | 454.899 | 0.284 | 0.296 | 160.523 | 110.613 | 160.835 | 0.046 | 89.505 | 0 | test |
| 0.018 | 155.994 | 163.906 | 155.651 | -0.002 | 76.184 | 1.495 | 440.101 | 0.269 | 0.277 | 155.466 | 107.010 | 155.648 | 0.047 | 126.259 | 1 | test |
| 0.020 | 161.717 | 169.594 | 161.218 | -0.008 | 68.984 | 1.493 | 456.051 | 0.275 | 0.238 | 161.037 | 111.495 | 161.358 | 0.099 | 95.549 | 0 | test |
| 0.021 | 147.659 | 156.406 | 147.088 | -0.015 | 33.676 | 1.398 | 416.140 | 0.278 | 0.287 | 146.859 | 100.111 | 147.194 | 0.057 | 132.223 | 1 | test |
| 0.022 | 164.980 | 181.219 | 162.789 | 0.033 | 69.112 | 0.992 | 460.643 | 0.333 | 0.358 | 161.954 | 108.102 | 163.041 | 0.023 | 52.882 | 0 | test |
| 0.020 | 165.263 | 179.186 | 162.699 | 0.022 | 66.815 | 0.917 | 460.409 | 0.337 | 0.348 | 161.821 | 108.195 | 162.913 | 0.023 | 52.288 | 0 | test |
| 0.020 | 165.119 | 180.814 | 162.287 | 0.064 | 63.496 | 0.906 | 459.545 | 0.335 | 0.341 | 161.261 | 106.519 | 162.622 | 0.026 | 62.999 | 0 | test |
| 0.017 | 167.401 | 181.172 | 165.102 | 0.034 | 55.219 | 0.992 | 467.092 | 0.325 | 0.330 | 164.242 | 109.847 | 165.431 | 0.022 | 51.979 | 0 | test |
| 0.022 | 152.600 | 160.984 | 152.134 | 0.002 | 45.663 | 1.568 | 430.305 | 0.269 | 0.232 | 151.913 | 104.155 | 152.247 | 0.047 | 107.150 | 1 | test |
| 0.021 | 149.853 | 157.656 | 149.243 | 0.000 | 44.940 | 1.465 | 422.093 | 0.288 | 0.267 | 149.046 | 102.541 | 149.387 | 0.049 | 101.171 | 1 | test |
| 0.016 | 147.518 | 158.844 | 146.506 | 0.006 | 27.101 | 1.408 | 414.711 | 0.321 | 0.306 | 146.141 | 99.587 | 146.714 | 0.036 | 122.286 | 1 | test |
| 0.017 | 161.263 | 169.188 | 160.894 | 0.014 | 43.513 | 1.440 | 454.513 | 0.286 | 0.290 | 160.812 | 109.849 | 160.847 | 0.048 | 95.423 | 1 | test |
| 0.026 | 155.944 | 163.641 | 155.592 | 0.014 | 55.338 | 1.502 | 439.826 | 0.274 | 0.294 | 155.444 | 105.935 | 155.609 | 0.103 | 119.573 | 0 | test |
| 0.018 | 144.541 | 153.656 | 143.902 | 0.008 | 29.428 | 1.443 | 407.233 | 0.292 | 0.261 | 143.677 | 98.118 | 144.058 | 0.069 | 165.283 | 1 | test |
| 0.019 | 163.643 | 174.375 | 163.193 | 0.018 | 62.149 | 1.540 | 461.806 | 0.285 | 0.291 | 163.070 | 110.205 | 163.240 | 0.084 | 133.770 | 0 | test |
| 0.014 | 155.478 | 163.953 | 155.124 | 0.017 | 24.839 | 1.499 | 438.372 | 0.264 | 0.282 | 154.981 | 105.760 | 155.105 | 0.046 | 117.788 | 1 | test |
| 0.018 | 121.108 | 132.172 | 119.872 | 0.012 | 17.277 | 1.421 | 339.095 | 0.293 | 0.300 | 119.348 | 80.219 | 120.101 | 0.037 | 93.328 | 2 | test |
| 0.017 | 162.990 | 179.281 | 159.620 | 0.064 | 41.356 | 0.741 | 451.675 | 0.321 | 0.301 | 158.132 | 103.398 | 160.447 | 0.031 | 59.648 | 1 | test |
| 0.020 | 164.666 | 181.031 | 161.571 | 0.051 | 30.105 | 0.801 | 457.168 | 0.346 | 0.329 | 160.368 | 104.751 | 162.036 | 0.027 | 50.742 | 0 | test |
| 0.021 | 155.231 | 163.828 | 154.773 | -0.011 | 39.172 | 1.690 | 437.893 | 0.283 | 0.246 | 154.548 | 107.868 | 154.967 | 0.042 | 106.094 | 1 | test |
| 0.014 | 162.465 | 171.042 | 161.984 | 0.013 | 67.830 | 1.487 | 457.985 | 0.273 | 0.286 | 161.813 | 110.994 | 162.035 | 0.104 | 195.199 | 0 | test |
| 0.020 | 148.347 | 157.469 | 147.915 | -0.018 | 57.000 | 1.576 | 418.781 | 0.261 | 0.248 | 147.761 | 101.809 | 148.021 | 0.051 | 110.905 | 1 | test |
| 0.023 | 146.003 | 153.141 | 145.505 | 0.016 | 50.077 | 1.507 | 411.660 | 0.268 | 0.244 | 145.334 | 99.602 | 145.639 | 0.053 | 134.338 | 1 | test |
| 0.018 | 161.411 | 168.109 | 160.921 | -0.004 | 22.669 | 1.495 | 455.308 | 0.277 | 0.266 | 160.749 | 110.460 | 161.083 | 0.088 | 130.572 | 0 | test |
| 0.015 | 126.508 | 136.719 | 125.662 | -0.001 | 13.170 | 1.458 | 355.474 | 0.290 | 0.278 | 125.348 | 85.209 | 125.843 | 0.055 | 153.851 | 2 | test |
| 0.019 | 160.054 | 168.828 | 159.445 | 0.009 | 23.668 | 1.440 | 450.985 | 0.315 | 0.283 | 159.224 | 108.487 | 159.545 | 0.047 | 70.820 | 1 | test |
| 0.016 | 165.075 | 173.438 | 164.546 | -0.006 | 57.651 | 1.599 | 465.646 | 0.287 | 0.228 | 164.374 | 112.167 | 164.695 | 0.041 | 110.678 | 0 | test |
| 0.021 | 149.375 | 159.328 | 148.683 | -0.018 | 27.436 | 1.475 | 420.818 | 0.273 | 0.258 | 148.479 | 102.210 | 148.899 | 0.058 | 118.175 | 1 | test |
| 0.018 | 154.455 | 162.812 | 153.893 | 0.001 | 39.767 | 1.512 | 435.325 | 0.284 | 0.249 | 153.650 | 105.663 | 154.055 | 0.048 | 101.386 | 1 | test |
| 0.023 | 141.660 | 149.781 | 140.974 | -0.025 | 54.972 | 1.398 | 398.512 | 0.297 | 0.308 | 140.779 | 95.233 | 141.024 | 0.081 | 113.964 | 1 | test |
| 0.020 | 159.881 | 174.062 | 157.357 | 0.010 | 62.148 | 0.985 | 445.446 | 0.345 | 0.312 | 156.451 | 104.600 | 157.811 | 0.025 | 66.755 | 0 | test |
| 0.018 | 161.221 | 177.125 | 158.080 | 0.050 | 47.676 | 0.807 | 447.524 | 0.327 | 0.346 | 156.921 | 103.442 | 158.421 | 0.033 | 54.934 | 0 | test |
| 0.021 | 160.761 | 168.562 | 160.387 | 0.018 | 45.877 | 1.478 | 453.276 | 0.287 | 0.270 | 160.239 | 109.768 | 160.379 | 0.047 | 84.738 | 0 | test |
| 0.017 | 117.361 | 127.625 | 116.771 | 0.003 | 27.097 | 1.546 | 330.243 | 0.276 | 0.272 | 116.593 | 79.121 | 116.875 | 0.067 | 156.568 | 2 | test |
| 0.031 | 151.421 | 160.625 | 150.998 | 0.010 | 30.712 | 1.613 | 426.645 | 0.265 | 0.257 | 150.991 | 102.828 | 151.054 | 0.119 | 127.000 | 0 | test |
| 0.016 | 155.059 | 164.359 | 154.461 | -0.023 | 39.025 | 1.459 | 437.177 | 0.291 | 0.277 | 154.268 | 105.364 | 154.592 | 0.050 | 164.756 | 1 | test |
| 0.021 | 154.815 | 163.297 | 154.212 | -0.014 | 39.359 | 1.501 | 436.209 | 0.294 | 0.274 | 154.046 | 105.945 | 154.341 | 0.048 | 149.738 | 1 | test |
| 0.018 | 154.259 | 162.500 | 153.619 | -0.004 | 18.768 | 1.469 | 434.471 | 0.298 | 0.290 | 153.449 | 104.617 | 153.721 | 0.050 | 137.020 | 1 | test |
| 0.020 | 160.559 | 169.828 | 160.204 | 0.003 | 42.880 | 1.566 | 453.454 | 0.273 | 0.293 | 159.985 | 109.835 | 160.262 | 0.094 | 170.556 | 0 | test |
| 0.020 | 147.392 | 157.094 | 146.695 | 0.001 | 36.765 | 1.504 | 414.686 | 0.305 | 0.292 | 146.433 | 99.841 | 146.754 | 0.032 | 106.841 | 1 | test |
| 0.021 | 163.259 | 172.125 | 162.538 | 0.003 | 76.519 | 1.526 | 460.039 | 0.309 | 0.272 | 162.246 | 110.953 | 162.756 | 0.045 | 88.416 | 0 | test |
| 0.019 | 154.251 | 162.439 | 153.586 | -0.012 | 44.176 | 1.438 | 434.492 | 0.281 | 0.282 | 153.388 | 104.707 | 153.719 | 0.051 | 123.417 | 0 | test |
| 0.018 | 150.573 | 162.219 | 150.151 | 0.005 | 20.224 | 1.434 | 424.960 | 0.267 | 0.280 | 149.970 | 103.088 | 150.245 | 0.050 | 106.671 | 1 | test |
| 0.021 | 144.620 | 152.031 | 144.180 | 0.014 | 46.374 | 1.453 | 407.595 | 0.261 | 0.284 | 144.025 | 98.373 | 144.235 | 0.058 | 112.881 | 1 | test |
| 0.019 | 153.724 | 164.391 | 152.777 | 0.013 | 29.457 | 1.421 | 432.134 | 0.303 | 0.288 | 152.475 | 103.481 | 152.982 | 0.032 | 99.914 | 1 | test |
| 0.018 | 155.000 | 165.141 | 154.485 | -0.017 | 39.048 | 1.527 | 437.323 | 0.275 | 0.277 | 154.260 | 105.801 | 154.612 | 0.047 | 154.252 | 0 | test |
| 0.015 | 141.789 | 152.172 | 140.626 | -0.017 | 33.375 | 1.443 | 397.924 | 0.307 | 0.279 | 140.227 | 94.682 | 140.984 | 0.041 | 119.335 | 1 | test |
| 0.018 | 157.326 | 167.891 | 156.404 | 0.027 | 38.809 | 1.400 | 442.439 | 0.318 | 0.280 | 156.080 | 106.738 | 156.603 | 0.029 | 86.028 | 1 | test |
| 0.019 | 149.108 | 161.059 | 148.218 | -0.008 | 42.945 | 1.666 | 419.418 | 0.305 | 0.267 | 147.910 | 100.765 | 148.386 | 0.034 | 111.092 | 1 | test |
| 0.019 | 136.963 | 145.203 | 136.202 | -0.027 | 52.368 | 1.431 | 385.193 | 0.305 | 0.283 | 136.007 | 93.069 | 136.381 | 0.099 | 154.029 | 2 | test |
| 0.017 | 154.075 | 162.266 | 153.468 | -0.005 | 13.899 | 1.383 | 434.440 | 0.290 | 0.294 | 153.268 | 104.847 | 153.629 | 0.047 | 114.932 | 1 | test |
| 0.023 | 160.585 | 168.000 | 160.122 | -0.020 | 19.640 | 1.472 | 452.974 | 0.284 | 0.277 | 159.946 | 109.399 | 160.223 | 0.098 | 134.272 | 0 | test |
| 0.019 | 150.260 | 158.589 | 149.750 | 0.002 | 24.574 | 1.447 | 423.228 | 0.280 | 0.281 | 149.628 | 102.805 | 149.804 | 0.050 | 137.423 | 1 | test |
| 0.015 | 154.659 | 166.406 | 153.341 | 0.033 | 25.055 | 1.325 | 433.415 | 0.302 | 0.329 | 152.886 | 103.478 | 153.427 | 0.037 | 103.543 | 1 | test |
| 0.022 | 159.943 | 169.188 | 159.502 | 0.000 | 83.667 | 1.563 | 451.125 | 0.285 | 0.251 | 159.363 | 109.523 | 159.559 | 0.043 | 123.027 | 0 | test |
| 0.019 | 152.853 | 162.172 | 152.158 | -0.025 | 57.130 | 1.648 | 430.546 | 0.292 | 0.274 | 151.881 | 104.198 | 152.329 | 0.053 | 120.781 | 1 | test |
| 0.016 | 150.867 | 160.062 | 150.211 | 0.016 | 18.564 | 1.434 | 425.206 | 0.299 | 0.284 | 149.935 | 102.188 | 150.357 | 0.052 | 181.016 | 1 | test |
| 0.020 | 159.839 | 171.047 | 159.271 | 0.023 | 60.807 | 1.606 | 450.868 | 0.314 | 0.281 | 159.000 | 109.023 | 159.411 | 0.104 | 162.633 | 0 | test |
| 0.026 | 162.105 | 169.938 | 161.724 | -0.027 | 57.363 | 1.470 | 457.264 | 0.278 | 0.300 | 161.626 | 109.465 | 161.755 | 0.083 | 118.883 | 0 | test |
| 0.015 | 158.101 | 175.469 | 154.242 | 0.059 | 53.108 | 0.658 | 436.292 | 0.321 | 0.291 | 152.655 | 98.025 | 155.176 | 0.044 | 98.612 | 1 | test |
| 0.023 | 146.753 | 156.141 | 145.969 | -0.002 | 42.836 | 1.566 | 412.915 | 0.293 | 0.240 | 145.731 | 98.330 | 146.206 | 0.068 | 97.627 | 1 | test |
| 0.015 | 155.039 | 165.969 | 154.189 | 0.001 | 22.032 | 1.417 | 436.265 | 0.296 | 0.299 | 153.905 | 104.619 | 154.352 | 0.058 | 135.240 | 1 | test |
| 0.020 | 159.968 | 168.516 | 159.309 | -0.011 | 52.194 | 1.471 | 450.707 | 0.297 | 0.277 | 159.085 | 107.904 | 159.467 | 0.113 | 123.229 | 0 | test |
| 0.020 | 157.853 | 166.797 | 157.352 | 0.009 | 33.481 | 1.551 | 445.059 | 0.271 | 0.241 | 157.089 | 107.563 | 157.505 | 0.116 | 123.983 | 0 | test |
| 0.021 | 154.891 | 163.375 | 154.354 | 0.027 | 49.163 | 1.467 | 436.437 | 0.292 | 0.286 | 154.135 | 104.673 | 154.412 | 0.053 | 111.239 | 1 | test |
| 0.019 | 161.426 | 168.938 | 160.843 | -0.032 | 22.227 | 1.542 | 455.121 | 0.295 | 0.271 | 160.596 | 109.303 | 161.007 | 0.047 | 93.128 | 0 | test |
| 0.022 | 161.373 | 169.531 | 160.926 | 0.019 | 69.993 | 1.427 | 455.433 | 0.287 | 0.287 | 160.675 | 109.374 | 160.980 | 0.044 | 78.737 | 0 | test |
| 0.018 | 133.001 | 141.842 | 132.210 | 0.022 | 27.089 | 1.438 | 373.903 | 0.286 | 0.265 | 131.933 | 89.698 | 132.377 | 0.046 | 131.286 | 2 | test |
| 0.020 | 157.663 | 166.328 | 157.118 | -0.004 | 31.472 | 1.615 | 444.411 | 0.272 | 0.233 | 156.911 | 107.892 | 157.270 | 0.018 | 52.864 | 0 | test |
| 0.021 | 154.942 | 164.291 | 154.376 | 0.028 | 29.095 | 1.489 | 436.883 | 0.288 | 0.292 | 154.043 | 104.287 | 154.509 | 0.047 | 115.187 | 1 | test |
| 0.017 | 135.882 | 153.328 | 131.417 | 0.011 | 26.539 | 0.695 | 372.266 | 0.327 | 0.359 | 129.886 | 82.782 | 131.944 | 0.069 | 93.069 | 2 | test |
| 0.021 | 164.924 | 173.625 | 164.199 | -0.020 | 66.982 | 1.424 | 464.614 | 0.304 | 0.281 | 163.930 | 111.689 | 164.364 | 0.039 | 79.952 | 0 | test |
| 0.018 | 162.066 | 170.359 | 161.463 | 0.000 | 26.816 | 1.542 | 456.704 | 0.315 | 0.296 | 161.280 | 109.580 | 161.559 | 0.108 | 124.428 | 0 | test |
| 0.023 | 156.066 | 163.578 | 155.603 | -0.047 | 63.611 | 1.431 | 440.606 | 0.277 | 0.283 | 155.428 | 106.624 | 155.715 | 0.094 | 137.774 | 1 | test |
| 0.023 | 153.454 | 161.062 | 152.962 | -0.012 | 54.729 | 1.494 | 433.243 | 0.278 | 0.270 | 152.739 | 103.978 | 153.103 | 0.047 | 105.154 | 0 | test |
| 0.019 | 154.253 | 164.938 | 153.642 | 0.034 | 49.442 | 1.517 | 434.617 | 0.284 | 0.279 | 153.432 | 105.284 | 153.752 | 0.054 | 96.439 | 1 | test |
| 0.015 | 163.404 | 180.153 | 160.558 | 0.066 | 46.475 | 0.838 | 453.948 | 0.328 | 0.349 | 159.416 | 104.564 | 160.688 | 0.030 | 69.795 | 0 | test |
| 0.023 | 161.324 | 168.516 | 160.866 | -0.013 | 43.885 | 1.502 | 455.204 | 0.295 | 0.256 | 160.660 | 109.700 | 160.995 | 0.098 | 105.986 | 0 | test |
| 0.023 | 152.063 | 161.625 | 151.436 | 0.018 | 40.745 | 1.584 | 428.237 | 0.292 | 0.274 | 151.148 | 101.980 | 151.540 | 0.052 | 87.522 | 1 | test |
| 0.018 | 166.470 | 172.906 | 166.085 | 0.025 | 27.965 | 1.487 | 470.023 | 0.272 | 0.234 | 165.910 | 114.147 | 166.154 | 0.074 | 144.513 | 0 | test |
| 0.020 | 155.324 | 164.484 | 154.806 | 0.019 | 54.971 | 1.552 | 437.714 | 0.272 | 0.251 | 154.618 | 105.286 | 154.907 | 0.048 | 125.499 | 1 | test |
| 0.019 | 161.360 | 169.391 | 160.856 | -0.002 | 34.065 | 1.537 | 454.965 | 0.293 | 0.250 | 160.691 | 110.621 | 160.984 | 0.098 | 137.848 | 0 | test |
| 0.019 | 151.531 | 159.125 | 151.022 | 0.018 | 25.033 | 1.459 | 427.114 | 0.277 | 0.270 | 150.794 | 104.038 | 151.105 | 0.048 | 133.703 | 1 | test |
| 0.020 | 150.845 | 161.438 | 150.098 | -0.038 | 44.710 | 1.469 | 424.786 | 0.290 | 0.256 | 149.811 | 102.270 | 150.356 | 0.055 | 96.521 | 1 | test |
| 0.020 | 144.334 | 152.602 | 143.641 | 0.031 | 29.071 | 1.383 | 406.347 | 0.299 | 0.293 | 143.389 | 98.656 | 143.756 | 0.068 | 101.297 | 1 | test |
| 0.019 | 159.886 | 173.484 | 157.793 | 0.033 | 43.911 | 1.057 | 446.021 | 0.306 | 0.271 | 156.971 | 105.710 | 158.428 | 0.023 | 38.562 | 1 | test |
| 0.019 | 148.427 | 157.106 | 147.752 | -0.014 | 27.750 | 1.457 | 418.028 | 0.298 | 0.259 | 147.548 | 99.998 | 147.940 | 0.055 | 161.184 | 1 | test |
| 0.019 | 163.086 | 172.906 | 162.455 | 0.010 | 68.214 | 1.433 | 459.966 | 0.296 | 0.283 | 162.217 | 111.258 | 162.594 | 0.043 | 92.558 | 0 | test |
| 0.016 | 145.492 | 155.203 | 144.828 | 0.010 | 35.291 | 1.394 | 409.308 | 0.290 | 0.315 | 144.614 | 99.252 | 144.847 | 0.067 | 168.844 | 1 | test |
| 0.016 | 145.203 | 153.500 | 144.460 | 0.024 | 20.489 | 1.365 | 408.645 | 0.301 | 0.289 | 144.229 | 98.257 | 144.580 | 0.067 | 125.019 | 1 | test |
| 0.019 | 159.436 | 174.125 | 156.786 | 0.015 | 38.011 | 0.881 | 443.649 | 0.320 | 0.329 | 155.797 | 105.007 | 157.214 | 0.027 | 43.486 | 1 | test |
| 0.019 | 127.569 | 141.312 | 123.118 | 0.000 | 19.402 | 0.781 | 348.910 | 0.350 | 0.346 | 121.532 | 77.857 | 123.907 | 0.082 | 79.522 | 2 | test |
| 0.021 | 161.299 | 176.406 | 158.322 | 0.045 | 34.719 | 0.796 | 447.547 | 0.336 | 0.333 | 157.135 | 104.029 | 158.688 | 0.029 | 44.549 | 0 | test |
| 0.019 | 164.178 | 181.578 | 160.579 | 0.060 | 36.232 | 0.643 | 454.122 | 0.319 | 0.301 | 158.942 | 103.706 | 161.292 | 0.034 | 51.047 | 1 | test |
| 0.018 | 161.172 | 175.502 | 158.686 | 0.042 | 56.604 | 0.959 | 448.401 | 0.337 | 0.321 | 157.915 | 103.595 | 159.159 | 0.026 | 57.032 | 0 | test |
| 0.019 | 162.321 | 178.047 | 159.083 | 0.066 | 41.459 | 0.776 | 449.864 | 0.336 | 0.319 | 157.784 | 104.191 | 159.479 | 0.032 | 74.277 | 0 | test |
| 0.021 | 154.588 | 170.469 | 150.937 | 0.031 | 34.587 | 0.759 | 427.286 | 0.343 | 0.331 | 149.644 | 98.045 | 151.480 | 0.038 | 64.092 | 1 | test |
| 0.018 | 166.146 | 181.528 | 163.173 | 0.091 | 27.603 | 0.832 | 462.085 | 0.344 | 0.328 | 161.850 | 106.811 | 163.744 | 0.028 | 44.145 | 1 | test |
| 0.015 | 165.875 | 183.188 | 162.118 | 0.108 | 33.297 | 0.669 | 458.396 | 0.341 | 0.323 | 160.438 | 105.491 | 162.663 | 0.035 | 81.975 | 0 | test |
| 0.022 | 153.608 | 162.547 | 152.946 | 0.007 | 75.023 | 1.471 | 432.507 | 0.284 | 0.289 | 152.680 | 103.791 | 153.016 | 0.052 | 112.068 | 1 | test |
| 0.020 | 154.305 | 163.625 | 153.634 | 0.027 | 31.858 | 1.461 | 434.596 | 0.281 | 0.283 | 153.363 | 104.780 | 153.765 | 0.053 | 89.159 | 1 | test |
| 0.020 | 169.818 | 178.688 | 169.364 | -0.012 | 49.817 | 1.463 | 479.814 | 0.284 | 0.253 | 169.168 | 116.721 | 169.501 | 0.061 | 113.204 | 0 | test |
| 0.020 | 154.199 | 164.734 | 153.491 | 0.009 | 38.471 | 1.380 | 434.303 | 0.306 | 0.300 | 153.195 | 105.141 | 153.590 | 0.058 | 85.020 | 1 | test |
| 0.020 | 150.822 | 159.828 | 150.270 | 0.005 | 30.401 | 1.420 | 424.832 | 0.285 | 0.257 | 150.113 | 101.708 | 150.350 | 0.053 | 112.676 | 1 | test |
| 0.018 | 155.158 | 163.500 | 154.738 | -0.014 | 15.892 | 1.388 | 437.699 | 0.266 | 0.277 | 154.554 | 106.026 | 154.812 | 0.106 | 119.046 | 1 | test |
| 0.022 | 155.274 | 163.281 | 154.754 | -0.028 | 39.451 | 1.476 | 437.989 | 0.285 | 0.248 | 154.590 | 105.673 | 154.921 | 0.112 | 126.738 | 1 | test |
| 0.028 | 162.635 | 176.719 | 160.365 | 0.047 | 41.962 | 0.966 | 453.667 | 0.325 | 0.310 | 159.447 | 106.402 | 160.789 | 0.026 | 36.915 | 0 | test |
| 0.018 | 154.318 | 163.891 | 153.363 | -0.015 | 22.862 | 1.404 | 433.945 | 0.314 | 0.311 | 153.017 | 103.260 | 153.547 | 0.056 | 120.044 | 0 | test |
| 0.019 | 158.344 | 166.641 | 157.592 | -0.013 | 19.280 | 1.446 | 446.038 | 0.309 | 0.280 | 157.271 | 107.727 | 157.843 | 0.050 | 75.685 | 1 | test |
| 0.021 | 152.931 | 161.469 | 152.305 | 0.003 | 40.886 | 1.421 | 430.726 | 0.296 | 0.298 | 152.102 | 103.688 | 152.424 | 0.047 | 104.829 | 1 | test |
| 0.019 | 165.824 | 174.375 | 165.210 | -0.010 | 56.497 | 1.420 | 467.437 | 0.305 | 0.282 | 165.029 | 113.752 | 165.295 | 0.089 | 131.235 | 0 | test |
| 0.019 | 151.440 | 160.594 | 150.756 | -0.018 | 30.289 | 1.459 | 426.516 | 0.281 | 0.258 | 150.530 | 103.272 | 150.951 | 0.051 | 115.308 | 0 | test |
| 0.018 | 148.725 | 159.656 | 147.780 | -0.001 | 20.693 | 1.425 | 418.092 | 0.298 | 0.301 | 147.510 | 99.464 | 147.965 | 0.034 | 121.061 | 1 | test |
| 0.024 | 128.593 | 138.594 | 127.758 | 0.015 | 24.170 | 1.420 | 361.509 | 0.304 | 0.279 | 127.484 | 86.652 | 127.908 | 0.050 | 81.415 | 2 | test |
| 0.021 | 154.934 | 162.953 | 154.490 | 0.007 | 58.321 | 1.644 | 436.937 | 0.260 | 0.238 | 154.202 | 105.605 | 154.606 | 0.044 | 96.309 | 1 | test |
| 0.019 | 154.751 | 165.000 | 153.851 | -0.019 | 26.172 | 1.489 | 435.295 | 0.287 | 0.260 | 153.565 | 103.929 | 154.125 | 0.055 | 115.947 | 1 | test |
| 0.021 | 153.412 | 161.219 | 152.994 | 0.017 | 57.973 | 1.506 | 433.018 | 0.277 | 0.256 | 152.858 | 104.481 | 153.076 | 0.112 | 141.974 | 0 | test |
| 0.019 | 151.749 | 160.250 | 151.120 | 0.001 | 32.572 | 1.449 | 427.625 | 0.279 | 0.261 | 150.883 | 103.114 | 151.265 | 0.051 | 123.843 | 1 | test |
| 0.020 | 158.887 | 168.422 | 158.206 | -0.016 | 61.037 | 1.604 | 447.751 | 0.310 | 0.269 | 157.800 | 107.941 | 158.403 | 0.049 | 95.742 | 1 | test |
| 0.019 | 145.387 | 153.859 | 144.770 | 0.013 | 27.029 | 1.408 | 409.797 | 0.293 | 0.273 | 144.545 | 97.856 | 144.911 | 0.065 | 105.824 | 1 | test |
| 0.020 | 156.831 | 164.625 | 156.331 | -0.008 | 30.493 | 1.473 | 442.247 | 0.299 | 0.250 | 156.161 | 106.767 | 156.454 | 0.116 | 127.074 | 0 | test |
| 0.019 | 154.777 | 163.594 | 154.161 | -0.011 | 31.784 | 1.475 | 436.458 | 0.281 | 0.289 | 153.879 | 105.089 | 154.300 | 0.047 | 111.311 | 1 | test |
| 0.018 | 162.981 | 181.375 | 159.855 | 0.058 | 37.857 | 0.744 | 452.307 | 0.321 | 0.275 | 158.357 | 103.937 | 160.747 | 0.033 | 80.905 | 0 | test |
| 0.019 | 152.559 | 161.875 | 151.833 | 0.028 | 41.239 | 1.426 | 429.334 | 0.299 | 0.290 | 151.641 | 103.176 | 151.920 | 0.029 | 78.559 | 1 | test |
| 0.017 | 156.161 | 164.766 | 155.630 | 0.016 | 33.984 | 1.453 | 440.299 | 0.283 | 0.280 | 155.388 | 105.592 | 155.719 | 0.115 | 199.954 | 1 | test |
| 0.017 | 146.458 | 157.500 | 144.933 | 0.002 | 27.315 | 1.304 | 410.010 | 0.326 | 0.310 | 144.435 | 97.545 | 145.279 | 0.043 | 111.767 | 1 | test |
| 0.021 | 156.404 | 165.812 | 155.746 | -0.025 | 41.205 | 1.412 | 440.619 | 0.283 | 0.321 | 155.554 | 107.260 | 155.834 | 0.047 | 80.754 | 0 | test |
| 0.022 | 145.052 | 153.562 | 144.410 | 0.000 | 39.258 | 1.427 | 408.471 | 0.277 | 0.257 | 144.170 | 98.141 | 144.553 | 0.067 | 102.314 | 1 | test |
| 0.022 | 164.914 | 172.422 | 164.487 | -0.001 | 73.995 | 1.523 | 465.242 | 0.286 | 0.266 | 164.195 | 112.348 | 164.569 | 0.044 | 61.999 | 0 | test |
| 0.016 | 151.112 | 160.406 | 150.200 | -0.007 | 27.123 | 1.460 | 424.887 | 0.294 | 0.266 | 149.924 | 101.909 | 150.458 | 0.031 | 91.976 | 1 | test |
| 0.020 | 152.233 | 159.500 | 151.917 | 0.008 | 52.353 | 1.429 | 429.388 | 0.285 | 0.286 | 151.822 | 103.589 | 151.870 | 0.115 | 147.779 | 1 | test |
| 0.015 | 143.580 | 152.922 | 142.755 | 0.002 | 27.308 | 1.554 | 403.775 | 0.291 | 0.252 | 142.462 | 95.985 | 142.995 | 0.081 | 173.390 | 1 | test |
| 0.017 | 160.424 | 170.391 | 159.475 | -0.012 | 34.814 | 1.406 | 451.091 | 0.311 | 0.296 | 159.140 | 108.387 | 159.708 | 0.049 | 121.363 | 1 | test |
| 0.019 | 126.852 | 134.875 | 126.210 | -0.028 | 24.593 | 1.452 | 357.063 | 0.273 | 0.276 | 126.004 | 85.849 | 126.348 | 0.119 | 188.914 | 2 | test |
| 0.017 | 150.678 | 160.641 | 149.925 | 0.027 | 21.277 | 1.505 | 424.013 | 0.286 | 0.245 | 149.557 | 102.164 | 150.126 | 0.061 | 110.223 | 1 | test |
| 0.018 | 154.076 | 162.312 | 153.518 | 0.004 | 24.354 | 1.500 | 434.364 | 0.293 | 0.265 | 153.310 | 105.061 | 153.669 | 0.050 | 108.589 | 1 | test |
| 0.019 | 154.565 | 165.031 | 153.837 | -0.016 | 28.092 | 1.548 | 435.143 | 0.286 | 0.258 | 153.575 | 104.663 | 154.022 | 0.029 | 89.311 | 0 | test |
| 0.023 | 154.806 | 162.500 | 154.371 | 0.002 | 41.250 | 1.520 | 436.622 | 0.280 | 0.236 | 154.198 | 105.383 | 154.462 | 0.114 | 171.810 | 1 | test |
| 0.016 | 148.672 | 157.438 | 147.949 | 0.018 | 32.477 | 1.468 | 418.422 | 0.307 | 0.278 | 147.710 | 100.890 | 148.095 | 0.062 | 145.222 | 1 | test |
| 0.019 | 148.383 | 158.500 | 147.621 | -0.007 | 37.271 | 1.457 | 417.212 | 0.290 | 0.309 | 147.379 | 99.742 | 147.665 | 0.033 | 94.792 | 1 | test |
| 0.015 | 160.930 | 173.031 | 159.950 | -0.009 | 30.940 | 1.429 | 452.587 | 0.301 | 0.296 | 159.621 | 108.478 | 160.123 | 0.018 | 70.127 | 0 | test |
| 0.019 | 153.414 | 162.406 | 152.854 | 0.028 | 43.062 | 1.475 | 432.565 | 0.276 | 0.255 | 152.613 | 104.084 | 152.950 | 0.050 | 109.935 | 1 | test |
| 0.013 | 152.938 | 161.406 | 152.464 | 0.031 | 26.093 | 1.164 | 431.656 | 0.291 | 0.285 | 152.282 | 102.552 | 152.497 | 0.045 | 72.132 | 1 | test |
| 0.016 | 145.207 | 154.594 | 144.320 | -0.009 | 9.201 | 1.426 | 407.939 | 0.304 | 0.310 | 144.108 | 97.736 | 144.462 | 0.036 | 119.842 | 2 | test |
| 0.024 | 165.818 | 179.688 | 163.614 | 0.009 | 64.648 | 0.998 | 463.090 | 0.314 | 0.293 | 162.730 | 108.868 | 164.266 | 0.039 | 51.300 | 0 | test |
| 0.020 | 157.825 | 166.188 | 157.337 | -0.026 | 27.393 | 1.507 | 445.080 | 0.288 | 0.251 | 157.175 | 108.257 | 157.457 | 0.046 | 88.970 | 0 | test |
| 0.018 | 144.480 | 156.938 | 143.339 | 0.034 | 15.376 | 1.342 | 405.469 | 0.316 | 0.314 | 142.874 | 96.602 | 143.507 | 0.041 | 101.940 | 1 | test |
| 0.020 | 162.251 | 171.875 | 161.467 | -0.030 | 56.544 | 1.443 | 457.277 | 0.288 | 0.278 | 161.220 | 110.132 | 161.688 | 0.047 | 64.786 | 0 | test |
| 0.019 | 154.207 | 162.959 | 153.501 | 0.000 | 23.551 | 1.536 | 434.165 | 0.312 | 0.274 | 153.300 | 103.905 | 153.670 | 0.029 | 106.526 | 0 | test |
| 0.018 | 155.486 | 163.812 | 155.043 | -0.003 | 41.725 | 1.477 | 438.621 | 0.286 | 0.250 | 154.873 | 106.154 | 155.141 | 0.103 | 146.082 | 1 | test |
| 0.017 | 154.614 | 164.942 | 153.858 | -0.007 | 27.244 | 1.433 | 435.534 | 0.317 | 0.288 | 153.598 | 104.918 | 154.038 | 0.051 | 139.045 | 1 | test |
| 0.020 | 159.811 | 172.531 | 157.838 | -0.007 | 39.864 | 1.124 | 446.676 | 0.326 | 0.326 | 157.241 | 105.910 | 158.243 | 0.042 | 59.404 | 0 | test |
| 0.022 | 157.895 | 172.938 | 154.313 | 0.051 | 39.928 | 0.758 | 436.633 | 0.324 | 0.343 | 153.005 | 99.935 | 154.981 | 0.039 | 53.879 | 1 | test |
| 0.016 | 141.052 | 158.375 | 135.983 | 0.031 | 28.286 | 0.585 | 384.811 | 0.322 | 0.314 | 133.833 | 83.900 | 136.977 | 0.072 | 86.862 | 2 | test |
| 0.015 | 120.531 | 135.953 | 116.883 | -0.013 | 19.736 | 0.968 | 331.001 | 0.337 | 0.330 | 115.653 | 73.817 | 117.332 | 0.083 | 130.094 | 3 | test |
| 0.017 | 115.644 | 130.359 | 111.527 | -0.031 | 32.580 | 0.830 | 315.772 | 0.316 | 0.293 | 109.949 | 68.807 | 112.566 | 0.096 | 120.682 | 3 | test |
| 0.019 | 110.485 | 124.562 | 106.503 | -0.007 | 25.799 | 0.942 | 301.586 | 0.339 | 0.332 | 105.099 | 65.794 | 107.154 | 0.109 | 103.704 | 3 | test |
| 0.016 | 112.693 | 127.500 | 108.357 | -0.044 | 20.404 | 0.860 | 306.910 | 0.322 | 0.299 | 106.891 | 66.633 | 109.515 | 0.095 | 113.694 | 3 | test |
| 0.020 | 112.569 | 126.828 | 110.529 | -0.009 | 26.592 | 2.122 | 313.986 | 0.358 | 0.273 | 110.064 | 72.841 | 110.976 | 0.053 | 131.527 | 3 | test |
| 0.018 | 110.045 | 126.531 | 107.232 | 0.008 | 19.369 | 1.622 | 304.574 | 0.351 | 0.294 | 106.455 | 68.951 | 107.732 | 0.082 | 128.865 | 3 | test |
| 0.016 | 119.814 | 136.042 | 114.405 | -0.026 | 17.581 | 0.689 | 324.450 | 0.339 | 0.328 | 112.506 | 69.372 | 115.417 | 0.097 | 140.066 | 3 | test |
| 0.017 | 95.022 | 112.328 | 92.517 | -0.024 | 43.332 | 1.647 | 262.769 | 0.331 | 0.261 | 91.712 | 58.848 | 93.268 | 0.107 | 181.225 | 3 | test |
| 0.017 | 113.313 | 127.734 | 110.616 | -0.008 | 23.179 | 2.070 | 314.542 | 0.370 | 0.232 | 109.976 | 71.730 | 111.439 | 0.062 | 169.001 | 3 | test |
| 0.015 | 116.065 | 129.420 | 113.096 | -0.003 | 17.684 | 1.285 | 320.625 | 0.318 | 0.273 | 112.116 | 72.326 | 113.871 | 0.071 | 150.543 | 3 | test |
| 0.016 | 107.514 | 119.641 | 103.424 | -0.046 | 20.276 | 0.987 | 292.871 | 0.335 | 0.303 | 102.064 | 64.415 | 104.720 | 0.111 | 117.060 | 3 | test |
| 0.017 | 106.707 | 125.497 | 98.311 | -0.135 | 27.079 | 1.135 | 285.217 | 0.368 | 0.231 | 96.318 | 55.616 | 101.387 | 0.078 | 265.260 | 3 | test |
| 0.020 | 114.494 | 129.984 | 111.317 | -0.014 | 29.628 | 1.069 | 315.180 | 0.333 | 0.327 | 110.225 | 71.030 | 111.946 | 0.080 | 76.436 | 3 | test |
| 0.018 | 101.896 | 114.656 | 97.344 | -0.049 | 25.112 | 0.917 | 275.853 | 0.339 | 0.328 | 95.855 | 59.222 | 98.209 | 0.126 | 142.205 | 3 | test |
| 0.018 | 108.300 | 121.356 | 105.295 | -0.002 | 23.813 | 1.097 | 297.858 | 0.324 | 0.316 | 104.193 | 66.916 | 105.778 | 0.094 | 117.877 | 3 | test |
| 0.016 | 122.865 | 137.797 | 118.178 | -0.013 | 20.314 | 0.731 | 334.697 | 0.319 | 0.308 | 116.344 | 72.729 | 119.308 | 0.088 | 119.705 | 3 | test |
| 0.017 | 118.029 | 131.656 | 114.578 | -0.013 | 16.639 | 1.028 | 324.366 | 0.342 | 0.311 | 113.512 | 73.068 | 115.412 | 0.079 | 80.122 | 2 | test |
| 0.017 | 111.849 | 125.344 | 107.694 | -0.004 | 29.138 | 0.848 | 304.843 | 0.320 | 0.312 | 106.175 | 66.568 | 108.770 | 0.107 | 120.133 | 3 | test |
| 0.016 | 103.414 | 115.841 | 101.703 | 0.023 | 26.676 | 1.434 | 287.817 | 0.306 | 0.312 | 101.089 | 66.453 | 101.931 | 0.071 | 141.481 | 3 | test |
| 0.017 | 124.408 | 137.422 | 121.153 | -0.012 | 15.662 | 0.942 | 342.829 | 0.327 | 0.283 | 119.913 | 77.915 | 121.912 | 0.063 | 89.724 | 2 | test |
| 0.016 | 121.153 | 134.281 | 117.987 | -0.013 | 21.221 | 1.018 | 334.016 | 0.335 | 0.322 | 116.947 | 76.506 | 118.560 | 0.067 | 94.019 | 2 | test |
| 0.018 | 104.961 | 118.641 | 101.528 | -0.025 | 27.915 | 1.105 | 287.794 | 0.334 | 0.324 | 100.407 | 63.978 | 102.428 | 0.110 | 118.996 | 3 | test |
| 0.018 | 114.615 | 129.438 | 109.390 | -0.038 | 24.746 | 0.717 | 309.879 | 0.318 | 0.285 | 107.456 | 65.932 | 111.022 | 0.106 | 117.178 | 3 | test |
| 0.015 | 116.259 | 129.156 | 112.015 | -0.023 | 15.704 | 0.848 | 317.420 | 0.339 | 0.317 | 110.564 | 70.392 | 112.974 | 0.094 | 97.880 | 3 | test |
| 0.014 | 97.113 | 111.458 | 94.174 | -0.050 | 30.686 | 1.346 | 266.832 | 0.314 | 0.247 | 93.198 | 58.889 | 95.235 | 0.107 | 160.921 | 3 | test |
| 0.018 | 109.082 | 122.844 | 104.536 | -0.030 | 28.835 | 0.835 | 296.168 | 0.326 | 0.310 | 102.967 | 64.190 | 105.607 | 0.110 | 87.421 | 3 | test |
| 0.018 | 131.818 | 143.453 | 129.889 | 0.029 | 23.026 | 1.566 | 367.582 | 0.347 | 0.274 | 129.227 | 86.771 | 130.350 | 0.037 | 96.441 | 2 | test |
| 0.020 | 129.552 | 142.797 | 125.852 | -0.008 | 15.235 | 0.904 | 356.251 | 0.341 | 0.313 | 124.602 | 81.188 | 126.674 | 0.064 | 81.452 | 2 | test |
| 0.018 | 130.644 | 149.969 | 127.206 | -0.017 | 49.921 | 0.892 | 359.932 | 0.331 | 0.319 | 126.061 | 80.360 | 127.920 | 0.063 | 61.644 | 2 | test |
| 0.017 | 123.232 | 137.625 | 119.329 | -0.002 | 25.675 | 0.805 | 337.486 | 0.318 | 0.354 | 117.852 | 75.164 | 119.958 | 0.081 | 104.921 | 2 | test |
| 0.019 | 136.289 | 150.938 | 132.455 | -0.004 | 26.255 | 0.849 | 375.273 | 0.340 | 0.308 | 131.213 | 84.452 | 133.377 | 0.055 | 70.112 | 2 | test |
| 0.017 | 121.764 | 134.281 | 119.270 | 0.000 | 20.451 | 1.296 | 337.892 | 0.321 | 0.294 | 118.536 | 77.773 | 119.906 | 0.051 | 115.949 | 2 | test |
| 0.016 | 96.203 | 112.062 | 92.556 | -0.021 | 29.164 | 1.671 | 263.859 | 0.337 | 0.245 | 91.528 | 57.177 | 93.521 | 0.116 | 217.467 | 3 | test |
| 0.018 | 124.072 | 138.469 | 119.654 | -0.003 | 25.596 | 0.758 | 338.847 | 0.323 | 0.312 | 118.037 | 73.893 | 120.767 | 0.091 | 92.103 | 2 | test |
| 0.015 | 89.994 | 103.797 | 87.259 | -0.019 | 37.594 | 1.346 | 247.233 | 0.307 | 0.294 | 86.269 | 54.449 | 87.990 | 0.134 | 169.960 | 3 | test |
| 0.015 | 114.098 | 126.688 | 111.631 | -0.002 | 23.208 | 1.389 | 316.411 | 0.319 | 0.278 | 110.805 | 72.671 | 112.240 | 0.064 | 124.767 | 3 | test |
| 0.020 | 129.987 | 145.750 | 125.869 | -0.013 | 31.776 | 0.772 | 356.439 | 0.335 | 0.364 | 124.513 | 79.569 | 126.416 | 0.075 | 87.929 | 2 | test |
| 0.018 | 133.427 | 143.500 | 131.959 | 0.004 | 20.288 | 1.411 | 373.289 | 0.339 | 0.268 | 131.471 | 88.288 | 132.368 | 0.030 | 80.826 | 2 | test |
| 0.021 | 132.950 | 145.750 | 129.673 | 0.011 | 29.042 | 0.939 | 366.848 | 0.335 | 0.319 | 128.486 | 83.094 | 130.555 | 0.057 | 60.155 | 2 | test |
| 0.016 | 126.886 | 141.766 | 121.813 | -0.017 | 26.728 | 0.676 | 344.938 | 0.323 | 0.281 | 119.866 | 74.546 | 123.302 | 0.087 | 89.104 | 2 | test |
| 0.019 | 123.343 | 139.156 | 119.845 | -0.010 | 24.115 | 1.021 | 339.454 | 0.328 | 0.288 | 118.766 | 76.068 | 121.024 | 0.073 | 94.496 | 2 | test |
| 0.016 | 118.956 | 134.238 | 113.951 | -0.045 | 26.072 | 0.699 | 323.059 | 0.337 | 0.328 | 112.122 | 69.231 | 115.098 | 0.099 | 89.134 | 3 | test |
| 0.015 | 125.594 | 138.375 | 121.844 | -0.003 | 21.074 | 0.890 | 345.188 | 0.334 | 0.336 | 120.576 | 77.615 | 122.430 | 0.076 | 107.153 | 2 | test |
| 0.018 | 126.475 | 139.312 | 123.533 | 0.011 | 29.313 | 1.023 | 349.285 | 0.327 | 0.342 | 122.562 | 80.346 | 123.882 | 0.060 | 92.541 | 2 | test |
| 0.018 | 126.621 | 142.578 | 121.767 | -0.008 | 16.375 | 1.201 | 346.766 | 0.370 | 0.305 | 120.528 | 76.567 | 122.668 | 0.079 | 155.340 | 2 | test |
| 0.017 | 110.990 | 123.625 | 109.805 | 0.011 | 18.107 | 1.481 | 310.595 | 0.301 | 0.278 | 109.387 | 73.375 | 110.089 | 0.045 | 117.073 | 3 | test |
| 0.014 | 108.619 | 125.250 | 103.637 | -0.049 | 23.675 | 0.788 | 293.762 | 0.330 | 0.297 | 101.848 | 61.753 | 104.872 | 0.110 | 130.530 | 3 | test |
| 0.017 | 114.971 | 127.938 | 110.395 | -0.030 | 25.050 | 0.832 | 313.033 | 0.342 | 0.346 | 108.938 | 68.530 | 111.086 | 0.101 | 97.096 | 3 | test |
| 0.016 | 125.055 | 140.038 | 120.442 | -0.019 | 21.094 | 0.798 | 341.504 | 0.350 | 0.317 | 118.954 | 75.075 | 121.171 | 0.088 | 136.375 | 2 | test |
| 0.017 | 120.608 | 134.906 | 115.794 | -0.042 | 18.061 | 0.736 | 327.744 | 0.342 | 0.349 | 114.183 | 71.314 | 116.512 | 0.095 | 122.259 | 2 | test |
| 0.017 | 121.819 | 139.344 | 116.199 | -0.039 | 32.114 | 0.645 | 329.706 | 0.336 | 0.324 | 114.250 | 69.152 | 117.404 | 0.093 | 125.998 | 3 | test |
| 0.021 | 123.778 | 136.516 | 120.612 | 0.006 | 34.877 | 0.967 | 341.321 | 0.326 | 0.346 | 119.481 | 78.041 | 121.042 | 0.063 | 85.066 | 2 | test |
| 0.018 | 113.016 | 126.484 | 110.291 | 0.004 | 32.838 | 1.475 | 313.000 | 0.317 | 0.307 | 109.299 | 71.540 | 110.823 | 0.075 | 153.357 | 3 | test |
| 0.017 | 108.714 | 126.812 | 104.814 | -0.041 | 37.324 | 1.181 | 297.930 | 0.332 | 0.295 | 103.658 | 63.799 | 105.624 | 0.101 | 122.303 | 3 | test |
| 0.017 | 121.671 | 134.688 | 117.331 | -0.019 | 18.254 | 0.843 | 332.270 | 0.354 | 0.313 | 115.883 | 73.398 | 118.486 | 0.086 | 110.611 | 2 | test |
| 0.019 | 125.362 | 135.625 | 123.850 | -0.030 | 19.359 | 1.366 | 350.622 | 0.327 | 0.320 | 123.374 | 83.050 | 124.167 | 0.070 | 152.093 | 2 | test |
| 0.015 | 120.734 | 135.245 | 116.497 | 0.003 | 22.148 | 0.933 | 329.733 | 0.339 | 0.304 | 115.061 | 72.937 | 117.366 | 0.086 | 111.674 | 2 | test |
| 0.016 | 121.713 | 136.719 | 116.974 | -0.033 | 16.041 | 0.889 | 332.228 | 0.333 | 0.319 | 115.536 | 72.716 | 117.820 | 0.091 | 156.672 | 3 | test |
| 0.019 | 137.259 | 150.203 | 134.350 | 0.012 | 21.997 | 1.026 | 380.257 | 0.336 | 0.305 | 133.336 | 88.038 | 134.991 | 0.043 | 80.199 | 2 | test |
| 0.015 | 126.731 | 139.797 | 123.089 | -0.008 | 16.871 | 0.888 | 348.527 | 0.316 | 0.303 | 121.897 | 79.059 | 124.170 | 0.068 | 85.489 | 2 | test |
| 0.017 | 127.540 | 142.250 | 123.521 | -0.007 | 17.987 | 1.038 | 350.757 | 0.340 | 0.326 | 122.237 | 78.245 | 124.283 | 0.070 | 104.464 | 2 | test |
| 0.017 | 127.290 | 138.547 | 125.473 | 0.004 | 15.784 | 1.278 | 354.852 | 0.327 | 0.318 | 124.865 | 83.472 | 125.793 | 0.039 | 91.326 | 2 | test |
| 0.016 | 130.656 | 143.500 | 127.594 | -0.026 | 20.092 | 0.959 | 361.392 | 0.327 | 0.352 | 126.607 | 83.138 | 127.928 | 0.052 | 73.836 | 2 | test |
| 0.018 | 105.342 | 121.062 | 102.623 | -0.010 | 15.524 | 1.531 | 291.242 | 0.326 | 0.294 | 101.873 | 65.886 | 103.332 | 0.082 | 158.273 | 3 | test |
| 0.018 | 114.624 | 132.516 | 111.925 | -0.023 | 16.840 | 2.142 | 318.819 | 0.359 | 0.258 | 111.358 | 72.515 | 112.571 | 0.059 | 155.317 | 3 | test |
| 0.019 | 126.725 | 139.500 | 124.120 | -0.008 | 24.398 | 1.596 | 352.188 | 0.324 | 0.271 | 123.389 | 81.757 | 124.988 | 0.044 | 131.958 | 2 | test |
| 0.018 | 117.454 | 129.203 | 115.477 | -0.021 | 34.514 | 1.538 | 327.523 | 0.342 | 0.284 | 114.915 | 76.215 | 115.944 | 0.046 | 124.176 | 3 | test |
| 0.018 | 101.333 | 112.938 | 98.133 | -0.010 | 26.517 | 1.122 | 277.537 | 0.324 | 0.321 | 97.053 | 61.668 | 98.809 | 0.114 | 123.427 | 3 | test |
| 0.013 | 98.159 | 111.594 | 92.758 | -0.081 | 19.527 | 0.806 | 263.107 | 0.344 | 0.314 | 90.899 | 54.297 | 93.826 | 0.128 | 152.047 | 3 | test |
| 0.018 | 110.899 | 124.406 | 107.136 | -0.028 | 20.130 | 0.897 | 303.397 | 0.327 | 0.345 | 105.776 | 66.755 | 107.718 | 0.103 | 106.555 | 3 | test |
| 0.018 | 128.807 | 141.539 | 125.662 | -0.011 | 22.510 | 0.938 | 355.387 | 0.328 | 0.342 | 124.639 | 81.536 | 126.104 | 0.058 | 75.800 | 2 | test |
| 0.015 | 111.737 | 125.266 | 107.328 | -0.037 | 18.857 | 0.844 | 304.020 | 0.328 | 0.339 | 105.855 | 66.288 | 107.964 | 0.106 | 99.788 | 3 | test |
| 0.026 | 137.555 | 151.641 | 134.273 | 0.003 | 19.164 | 1.028 | 379.427 | 0.340 | 0.347 | 133.203 | 87.807 | 134.796 | 0.054 | 86.394 | 2 | test |
| 0.018 | 125.773 | 140.562 | 121.386 | -0.019 | 20.138 | 0.811 | 344.126 | 0.351 | 0.313 | 119.988 | 76.152 | 122.446 | 0.079 | 109.118 | 2 | test |
| 0.019 | 123.124 | 136.000 | 121.333 | 0.019 | 11.342 | 1.284 | 343.164 | 0.317 | 0.343 | 120.642 | 79.593 | 121.517 | 0.043 | 96.074 | 2 | test |
| 0.016 | 121.759 | 135.531 | 120.238 | -0.016 | 14.683 | 1.453 | 340.473 | 0.322 | 0.287 | 119.784 | 79.538 | 120.608 | 0.039 | 108.206 | 2 | test |
| 0.016 | 103.169 | 118.688 | 98.546 | -0.049 | 20.532 | 0.856 | 279.609 | 0.325 | 0.329 | 96.913 | 58.850 | 99.353 | 0.124 | 146.300 | 3 | test |
| 0.015 | 125.815 | 139.683 | 122.331 | 0.000 | 14.322 | 0.881 | 346.369 | 0.327 | 0.360 | 121.140 | 78.088 | 122.670 | 0.071 | 91.097 | 2 | test |
| 0.018 | 120.940 | 133.469 | 118.134 | -0.014 | 14.391 | 1.117 | 334.706 | 0.335 | 0.324 | 117.227 | 77.027 | 118.763 | 0.061 | 99.434 | 2 | test |
| 0.019 | 124.981 | 139.312 | 121.783 | -0.017 | 20.777 | 0.986 | 344.772 | 0.336 | 0.324 | 120.683 | 78.926 | 122.395 | 0.067 | 108.406 | 2 | test |
| 0.019 | 126.134 | 139.719 | 122.727 | -0.008 | 23.004 | 0.890 | 347.480 | 0.318 | 0.311 | 121.515 | 78.924 | 123.460 | 0.065 | 83.333 | 2 | test |
| 0.016 | 113.510 | 124.505 | 112.009 | -0.014 | 14.590 | 1.413 | 317.047 | 0.331 | 0.295 | 111.534 | 74.161 | 112.359 | 0.047 | 88.990 | 3 | test |
| 0.015 | 100.609 | 113.273 | 98.949 | 0.001 | 17.273 | 1.726 | 280.525 | 0.322 | 0.269 | 98.529 | 65.277 | 99.313 | 0.068 | 158.212 | 3 | test |
| 0.015 | 100.546 | 114.328 | 96.184 | -0.054 | 28.088 | 0.898 | 272.754 | 0.320 | 0.313 | 94.584 | 58.439 | 97.108 | 0.124 | 139.836 | 3 | test |
| 0.015 | 118.663 | 129.406 | 117.702 | 0.003 | 12.434 | 1.441 | 332.940 | 0.316 | 0.305 | 117.382 | 79.726 | 117.839 | 0.037 | 107.653 | 3 | test |
| 0.023 | 120.550 | 134.495 | 119.092 | 0.027 | 39.566 | 1.410 | 337.021 | 0.332 | 0.279 | 118.546 | 78.847 | 119.486 | 0.088 | 98.421 | 2 | test |
| 0.016 | 111.620 | 123.125 | 109.772 | 0.010 | 13.585 | 1.517 | 310.758 | 0.330 | 0.308 | 109.132 | 72.325 | 110.024 | 0.056 | 128.252 | 3 | test |
| 0.015 | 112.261 | 126.688 | 106.619 | -0.072 | 22.471 | 0.679 | 302.760 | 0.326 | 0.300 | 104.636 | 63.585 | 107.993 | 0.102 | 120.004 | 3 | test |
| 0.015 | 131.931 | 146.828 | 128.228 | -0.007 | 11.990 | 1.066 | 363.908 | 0.333 | 0.291 | 126.990 | 82.047 | 129.104 | 0.061 | 114.685 | 2 | test |
| 0.017 | 107.486 | 118.188 | 105.398 | -0.007 | 18.525 | 1.616 | 298.852 | 0.353 | 0.282 | 104.777 | 69.576 | 105.949 | 0.062 | 128.972 | 3 | test |
| 0.017 | 113.083 | 127.062 | 110.637 | -0.003 | 22.233 | 1.547 | 313.864 | 0.327 | 0.289 | 109.919 | 72.354 | 111.252 | 0.068 | 135.016 | 3 | test |
| 0.017 | 110.642 | 124.406 | 108.593 | -0.021 | 20.227 | 1.542 | 307.787 | 0.327 | 0.282 | 107.935 | 71.589 | 109.192 | 0.058 | 147.035 | 3 | test |
| 0.015 | 115.052 | 125.719 | 113.110 | 0.000 | 11.618 | 1.483 | 320.352 | 0.338 | 0.309 | 112.522 | 75.406 | 113.453 | 0.052 | 125.676 | 3 | test |
| 0.015 | 97.471 | 108.438 | 96.263 | -0.022 | 21.087 | 1.559 | 272.220 | 0.289 | 0.269 | 95.904 | 64.018 | 96.565 | 0.063 | 170.647 | 3 | test |
| 0.019 | 103.584 | 117.641 | 101.650 | -0.014 | 31.717 | 1.890 | 288.591 | 0.344 | 0.234 | 101.178 | 66.612 | 102.251 | 0.066 | 166.120 | 3 | test |
| 0.017 | 118.546 | 131.891 | 115.455 | 0.003 | 23.645 | 1.029 | 326.941 | 0.323 | 0.334 | 114.314 | 73.919 | 115.830 | 0.074 | 110.994 | 3 | test |
| 0.013 | 104.624 | 117.312 | 100.386 | -0.046 | 19.425 | 0.927 | 284.334 | 0.344 | 0.313 | 98.935 | 61.966 | 101.332 | 0.115 | 134.011 | 3 | test |

F1 original_shape_Flatness，F2 additivegaussiannoise_firstorder_RootMeanSquared,F3 binomialblurimage_firstorder_90Percentile,F4 curvatureflow_firstorder_RootMeanSquared,F5 log_firstorder_log-sigma-0-5-mm-3D-Skewness,F6 log_firstorder_log-sigma-4-0-mm-3D-Variance,F7 log_glrlm_log-sigma-1-0-mm-3D-RunVariance, F8 wavelet_firstorder_wavelet-LLL-RootMeanSquared, F9 wavelet_glcm_wavelet-LLH-Imc2, F10 wavelet_glcm_wavelet-HLL-Imc2,F11 discretegaussian_firstorder_RootMeanSquared,F12 specklenoise_firstorder_Median,F13 recursivegaussian_firstorder_RootMeanSquared,F14 shotnoise_glszm_SmallAreaLowGrayLevelEmphasis,F15 shotnoise_ngtdm_Busyness.
