# Supplementary material for: Diagnostic of fatty liver using radiomics and deep learning models on non-contrast abdominal CT
Source: PLoS One. 2025 Feb 13;20(2):e0310938. doi: 10.1371/journal.pone.0310938 (PMC11825062; doi:10.1371/journal.pone.0310938)
Supplement: S2 Table — (DOCX) [file pone.0310938.s004.docx]

**S2 Table. 3D radiomics model training and test set features**

| Label | group | A | B | C | D | E | F | G | H | I | J | K | L | M | N | O | P | Q | R | S | T |
| --- | --- | --- | --- | --- | --- | --- | --- | --- | --- | --- | --- | --- | --- | --- | --- | --- | --- | --- | --- | --- | --- |
| 0 | train | 15.119 | 14.406 | 15.111 | 0.007 | 42.189 | 78.901 | 0.555 | 9.11 | 477967.753 | 0.004 | 247.359 | 2.659 | 983.403 | 0.002 | 40.079 | 40.882 | 55.639 | 174.745 | 5.098 | 296.124 |
| 0 | test | 17.104 | 16.709 | 17.063 | 0.008 | 40.53 | 86.013 | 0.971 | 5.523 | 327492.957 | 0.008 | 234.861 | 2.582 | 996.269 | 0.004 | 42.377 | 43.286 | 58.465 | 181.123 | 5.207 | 278.042 |
| 1 | test | 14.544 | 13.764 | 14.569 | 0.007 | 41.37 | 70.847 | 0.999 | 5.514 | 318936.904 | 0.006 | 213.786 | 2.634 | 868.543 | 0.003 | 39.702 | 40.645 | 54.79 | 173.337 | 4.971 | 272.56 |
| 0 | train | 15.844 | 15.666 | 15.834 | 0.006 | 51.042 | 63.598 | 0.387 | 13.444 | 349572.474 | 0.003 | 214.276 | 2.691 | 899.884 | 0.002 | 42.793 | 43.786 | 59.185 | 181.982 | 5.037 | 277.809 |
| 0 | test | 16.677 | 16.222 | 16.645 | 0.008 | 43.3 | 80.105 | 0.981 | 5.434 | 374381.138 | 0.006 | 285.148 | 2.657 | 991.14 | 0.004 | 41.956 | 42.894 | 57.878 | 179.534 | 5.155 | 278.956 |
| 1 | train | 16.469 | 15.384 | 16.433 | 0.008 | 43.607 | 85.326 | 0.515 | 9.406 | 425721.372 | 0.004 | 257.899 | 2.69 | 728.372 | 0.003 | 39.873 | 40.618 | 55.277 | 173.96 | 4.956 | 295.361 |
| 1 | train | 14.814 | 13.531 | 14.808 | 0.007 | 49.34 | 72.262 | 0.961 | 5.505 | 304818.25 | 0.002 | 287.081 | 2.699 | 872.888 | 0.002 | 38.347 | 39.218 | 53.289 | 169.853 | 4.907 | 275.133 |
| 2 | test | 10.862 | 11.004 | 10.788 | 0.007 | 26.671 | 72.902 | 0.91 | 5.54 | 609107.385 | 0.005 | 523.399 | 2.786 | 1121.886 | 0.003 | 29.682 | 30.377 | 41.994 | 144.477 | 4.893 | 270.255 |
| 2 | train | 8.459 | 9.07 | 8.387 | 0.006 | 35.738 | 60.524 | 0.977 | 5.391 | 454948.619 | 0.004 | 440.25 | 2.781 | 802.339 | 0.002 | 25.961 | 26.678 | 36.678 | 132.248 | 4.716 | 253.202 |
| 0 | test | 17.009 | 17.642 | 16.956 | 0.007 | 40.263 | 84.733 | 1 | 5.544 | 399537.264 | 0.007 | 241.097 | 2.724 | 913.816 | 0.003 | 42.775 | 43.692 | 58.972 | 182.046 | 5.154 | 281.03 |
| 1 | test | 16.173 | 15.5 | 16.124 | 0.008 | 40.504 | 100.312 | 0.494 | 9.541 | 468835.307 | 0.01 | 386.512 | 2.535 | 1122.317 | 0.003 | 39.027 | 39.731 | 54.189 | 171.669 | 5.06 | 293.432 |
| 2 | test | 9.753 | 10.454 | 9.67 | 0.007 | 43.25 | 83.002 | 0.537 | 9.113 | 476385.027 | 0.008 | 508.279 | 2.711 | 527.786 | 0.003 | 27.234 | 27.925 | 38.753 | 136.348 | 5.087 | 263.508 |
| 1 | test | 14.704 | 14.132 | 14.703 | 0.007 | 46.416 | 74.601 | 0.993 | 5.509 | 404144.6 | 0.002 | 231.533 | 2.744 | 783.69 | 0.002 | 39.047 | 39.955 | 54.099 | 171.491 | 4.982 | 285.594 |
| 0 | train | 16.297 | 16.099 | 16.277 | 0.007 | 47.777 | 62.867 | 1.031 | 5.461 | 341469.977 | 0.003 | 218.196 | 2.661 | 945.196 | 0.003 | 42.679 | 43.589 | 58.765 | 181.58 | 5.185 | 271.105 |
| 1 | train | 15.405 | 14.511 | 15.396 | 0.007 | 41.709 | 78.781 | 0.543 | 9.131 | 479044.398 | 0.005 | 302.495 | 2.548 | 1083.124 | 0.003 | 39.073 | 39.911 | 54.144 | 171.947 | 5.018 | 279.859 |
| 0 | train | 16.874 | 15.717 | 16.827 | 0.009 | 31.519 | 86.388 | 0.528 | 9.369 | 427705.334 | 0.007 | 281.408 | 2.663 | 1098.722 | 0.004 | 41.211 | 41.944 | 57.011 | 177.512 | 5.178 | 297.656 |
| 0 | train | 23.662 | 21.494 | 23.506 | 0.011 | 35.381 | 109.857 | 0.323 | 12.179 | 403246.294 | 0.018 | 269.704 | 2.541 | 167.837 | 0.005 | 49.163 | 49.128 | 67.711 | 199.12 | 5.282 | 310.256 |
| 1 | test | 21.635 | 20.563 | 21.498 | 0.012 | 27.769 | 113.094 | 0.294 | 12.565 | 339142.534 | 0.019 | 245.805 | 2.451 | 105.379 | 0.006 | 45.986 | 45.713 | 63.674 | 190.496 | 5.172 | 315.478 |
| 0 | test | 16.334 | 15.865 | 16.304 | 0.007 | 42.759 | 71.673 | 1.058 | 5.45 | 305098.397 | 0.003 | 202.301 | 2.721 | 884.922 | 0.003 | 42.9 | 43.951 | 58.984 | 182.422 | 5.273 | 265.441 |
| 1 | train | 15.074 | 13.741 | 15.06 | 0.008 | 42.033 | 73.23 | 0.925 | 5.546 | 343805.92 | 0.006 | 331.277 | 2.693 | 970.438 | 0.003 | 38.364 | 39.127 | 53.254 | 169.999 | 4.877 | 279.794 |
| 0 | train | 16.721 | 16.273 | 16.69 | 0.008 | 34.425 | 78.895 | 0.556 | 9.153 | 349641.586 | 0.005 | 237.685 | 2.636 | 910.375 | 0.003 | 42.2 | 43.038 | 58.381 | 180.396 | 5.31 | 287.095 |
| 0 | test | 18.129 | 18.211 | 18.054 | 0.008 | 35.716 | 86.053 | 0.552 | 9.204 | 319633.702 | 0.008 | 201.547 | 2.641 | 827.403 | 0.004 | 44.076 | 45.053 | 60.713 | 185.629 | 5.19 | 284.767 |
| 1 | test | 17.385 | 19.495 | 17.272 | 0.013 | 31.78 | 101.84 | 0.187 | 18.538 | 151461.316 | 0.023 | 182.234 | 2.607 | 206.933 | 0.006 | 38.766 | 38.346 | 54.193 | 168.347 | 5.042 | 309.574 |
| 2 | train | 18.183 | 20.748 | 18.059 | 0.014 | 35.605 | 107.004 | 0.153 | 20.046 | 170482.787 | 0.028 | 311.006 | 2.555 | 68.899 | 0.007 | 40.083 | 39.412 | 56.34 | 171.695 | 5.173 | 313.02 |
| 0 | train | 17.279 | 17.818 | 17.228 | 0.007 | 41.194 | 81.427 | 1.205 | 4.957 | 650692.779 | 0.004 | 259.177 | 2.674 | 1238.628 | 0.004 | 43.834 | 44.732 | 60.497 | 185.425 | 5.28 | 285.044 |
| 0 | test | 15.115 | 14.846 | 15.116 | 0.007 | 54.048 | 74.713 | 0.996 | 5.438 | 331939.36 | 0.004 | 232.478 | 2.567 | 946.793 | 0.003 | 40.15 | 41.006 | 55.592 | 174.573 | 5.065 | 273.85 |
| 0 | train | 17.062 | 15.969 | 16.996 | 0.008 | 34.745 | 87.115 | 0.518 | 9.459 | 414971.168 | 0.008 | 267.681 | 2.504 | 979.769 | 0.003 | 40.979 | 41.633 | 56.861 | 177.082 | 5.194 | 299.981 |
| 0 | test | 15.402 | 14.122 | 15.403 | 0.007 | 37.236 | 77.13 | 1.013 | 5.415 | 509776.595 | 0.006 | 290.86 | 2.606 | 1112.558 | 0.002 | 40.386 | 41.275 | 55.929 | 175.683 | 5.007 | 263.704 |
| 1 | train | 14.021 | 13.663 | 14.046 | 0.007 | 44.858 | 66.236 | 0.584 | 9.014 | 302994.414 | 0.004 | 199.655 | 2.577 | 927.735 | 0.002 | 38.828 | 39.674 | 53.86 | 170.923 | 4.95 | 299.529 |
| 0 | test | 14.813 | 14.127 | 14.819 | 0.007 | 34.834 | 79.311 | 0.943 | 5.483 | 576522.969 | 0.005 | 390.292 | 2.522 | 1302.096 | 0.002 | 38.848 | 39.758 | 53.816 | 171.676 | 4.87 | 268.451 |
| 1 | train | 13.781 | 13.672 | 13.766 | 0.007 | 48.687 | 80.415 | 0.541 | 9.135 | 443897.835 | 0.003 | 365.852 | 2.689 | 935.017 | 0.003 | 36.002 | 36.771 | 50.207 | 163.073 | 4.816 | 271.083 |
| 0 | train | 16.799 | 15.293 | 16.754 | 0.01 | 41.849 | 87.394 | 0.523 | 9.246 | 244968.356 | 0.008 | 232.344 | 2.647 | 724.144 | 0.004 | 41.418 | 42.091 | 57.323 | 177.755 | 5.25 | 285.579 |
| 0 | train | 16.52 | 15.153 | 16.487 | 0.008 | 45.087 | 81.837 | 0.915 | 5.579 | 350891.117 | 0.007 | 301.146 | 2.616 | 951.622 | 0.003 | 40.553 | 41.306 | 56.109 | 175.162 | 5.004 | 281.347 |
| 0 | train | 16.131 | 15.424 | 16.096 | 0.007 | 32.659 | 81.498 | 0.536 | 9.285 | 523787.404 | 0.006 | 237.34 | 2.763 | 794.744 | 0.003 | 39.856 | 40.621 | 55.324 | 174.535 | 5.047 | 289.999 |
| 0 | train | 15.304 | 14.582 | 15.305 | 0.007 | 38.295 | 70.023 | 1.125 | 5.125 | 527515.709 | 0.005 | 315.628 | 2.516 | 1165.739 | 0.003 | 40.57 | 41.496 | 56.1 | 176.051 | 5.156 | 270.571 |
| 0 | train | 16.185 | 15.253 | 16.166 | 0.008 | 45.871 | 70.875 | 1.023 | 5.392 | 270810.083 | 0.005 | 180.576 | 2.589 | 872.323 | 0.003 | 42.581 | 43.557 | 58.747 | 181.598 | 5.189 | 310.434 |
| 0 | train | 15.56 | 14.163 | 15.543 | 0.007 | 52.2 | 78.14 | 0.99 | 5.426 | 325910.917 | 0.005 | 231.73 | 2.517 | 861.637 | 0.003 | 40.917 | 41.729 | 56.751 | 176.258 | 5.044 | 291.552 |
| 0 | train | 15.624 | 15.458 | 15.619 | 0.007 | 53.143 | 71.177 | 0.973 | 5.465 | 292083.382 | 0.003 | 247.263 | 2.531 | 869.45 | 0.003 | 40.883 | 41.721 | 56.436 | 176.333 | 4.932 | 277.339 |
| 0 | test | 17.573 | 17.383 | 17.516 | 0.008 | 37.449 | 78.398 | 0.551 | 9.15 | 372547.455 | 0.006 | 230.358 | 2.499 | 1034.892 | 0.004 | 43.169 | 43.92 | 59.641 | 183.129 | 5.295 | 290.036 |
| 0 | train | 16.377 | 16.669 | 16.348 | 0.007 | 45.411 | 66.632 | 0.58 | 9.001 | 344792.11 | 0.003 | 174.653 | 2.537 | 1034.172 | 0.003 | 42.373 | 43.322 | 58.65 | 181.101 | 5.139 | 297.596 |
| 0 | train | 16.16 | 15.484 | 16.133 | 0.007 | 44.333 | 88.362 | 0.559 | 9.023 | 461922.184 | 0.003 | 340.24 | 2.634 | 1067.809 | 0.003 | 41.291 | 42.144 | 57.182 | 177.844 | 5.054 | 268.764 |
| 2 | train | 9.525 | 10.338 | 9.442 | 0.007 | 38.047 | 73.771 | 0.961 | 5.365 | 753514.253 | 0.008 | 753.469 | 2.822 | 397.71 | 0.003 | 26.742 | 27.364 | 37.945 | 135.031 | 4.909 | 285.543 |
| 1 | test | 14.584 | 14.343 | 14.602 | 0.006 | 47.845 | 62.947 | 1.054 | 5.439 | 433177.723 | 0.003 | 287.839 | 2.664 | 948.279 | 0.002 | 40.936 | 41.963 | 56.591 | 176.375 | 5.004 | 260.987 |
| 0 | train | 16.327 | 15.734 | 16.306 | 0.008 | 46.849 | 81.194 | 0.948 | 5.49 | 250254.434 | 0.006 | 216.967 | 2.545 | 846.381 | 0.003 | 41.537 | 42.274 | 57.477 | 178.164 | 5.141 | 304.474 |
| 1 | train | 11.581 | 11.458 | 11.52 | 0.008 | 46.615 | 74.252 | 0.556 | 8.994 | 412605.799 | 0.005 | 372.891 | 2.716 | 1049.774 | 0.004 | 32.649 | 33.413 | 45.593 | 152.712 | 5.044 | 258.477 |
| 1 | train | 14.109 | 13.71 | 14.052 | 0.008 | 43.101 | 80.665 | 0.494 | 9.537 | 392521.448 | 0.011 | 320.665 | 2.527 | 762.225 | 0.004 | 34.891 | 35.443 | 48.903 | 159.709 | 4.901 | 301.046 |
| 1 | train | 14.939 | 14.339 | 14.942 | 0.006 | 49.232 | 65.712 | 1.029 | 5.43 | 362906.361 | 0.004 | 236.928 | 2.666 | 873.599 | 0.002 | 41.451 | 42.436 | 57.32 | 178.482 | 4.839 | 283.575 |
| 1 | test | 16.192 | 15.402 | 16.167 | 0.007 | 45.987 | 86.536 | 0.549 | 9.13 | 424065.215 | 0.005 | 311.359 | 2.65 | 974.124 | 0.003 | 40.563 | 41.408 | 56.196 | 175.862 | 4.939 | 282.033 |
| 0 | train | 17.584 | 17.087 | 17.52 | 0.008 | 47.339 | 82.894 | 0.556 | 9.183 | 315552.879 | 0.007 | 177.166 | 2.644 | 899.661 | 0.004 | 43.557 | 44.302 | 60.126 | 184.266 | 5.296 | 287.07 |
| 1 | train | 14.878 | 14.405 | 14.881 | 0.007 | 34.404 | 74.346 | 0.993 | 5.461 | 547215.424 | 0.003 | 293.006 | 2.765 | 1215.547 | 0.003 | 39.622 | 40.51 | 54.94 | 173.545 | 5.113 | 285.108 |
| 1 | train | 13.84 | 13.279 | 13.815 | 0.007 | 41.77 | 82.588 | 0.551 | 9.145 | 430421.241 | 0.006 | 272.983 | 2.638 | 964.009 | 0.003 | 36.087 | 36.824 | 50.31 | 163.448 | 5.045 | 299.747 |
| 0 | train | 17.223 | 16.414 | 17.177 | 0.008 | 46.164 | 79.218 | 0.552 | 9.173 | 336498.764 | 0.006 | 232.909 | 2.6 | 881.235 | 0.004 | 41.99 | 42.851 | 57.92 | 179.38 | 5.244 | 290.587 |
| 1 | train | 15.123 | 15.162 | 15.121 | 0.007 | 41.061 | 65.041 | 1.099 | 5.313 | 416929.34 | 0.004 | 250.186 | 2.682 | 925.863 | 0.003 | 40.374 | 41.283 | 55.967 | 175.081 | 5.13 | 262.438 |
| 0 | train | 15.486 | 14.882 | 15.488 | 0.007 | 37.411 | 68.317 | 0.607 | 8.838 | 413137.451 | 0.004 | 192.464 | 2.75 | 1004.783 | 0.003 | 40.902 | 41.841 | 56.627 | 176.771 | 5.112 | 284.808 |
| 0 | train | 15.301 | 15.818 | 15.29 | 0.006 | 39.156 | 68.584 | 1.137 | 5.198 | 454425.66 | 0.004 | 257.949 | 2.594 | 1018.067 | 0.002 | 41.49 | 42.443 | 57.394 | 178.594 | 5.184 | 273.531 |
| 1 | test | 14.883 | 13.896 | 14.888 | 0.006 | 38.569 | 65.483 | 1.01 | 5.412 | 335870.595 | 0.005 | 181.092 | 2.651 | 877.451 | 0.002 | 40.24 | 41.224 | 55.881 | 174.749 | 4.801 | 286.436 |
| 1 | test | 15.554 | 14.643 | 15.536 | 0.008 | 40.182 | 82.384 | 0.961 | 5.581 | 358766.565 | 0.005 | 223.309 | 2.637 | 794.041 | 0.003 | 39.884 | 40.665 | 55.168 | 173.694 | 5.112 | 277.121 |
| 1 | train | 13.487 | 12.923 | 13.485 | 0.007 | 42.951 | 67.859 | 0.99 | 5.503 | 371635.119 | 0.006 | 272.045 | 2.647 | 916.057 | 0.003 | 37.473 | 38.301 | 52.194 | 166.845 | 5.119 | 267.759 |
| 1 | test | 13.785 | 13.01 | 13.802 | 0.008 | 37.456 | 73.764 | 0.971 | 5.494 | 355505.754 | 0.003 | 247.904 | 2.637 | 939.035 | 0.003 | 37.635 | 38.496 | 52.25 | 167.42 | 4.898 | 264.347 |
| 1 | train | 15.785 | 15.063 | 15.781 | 0.006 | 48.176 | 61.758 | 1.044 | 5.458 | 354548.174 | 0.004 | 238.973 | 2.715 | 959.27 | 0.003 | 41.863 | 42.954 | 57.637 | 179.22 | 5.132 | 278.471 |
| 0 | test | 14.31 | 13.439 | 14.313 | 0.008 | 44.728 | 76.807 | 0.998 | 5.485 | 260261.502 | 0.005 | 223.945 | 2.626 | 754.599 | 0.003 | 38.59 | 39.335 | 53.569 | 169.5 | 5.058 | 269.706 |
| 1 | train | 14.711 | 13.746 | 14.675 | 0.009 | 42.419 | 79.561 | 0.349 | 14.037 | 354524.651 | 0.007 | 253.22 | 2.901 | 901.273 | 0.003 | 37.175 | 37.772 | 51.781 | 165.328 | 5.034 | 296.179 |
| 0 | train | 17.392 | 15.22 | 17.322 | 0.01 | 34.861 | 93.344 | 0.498 | 9.607 | 312398.475 | 0.007 | 239.642 | 2.605 | 858.425 | 0.004 | 41.482 | 42.043 | 57.539 | 177.813 | 5.266 | 291.571 |
| 1 | test | 12.527 | 12.679 | 12.535 | 0.007 | 48.619 | 69.169 | 0.979 | 5.46 | 326265.672 | 0.003 | 221.304 | 2.751 | 815.809 | 0.003 | 35.895 | 36.82 | 50.018 | 162.492 | 5.122 | 271.167 |
| 1 | train | 14.566 | 13.933 | 14.579 | 0.007 | 34.89 | 70.724 | 0.579 | 9.066 | 345589.604 | 0.005 | 189.388 | 2.607 | 883.572 | 0.003 | 39.299 | 40.121 | 54.485 | 171.785 | 4.968 | 278.762 |
| 0 | train | 14.351 | 13.586 | 14.367 | 0.007 | 43.924 | 76.203 | 0.951 | 5.462 | 474616.38 | 0.004 | 362.376 | 2.427 | 1193.362 | 0.002 | 38.591 | 39.47 | 53.499 | 170.382 | 4.707 | 270.988 |
| 1 | train | 14.755 | 13.316 | 14.755 | 0.007 | 35.08 | 71.295 | 0.379 | 13.562 | 305988.356 | 0.004 | 228.821 | 2.577 | 877.731 | 0.003 | 38.301 | 39.056 | 53.032 | 169.138 | 5.027 | 288.014 |
| 0 | test | 16.327 | 15.47 | 16.288 | 0.007 | 37.738 | 79.34 | 0.547 | 9.246 | 373934.046 | 0.005 | 241.715 | 2.583 | 751.923 | 0.003 | 40.564 | 41.377 | 56.17 | 176.019 | 5.276 | 282.736 |
| 0 | train | 16.4 | 15.674 | 16.368 | 0.008 | 56.633 | 84.877 | 0.55 | 9.064 | 389609.819 | 0.005 | 281.831 | 2.57 | 962.822 | 0.003 | 41.513 | 42.324 | 57.477 | 178.323 | 5.102 | 277.627 |
| 1 | train | 13.363 | 13.408 | 13.315 | 0.008 | 37.714 | 79.785 | 0.51 | 9.362 | 584976.299 | 0.007 | 482.464 | 2.733 | 1048.924 | 0.004 | 34.226 | 34.897 | 47.889 | 158.001 | 4.973 | 272.55 |
| 0 | train | 14.99 | 15.284 | 14.989 | 0.007 | 40.615 | 68.722 | 1.01 | 5.477 | 375416.404 | 0.004 | 219.113 | 2.626 | 974.491 | 0.002 | 40.764 | 41.683 | 56.389 | 176.768 | 5.05 | 280.227 |
| 0 | train | 14.383 | 13.325 | 14.4 | 0.007 | 40.165 | 69.945 | 1.027 | 5.485 | 316655.678 | 0.004 | 259.831 | 2.521 | 751.613 | 0.003 | 39.813 | 40.651 | 55.119 | 172.85 | 5.024 | 269.713 |
| 1 | test | 14.148 | 13.252 | 14.173 | 0.006 | 44.337 | 69.667 | 1.029 | 5.48 | 377475.638 | 0.005 | 221.295 | 2.783 | 957.801 | 0.002 | 39.592 | 40.619 | 54.688 | 173.484 | 4.895 | 274.159 |
| 0 | test | 21.395 | 19.935 | 21.261 | 0.011 | 31.038 | 108.908 | 0.354 | 11.648 | 283096.969 | 0.014 | 174.301 | 2.526 | 174.427 | 0.006 | 45.565 | 45.658 | 63.146 | 189.326 | 5.277 | 307.802 |
| 1 | train | 15.859 | 15.086 | 15.845 | 0.007 | 46.687 | 74.751 | 0.957 | 5.5 | 434854.601 | 0.004 | 309.695 | 2.599 | 1013.895 | 0.003 | 40.144 | 41.048 | 55.637 | 175.069 | 5.004 | 284.705 |
| 0 | test | 16.407 | 15.977 | 16.385 | 0.008 | 50.657 | 71.629 | 0.58 | 9.022 | 258285.073 | 0.003 | 189.821 | 2.628 | 750.689 | 0.003 | 42.034 | 42.952 | 57.877 | 179.418 | 5.22 | 279.737 |
| 0 | test | 15.543 | 15.134 | 15.546 | 0.007 | 52.232 | 60.975 | 0.595 | 8.948 | 347044.294 | 0.004 | 205.442 | 2.678 | 900.575 | 0.002 | 41.994 | 42.974 | 57.957 | 179.556 | 4.923 | 286.174 |
| 0 | test | 15.977 | 15.829 | 15.972 | 0.007 | 38.032 | 72.492 | 0.997 | 5.403 | 363940.535 | 0.006 | 250.799 | 2.514 | 959.906 | 0.003 | 41.607 | 42.473 | 57.519 | 178.922 | 4.942 | 287.445 |
| 1 | train | 14.725 | 13.496 | 14.723 | 0.007 | 45.798 | 72.647 | 0.984 | 5.438 | 434007.603 | 0.005 | 288.251 | 2.737 | 1052.448 | 0.002 | 39.018 | 39.865 | 54.229 | 171.76 | 5.008 | 308.154 |
| 2 | train | 9.06 | 9.191 | 8.979 | 0.007 | 38.503 | 65.396 | 0.556 | 8.976 | 385112.591 | 0.002 | 427.59 | 2.586 | 812.959 | 0.002 | 27.221 | 27.94 | 38.422 | 135.929 | 4.727 | 238.424 |
| 2 | test | 10.865 | 11.309 | 10.795 | 0.007 | 34.224 | 66.986 | 0.554 | 9.028 | 481972.262 | 0.004 | 368.598 | 2.805 | 799.748 | 0.003 | 30.49 | 31.279 | 42.823 | 146.184 | 5.034 | 256.006 |
| 0 | test | 16.377 | 16.489 | 16.348 | 0.007 | 43.098 | 75.885 | 0.579 | 9.13 | 455914.938 | 0.005 | 255.144 | 2.799 | 952.775 | 0.003 | 41.359 | 42.303 | 57.186 | 177.884 | 5.127 | 286.092 |
| 1 | train | 14.407 | 13.806 | 14.376 | 0.008 | 43.185 | 82.762 | 0.885 | 5.722 | 498635.138 | 0.011 | 400.037 | 2.705 | 1053.236 | 0.003 | 36.386 | 37.028 | 50.601 | 164.287 | 4.814 | 287.536 |
| 0 | test | 17.128 | 17.159 | 17.079 | 0.007 | 47.045 | 76.666 | 0.994 | 5.522 | 391473.953 | 0.005 | 204.73 | 2.743 | 1010.459 | 0.003 | 43.308 | 44.23 | 59.785 | 183.723 | 5.249 | 284.714 |
| 1 | test | 13.427 | 13.226 | 13.433 | 0.007 | 40.18 | 68.34 | 0.562 | 9.037 | 449841.567 | 0.003 | 293.695 | 2.797 | 1078.554 | 0.002 | 36.789 | 37.68 | 51.156 | 165.415 | 4.787 | 267.39 |
| 0 | train | 15.521 | 14.567 | 15.519 | 0.006 | 40.842 | 67.712 | 0.603 | 9.015 | 350180.461 | 0.004 | 185.394 | 2.767 | 899.289 | 0.002 | 42.135 | 43.119 | 58.141 | 180 | 4.971 | 287.381 |
| 1 | train | 14.18 | 13.172 | 14.204 | 0.007 | 43.867 | 67.504 | 0.578 | 9.057 | 287778.041 | 0.005 | 190.527 | 2.615 | 892.223 | 0.002 | 39.689 | 40.485 | 55.096 | 173.033 | 4.805 | 289.076 |
| 1 | train | 15.488 | 14.898 | 15.491 | 0.007 | 46.395 | 67.722 | 0.995 | 5.514 | 359403.621 | 0.004 | 222.43 | 2.748 | 1000.11 | 0.003 | 40.629 | 41.566 | 56.21 | 176.27 | 5.119 | 285.386 |
| 2 | test | 10.447 | 10.761 | 10.357 | 0.007 | 43.643 | 68.148 | 0.955 | 5.427 | 374873.802 | 0.007 | 281.18 | 2.784 | 917.2 | 0.004 | 30.023 | 30.816 | 41.915 | 144.723 | 4.941 | 275.477 |
| 0 | train | 16.972 | 15.909 | 16.917 | 0.009 | 44.546 | 82.772 | 0.53 | 9.377 | 425814.391 | 0.007 | 320.877 | 2.631 | 1007.97 | 0.003 | 41.633 | 42.349 | 57.674 | 178.822 | 5.256 | 285.765 |
| 1 | train | 15.242 | 14.109 | 15.234 | 0.008 | 48.659 | 70.958 | 0.972 | 5.476 | 255292.156 | 0.002 | 188.611 | 2.426 | 928.134 | 0.003 | 40.12 | 40.892 | 55.595 | 174.118 | 5.026 | 275.546 |
| 0 | train | 15.573 | 14.932 | 15.543 | 0.007 | 45.793 | 88.883 | 0.942 | 5.597 | 437924.271 | 0.009 | 379.794 | 2.68 | 1158.155 | 0.003 | 38.823 | 39.558 | 53.855 | 171.681 | 5.087 | 284.634 |
| 2 | train | 9.048 | 9.317 | 8.973 | 0.007 | 41.893 | 70.484 | 0.559 | 8.981 | 432211.874 | 0.007 | 507.864 | 2.785 | 933.72 | 0.003 | 26.581 | 27.27 | 37.707 | 134.054 | 4.849 | 260.435 |
| 0 | test | 15.126 | 14.112 | 15.124 | 0.007 | 46.113 | 64.805 | 1.045 | 5.447 | 271717.542 | 0.004 | 140.476 | 2.7 | 810.268 | 0.002 | 41.541 | 42.6 | 57.422 | 179.004 | 4.992 | 294.443 |
| 1 | test | 15.235 | 14.525 | 15.195 | 0.008 | 39.931 | 82.25 | 0.516 | 9.341 | 481366.096 | 0.008 | 401.175 | 2.557 | 1162.867 | 0.003 | 37.524 | 38.259 | 52.192 | 167.38 | 4.962 | 273.608 |
| 2 | train | 7.529 | 9.005 | 7.442 | 0.006 | 33.79 | 60.695 | 0.563 | 8.884 | 467957.822 | 0.002 | 371.211 | 2.66 | 735.54 | 0.003 | 23.304 | 24.027 | 33.105 | 123.231 | 4.879 | 231.359 |
| 1 | train | 14.352 | 13.075 | 14.363 | 0.007 | 44.074 | 73.46 | 0.36 | 13.599 | 539874.185 | 0.006 | 362.166 | 2.687 | 1267.02 | 0.002 | 37.913 | 38.683 | 52.627 | 168.718 | 4.63 | 284.614 |
| 0 | train | 16.454 | 15.658 | 16.423 | 0.008 | 38.666 | 79.466 | 0.552 | 9.236 | 255237.91 | 0.006 | 151.016 | 2.546 | 720.051 | 0.004 | 41.514 | 42.294 | 57.426 | 177.838 | 5.263 | 284.923 |
| 1 | train | 15.419 | 14.853 | 15.411 | 0.007 | 41.242 | 78.077 | 0.565 | 9.052 | 440611.033 | 0.003 | 213.662 | 2.6 | 988.395 | 0.003 | 40.14 | 41.027 | 55.712 | 175.153 | 5.149 | 294.702 |
| 0 | train | 15.48 | 15.195 | 15.486 | 0.006 | 46.329 | 65.283 | 1.035 | 5.458 | 329718.287 | 0.002 | 207.591 | 2.754 | 823.662 | 0.002 | 41.638 | 42.55 | 57.573 | 179.311 | 5.028 | 277.179 |
| 2 | train | 11.01 | 11.237 | 10.941 | 0.008 | 37.035 | 70.11 | 0.54 | 9.163 | 600326.046 | 0.005 | 379.263 | 2.754 | 1035.555 | 0.004 | 30.456 | 31.143 | 42.862 | 146.526 | 5.092 | 277.829 |
| 0 | train | 16.198 | 15.158 | 16.169 | 0.008 | 37.109 | 75.65 | 0.557 | 9.081 | 339351.509 | 0.007 | 215.032 | 2.604 | 911.906 | 0.003 | 40.55 | 41.429 | 56.064 | 175.934 | 5.123 | 277.746 |
| 0 | test | 16.066 | 15.54 | 16.048 | 0.007 | 34.067 | 73.933 | 0.601 | 8.802 | 467962.604 | 0.005 | 235.381 | 2.745 | 1068.66 | 0.003 | 41.313 | 42.343 | 57.044 | 178.107 | 5.069 | 271.795 |
| 1 | test | 14.568 | 13.747 | 14.565 | 0.007 | 48.853 | 82.827 | 0.951 | 5.53 | 369486.036 | 0.003 | 318.184 | 2.595 | 1003.004 | 0.003 | 38.316 | 39.18 | 53.12 | 169.664 | 4.893 | 273.359 |
| 1 | train | 14.787 | 13.789 | 14.772 | 0.007 | 40.699 | 76.253 | 0.392 | 13.048 | 515564.485 | 0.006 | 333.965 | 2.594 | 1089.885 | 0.002 | 38.121 | 38.872 | 52.953 | 169.064 | 5.007 | 296.258 |
| 0 | test | 15.561 | 15.222 | 15.555 | 0.006 | 32.251 | 75.903 | 1.035 | 5.27 | 619431.333 | 0.003 | 315.825 | 2.766 | 1277.787 | 0.002 | 41.171 | 42.13 | 57.001 | 178.418 | 4.979 | 289.438 |
| 0 | train | 16.592 | 16.178 | 16.563 | 0.008 | 44.174 | 77.803 | 0.562 | 9.088 | 394969.068 | 0.007 | 275.985 | 2.68 | 958.698 | 0.003 | 41.744 | 42.638 | 57.699 | 179.049 | 5.268 | 282.475 |
| 0 | test | 15.422 | 14.407 | 15.421 | 0.007 | 39.687 | 73.767 | 1.089 | 5.342 | 363252.843 | 0.005 | 176.304 | 2.659 | 940.045 | 0.003 | 41.777 | 42.746 | 57.766 | 178.927 | 5.271 | 281.186 |
| 0 | test | 16.38 | 16.92 | 16.345 | 0.007 | 46.339 | 67.991 | 1.082 | 5.369 | 404489.249 | 0.006 | 244.861 | 2.587 | 829.035 | 0.003 | 42.308 | 43.242 | 58.444 | 180.646 | 5.186 | 270.809 |
| 1 | test | 14.204 | 13.248 | 14.213 | 0.007 | 41.695 | 70.678 | 0.971 | 5.474 | 496279.44 | 0.007 | 379.776 | 2.655 | 1217.303 | 0.002 | 37.889 | 38.78 | 52.543 | 168.819 | 4.756 | 278.955 |
| 1 | test | 15.868 | 14.742 | 15.852 | 0.007 | 37.944 | 83.667 | 0.936 | 5.564 | 354918.99 | 0.006 | 239.572 | 2.601 | 825.242 | 0.003 | 40.324 | 41.146 | 55.919 | 175.372 | 4.951 | 276.651 |
| 0 | train | 16.726 | 15.454 | 16.662 | 0.008 | 43.016 | 86.062 | 0.357 | 13.671 | 494912.871 | 0.006 | 278.63 | 2.613 | 1011.951 | 0.003 | 39.891 | 40.44 | 55.384 | 174.006 | 5.171 | 300.166 |
| 0 | train | 21.994 | 20.372 | 21.855 | 0.009 | 33.714 | 106.9 | 0.424 | 10.668 | 332868.652 | 0.01 | 197.258 | 2.511 | 569.838 | 0.004 | 47.469 | 47.848 | 65.313 | 194.678 | 5.2 | 306.66 |
| 1 | test | 13.708 | 12.88 | 13.708 | 0.007 | 36.695 | 74.387 | 0.971 | 5.445 | 394502.512 | 0.003 | 262.039 | 2.577 | 1039.4 | 0.003 | 37.05 | 37.899 | 51.505 | 166.301 | 4.952 | 282.868 |
| 1 | train | 22.256 | 20.775 | 22.113 | 0.012 | 42.626 | 113.029 | 0.2 | 18.421 | 364335.823 | 0.021 | 368.837 | 2.447 | 106.574 | 0.006 | 46.796 | 46.54 | 64.789 | 192.411 | 5.304 | 322.096 |
| 0 | train | 15.115 | 15.723 | 15.124 | 0.006 | 36.293 | 66.567 | 0.577 | 9.034 | 467316.843 | 0.004 | 273.774 | 2.592 | 1100.261 | 0.002 | 40.692 | 41.654 | 56.32 | 176.352 | 5.015 | 281.788 |
| 0 | test | 15.912 | 15.264 | 15.903 | 0.008 | 33.621 | 68.143 | 1.023 | 5.477 | 363589.355 | 0.003 | 217.608 | 2.605 | 971.627 | 0.003 | 41.979 | 42.898 | 57.879 | 179.538 | 5.233 | 276.894 |
| 0 | train | 21.957 | 20.387 | 21.814 | 0.01 | 34.093 | 105.956 | 0.352 | 11.698 | 320075.691 | 0.016 | 194.253 | 2.466 | 202.526 | 0.006 | 46.575 | 46.648 | 64.435 | 191.876 | 5.131 | 321.76 |
| 0 | train | 15.099 | 15.422 | 15.106 | 0.007 | 42.024 | 75.519 | 0.585 | 9.025 | 387823.302 | 0.002 | 210.111 | 2.644 | 1020.165 | 0.002 | 40.627 | 41.51 | 56.275 | 176.204 | 5.005 | 288.189 |
| 1 | train | 14.825 | 13.422 | 14.813 | 0.008 | 47.072 | 79.636 | 0.953 | 5.538 | 304871.203 | 0.005 | 238.294 | 2.593 | 851.568 | 0.003 | 37.93 | 38.688 | 52.636 | 167.929 | 4.808 | 277.979 |
| 1 | test | 15.122 | 13.785 | 15.106 | 0.008 | 40.418 | 70.989 | 0.55 | 9.199 | 296981.61 | 0.004 | 256.015 | 2.513 | 959.284 | 0.003 | 39.096 | 39.823 | 54.163 | 171.227 | 5.121 | 282.179 |
| 1 | train | 13.333 | 12.877 | 13.323 | 0.008 | 44.612 | 73.233 | 0.958 | 5.44 | 394499.795 | 0.009 | 351.97 | 2.637 | 890.399 | 0.003 | 35.856 | 36.607 | 49.955 | 161.914 | 4.891 | 296.394 |
| 1 | train | 14.996 | 14.221 | 15.005 | 0.007 | 41.516 | 71.965 | 0.574 | 9.049 | 393298.831 | 0.005 | 216.905 | 2.594 | 1067.016 | 0.002 | 39.832 | 40.627 | 55.138 | 173.649 | 4.981 | 299.357 |
| 2 | train | 9.154 | 9.573 | 9.077 | 0.007 | 32.078 | 65.124 | 0.572 | 9.033 | 431376.23 | 0.006 | 315.514 | 2.525 | 999.631 | 0.003 | 27.236 | 27.967 | 38.555 | 136.258 | 4.956 | 260.478 |
| 2 | train | 10.04 | 10.002 | 9.972 | 0.007 | 43.983 | 66.652 | 0.984 | 5.38 | 503559.284 | 0.006 | 415.159 | 2.686 | 1057.771 | 0.003 | 29.315 | 30.147 | 41.284 | 142.704 | 4.929 | 260.502 |
| 0 | train | 14.443 | 13.88 | 14.461 | 0.007 | 45.562 | 82.347 | 0.596 | 8.933 | 318795.451 | 0.003 | 224.31 | 2.517 | 889.872 | 0.002 | 39.675 | 40.639 | 54.936 | 173.466 | 4.969 | 287.309 |
| 1 | test | 15.241 | 14.891 | 15.238 | 0.007 | 49.363 | 74.726 | 1.007 | 5.453 | 331599.712 | 0.004 | 250.701 | 2.638 | 902.401 | 0.003 | 40.238 | 41.144 | 55.746 | 175.017 | 4.99 | 299.539 |
| 1 | test | 14.486 | 13.21 | 14.471 | 0.007 | 52.117 | 81.585 | 0.548 | 9.128 | 360173.191 | 0.008 | 265.172 | 2.647 | 938.501 | 0.002 | 37.198 | 37.954 | 51.74 | 166.269 | 4.917 | 278.409 |
| 1 | train | 13.638 | 13.012 | 13.653 | 0.007 | 43.329 | 66.162 | 0.997 | 5.476 | 283029.749 | 0.004 | 180.653 | 2.698 | 825.507 | 0.003 | 38.329 | 39.201 | 53.248 | 169.539 | 5.091 | 279.517 |
| 0 | test | 15.231 | 15.216 | 15.231 | 0.007 | 37.518 | 69.853 | 0.587 | 9.05 | 390356.263 | 0.006 | 219.888 | 2.623 | 1003.906 | 0.003 | 40.306 | 41.267 | 55.823 | 175.448 | 5.116 | 274.599 |
| 1 | test | 16.795 | 15.525 | 16.737 | 0.008 | 46.881 | 92.646 | 0.528 | 9.359 | 328759.293 | 0.012 | 235.096 | 2.587 | 875.282 | 0.003 | 41.086 | 41.823 | 57.017 | 176.839 | 5.307 | 282.498 |
| 0 | test | 16.223 | 15.852 | 16.206 | 0.008 | 42.041 | 76.38 | 1 | 5.419 | 334605.875 | 0.006 | 268.371 | 2.561 | 968.379 | 0.003 | 42.015 | 42.885 | 58.052 | 179.901 | 5.164 | 275.756 |
| 1 | test | 14.01 | 12.974 | 14.035 | 0.007 | 41.223 | 66.077 | 1.041 | 5.471 | 271697.243 | 0.002 | 169.82 | 2.806 | 770.754 | 0.002 | 39.243 | 40.171 | 54.402 | 171.353 | 4.803 | 267.831 |
| 0 | test | 16.89 | 17.272 | 16.862 | 0.007 | 37.555 | 66.964 | 0.383 | 13.516 | 334844.511 | 0.003 | 188.793 | 2.547 | 948.522 | 0.003 | 43.818 | 44.78 | 60.387 | 184.946 | 5.184 | 284.468 |
| 1 | train | 15.387 | 14.576 | 15.348 | 0.008 | 42.058 | 79.026 | 0.515 | 9.384 | 416866.843 | 0.007 | 273.404 | 2.566 | 835.924 | 0.003 | 38.645 | 39.328 | 53.93 | 170.792 | 4.976 | 294.129 |
| 0 | train | 14.107 | 13.985 | 14.11 | 0.007 | 42.855 | 76.901 | 0.984 | 5.444 | 466213.306 | 0.003 | 409.091 | 2.651 | 1040.697 | 0.002 | 38.134 | 39.02 | 52.926 | 169.167 | 4.904 | 289.677 |
| 0 | train | 17.157 | 18.172 | 17.101 | 0.007 | 44.633 | 76.587 | 0.601 | 9.055 | 390215.143 | 0.003 | 191.807 | 2.761 | 959.206 | 0.003 | 44.389 | 45.395 | 61.254 | 186.838 | 5.169 | 289.909 |
| 0 | train | 16.344 | 15.492 | 16.312 | 0.008 | 45.261 | 80.321 | 0.961 | 5.529 | 327266.041 | 0.004 | 255.816 | 2.538 | 879.698 | 0.003 | 41.367 | 42.147 | 57.165 | 177.45 | 5.189 | 277.07 |
| 0 | train | 15.891 | 15.039 | 15.887 | 0.007 | 40.867 | 73.979 | 1.007 | 5.492 | 386276.438 | 0.006 | 221.561 | 2.679 | 1005.933 | 0.003 | 42.003 | 42.942 | 58.034 | 180.054 | 5.152 | 267.622 |
| 1 | train | 14.678 | 14.092 | 14.684 | 0.007 | 44.392 | 75.538 | 0.385 | 13.397 | 320186.039 | 0.006 | 178.787 | 2.678 | 872.88 | 0.003 | 39.583 | 40.511 | 54.886 | 173.167 | 5.059 | 281.896 |
| 1 | test | 12.556 | 12.896 | 12.547 | 0.007 | 42.07 | 66.475 | 0.976 | 5.468 | 401513.541 | 0.002 | 293.073 | 2.754 | 1114.896 | 0.003 | 34.825 | 35.704 | 48.478 | 159.579 | 4.822 | 283.798 |
| 1 | train | 15.238 | 14.643 | 15.24 | 0.006 | 52.529 | 69.69 | 0.593 | 8.991 | 360973.161 | 0.006 | 223.047 | 2.732 | 940.935 | 0.002 | 40.998 | 41.953 | 56.795 | 177.186 | 5.088 | 291.835 |
| 0 | train | 22.315 | 20.49 | 22.171 | 0.011 | 37.903 | 107.41 | 0.35 | 11.671 | 211256.329 | 0.014 | 160.947 | 2.488 | 276.602 | 0.005 | 47.441 | 47.475 | 65.503 | 193.996 | 5.198 | 313.892 |
| 1 | train | 14.959 | 13.965 | 14.936 | 0.007 | 39.67 | 81.644 | 0.915 | 5.631 | 335471.149 | 0.007 | 270.103 | 2.707 | 761.294 | 0.002 | 37.832 | 38.531 | 52.601 | 167.985 | 4.846 | 277.855 |
| 1 | train | 18.22 | 18.709 | 18.111 | 0.012 | 33.403 | 99.721 | 0.328 | 11.903 | 251633.198 | 0.021 | 215.44 | 2.424 | 243.176 | 0.006 | 40.165 | 40.09 | 55.987 | 174.56 | 5.124 | 314.451 |
| 0 | train | 19.26 | 17.686 | 19.154 | 0.009 | 36.834 | 100.27 | 0.447 | 10.302 | 345714.121 | 0.011 | 244.011 | 2.677 | 336.27 | 0.005 | 43.411 | 43.828 | 60.073 | 183.888 | 4.974 | 310.08 |
| 0 | test | 22.001 | 20.36 | 21.859 | 0.011 | 39.72 | 111.463 | 0.327 | 11.985 | 275767.912 | 0.019 | 176.157 | 2.402 | 267.744 | 0.005 | 46.47 | 46.554 | 64.285 | 191.862 | 5.317 | 314.971 |
| 0 | test | 22.253 | 20.872 | 22.112 | 0.011 | 30.527 | 112.009 | 0.198 | 18.393 | 318409.637 | 0.02 | 226.963 | 2.453 | 204.657 | 0.006 | 46.663 | 46.478 | 64.475 | 192.181 | 5.112 | 316.056 |
| 0 | test | 22.59 | 20.455 | 22.444 | 0.012 | 39.548 | 114.706 | 0.301 | 12.49 | 314259.45 | 0.021 | 221.606 | 2.442 | 165.225 | 0.006 | 47.235 | 46.978 | 65.322 | 193.316 | 5.279 | 317.537 |
| 0 | train | 20.759 | 20.341 | 20.627 | 0.012 | 33.722 | 111.625 | 0.286 | 12.691 | 292036.647 | 0.02 | 273.126 | 2.478 | 141.808 | 0.006 | 44.319 | 43.969 | 61.539 | 185.725 | 5.085 | 319.658 |
| 1 | train | 19.714 | 19.105 | 19.592 | 0.012 | 37.072 | 101.113 | 0.139 | 21.082 | 102193.566 | 0.017 | 142.282 | 2.67 | 110.268 | 0.006 | 42.553 | 42.568 | 59.08 | 178.965 | 5.128 | 320.744 |
| 0 | test | 22.561 | 20.613 | 22.417 | 0.011 | 24.327 | 110.755 | 0.34 | 11.798 | 314424.163 | 0.017 | 173.059 | 2.513 | 307.082 | 0.006 | 47.316 | 47.354 | 65.148 | 193.881 | 5.222 | 309.712 |
| 0 | train | 14.835 | 13.873 | 14.849 | 0.007 | 38.552 | 70.931 | 0.591 | 8.995 | 355497.324 | 0.004 | 267.614 | 2.684 | 896.256 | 0.003 | 40.653 | 41.507 | 56.314 | 175.655 | 5.092 | 284.139 |
| 1 | train | 13.33 | 13.207 | 13.303 | 0.008 | 43.649 | 85.38 | 0.546 | 9.139 | 450398.026 | 0.011 | 363.332 | 2.653 | 1120.226 | 0.003 | 34.951 | 35.672 | 48.728 | 159.767 | 4.922 | 285.899 |
| 2 | train | 10.573 | 10.663 | 10.487 | 0.006 | 36.946 | 61.982 | 0.972 | 5.406 | 480921.859 | 0.005 | 356.586 | 2.531 | 1015.565 | 0.003 | 30.739 | 31.6 | 43.075 | 147.374 | 4.849 | 251.225 |
| 0 | train | 16.42 | 16.092 | 16.398 | 0.008 | 38.497 | 84.831 | 0.968 | 5.534 | 318871.486 | 0.008 | 197.806 | 2.724 | 884.766 | 0.003 | 41.364 | 42.207 | 57.262 | 178.094 | 5.293 | 285.517 |
| 1 | train | 13.67 | 13.763 | 13.696 | 0.007 | 40.826 | 67.686 | 1.005 | 5.467 | 399023.182 | 0.003 | 256.941 | 2.736 | 979.401 | 0.002 | 38.482 | 39.311 | 53.409 | 169.882 | 4.844 | 289.315 |
| 1 | train | 13.801 | 13.248 | 13.82 | 0.007 | 34.818 | 76.12 | 0.575 | 8.961 | 443010.063 | 0.004 | 266.212 | 2.614 | 1029.739 | 0.002 | 37.873 | 38.711 | 52.568 | 168.428 | 4.782 | 269.749 |
| 0 | train | 15.933 | 14.939 | 15.915 | 0.008 | 41.012 | 72.926 | 0.987 | 5.495 | 358676.202 | 0.006 | 231.254 | 2.631 | 964.129 | 0.003 | 40.855 | 41.68 | 56.407 | 176.5 | 5.234 | 289.071 |
| 1 | test | 14.227 | 13.096 | 14.245 | 0.007 | 42.729 | 70.51 | 0.985 | 5.448 | 346591.143 | 0.006 | 305.776 | 2.569 | 927.893 | 0.003 | 38.368 | 39.187 | 53.173 | 169.096 | 4.771 | 262.961 |
| 1 | train | 13.316 | 14.218 | 13.358 | 0.006 | 41.706 | 60.223 | 1.019 | 5.443 | 399789.665 | 0.003 | 212.173 | 2.758 | 971.722 | 0.002 | 38.38 | 39.307 | 53.299 | 169.731 | 4.736 | 269.413 |
| 1 | train | 13.135 | 13.148 | 13.073 | 0.008 | 41.829 | 81.081 | 0.517 | 9.246 | 573319.546 | 0.006 | 449.959 | 2.702 | 959.045 | 0.004 | 33.694 | 34.418 | 47.38 | 156.879 | 5.184 | 286.843 |
| 1 | test | 14.44 | 13.776 | 14.446 | 0.007 | 35.707 | 76.461 | 0.961 | 5.495 | 375069.517 | 0.004 | 242.165 | 2.797 | 975.03 | 0.002 | 38.089 | 38.895 | 52.906 | 168.453 | 4.779 | 275.466 |
| 1 | train | 12.752 | 12.761 | 12.74 | 0.007 | 46.706 | 67.342 | 0.968 | 5.441 | 356540.86 | 0.004 | 267.501 | 2.624 | 1005.498 | 0.003 | 35.329 | 36.181 | 49.303 | 161.417 | 4.927 | 257.391 |
| 1 | test | 13.492 | 13.584 | 13.446 | 0.008 | 23.052 | 85.256 | 0.894 | 5.617 | 520403.177 | 0.008 | 429.531 | 2.668 | 977.771 | 0.004 | 34.671 | 35.342 | 48.469 | 159.403 | 4.992 | 305.382 |
| 1 | train | 13.047 | 13.285 | 13.046 | 0.007 | 47.5 | 67.554 | 0.574 | 9.003 | 393512.939 | 0.004 | 298.199 | 2.684 | 1035.12 | 0.003 | 35.949 | 36.897 | 50.072 | 162.948 | 5.051 | 289.799 |
| 0 | train | 15.533 | 14.944 | 15.514 | 0.007 | 47.21 | 78.975 | 0.965 | 5.537 | 350943.112 | 0.005 | 293.451 | 2.718 | 808.959 | 0.003 | 39.429 | 40.177 | 54.64 | 171.907 | 5.053 | 286.057 |
| 0 | train | 16.467 | 15.595 | 16.433 | 0.008 | 44.264 | 77.356 | 0.964 | 5.571 | 331113.851 | 0.009 | 288.716 | 2.721 | 814.918 | 0.003 | 40.711 | 41.454 | 56.36 | 175.662 | 5.12 | 296.672 |
| 1 | train | 15.205 | 14.44 | 15.201 | 0.007 | 49.777 | 74.946 | 0.977 | 5.442 | 381299.918 | 0.003 | 348.157 | 2.467 | 1046.633 | 0.002 | 39.519 | 40.407 | 54.71 | 173.179 | 4.915 | 271.286 |
| 1 | test | 16.13 | 16.281 | 16.106 | 0.006 | 40.504 | 77.949 | 1.038 | 5.413 | 487044.812 | 0.004 | 292.132 | 2.602 | 1038.958 | 0.002 | 42.376 | 43.34 | 58.597 | 181.306 | 5.043 | 268.9 |
| 0 | train | 15.798 | 14.82 | 15.788 | 0.008 | 36.537 | 83.183 | 0.967 | 5.575 | 345105.282 | 0.004 | 188.936 | 2.679 | 818.169 | 0.003 | 40.579 | 41.404 | 56.142 | 175.338 | 5.221 | 297.629 |
| 0 | test | 15.69 | 14.772 | 15.685 | 0.007 | 37.084 | 70.009 | 1.032 | 5.416 | 349087.42 | 0.004 | 224.206 | 2.465 | 911.769 | 0.003 | 42.072 | 43.09 | 57.968 | 179.847 | 5.045 | 257.06 |
| 2 | train | 8.803 | 9.102 | 8.735 | 0.006 | 40.032 | 65.133 | 0.981 | 5.386 | 513214.408 | 0.005 | 397.287 | 2.889 | 1014.948 | 0.002 | 27.158 | 27.917 | 38.316 | 135.924 | 4.593 | 263.789 |
| 1 | test | 11.542 | 12.141 | 11.476 | 0.007 | 50.54 | 74.712 | 0.946 | 5.442 | 477372.501 | 0.005 | 538.792 | 2.468 | 1131.155 | 0.004 | 32.171 | 32.974 | 45.001 | 151.815 | 5.039 | 257.725 |
| 0 | test | 15.693 | 15.998 | 15.689 | 0.007 | 33.414 | 75.801 | 0.591 | 8.888 | 423782.311 | 0.003 | 201.739 | 2.582 | 1008.657 | 0.003 | 41.598 | 42.559 | 57.593 | 179.168 | 4.974 | 287 |
| 1 | train | 14.553 | 13.982 | 14.557 | 0.007 | 43.493 | 78.909 | 0.963 | 5.49 | 416982.711 | 0.006 | 276.459 | 2.618 | 975.736 | 0.002 | 38.275 | 39.084 | 53.162 | 169.429 | 4.928 | 269.469 |
| 1 | train | 13.617 | 13.562 | 13.584 | 0.008 | 45.101 | 79.521 | 0.537 | 9.204 | 480316.371 | 0.007 | 285.924 | 2.68 | 1055.027 | 0.004 | 36.045 | 36.723 | 50.462 | 163.219 | 5.167 | 279.095 |
| 0 | train | 13.042 | 12.623 | 13.07 | 0.007 | 44.15 | 67.61 | 0.601 | 9.046 | 311290.841 | 0.004 | 192.062 | 2.912 | 808.375 | 0.002 | 37.094 | 37.907 | 51.501 | 165.67 | 4.939 | 270.344 |
| 1 | test | 15.19 | 13.814 | 15.188 | 0.007 | 34.616 | 77.24 | 0.578 | 9.055 | 486767.181 | 0.006 | 257.155 | 2.66 | 1026.45 | 0.003 | 40.213 | 41.048 | 55.633 | 174.659 | 5.118 | 293.988 |
| 0 | train | 14.558 | 15.026 | 14.575 | 0.005 | 48.94 | 58.195 | 0.618 | 8.938 | 444274.758 | 0.003 | 203.53 | 2.731 | 1027.956 | 0.002 | 41.047 | 42.123 | 56.8 | 177.824 | 4.923 | 301.333 |
| 1 | train | 14.013 | 12.97 | 14.016 | 0.007 | 42.519 | 73.427 | 0.957 | 5.505 | 382120.851 | 0.002 | 290.368 | 2.553 | 1078.254 | 0.002 | 37.497 | 38.229 | 52.133 | 167.222 | 4.729 | 271.74 |
| 1 | train | 14.112 | 13.321 | 14.103 | 0.007 | 41.889 | 85.784 | 0.556 | 9.109 | 410232.601 | 0.008 | 339.073 | 2.65 | 963.631 | 0.002 | 36.901 | 37.68 | 51.275 | 165.216 | 4.796 | 280.252 |
| 2 | test | 9.301 | 9.978 | 9.216 | 0.007 | 38.974 | 68.87 | 0.919 | 5.52 | 517412.908 | 0.004 | 479.155 | 2.797 | 688.964 | 0.003 | 26.7 | 27.319 | 37.899 | 134.318 | 4.726 | 278.049 |
| 1 | train | 21.18 | 19.859 | 21.045 | 0.011 | 29.805 | 100.397 | 0.341 | 11.79 | 223542.418 | 0.018 | 155.518 | 2.427 | 371.164 | 0.006 | 45.53 | 45.588 | 62.953 | 188.728 | 5.25 | 307.402 |
| 1 | train | 19.471 | 19.782 | 19.348 | 0.012 | 36.393 | 106.159 | 0.292 | 12.585 | 262255.926 | 0.019 | 236.986 | 2.572 | 55.93 | 0.006 | 42.381 | 42.086 | 58.994 | 180.091 | 5.097 | 314.665 |
| 1 | test | 22.361 | 20.999 | 22.217 | 0.012 | 40.632 | 115.75 | 0.296 | 12.593 | 448203.015 | 0.02 | 353.527 | 2.507 | 222.826 | 0.006 | 47.003 | 46.826 | 64.993 | 193.599 | 5.197 | 316.562 |
| 0 | train | 14.629 | 13.593 | 14.648 | 0.008 | 47.798 | 65.722 | 0.585 | 9.098 | 271403.839 | 0.003 | 166.822 | 2.663 | 841.145 | 0.003 | 40.255 | 41.123 | 55.677 | 174.922 | 5.016 | 292.595 |
| 0 | train | 23.394 | 20.972 | 23.245 | 0.013 | 43.498 | 117.081 | 0.266 | 13.021 | 230590.909 | 0.022 | 251.817 | 2.436 | 210.866 | 0.007 | 48.404 | 47.957 | 66.788 | 196.123 | 5.216 | 317.437 |
| 0 | train | 20.468 | 19.75 | 20.342 | 0.011 | 19.64 | 110.361 | 0.341 | 11.897 | 398752.378 | 0.016 | 236.692 | 2.444 | 270.622 | 0.005 | 44.182 | 44.133 | 61.334 | 186.11 | 5.274 | 311.994 |
| 0 | test | 23.06 | 20.97 | 22.913 | 0.012 | 48.086 | 107.631 | 0.181 | 19.025 | 221311.35 | 0.025 | 204.584 | 2.438 | 104.28 | 0.006 | 47.839 | 47.546 | 66.014 | 195.021 | 5.256 | 312.94 |
| 1 | train | 14.193 | 13.842 | 14.217 | 0.006 | 38.649 | 66.169 | 1.01 | 5.465 | 406433.865 | 0.004 | 237.981 | 2.742 | 1042.239 | 0.002 | 38.837 | 39.808 | 53.759 | 171.496 | 4.831 | 269.746 |
| 0 | train | 15.862 | 15.584 | 15.853 | 0.007 | 47.273 | 63.725 | 1.065 | 5.402 | 273828.282 | 0.005 | 215.668 | 2.502 | 819.073 | 0.003 | 42.358 | 43.243 | 58.526 | 180.386 | 5.211 | 264.664 |
| 1 | test | 14.988 | 15.407 | 14.999 | 0.006 | 48.334 | 71.001 | 1.024 | 5.382 | 456314.308 | 0.003 | 311.621 | 2.584 | 1062.316 | 0.002 | 40.782 | 41.855 | 56.349 | 176.865 | 5.004 | 267.972 |
| 1 | train | 17.169 | 16.4 | 17.111 | 0.008 | 43.877 | 80.519 | 0.525 | 9.346 | 448993.311 | 0.004 | 247.023 | 2.665 | 869.666 | 0.003 | 41.625 | 42.378 | 57.652 | 179.199 | 5.194 | 313.852 |
| 1 | train | 15.791 | 14.938 | 15.767 | 0.007 | 34.831 | 84.357 | 0.545 | 9.138 | 453066.191 | 0.005 | 265.487 | 2.763 | 1092.865 | 0.003 | 39.889 | 40.67 | 55.415 | 174.04 | 5.046 | 279.522 |
| 0 | test | 16.664 | 15.862 | 16.624 | 0.008 | 35.159 | 82.938 | 0.95 | 5.481 | 561152.58 | 0.005 | 358.22 | 2.546 | 1252.639 | 0.003 | 41.785 | 42.75 | 57.731 | 179.751 | 5.224 | 280.784 |
| 0 | train | 16.707 | 16.219 | 16.668 | 0.007 | 38.003 | 81.543 | 0.542 | 9.132 | 349251.722 | 0.005 | 242.735 | 2.514 | 1028.628 | 0.003 | 41.629 | 42.435 | 57.719 | 179.037 | 5.226 | 280.83 |
| 1 | train | 13.031 | 13.243 | 13.02 | 0.006 | 38.686 | 70.005 | 0.552 | 9.058 | 497417.903 | 0.004 | 280.927 | 2.676 | 1134.195 | 0.003 | 35.811 | 36.717 | 49.953 | 162.81 | 5.054 | 285.273 |
| 0 | train | 15.622 | 14.75 | 15.596 | 0.008 | 52.084 | 83.068 | 0.954 | 5.579 | 447378.639 | 0.007 | 323.65 | 2.738 | 1086.973 | 0.003 | 39.742 | 40.514 | 55.009 | 173.606 | 5.144 | 293.564 |
| 1 | train | 16.201 | 15.251 | 16.166 | 0.008 | 38.172 | 83.744 | 0.97 | 5.606 | 377025.509 | 0.005 | 273.31 | 2.732 | 914.427 | 0.003 | 41.159 | 41.936 | 56.973 | 177.336 | 5.092 | 280.648 |
| 0 | train | 14.772 | 12.987 | 14.766 | 0.008 | 40.096 | 79.04 | 0.989 | 5.451 | 333692.87 | 0.008 | 257.808 | 2.693 | 824.428 | 0.003 | 38.616 | 39.397 | 53.624 | 169.508 | 4.933 | 267.964 |
| 0 | train | 16.503 | 16.799 | 16.477 | 0.007 | 45.323 | 76.484 | 0.98 | 5.398 | 385110.908 | 0.004 | 287.537 | 2.646 | 1135.338 | 0.004 | 42.05 | 42.944 | 58.192 | 180.901 | 5.118 | 277.989 |
| 0 | train | 13.939 | 13.64 | 13.943 | 0.007 | 43.172 | 71.905 | 0.982 | 5.435 | 529998.281 | 0.004 | 383.03 | 2.592 | 1226.14 | 0.002 | 38.121 | 39.037 | 53.003 | 169.631 | 5.059 | 262.321 |
| 1 | train | 13.986 | 13.094 | 13.993 | 0.006 | 41.221 | 73.454 | 1.001 | 5.471 | 361386.812 | 0.004 | 184.761 | 2.725 | 914.383 | 0.002 | 38.6 | 39.423 | 53.568 | 170.381 | 4.861 | 274.578 |
| 0 | train | 15.772 | 14.855 | 15.764 | 0.007 | 40.995 | 72.578 | 0.572 | 9.15 | 282209.752 | 0.004 | 149.503 | 2.733 | 756.163 | 0.003 | 41.238 | 42.149 | 57.17 | 177.248 | 4.956 | 284.577 |
| 1 | train | 15.775 | 14.772 | 15.75 | 0.007 | 39.914 | 83.523 | 0.533 | 9.194 | 440071.288 | 0.008 | 251.087 | 2.575 | 1192.073 | 0.003 | 39.856 | 40.606 | 55.373 | 174.223 | 4.943 | 271.134 |
| 1 | test | 13.131 | 12.665 | 13.16 | 0.007 | 42.749 | 62.865 | 1.037 | 5.473 | 279958.179 | 0.004 | 231.245 | 2.531 | 794.053 | 0.002 | 36.886 | 37.782 | 51.192 | 164.966 | 4.926 | 273.355 |
| 1 | test | 13.015 | 12.465 | 13.033 | 0.007 | 39.686 | 68.738 | 0.569 | 9.023 | 376641.218 | 0.006 | 285.647 | 2.593 | 1011.611 | 0.002 | 35.959 | 36.816 | 49.929 | 162.585 | 4.816 | 260.497 |
| 1 | train | 13.241 | 12.983 | 13.244 | 0.007 | 43.204 | 76.454 | 0.978 | 5.514 | 432932.294 | 0.004 | 326.464 | 2.889 | 1075.965 | 0.003 | 35.656 | 36.504 | 49.543 | 161.711 | 4.811 | 261.768 |
| 0 | test | 14.824 | 14.331 | 14.847 | 0.007 | 36.654 | 66.466 | 1.002 | 5.475 | 408338.667 | 0.004 | 278.232 | 2.712 | 1072.29 | 0.003 | 40.176 | 41.12 | 55.529 | 174.845 | 5.039 | 279.993 |
| 1 | train | 14.614 | 13.505 | 14.631 | 0.007 | 40.999 | 72.882 | 0.981 | 5.527 | 306019.295 | 0.003 | 248.081 | 2.466 | 923.201 | 0.003 | 39.804 | 40.558 | 55.18 | 173.545 | 4.979 | 268.146 |
| 2 | test | 9.874 | 9.548 | 9.805 | 0.007 | 28.316 | 64.587 | 0.556 | 8.96 | 578203.053 | 0.006 | 388.331 | 2.935 | 1085.456 | 0.003 | 29.257 | 30.004 | 41.283 | 143.062 | 4.786 | 278.284 |
| 0 | train | 15.11 | 15.394 | 15.11 | 0.007 | 50.146 | 73.146 | 0.617 | 8.981 | 336881.73 | 0.004 | 181.649 | 2.658 | 803.914 | 0.003 | 40.96 | 41.848 | 56.538 | 176.568 | 5.134 | 291.877 |
| 1 | train | 15.426 | 14.514 | 15.399 | 0.007 | 36.784 | 80.058 | 0.898 | 5.655 | 380892.119 | 0.004 | 208.59 | 2.573 | 533.452 | 0.002 | 38.198 | 38.918 | 53.074 | 169.438 | 4.834 | 298.56 |
| 1 | train | 15.987 | 15.159 | 15.951 | 0.007 | 44.145 | 91.458 | 0.899 | 5.663 | 572459.605 | 0.005 | 368.444 | 2.569 | 1132.304 | 0.002 | 39.563 | 40.196 | 54.922 | 173.555 | 4.973 | 294.382 |
| 0 | train | 14.481 | 14.094 | 14.507 | 0.007 | 42.794 | 62.45 | 1.057 | 5.37 | 242458.799 | 0.003 | 216.185 | 2.525 | 773.025 | 0.002 | 40.258 | 41.252 | 55.521 | 174.193 | 4.97 | 270.442 |
| 1 | test | 15.804 | 15.659 | 15.785 | 0.007 | 41.181 | 78.166 | 0.99 | 5.516 | 388421.68 | 0.003 | 230.718 | 2.846 | 861.985 | 0.003 | 40.596 | 41.491 | 56.183 | 176.441 | 5.081 | 268.348 |
| 0 | train | 16.758 | 16.178 | 16.727 | 0.008 | 38.485 | 88.318 | 0.955 | 5.576 | 292286.649 | 0.006 | 206.808 | 2.636 | 892.699 | 0.003 | 41.941 | 42.763 | 57.994 | 180.089 | 5.244 | 275.002 |
| 1 | train | 14.574 | 13.515 | 14.584 | 0.007 | 42.926 | 75.991 | 0.96 | 5.492 | 435129.76 | 0.004 | 312.213 | 2.658 | 1025.375 | 0.002 | 38.666 | 39.526 | 53.58 | 170.487 | 4.709 | 294.656 |
| 0 | test | 16.437 | 15.619 | 16.407 | 0.008 | 41.385 | 76.591 | 1.043 | 5.393 | 339305.162 | 0.004 | 255.727 | 2.593 | 889.401 | 0.003 | 42.024 | 42.906 | 58.043 | 179.308 | 5.343 | 295.298 |
| 0 | train | 14.741 | 14.127 | 14.731 | 0.008 | 39.477 | 80.79 | 0.559 | 9.102 | 331989.672 | 0.006 | 243.601 | 2.599 | 914.486 | 0.003 | 39.026 | 39.78 | 54.208 | 171.215 | 5.092 | 288.207 |
| 0 | train | 16.36 | 16.045 | 16.33 | 0.008 | 45.495 | 86.072 | 0.959 | 5.594 | 325702.647 | 0.004 | 202.649 | 2.73 | 814.589 | 0.003 | 41.098 | 41.98 | 56.879 | 177.252 | 5.148 | 293.473 |
| 0 | train | 14.658 | 14.385 | 14.673 | 0.006 | 42.123 | 62.972 | 0.63 | 8.879 | 355619.287 | 0.004 | 205.834 | 2.638 | 888.082 | 0.002 | 41.375 | 42.372 | 57.162 | 178.157 | 4.92 | 278.591 |
| 1 | test | 13.693 | 12.801 | 13.707 | 0.007 | 46.041 | 71.166 | 0.961 | 5.48 | 389374.105 | 0.006 | 343.362 | 2.483 | 1014.838 | 0.002 | 37.108 | 37.932 | 51.511 | 166.055 | 4.696 | 257.221 |
| 1 | train | 14.884 | 14.657 | 14.899 | 0.007 | 38.417 | 66.33 | 0.597 | 9.001 | 282609.387 | 0.005 | 139.072 | 2.658 | 842.884 | 0.003 | 40.625 | 41.497 | 56.238 | 175.885 | 4.986 | 285.302 |
| 0 | train | 23.635 | 21.319 | 23.484 | 0.011 | 39.546 | 111.074 | 0.334 | 11.971 | 296077.139 | 0.017 | 193.747 | 2.477 | 186.535 | 0.005 | 49.606 | 49.509 | 68.342 | 200.247 | 5.332 | 319.509 |
| 1 | test | 14.86 | 13.596 | 14.862 | 0.007 | 35.214 | 69.511 | 1.028 | 5.452 | 351112.579 | 0.006 | 208.992 | 2.69 | 965.125 | 0.002 | 40.98 | 41.977 | 56.686 | 177.224 | 5.071 | 277.642 |
| 1 | test | 12.876 | 13.018 | 12.856 | 0.008 | 46.707 | 76.171 | 0.94 | 5.471 | 370425.191 | 0.007 | 357.766 | 2.735 | 910.548 | 0.004 | 34.688 | 35.458 | 48.358 | 158.712 | 4.932 | 284.616 |
| 1 | train | 15.093 | 14.154 | 15.08 | 0.007 | 48.71 | 77.218 | 0.937 | 5.569 | 349579.686 | 0.007 | 274.215 | 2.679 | 747.401 | 0.002 | 38.452 | 39.239 | 53.421 | 169.467 | 4.897 | 289.087 |
| 0 | test | 20.498 | 20.116 | 20.365 | 0.012 | 34.816 | 111.756 | 0.19 | 18.691 | 297182.318 | 0.02 | 248.762 | 2.469 | 289.272 | 0.006 | 43.719 | 43.485 | 60.598 | 183.657 | 5.178 | 317.628 |
| 0 | test | 21.48 | 20.562 | 21.342 | 0.012 | 35.193 | 111.062 | 0.287 | 12.695 | 300927.516 | 0.022 | 228.771 | 2.506 | 90.905 | 0.006 | 45.454 | 45.192 | 63.019 | 188.885 | 5.108 | 314.123 |
| 0 | train | 17.526 | 18.003 | 17.467 | 0.008 | 37.093 | 77.648 | 1.069 | 5.187 | 508890.996 | 0.003 | 254.198 | 2.569 | 1171.788 | 0.004 | 44.347 | 45.309 | 61.134 | 187.058 | 5.279 | 302.677 |
| 1 | train | 15.155 | 14.449 | 15.165 | 0.007 | 50.091 | 64.769 | 1.007 | 5.462 | 331561.308 | 0.005 | 296.137 | 2.569 | 894.605 | 0.002 | 40.644 | 41.531 | 56.253 | 175.922 | 4.968 | 277.566 |
| 0 | test | 15.65 | 15.483 | 15.645 | 0.007 | 36.58 | 69.337 | 1.005 | 5.448 | 389513.2 | 0.004 | 266.189 | 2.688 | 1058.997 | 0.003 | 41.182 | 42.138 | 56.932 | 177.917 | 5.142 | 269.812 |
| 1 | train | 13.701 | 12.759 | 13.73 | 0.007 | 47.34 | 78.614 | 1.022 | 5.47 | 281432.101 | 0.004 | 262.47 | 2.436 | 870.448 | 0.002 | 37.936 | 38.791 | 52.731 | 168.191 | 4.812 | 253.806 |
| 2 | test | 7.459 | 8.718 | 7.369 | 0.006 | 37.011 | 70.303 | 0.575 | 8.83 | 509898.912 | 0.007 | 591.094 | 2.434 | 750.883 | 0.003 | 23.231 | 23.931 | 33.114 | 122.946 | 5.006 | 245.539 |
| 1 | train | 13.742 | 12.819 | 13.772 | 0.007 | 42.532 | 69.669 | 0.986 | 5.461 | 287718.433 | 0.006 | 189.474 | 2.572 | 866.468 | 0.002 | 37.768 | 38.619 | 52.418 | 167.388 | 4.84 | 281.672 |
| 0 | train | 14.023 | 13.634 | 14.043 | 0.006 | 40.653 | 68.812 | 1.013 | 5.4 | 361494.222 | 0.004 | 219.459 | 2.594 | 973.38 | 0.002 | 38.959 | 39.877 | 54.003 | 171.434 | 4.871 | 280.581 |
| 0 | test | 14.867 | 15.082 | 14.875 | 0.007 | 44.587 | 67.621 | 1.025 | 5.445 | 321611.402 | 0.003 | 218.107 | 2.648 | 953.717 | 0.003 | 40.407 | 41.296 | 55.921 | 175.022 | 5.085 | 282.717 |
| 1 | test | 14.477 | 13.777 | 14.48 | 0.007 | 41.47 | 77.867 | 0.968 | 5.461 | 450698.924 | 0.006 | 317.262 | 2.676 | 1068.564 | 0.002 | 38.433 | 39.285 | 53.248 | 169.904 | 4.946 | 276.909 |
| 1 | test | 14.269 | 13.775 | 14.266 | 0.007 | 43.362 | 70.726 | 0.96 | 5.485 | 425785.696 | 0.004 | 303.378 | 2.64 | 1129.774 | 0.002 | 37.733 | 38.519 | 52.516 | 167.924 | 4.933 | 277.434 |
| 0 | train | 15.55 | 14.242 | 15.557 | 0.007 | 39.9 | 63.04 | 1.024 | 5.554 | 316002.102 | 0.004 | 251.719 | 2.365 | 787.354 | 0.003 | 41.713 | 42.642 | 57.494 | 178.426 | 5.073 | 264.949 |
| 1 | test | 15.512 | 14.226 | 15.502 | 0.007 | 41.84 | 83.485 | 0.937 | 5.529 | 428735.799 | 0.003 | 300.396 | 2.525 | 1082.865 | 0.002 | 39.243 | 40.065 | 54.343 | 172.573 | 4.841 | 270.734 |
| 1 | train | 15.279 | 14.77 | 15.281 | 0.007 | 47.394 | 72.757 | 1.014 | 5.492 | 284981.663 | 0.004 | 202.403 | 2.57 | 854.313 | 0.003 | 40.731 | 41.545 | 56.319 | 176.251 | 5.09 | 282.657 |
| 1 | train | 12.183 | 12.554 | 12.151 | 0.007 | 30.874 | 76.904 | 0.365 | 13.588 | 469341.988 | 0.005 | 317.646 | 2.817 | 1068.848 | 0.003 | 33.632 | 34.394 | 47.005 | 155.803 | 4.922 | 271.524 |
| 1 | train | 13.91 | 13.738 | 13.873 | 0.007 | 42.206 | 84.346 | 0.897 | 5.567 | 542588.883 | 0.006 | 408.18 | 2.601 | 935.989 | 0.004 | 35.484 | 36.253 | 49.703 | 162.324 | 4.889 | 278.481 |
| 1 | train | 15.789 | 14.725 | 15.765 | 0.007 | 48.229 | 76.684 | 0.938 | 5.472 | 378795.008 | 0.004 | 392.703 | 2.529 | 1046.589 | 0.003 | 39.934 | 40.728 | 55.401 | 174.52 | 4.956 | 284.432 |
| 0 | train | 14.362 | 14.967 | 14.388 | 0.006 | 42.486 | 81.605 | 1.037 | 5.47 | 384872.839 | 0.004 | 191.978 | 2.739 | 875.698 | 0.002 | 39.581 | 40.548 | 54.708 | 172.979 | 4.955 | 278.776 |
| 1 | train | 12.258 | 12.292 | 12.242 | 0.008 | 45.141 | 70.603 | 0.561 | 9.156 | 367064.524 | 0.005 | 270.992 | 2.748 | 852.157 | 0.004 | 34.398 | 35.132 | 47.882 | 157.794 | 4.903 | 253.613 |
| 0 | test | 15.736 | 14.583 | 15.712 | 0.007 | 36.683 | 78.717 | 0.37 | 13.52 | 336411.328 | 0.004 | 238.315 | 2.627 | 857.039 | 0.003 | 40.739 | 41.575 | 56.354 | 175.943 | 5.162 | 269.854 |
| 1 | test | 13.477 | 13.091 | 13.444 | 0.008 | 34.493 | 74.434 | 0.533 | 9.183 | 420321.045 | 0.007 | 340.911 | 2.65 | 1072.947 | 0.003 | 35.108 | 35.844 | 48.942 | 160.525 | 4.865 | 274.43 |
| 0 | train | 15.825 | 15.962 | 15.813 | 0.007 | 40.554 | 77.376 | 0.995 | 5.447 | 330754.622 | 0.004 | 232.863 | 2.644 | 885.188 | 0.003 | 41.081 | 41.968 | 56.805 | 177.362 | 5.065 | 278.344 |
| 0 | train | 17.466 | 16.96 | 17.403 | 0.008 | 41.241 | 78.919 | 0.963 | 5.534 | 343227.132 | 0.006 | 210.251 | 2.613 | 902.451 | 0.003 | 43.689 | 44.597 | 60.421 | 184.676 | 5.374 | 295.16 |
| 1 | train | 14.018 | 13.623 | 13.979 | 0.008 | 40.144 | 81.863 | 0.509 | 9.435 | 580630.03 | 0.007 | 485.147 | 2.695 | 1084.626 | 0.004 | 35.561 | 36.245 | 49.668 | 161.996 | 4.92 | 288.761 |
| 1 | train | 14.135 | 13.801 | 14.158 | 0.007 | 40.365 | 72.097 | 0.986 | 5.44 | 379778.178 | 0.004 | 308.797 | 2.658 | 930.311 | 0.002 | 38.393 | 39.346 | 53.184 | 169.606 | 4.763 | 271.731 |
| 1 | train | 12.783 | 13.28 | 12.775 | 0.007 | 50.399 | 69.65 | 0.578 | 8.981 | 384488.787 | 0.003 | 233.914 | 2.692 | 932.849 | 0.003 | 35.253 | 36.11 | 49.225 | 160.725 | 4.899 | 288.566 |
| 0 | test | 17.485 | 17.214 | 17.438 | 0.008 | 43.077 | 91.262 | 0.948 | 5.528 | 314576.832 | 0.006 | 214.586 | 2.56 | 918.789 | 0.004 | 42.864 | 43.714 | 59.037 | 182.21 | 5.268 | 277.048 |
| 1 | train | 12.66 | 12.572 | 12.655 | 0.008 | 46.25 | 71.608 | 0.97 | 5.461 | 319112.847 | 0.007 | 312.526 | 2.371 | 875.036 | 0.004 | 35.239 | 36.043 | 49.138 | 160.34 | 5.019 | 254.439 |
| 0 | test | 15.664 | 15.141 | 15.656 | 0.007 | 35.892 | 73.826 | 0.972 | 5.505 | 414632.129 | 0.003 | 315.048 | 2.67 | 1009.222 | 0.002 | 40.907 | 41.834 | 56.512 | 177.115 | 4.912 | 262.657 |
| 1 | train | 15.278 | 13.935 | 15.268 | 0.007 | 42.494 | 77.465 | 0.961 | 5.533 | 345423.189 | 0.005 | 282.9 | 2.541 | 931.022 | 0.003 | 39.637 | 40.342 | 55.078 | 173.48 | 5.01 | 288.605 |
| 1 | train | 13.713 | 13.066 | 13.714 | 0.007 | 34.69 | 74.736 | 0.963 | 5.47 | 490045.421 | 0.004 | 309.039 | 2.549 | 1082.242 | 0.002 | 36.665 | 37.475 | 51.006 | 164.99 | 4.738 | 267.287 |
| 1 | train | 20.77 | 20.283 | 20.636 | 0.013 | 31.548 | 108.797 | 0.173 | 19.322 | 242630.284 | 0.021 | 252.49 | 2.454 | 184.124 | 0.007 | 44.495 | 44.055 | 61.882 | 185.967 | 5.238 | 318.547 |
| 0 | train | 18.42 | 19 | 18.307 | 0.011 | 32.734 | 105.328 | 0.33 | 11.936 | 275449.979 | 0.014 | 272.117 | 2.631 | 149.124 | 0.005 | 41.216 | 41.061 | 57.573 | 177.238 | 5.227 | 308.032 |
| 1 | train | 20.923 | 20.635 | 20.789 | 0.013 | 24.969 | 115.321 | 0.171 | 19.401 | 279455.703 | 0.022 | 253.147 | 2.457 | 126.721 | 0.007 | 44.394 | 43.932 | 61.739 | 185.382 | 5.065 | 312.55 |
| 1 | train | 14.086 | 13.589 | 14.101 | 0.007 | 44.822 | 72.696 | 0.993 | 5.466 | 334741.808 | 0.004 | 236.029 | 2.626 | 909.653 | 0.003 | 38.653 | 39.493 | 53.679 | 170.314 | 5.043 | 278.604 |
| 0 | train | 17.591 | 18.631 | 17.54 | 0.007 | 45.307 | 74.04 | 0.98 | 5.474 | 455136.955 | 0.004 | 323.424 | 2.548 | 1169.295 | 0.004 | 44.354 | 45.27 | 61.096 | 186.637 | 5.035 | 285.266 |
| 1 | test | 13.929 | 13.23 | 13.957 | 0.007 | 35.729 | 65.507 | 0.997 | 5.462 | 331070.554 | 0.004 | 203.426 | 2.718 | 899.905 | 0.002 | 38.327 | 39.244 | 53.101 | 169.136 | 5.037 | 273.093 |
| 0 | train | 15.047 | 14.175 | 15.046 | 0.007 | 40.15 | 68.581 | 1.052 | 5.489 | 325059.487 | 0.004 | 182.443 | 2.421 | 858.186 | 0.003 | 40.91 | 41.767 | 56.645 | 176.452 | 5.132 | 260.364 |
| 1 | train | 12.433 | 12.752 | 12.388 | 0.007 | 42.174 | 75.287 | 0.944 | 5.45 | 455589.415 | 0.004 | 373.845 | 2.654 | 971.956 | 0.003 | 33.498 | 34.256 | 46.893 | 155.653 | 5.045 | 276.293 |
| 0 | train | 15.934 | 15.712 | 15.929 | 0.006 | 53.978 | 67.735 | 0.598 | 8.915 | 325182.197 | 0.004 | 225.765 | 2.658 | 909.271 | 0.003 | 42.789 | 43.765 | 59.076 | 182.09 | 5.084 | 281.54 |
| 1 | train | 13.546 | 13.25 | 13.505 | 0.009 | 43.453 | 80.423 | 0.911 | 5.588 | 366369.923 | 0.009 | 304.042 | 2.665 | 1013.85 | 0.004 | 35.017 | 35.627 | 48.895 | 159.761 | 4.995 | 282.532 |
| 1 | test | 12.265 | 12.723 | 12.268 | 0.007 | 44.844 | 64.007 | 0.993 | 5.426 | 313359.501 | 0.005 | 320.418 | 2.332 | 898.68 | 0.003 | 34.861 | 35.767 | 48.491 | 159.107 | 4.963 | 265.022 |
| 2 | train | 10.917 | 11.132 | 10.848 | 0.007 | 31.269 | 70.09 | 0.957 | 5.488 | 406892.541 | 0.007 | 299.709 | 2.671 | 649.076 | 0.003 | 30.837 | 31.635 | 43.271 | 147.564 | 5.005 | 254.925 |
| 0 | train | 15.872 | 14.656 | 15.856 | 0.008 | 40.903 | 75.839 | 1 | 5.474 | 345081.764 | 0.007 | 209.209 | 2.578 | 871.206 | 0.003 | 41.397 | 42.144 | 57.352 | 177.552 | 5.193 | 275.356 |
| 1 | test | 16.169 | 15.184 | 16.143 | 0.008 | 42.747 | 80.189 | 0.526 | 9.329 | 363318.455 | 0.007 | 253.82 | 2.607 | 879.825 | 0.003 | 40.146 | 40.839 | 55.692 | 174.829 | 5.061 | 283.431 |
| 0 | train | 22.158 | 20.631 | 22.017 | 0.013 | 31.48 | 112.177 | 0.285 | 12.752 | 274607.589 | 0.022 | 267.397 | 2.416 | 123.769 | 0.007 | 46.497 | 46.043 | 64.502 | 191.352 | 5.27 | 311.439 |
| 1 | train | 13.54 | 13.219 | 13.589 | 0.007 | 33.232 | 73.345 | 0.992 | 5.46 | 338061.272 | 0.005 | 190.352 | 2.643 | 1008.351 | 0.003 | 38.527 | 39.362 | 53.369 | 169.916 | 4.763 | 281.35 |
| 0 | train | 13.195 | 14.35 | 13.241 | 0.005 | 54.428 | 61.633 | 0.4 | 13.222 | 465257.544 | 0.004 | 260.357 | 2.755 | 1011.074 | 0.002 | 37.839 | 38.894 | 52.561 | 168.118 | 4.961 | 301.151 |
| 2 | train | 10.213 | 10.204 | 10.14 | 0.007 | 29.593 | 64.584 | 0.585 | 8.821 | 648493.11 | 0.007 | 393.029 | 2.931 | 1089.431 | 0.003 | 29.562 | 30.324 | 41.568 | 143.882 | 4.993 | 291.959 |
| 1 | train | 15.661 | 14.921 | 15.634 | 0.007 | 33.81 | 88.561 | 0.544 | 9.215 | 424803.26 | 0.005 | 268.168 | 2.681 | 865.312 | 0.003 | 39.46 | 40.287 | 54.752 | 172.579 | 5.121 | 302.802 |
| 0 | test | 14.215 | 13.477 | 14.222 | 0.007 | 41.45 | 78.763 | 0.374 | 13.56 | 443052.506 | 0.006 | 264.297 | 2.619 | 1162.165 | 0.002 | 37.915 | 38.799 | 52.54 | 168.395 | 5.02 | 289.916 |
| 0 | train | 14.303 | 13.662 | 14.323 | 0.007 | 45.111 | 69.707 | 0.976 | 5.439 | 437865.517 | 0.004 | 259.337 | 2.586 | 1144.14 | 0.002 | 38.854 | 39.68 | 53.926 | 171.362 | 4.808 | 284.756 |
| 1 | test | 13.88 | 13.557 | 13.836 | 0.008 | 43.014 | 84.423 | 0.523 | 9.294 | 460197.277 | 0.007 | 316.715 | 2.717 | 931.83 | 0.004 | 35.289 | 35.946 | 49.271 | 160.71 | 5.022 | 299.521 |
| 1 | test | 15.213 | 14.522 | 15.165 | 0.008 | 31.648 | 86.244 | 0.509 | 9.58 | 503538.144 | 0.009 | 389.812 | 2.84 | 1075.981 | 0.003 | 37.527 | 38.115 | 52.314 | 167.469 | 4.956 | 291.615 |
| 0 | train | 15.133 | 14.09 | 15.136 | 0.007 | 42.969 | 74.083 | 0.998 | 5.46 | 412119.374 | 0.005 | 279.6 | 2.542 | 1017.773 | 0.002 | 40.536 | 41.469 | 56.092 | 175.69 | 5.064 | 262.1 |
| 1 | train | 13.998 | 13.514 | 13.96 | 0.008 | 41.456 | 80.303 | 0.517 | 9.482 | 602180.602 | 0.009 | 434.152 | 2.65 | 1095.261 | 0.004 | 35.971 | 36.601 | 50.228 | 163.09 | 5.129 | 289.857 |
| 1 | train | 13.519 | 13.545 | 13.522 | 0.006 | 50.141 | 71.636 | 0.582 | 9.046 | 423940.894 | 0.004 | 266.776 | 2.687 | 963.261 | 0.002 | 37.647 | 38.506 | 52.479 | 167.821 | 4.959 | 286.781 |
| 1 | train | 13.332 | 13.15 | 13.313 | 0.007 | 43.344 | 77.46 | 0.94 | 5.499 | 489480.642 | 0.008 | 435.018 | 2.648 | 1066.576 | 0.003 | 35.179 | 35.962 | 48.975 | 160.741 | 4.732 | 271.749 |
| 1 | train | 15.513 | 14.587 | 15.463 | 0.008 | 39.786 | 84.829 | 0.516 | 9.512 | 402125.037 | 0.009 | 264.526 | 2.615 | 993.952 | 0.003 | 38.339 | 38.863 | 53.413 | 169.296 | 5.067 | 283.234 |
| 1 | train | 14.441 | 13.56 | 14.432 | 0.007 | 44.143 | 80.017 | 0.941 | 5.57 | 366651.014 | 0.009 | 262.262 | 2.768 | 933.608 | 0.003 | 37.409 | 38.14 | 52.009 | 167.068 | 4.734 | 273.756 |
| 1 | train | 13.886 | 13.711 | 13.902 | 0.006 | 40.762 | 62.548 | 0.604 | 9.006 | 397124.845 | 0.005 | 194.715 | 2.764 | 986.477 | 0.002 | 38.791 | 39.752 | 53.87 | 170.618 | 4.934 | 270.863 |
| 1 | test | 14.346 | 13.488 | 14.333 | 0.007 | 42.864 | 76.618 | 0.612 | 8.618 | 557713.712 | 0.004 | 362.316 | 2.756 | 939.438 | 0.002 | 37.147 | 38.001 | 51.655 | 166.155 | 4.962 | 296.067 |
| 0 | train | 15.526 | 14.165 | 15.525 | 0.008 | 48.456 | 80.902 | 0.563 | 9.098 | 307320.078 | 0.004 | 247.768 | 2.554 | 868.191 | 0.003 | 40.837 | 41.628 | 56.491 | 176.043 | 5.106 | 272.093 |
| 0 | train | 15.746 | 14.78 | 15.737 | 0.007 | 36.834 | 82.477 | 1.004 | 5.429 | 369247.324 | 0.005 | 235.457 | 2.488 | 946.574 | 0.003 | 41.813 | 42.616 | 57.779 | 179.15 | 5.149 | 264.276 |
| 1 | train | 13.61 | 13.482 | 13.633 | 0.006 | 37.549 | 70.98 | 1.028 | 5.451 | 352363.624 | 0.005 | 167.15 | 2.766 | 842.384 | 0.002 | 37.806 | 38.698 | 52.561 | 167.973 | 4.973 | 302.372 |
| 1 | train | 13.609 | 13.514 | 13.586 | 0.007 | 48.787 | 82.246 | 0.931 | 5.504 | 500425.97 | 0.005 | 457.894 | 2.681 | 1192.274 | 0.003 | 35.504 | 36.275 | 49.557 | 161.694 | 4.851 | 285.269 |
| 1 | train | 15.287 | 14.891 | 15.288 | 0.007 | 46.237 | 79.912 | 1.001 | 5.433 | 350622.973 | 0.007 | 315.496 | 2.515 | 965.431 | 0.003 | 40.471 | 41.278 | 55.935 | 175.625 | 5.011 | 267.299 |
| 2 | test | 9.992 | 10.29 | 9.909 | 0.007 | 38.007 | 68.271 | 0.539 | 9.152 | 453404.739 | 0.008 | 430.702 | 2.768 | 911.471 | 0.003 | 28.259 | 28.875 | 39.861 | 139.216 | 4.898 | 274.471 |
| 0 | train | 13.906 | 13.905 | 13.939 | 0.006 | 46.454 | 69.784 | 1.009 | 5.444 | 331795.815 | 0.003 | 260.041 | 2.508 | 944.033 | 0.002 | 39.386 | 40.342 | 54.418 | 172.952 | 4.839 | 252.028 |
| 1 | train | 15.087 | 14.007 | 15.078 | 0.007 | 30.214 | 71.992 | 0.924 | 5.572 | 387671.894 | 0.007 | 246.442 | 2.578 | 988.451 | 0.002 | 38.729 | 39.463 | 53.791 | 170.914 | 4.899 | 297.148 |
| 0 | train | 15.711 | 14.867 | 15.668 | 0.008 | 33.739 | 84.534 | 0.934 | 5.545 | 365053.449 | 0.005 | 264.694 | 2.603 | 920.312 | 0.003 | 38.425 | 39.149 | 53.321 | 169.557 | 4.994 | 283.582 |
| 0 | train | 16.098 | 15.055 | 16.076 | 0.008 | 43.696 | 84.749 | 0.564 | 9.153 | 313948.467 | 0.004 | 192.162 | 2.546 | 874.384 | 0.003 | 40.818 | 41.682 | 56.486 | 176.003 | 5.277 | 284.88 |
| 1 | train | 14.031 | 12.791 | 14.049 | 0.007 | 27.805 | 72.42 | 0.63 | 8.762 | 435869.105 | 0.005 | 235.745 | 2.599 | 992.977 | 0.002 | 38.75 | 39.593 | 53.883 | 170.695 | 4.945 | 288.879 |
| 1 | train | 12.966 | 12.928 | 12.945 | 0.007 | 38.294 | 78.47 | 0.989 | 5.361 | 424177.539 | 0.005 | 399.806 | 2.598 | 1075.047 | 0.003 | 34.64 | 35.479 | 48.194 | 159.039 | 4.924 | 265.602 |
| 0 | train | 16.81 | 16.702 | 16.776 | 0.008 | 46.287 | 75.523 | 0.555 | 9.079 | 349329.804 | 0.003 | 254.267 | 2.627 | 1014.741 | 0.003 | 42.443 | 43.323 | 58.608 | 181.006 | 5.184 | 279.944 |
| 1 | train | 14.656 | 14.368 | 14.665 | 0.006 | 45.123 | 67.615 | 0.585 | 9.043 | 372324.084 | 0.004 | 189.381 | 2.709 | 824.336 | 0.002 | 39.653 | 40.583 | 55.011 | 172.845 | 4.852 | 296.391 |
| 1 | test | 14.697 | 14.132 | 14.709 | 0.006 | 46.406 | 76.338 | 0.968 | 5.489 | 579092.105 | 0.003 | 350.567 | 2.705 | 1193.685 | 0.002 | 39.454 | 40.367 | 54.711 | 173.285 | 4.741 | 284.246 |
| 0 | test | 15.199 | 14.918 | 15.205 | 0.007 | 46.33 | 68.553 | 1.006 | 5.405 | 412740.583 | 0.003 | 285.563 | 2.578 | 1089.533 | 0.002 | 40.652 | 41.628 | 56.143 | 176.207 | 5.087 | 280.618 |
| 0 | train | 16.251 | 15.561 | 16.219 | 0.008 | 31.064 | 79.962 | 0.609 | 8.891 | 300844.485 | 0.008 | 178.764 | 2.468 | 874.348 | 0.003 | 41.186 | 42.077 | 56.958 | 177.557 | 5.345 | 294.628 |
| 1 | test | 13.664 | 12.984 | 13.675 | 0.007 | 44.765 | 75.375 | 0.557 | 9.029 | 351347.128 | 0.004 | 234.343 | 2.538 | 925.967 | 0.003 | 37.219 | 38.048 | 51.754 | 166.13 | 4.833 | 285.04 |
| 1 | test | 17.598 | 16.837 | 17.512 | 0.009 | 48.269 | 95.626 | 0.452 | 10.217 | 537464.861 | 0.011 | 429.775 | 2.594 | 1092.21 | 0.004 | 40.631 | 41.081 | 56.529 | 176.554 | 5.146 | 306.941 |
| 1 | train | 14.599 | 13.273 | 14.606 | 0.007 | 34.14 | 68.486 | 0.976 | 5.474 | 433368.207 | 0.003 | 312.498 | 2.57 | 1118.858 | 0.002 | 38.965 | 39.782 | 54.018 | 171.623 | 4.93 | 277.743 |
| 1 | train | 13.305 | 13.46 | 13.272 | 0.007 | 45.936 | 75.18 | 0.936 | 5.512 | 504834.657 | 0.006 | 341.515 | 2.706 | 1102.545 | 0.003 | 34.901 | 35.632 | 48.617 | 159.764 | 4.865 | 276.437 |
| 1 | train | 14.45 | 13.637 | 14.46 | 0.007 | 49.445 | 81.371 | 0.99 | 5.459 | 326228.04 | 0.003 | 252.009 | 2.62 | 907.756 | 0.002 | 38.677 | 39.628 | 53.768 | 171.059 | 4.955 | 302.151 |
| 1 | train | 20.001 | 19.185 | 19.878 | 0.01 | 26.293 | 110.544 | 0.227 | 17.362 | 342640.651 | 0.017 | 206.415 | 2.543 | 284.02 | 0.005 | 43.336 | 43.427 | 60.054 | 183.216 | 5.193 | 308.269 |
| 0 | test | 14.484 | 13.944 | 14.482 | 0.007 | 38.716 | 72.386 | 0.593 | 8.709 | 531274.621 | 0.007 | 305.252 | 2.641 | 1215.932 | 0.002 | 38.712 | 39.658 | 53.743 | 171.238 | 4.962 | 286.411 |
| 1 | test | 14.962 | 13.973 | 14.959 | 0.007 | 34.174 | 67.777 | 0.609 | 8.864 | 391054.781 | 0.003 | 246.692 | 2.695 | 974.761 | 0.002 | 39.932 | 40.901 | 55.322 | 174.261 | 5.101 | 261.625 |
| 0 | train | 16.31 | 15.223 | 16.277 | 0.008 | 36.443 | 81.339 | 0.617 | 8.859 | 418267.628 | 0.003 | 203.913 | 2.673 | 986.961 | 0.003 | 42.674 | 43.476 | 59.045 | 181.439 | 5.404 | 304.173 |
| 1 | train | 13.35 | 12.352 | 13.38 | 0.008 | 38.933 | 63.504 | 0.641 | 8.649 | 286341.542 | 0.004 | 149.304 | 2.678 | 849.985 | 0.003 | 37.667 | 38.545 | 52.181 | 167.135 | 4.843 | 295.806 |
| 0 | train | 16.683 | 15.747 | 16.64 | 0.007 | 47.108 | 88.535 | 0.555 | 9.1 | 501102.825 | 0.007 | 307.73 | 2.516 | 1158.738 | 0.003 | 41.492 | 42.33 | 57.416 | 178.858 | 5.212 | 291.614 |
| 1 | train | 14.272 | 12.875 | 14.287 | 0.007 | 44.427 | 70.999 | 1.02 | 5.414 | 338899.908 | 0.007 | 284.679 | 2.442 | 946.378 | 0.002 | 39.047 | 39.945 | 54.097 | 171.302 | 4.987 | 265.941 |
| 1 | test | 13.474 | 13.153 | 13.469 | 0.006 | 37.236 | 72.272 | 0.956 | 5.481 | 678089.729 | 0.005 | 401.855 | 2.724 | 1331.509 | 0.002 | 36.167 | 37.025 | 50.296 | 164.166 | 4.858 | 283.39 |
| 1 | train | 15.693 | 15.398 | 15.682 | 0.007 | 28.538 | 82.523 | 0.648 | 8.571 | 416597.671 | 0.004 | 190.358 | 2.509 | 992.763 | 0.003 | 41.81 | 42.806 | 57.864 | 179.269 | 5.207 | 287.088 |
| 0 | test | 15.1 | 13.865 | 15.097 | 0.008 | 32.442 | 74.958 | 0.594 | 8.661 | 326365.415 | 0.006 | 200.406 | 2.606 | 885.625 | 0.003 | 39.205 | 40.13 | 54.294 | 171.704 | 5.101 | 294.332 |
| 1 | train | 14.962 | 13.118 | 14.974 | 0.007 | 38.637 | 71.822 | 0.561 | 9.232 | 314948.201 | 0.005 | 234.131 | 2.645 | 885.98 | 0.002 | 39.47 | 40.314 | 54.821 | 172.597 | 4.817 | 261.826 |
| 0 | test | 15.523 | 15.451 | 15.52 | 0.007 | 40.059 | 67.567 | 1.02 | 5.406 | 351121.285 | 0.004 | 226.503 | 2.666 | 926.473 | 0.003 | 41.455 | 42.419 | 57.135 | 178.107 | 5.003 | 272.906 |
| 1 | train | 14.863 | 13.488 | 14.861 | 0.006 | 41.182 | 71.912 | 1.036 | 5.33 | 520221.569 | 0.008 | 247.063 | 2.728 | 1122.484 | 0.002 | 39.607 | 40.52 | 54.982 | 173.461 | 4.873 | 281.947 |
| 2 | train | 10.829 | 11.364 | 10.739 | 0.007 | 35.688 | 65.719 | 0.987 | 5.377 | 473114.774 | 0.006 | 337.022 | 2.838 | 1163.688 | 0.004 | 30.803 | 31.634 | 43.132 | 147.55 | 4.975 | 277.254 |
| 1 | train | 20.232 | 19.722 | 20.102 | 0.012 | 46.762 | 102.049 | 0.201 | 18.188 | 239419.148 | 0.024 | 221.519 | 2.433 | 339.065 | 0.006 | 43.612 | 43.484 | 60.658 | 183.402 | 5.164 | 308.683 |
| 1 | train | 14.343 | 13.256 | 14.337 | 0.008 | 43.087 | 81.509 | 0.548 | 9.17 | 303922.54 | 0.004 | 226.28 | 2.651 | 841.267 | 0.002 | 37.267 | 38.014 | 51.814 | 166.075 | 4.803 | 290.67 |
| 1 | train | 14.679 | 14.021 | 14.654 | 0.007 | 43.924 | 83.622 | 0.527 | 9.302 | 472867.309 | 0.006 | 401.711 | 2.72 | 1086.64 | 0.002 | 36.993 | 37.704 | 51.488 | 165.976 | 4.855 | 295.206 |
| 1 | train | 15.358 | 14.114 | 15.319 | 0.008 | 46.236 | 89.074 | 0.892 | 5.646 | 328255.169 | 0.006 | 359.381 | 2.578 | 846.674 | 0.003 | 37.923 | 38.546 | 52.75 | 167.916 | 4.958 | 280.304 |
| 1 | test | 21.07 | 20.574 | 20.934 | 0.013 | 39.972 | 116.134 | 0.169 | 19.52 | 267136.709 | 0.025 | 289.621 | 2.448 | 147.044 | 0.007 | 44.778 | 44.361 | 62.216 | 186.459 | 5.198 | 317.723 |
| 1 | train | 19.878 | 19.76 | 19.753 | 0.012 | 27.255 | 105.605 | 0.311 | 12.249 | 287102.646 | 0.022 | 244.314 | 2.506 | 218.337 | 0.006 | 43.047 | 42.817 | 59.845 | 181.996 | 5.088 | 308.235 |
| 1 | train | 13.179 | 13.269 | 13.16 | 0.007 | 38.479 | 75.376 | 0.957 | 5.453 | 409431.902 | 0.006 | 335.829 | 2.613 | 1015.173 | 0.003 | 35.202 | 36.011 | 49.003 | 160.754 | 4.792 | 268.095 |
| 1 | test | 13.807 | 12.84 | 13.803 | 0.007 | 40.664 | 72.085 | 0.958 | 5.511 | 300617.704 | 0.009 | 207.441 | 2.703 | 804.995 | 0.002 | 36.686 | 37.442 | 50.976 | 164.553 | 4.732 | 272.396 |
| 0 | train | 15.657 | 14.815 | 15.624 | 0.007 | 51.803 | 89.897 | 0.908 | 5.617 | 388905.434 | 0.007 | 327.179 | 2.66 | 785.379 | 0.002 | 38.613 | 39.401 | 53.639 | 170.662 | 4.935 | 278.655 |
| 1 | test | 16.112 | 15.252 | 16.083 | 0.007 | 45.488 | 80.314 | 0.543 | 9.229 | 573401.72 | 0.004 | 358.239 | 2.656 | 1104.735 | 0.003 | 40.605 | 41.464 | 56.212 | 176.403 | 5.067 | 278.013 |
| 0 | train | 16.934 | 16.86 | 16.889 | 0.007 | 38.817 | 76.659 | 0.582 | 9.073 | 462621.377 | 0.005 | 219.602 | 2.635 | 1087.731 | 0.003 | 44.268 | 45.206 | 61.114 | 186.45 | 5.232 | 274.238 |
| 0 | test | 16.886 | 16.188 | 16.849 | 0.008 | 40.45 | 80.631 | 0.952 | 5.538 | 301339.173 | 0.004 | 242.077 | 2.71 | 890.74 | 0.004 | 42.002 | 42.853 | 57.944 | 179.519 | 5.278 | 278.38 |
| 1 | train | 14.823 | 14.512 | 14.823 | 0.007 | 38.268 | 79.332 | 0.956 | 5.518 | 373846.888 | 0.006 | 255.121 | 2.689 | 1010.626 | 0.002 | 38.956 | 39.806 | 54.073 | 171.861 | 4.969 | 279.877 |
| 0 | test | 14.792 | 14.058 | 14.811 | 0.006 | 43.264 | 68.832 | 1.069 | 5.452 | 314011.246 | 0.005 | 178.557 | 2.766 | 813.953 | 0.002 | 41.077 | 42.027 | 56.829 | 177.183 | 4.982 | 280.543 |
| 1 | test | 15.118 | 13.961 | 15.114 | 0.007 | 41.486 | 80.275 | 0.553 | 9.057 | 432142.975 | 0.004 | 277.485 | 2.555 | 1116.209 | 0.002 | 39.143 | 40.01 | 54.234 | 171.722 | 4.789 | 291.824 |
| 2 | train | 11.906 | 13.534 | 11.82 | 0.009 | 31.759 | 88.787 | 0.432 | 10.398 | 572759.426 | 0.011 | 522.808 | 2.779 | 195.409 | 0.004 | 30.185 | 30.478 | 42.726 | 145.477 | 5.017 | 297.071 |
| 2 | train | 9.723 | 10.461 | 9.639 | 0.008 | 42.445 | 78.919 | 0.898 | 5.595 | 390900.279 | 0.008 | 413.289 | 2.848 | 538.969 | 0.003 | 27.166 | 27.71 | 38.526 | 136.114 | 4.871 | 273.064 |
| 1 | train | 12.985 | 12.766 | 12.976 | 0.007 | 40.479 | 72.341 | 0.552 | 9.043 | 466288.083 | 0.006 | 392.097 | 2.693 | 1161.863 | 0.003 | 35.123 | 35.968 | 48.967 | 160.585 | 4.867 | 263.247 |
| 1 | train | 14.209 | 13.529 | 14.216 | 0.006 | 42.993 | 71.332 | 1.019 | 5.467 | 429757.302 | 0.005 | 307.091 | 2.509 | 970.216 | 0.002 | 38.797 | 39.667 | 53.863 | 170.839 | 5.09 | 260.933 |
| 0 | test | 15.32 | 15.113 | 15.321 | 0.007 | 43.134 | 69.483 | 1.006 | 5.438 | 417443.813 | 0.003 | 269.159 | 2.694 | 1067.315 | 0.003 | 40.636 | 41.539 | 56.24 | 176.364 | 4.96 | 296.882 |
| 1 | train | 15.073 | 14.574 | 15.084 | 0.006 | 42.085 | 78.617 | 1.023 | 5.485 | 419267.832 | 0.003 | 219.223 | 2.832 | 948.668 | 0.002 | 40.665 | 41.618 | 56.263 | 176.304 | 4.908 | 282.57 |
| 1 | train | 13.268 | 12.667 | 13.238 | 0.008 | 35.56 | 76.573 | 0.535 | 9.238 | 374005.036 | 0.007 | 281.899 | 2.731 | 894.911 | 0.004 | 35.068 | 35.71 | 48.913 | 160.229 | 4.938 | 283.087 |
| 1 | train | 12.687 | 12.997 | 12.625 | 0.008 | 30.639 | 86.804 | 0.526 | 9.194 | 601586.009 | 0.009 | 502.84 | 2.66 | 1141.917 | 0.005 | 33.076 | 33.694 | 46.269 | 154.75 | 4.961 | 308.096 |
| 0 | train | 16.486 | 17.097 | 16.46 | 0.007 | 39.225 | 75.973 | 1.027 | 5.495 | 368852.75 | 0.004 | 207.988 | 2.687 | 969.382 | 0.003 | 42.673 | 43.623 | 58.861 | 182.033 | 5.079 | 282.057 |
| 0 | test | 15.575 | 14.694 | 15.57 | 0.008 | 44.484 | 74.409 | 1.029 | 5.494 | 266273.49 | 0.002 | 226.684 | 2.427 | 838.8 | 0.003 | 41.715 | 42.656 | 57.46 | 178.198 | 5.129 | 262.356 |
| 2 | test | 11.482 | 11.734 | 11.409 | 0.008 | 32.491 | 74.004 | 0.933 | 5.487 | 625809.032 | 0.004 | 659.363 | 2.788 | 1375.102 | 0.004 | 31.432 | 32.199 | 43.97 | 149.879 | 5.035 | 272.218 |
| 0 | train | 14.145 | 13.413 | 14.164 | 0.007 | 39.604 | 55.938 | 1.117 | 5.397 | 227524.372 | 0.003 | 196.957 | 2.525 | 711.634 | 0.002 | 40.672 | 41.587 | 56.153 | 175.038 | 5.078 | 265.36 |
| 1 | train | 13.274 | 13.213 | 13.309 | 0.006 | 32.693 | 73.263 | 1.017 | 5.475 | 368689.53 | 0.004 | 226.484 | 2.85 | 843.656 | 0.002 | 37.744 | 38.632 | 52.383 | 167.144 | 4.682 | 261.425 |
| 0 | test | 16.166 | 15.493 | 16.148 | 0.008 | 31.421 | 77.287 | 0.59 | 8.928 | 424952.325 | 0.004 | 239.715 | 2.577 | 1063.992 | 0.003 | 42.282 | 43.217 | 58.227 | 180.657 | 5.218 | 273.97 |
| 1 | train | 13.471 | 14.171 | 13.514 | 0.006 | 49.563 | 62.174 | 0.591 | 8.992 | 330144.682 | 0.004 | 174.385 | 2.673 | 887.185 | 0.002 | 39.241 | 40.256 | 54.305 | 171.943 | 4.83 | 273.427 |
| 2 | train | 10.034 | 10.046 | 9.95 | 0.006 | 39.707 | 68.617 | 0.543 | 9.025 | 515774.687 | 0.006 | 330.207 | 2.718 | 361.371 | 0.003 | 28.749 | 29.517 | 40.513 | 141.753 | 4.595 | 282.684 |
| 1 | test | 14.208 | 13.606 | 14.229 | 0.007 | 47.836 | 74.602 | 0.568 | 8.982 | 388746.617 | 0.004 | 280.662 | 2.604 | 1028.552 | 0.002 | 38.384 | 39.37 | 53.246 | 170.142 | 4.824 | 276.962 |
| 1 | train | 16.399 | 15.828 | 16.346 | 0.008 | 51.506 | 93.845 | 0.857 | 5.774 | 388654.646 | 0.011 | 379.758 | 2.667 | 1050.712 | 0.003 | 39.334 | 39.927 | 54.618 | 172.757 | 5.025 | 300.441 |
| 2 | train | 11.743 | 12.879 | 11.66 | 0.009 | 33.391 | 83.343 | 0.53 | 9.316 | 861222.693 | 0.008 | 648.986 | 2.785 | 685.573 | 0.004 | 30.222 | 30.539 | 42.82 | 145.841 | 5.12 | 289.929 |
| 2 | test | 14.766 | 19.025 | 14.659 | 0.013 | 42.307 | 98.924 | 0.172 | 19.232 | 178185.202 | 0.019 | 417.005 | 2.631 | 47.336 | 0.007 | 34.277 | 33.782 | 48.539 | 153.982 | 5.004 | 316.871 |
| 0 | test | 17.61 | 17.44 | 17.55 | 0.008 | 45.57 | 82.965 | 0.544 | 9.242 | 318029.983 | 0.006 | 227.803 | 2.614 | 913.516 | 0.004 | 42.697 | 43.456 | 58.906 | 181.778 | 5.278 | 284.646 |
| 1 | train | 14.395 | 13.386 | 14.376 | 0.008 | 42.717 | 74.417 | 1.006 | 5.341 | 510578.471 | 0.008 | 398.307 | 2.736 | 1013.137 | 0.003 | 36.799 | 37.476 | 51.175 | 165.005 | 4.939 | 294.237 |
| 0 | train | 15.994 | 14.98 | 15.965 | 0.007 | 34.764 | 81.356 | 0.522 | 9.28 | 436521.166 | 0.008 | 339.437 | 2.56 | 1060.941 | 0.003 | 39.651 | 40.408 | 55.04 | 173.965 | 5.02 | 290.594 |
| 1 | train | 15.047 | 13.577 | 15.038 | 0.007 | 38.696 | 75.555 | 0.927 | 5.535 | 342803.218 | 0.006 | 295.894 | 2.598 | 896.086 | 0.002 | 38.198 | 39.001 | 52.974 | 169.154 | 4.767 | 290.079 |
| 0 | test | 17.138 | 17.492 | 17.095 | 0.008 | 34.032 | 80.805 | 1.033 | 5.4 | 357496.276 | 0.004 | 201.621 | 2.73 | 938.926 | 0.004 | 42.787 | 43.702 | 59.182 | 182.052 | 5.327 | 274.526 |
| 2 | train | 9.03 | 9.554 | 8.946 | 0.007 | 45.123 | 72.716 | 0.537 | 9.068 | 437795.324 | 0.007 | 517.101 | 2.895 | 945.055 | 0.003 | 25.919 | 26.557 | 36.73 | 131.842 | 4.801 | 273.256 |
| 1 | train | 17.15 | 15.664 | 17.09 | 0.009 | 42.903 | 82.064 | 0.883 | 5.748 | 395550.319 | 0.01 | 332.961 | 2.732 | 997.009 | 0.004 | 41.153 | 41.731 | 57.027 | 177.687 | 5.175 | 284.691 |
| 1 | test | 14.333 | 14.468 | 14.353 | 0.007 | 49.349 | 72.911 | 1.022 | 5.456 | 361623.722 | 0.006 | 312.178 | 2.724 | 974.008 | 0.002 | 39.189 | 40.106 | 54.32 | 172.144 | 4.986 | 268.153 |
| 0 | test | 13.594 | 12.808 | 13.616 | 0.007 | 49.883 | 66.357 | 0.99 | 5.408 | 289829.011 | 0.005 | 294.902 | 2.319 | 833.808 | 0.003 | 37.229 | 38.156 | 51.606 | 166.14 | 4.801 | 273.772 |
| 0 | train | 14.827 | 14.44 | 14.828 | 0.007 | 45.231 | 73.801 | 1.124 | 5.156 | 490876.53 | 0.003 | 246.757 | 2.71 | 980.615 | 0.003 | 39.356 | 40.263 | 54.522 | 172.942 | 5.238 | 286.228 |
| 0 | train | 21.748 | 19.227 | 21.61 | 0.011 | 24.226 | 96.425 | 0.499 | 10.093 | 589596.315 | 0.014 | 270.687 | 2.531 | 223.036 | 0.005 | 46.605 | 46.68 | 64.48 | 192.402 | 5.594 | 313.981 |
| 1 | test | 15.216 | 14.486 | 15.204 | 0.007 | 42.383 | 74.778 | 0.992 | 5.499 | 436944.939 | 0.008 | 246.603 | 2.662 | 976.743 | 0.002 | 39.071 | 39.905 | 54.176 | 171.736 | 5.053 | 284.959 |
| 1 | train | 15.422 | 14.558 | 15.368 | 0.008 | 40.231 | 93.315 | 0.881 | 5.757 | 464670.622 | 0.008 | 282.579 | 2.63 | 923.148 | 0.003 | 37.676 | 38.293 | 52.418 | 167.41 | 5.106 | 294.644 |
| 0 | train | 22.817 | 21.094 | 22.672 | 0.013 | 30.863 | 118.714 | 0.163 | 19.774 | 295647.682 | 0.023 | 286.068 | 2.462 | 177.933 | 0.007 | 47.578 | 47.041 | 65.857 | 194.169 | 5.221 | 322.002 |
| 1 | train | 19.364 | 20.788 | 19.239 | 0.013 | 42.68 | 113.836 | 0.164 | 19.725 | 241970.552 | 0.025 | 348.219 | 2.502 | 83.804 | 0.007 | 42.053 | 41.473 | 58.684 | 178.45 | 5.184 | 319.129 |
| 0 | test | 21.931 | 20.596 | 21.79 | 0.011 | 27.306 | 113.176 | 0.194 | 18.755 | 455183.166 | 0.019 | 308.232 | 2.58 | 75.756 | 0.005 | 46.238 | 45.994 | 64.04 | 191.076 | 5.311 | 321.321 |
| 1 | train | 16.322 | 15.326 | 16.279 | 0.008 | 36.747 | 84.435 | 0.519 | 9.388 | 445443.053 | 0.008 | 292.107 | 2.563 | 1186.155 | 0.003 | 39.646 | 40.327 | 54.967 | 173.781 | 4.928 | 294.601 |
| 0 | test | 13.726 | 13.949 | 13.757 | 0.007 | 35.368 | 74.527 | 1.036 | 5.459 | 385187.568 | 0.004 | 223.178 | 2.846 | 1072.427 | 0.002 | 38.461 | 39.431 | 53.172 | 169.987 | 4.981 | 284.954 |
| 0 | train | 15.332 | 14.031 | 15.329 | 0.007 | 36.345 | 76.344 | 0.576 | 9.039 | 373610.838 | 0.006 | 234.117 | 2.648 | 1068.862 | 0.003 | 40.378 | 41.284 | 55.84 | 175.382 | 4.968 | 287.734 |
| 1 | test | 14.795 | 13.523 | 14.792 | 0.007 | 46.654 | 84.18 | 1.081 | 5.311 | 296625.676 | 0.006 | 172.122 | 2.867 | 804.029 | 0.002 | 39.38 | 40.228 | 54.531 | 172.322 | 4.99 | 275.932 |
| 0 | train | 16.177 | 16.193 | 16.155 | 0.007 | 35.451 | 64.494 | 0.603 | 9.065 | 310465.196 | 0.003 | 136.592 | 2.726 | 791.345 | 0.002 | 43.918 | 44.823 | 60.633 | 185.009 | 5.097 | 277.065 |
| 0 | test | 15.315 | 15.23 | 15.319 | 0.006 | 42.771 | 62.413 | 1.037 | 5.415 | 573942.687 | 0.004 | 291.224 | 2.778 | 1155.782 | 0.002 | 41.239 | 42.211 | 56.884 | 177.982 | 4.976 | 289.291 |
| 1 | test | 14.956 | 13.577 | 14.962 | 0.007 | 45.152 | 67.748 | 1.016 | 5.398 | 298490.861 | 0.005 | 219.573 | 2.633 | 884.56 | 0.003 | 40 | 40.85 | 55.367 | 173.67 | 5.073 | 283.543 |
| 2 | train | 6.298 | 7.279 | 6.207 | 0.006 | 27.595 | 63.544 | 0.988 | 5.358 | 612251.341 | 0.004 | 532.824 | 2.719 | 421.102 | 0.002 | 20.758 | 21.405 | 29.949 | 114.808 | 4.781 | 255.989 |
| 0 | train | 14.445 | 14.332 | 14.466 | 0.006 | 41.949 | 78.077 | 1.04 | 5.452 | 392663.065 | 0.003 | 202.889 | 2.809 | 970.061 | 0.002 | 39.741 | 40.654 | 55.023 | 173.743 | 5.058 | 278.422 |
| 0 | test | 15.334 | 14.589 | 15.331 | 0.007 | 40.524 | 74.385 | 0.989 | 5.495 | 387437.977 | 0.004 | 355.175 | 2.656 | 890.671 | 0.003 | 40.255 | 41.118 | 55.79 | 174.822 | 5.133 | 273.814 |
| 1 | train | 12.541 | 12.807 | 12.523 | 0.007 | 35.747 | 75.376 | 0.559 | 9.082 | 511705.257 | 0.004 | 334.227 | 2.772 | 1049.837 | 0.003 | 34.59 | 35.311 | 48.231 | 158.739 | 4.772 | 270.902 |
| 1 | train | 20.634 | 20.149 | 20.501 | 0.012 | 28.229 | 109.609 | 0.296 | 12.514 | 265136.661 | 0.023 | 230.613 | 2.466 | 221.971 | 0.007 | 44.136 | 43.854 | 61.233 | 184.782 | 5.117 | 317.683 |
| 1 | test | 14.316 | 13.481 | 14.333 | 0.007 | 43.02 | 66.111 | 0.568 | 9.056 | 374440.815 | 0.004 | 247.266 | 2.573 | 968.162 | 0.002 | 38.904 | 39.777 | 53.97 | 170.737 | 4.888 | 287.385 |
| 0 | train | 16.659 | 16.088 | 16.624 | 0.008 | 34.662 | 77.632 | 0.608 | 8.702 | 387125.126 | 0.005 | 218.49 | 2.638 | 999.143 | 0.004 | 41.797 | 42.58 | 57.749 | 179.032 | 5.29 | 298.047 |
| 1 | train | 14.339 | 13.536 | 14.342 | 0.007 | 45.829 | 76.073 | 0.986 | 5.493 | 349630.028 | 0.006 | 196.467 | 2.813 | 834.7 | 0.002 | 38.201 | 39.063 | 53.096 | 169.126 | 4.722 | 273.5 |
| 0 | train | 15.484 | 14.933 | 15.484 | 0.007 | 39.388 | 62.585 | 0.597 | 9.042 | 346834.94 | 0.003 | 201.989 | 2.718 | 924.594 | 0.002 | 41.882 | 42.782 | 57.935 | 179.282 | 5.053 | 274.895 |
| 1 | train | 13.956 | 13.184 | 13.954 | 0.007 | 43.508 | 77.992 | 0.953 | 5.487 | 328218.177 | 0.006 | 274.182 | 2.599 | 900.208 | 0.002 | 37.068 | 37.802 | 51.591 | 165.81 | 4.79 | 268.794 |
| 1 | train | 14.01 | 13.222 | 13.997 | 0.008 | 38.759 | 78.091 | 0.925 | 5.58 | 361985.083 | 0.008 | 310.021 | 2.649 | 940.544 | 0.003 | 36.647 | 37.376 | 51.13 | 164.517 | 4.796 | 269.602 |
| 2 | train | 9.421 | 9.2 | 9.336 | 0.006 | 41.052 | 66.425 | 0.953 | 5.416 | 585488.01 | 0.006 | 466.496 | 2.832 | 1170.354 | 0.002 | 27.941 | 28.671 | 39.278 | 139.148 | 4.514 | 280.865 |
| 1 | train | 15.965 | 15.5 | 15.949 | 0.007 | 42.962 | 73.206 | 0.558 | 9.107 | 294435.117 | 0.006 | 207.287 | 2.497 | 894.605 | 0.003 | 41.037 | 41.875 | 56.812 | 177.192 | 5.12 | 287.586 |
| 1 | test | 13.86 | 13.039 | 13.88 | 0.007 | 41.636 | 68.315 | 0.559 | 9.085 | 313891.19 | 0.006 | 250.069 | 2.561 | 844.843 | 0.002 | 37.392 | 38.248 | 51.78 | 166.499 | 4.728 | 256.986 |
| 1 | train | 14.876 | 13.329 | 14.883 | 0.007 | 39.617 | 62.978 | 0.59 | 9.058 | 319391.782 | 0.006 | 235.619 | 2.529 | 904.01 | 0.002 | 40.669 | 41.553 | 56.297 | 175.965 | 5.059 | 271.879 |
| 2 | train | 15.512 | 20.002 | 15.403 | 0.013 | 30.541 | 106.831 | 0.158 | 19.734 | 215824.342 | 0.026 | 454.487 | 2.55 | 32.728 | 0.007 | 35.679 | 34.967 | 50.526 | 157.801 | 5.012 | 315.09 |
| 1 | test | 12.636 | 12.951 | 12.603 | 0.008 | 33.196 | 73.416 | 0.943 | 5.596 | 302938.636 | 0.007 | 163.827 | 2.844 | 627.818 | 0.004 | 33.492 | 34.325 | 46.671 | 154.748 | 4.789 | 267.21 |
| 2 | train | 12.378 | 12.498 | 12.317 | 0.008 | 41.018 | 78.574 | 0.519 | 9.355 | 431824.444 | 0.013 | 451.63 | 2.743 | 878.33 | 0.004 | 32.135 | 32.76 | 45.14 | 151.185 | 5.043 | 290.438 |
| 1 | train | 14.3 | 13.334 | 14.312 | 0.007 | 38.316 | 74.912 | 0.953 | 5.527 | 278773.938 | 0.005 | 216.293 | 2.713 | 780.961 | 0.002 | 38.01 | 38.785 | 52.797 | 168.458 | 4.619 | 267.918 |
| 1 | train | 13.693 | 12.816 | 13.733 | 0.006 | 36.528 | 61.182 | 1.233 | 4.994 | 443522.893 | 0.003 | 251.204 | 2.736 | 1003.233 | 0.002 | 39.274 | 40.264 | 54.464 | 172.534 | 4.995 | 277.59 |
| 0 | train | 15.157 | 14.478 | 15.159 | 0.006 | 52.355 | 74.575 | 1.006 | 5.432 | 397642.988 | 0.003 | 288.433 | 2.545 | 985.371 | 0.002 | 40.435 | 41.404 | 56.076 | 175.838 | 5.019 | 265.313 |
| 1 | test | 20.433 | 19.111 | 20.303 | 0.009 | 32.104 | 107.404 | 0.383 | 11.236 | 363740.383 | 0.014 | 221.409 | 2.68 | 96.75 | 0.005 | 44.205 | 44.576 | 61.085 | 186.332 | 5.057 | 302.578 |
| 0 | train | 22.984 | 20.741 | 22.836 | 0.013 | 29.822 | 113.484 | 0.173 | 19.35 | 342857.293 | 0.021 | 302.017 | 2.427 | 186.431 | 0.006 | 47.911 | 47.379 | 66.26 | 195.457 | 5.229 | 313.817 |
| 0 | train | 14.768 | 14.075 | 14.773 | 0.007 | 34.966 | 73.871 | 0.971 | 5.507 | 423020.066 | 0.005 | 281.636 | 2.596 | 1037.085 | 0.003 | 39.954 | 40.718 | 55.379 | 174.247 | 4.98 | 269.944 |
| 2 | train | 11.188 | 11.785 | 11.103 | 0.008 | 32.2 | 81.239 | 0.492 | 9.582 | 549233.149 | 0.008 | 598.467 | 2.92 | 929.613 | 0.004 | 29.383 | 29.797 | 41.465 | 143.106 | 4.819 | 278.048 |
| 0 | train | 15.391 | 14.339 | 15.397 | 0.007 | 36.738 | 78.152 | 0.575 | 9.04 | 359179.409 | 0.005 | 266.071 | 2.548 | 863.027 | 0.003 | 40.908 | 41.772 | 56.711 | 176.397 | 5.067 | 273.691 |
| 1 | train | 13.738 | 13.069 | 13.715 | 0.008 | 36.23 | 81.558 | 0.524 | 9.295 | 428976.827 | 0.005 | 306.647 | 2.724 | 975.231 | 0.003 | 35.667 | 36.37 | 49.587 | 161.898 | 4.618 | 288.281 |
| 0 | train | 20.097 | 20.024 | 19.971 | 0.011 | 38.436 | 107.742 | 0.31 | 12.307 | 343700.587 | 0.021 | 287.902 | 2.52 | 184.291 | 0.006 | 43.452 | 43.28 | 60.428 | 183.456 | 5.096 | 311.503 |
| 0 | train | 12.858 | 13.561 | 12.897 | 0.006 | 47.136 | 68.482 | 1.041 | 5.425 | 396116.598 | 0.006 | 347.825 | 2.749 | 947.133 | 0.002 | 37.016 | 37.923 | 51.423 | 166.035 | 4.851 | 262.948 |
| 0 | train | 15.293 | 14.314 | 15.297 | 0.007 | 46.65 | 63.121 | 1.024 | 5.501 | 367688.729 | 0.002 | 225.636 | 2.817 | 859.154 | 0.003 | 41.178 | 42.067 | 56.97 | 177.279 | 5.059 | 283.259 |
| 1 | train | 14.416 | 13.391 | 14.405 | 0.007 | 46.675 | 81.297 | 0.949 | 5.451 | 372707.548 | 0.007 | 362.15 | 2.597 | 1028.733 | 0.003 | 37.38 | 38.222 | 52.045 | 167.409 | 4.923 | 264.667 |
| 1 | train | 13.157 | 13.741 | 13.207 | 0.006 | 56.491 | 64.371 | 1.004 | 5.417 | 393938.889 | 0.003 | 256.102 | 2.878 | 870.763 | 0.002 | 37.742 | 38.687 | 52.337 | 168.181 | 4.493 | 293.472 |
| 1 | train | 14.349 | 13.357 | 14.341 | 0.007 | 42.705 | 75.974 | 0.97 | 5.517 | 407499.395 | 0.004 | 289.22 | 2.724 | 1032.548 | 0.002 | 37.987 | 38.762 | 52.931 | 168.833 | 4.916 | 273.28 |
| 1 | test | 13.394 | 12.961 | 13.375 | 0.007 | 39.125 | 69.248 | 0.564 | 9.082 | 334629.781 | 0.004 | 256.275 | 2.559 | 860.281 | 0.003 | 34.953 | 35.764 | 48.614 | 159.251 | 4.754 | 269.351 |
| 1 | train | 15.259 | 14.337 | 15.248 | 0.008 | 42.695 | 75.921 | 0.946 | 5.523 | 456414.718 | 0.006 | 424.578 | 2.698 | 1120.53 | 0.003 | 39.478 | 40.31 | 54.717 | 173.064 | 5.054 | 277.629 |
| 0 | train | 14.057 | 13.769 | 14.08 | 0.006 | 45.198 | 66.542 | 0.985 | 5.501 | 374751.198 | 0.004 | 261.783 | 2.602 | 956.623 | 0.002 | 38.841 | 39.687 | 53.823 | 171.11 | 4.808 | 258.806 |
| 1 | train | 14.856 | 13.309 | 14.846 | 0.008 | 40.206 | 75.279 | 0.965 | 5.492 | 352258.882 | 0.008 | 305.546 | 2.614 | 979.683 | 0.003 | 38.553 | 39.291 | 53.497 | 169.804 | 4.992 | 271.43 |
| 1 | train | 14.467 | 13.355 | 14.477 | 0.007 | 45.575 | 72.489 | 0.367 | 13.626 | 555157.429 | 0.005 | 268.444 | 2.725 | 1165.664 | 0.002 | 38.5 | 39.382 | 53.483 | 170.252 | 4.696 | 292.234 |
| 1 | train | 11.73 | 12.474 | 11.702 | 0.007 | 43.732 | 64.702 | 0.554 | 8.983 | 401566.14 | 0.003 | 311.037 | 2.679 | 941.236 | 0.003 | 33.349 | 34.199 | 46.786 | 155.76 | 5.078 | 247.872 |
| 0 | train | 14.548 | 13.77 | 14.551 | 0.007 | 41.232 | 73.733 | 0.557 | 9.076 | 394987.204 | 0.007 | 218.819 | 2.675 | 948.306 | 0.002 | 38.735 | 39.566 | 53.768 | 170.448 | 4.878 | 304.381 |
| 0 | test | 16.132 | 15.437 | 16.104 | 0.008 | 44.106 | 75.599 | 0.559 | 9.116 | 375881.155 | 0.004 | 291.022 | 2.683 | 964.364 | 0.003 | 40.893 | 41.675 | 56.662 | 176.507 | 5.162 | 275.889 |
| 0 | train | 15.512 | 15.698 | 15.501 | 0.007 | 44.666 | 77.673 | 0.997 | 5.555 | 383560.385 | 0.004 | 251.049 | 2.736 | 957.063 | 0.003 | 40.679 | 41.489 | 56.309 | 176.212 | 5.081 | 279.341 |
| 1 | test | 13.283 | 13.488 | 13.266 | 0.007 | 33.726 | 77.257 | 0.545 | 9.099 | 561239.46 | 0.006 | 342.08 | 2.739 | 1121.053 | 0.003 | 35.573 | 36.35 | 49.615 | 161.76 | 4.968 | 268.15 |
| 1 | train | 14.548 | 13.045 | 14.568 | 0.007 | 37.669 | 67.119 | 1.029 | 5.53 | 261745.741 | 0.003 | 174.746 | 2.785 | 780.086 | 0.002 | 40.199 | 41.046 | 55.704 | 174.651 | 4.913 | 265.348 |
| 0 | train | 16.483 | 15.672 | 16.459 | 0.008 | 42.885 | 84.319 | 0.549 | 9.212 | 312919.889 | 0.005 | 242.345 | 2.67 | 963.507 | 0.004 | 41.574 | 42.331 | 57.469 | 178.459 | 5.12 | 280.796 |
| 1 | train | 13.905 | 13.332 | 13.905 | 0.007 | 38.756 | 75.364 | 0.932 | 5.556 | 332228.172 | 0.005 | 191.493 | 2.731 | 930.038 | 0.003 | 36.746 | 37.504 | 51.078 | 165.087 | 4.727 | 283.883 |
| 1 | train | 13.369 | 13.488 | 13.348 | 0.007 | 47.643 | 72.811 | 0.546 | 9.076 | 456916.092 | 0.006 | 313.793 | 2.599 | 1148.873 | 0.003 | 35.313 | 36.067 | 49.246 | 161.227 | 4.829 | 286.581 |
| 1 | train | 14.515 | 13.41 | 14.53 | 0.007 | 42.797 | 78.278 | 0.988 | 5.573 | 300968.141 | 0.006 | 181.308 | 2.747 | 823.498 | 0.003 | 38.705 | 39.505 | 53.661 | 169.883 | 4.829 | 289.646 |
| 1 | train | 14.482 | 13.511 | 14.498 | 0.006 | 44.942 | 80.397 | 0.603 | 8.74 | 444072.186 | 0.003 | 318.884 | 2.677 | 1045.194 | 0.002 | 38.823 | 39.831 | 53.783 | 171.63 | 4.842 | 267.261 |
| 1 | test | 12.393 | 12.95 | 12.348 | 0.007 | 38.828 | 77.997 | 0.541 | 9.164 | 443564.065 | 0.004 | 363.225 | 2.503 | 920.179 | 0.004 | 33.199 | 33.948 | 46.385 | 154.375 | 4.843 | 262.786 |
| 0 | train | 23.268 | 21.171 | 23.119 | 0.013 | 30.152 | 116.755 | 0.171 | 19.435 | 271322.386 | 0.025 | 254.589 | 2.433 | 200.001 | 0.006 | 48.684 | 48.136 | 67.362 | 197.225 | 5.327 | 313.813 |
| 1 | test | 21.497 | 20.194 | 21.358 | 0.011 | 23.105 | 113.375 | 0.339 | 11.903 | 329252.129 | 0.017 | 200.96 | 2.525 | 209.589 | 0.006 | 45.881 | 45.887 | 63.409 | 189.813 | 5.176 | 315.152 |
| 1 | train | 22.466 | 21.007 | 22.322 | 0.014 | 33.794 | 120.441 | 0.165 | 19.63 | 282898.446 | 0.02 | 333.192 | 2.449 | 172.317 | 0.007 | 47.119 | 46.516 | 65.245 | 192.592 | 5.153 | 316.563 |
| 2 | test | 12.933 | 18.659 | 12.83 | 0.013 | 28.909 | 96.878 | 0.17 | 19.114 | 170887.636 | 0.023 | 449.913 | 2.727 | 25.43 | 0.007 | 31.16 | 30.631 | 44.405 | 143.215 | 4.783 | 302.932 |
| 1 | train | 19.041 | 19.012 | 18.925 | 0.011 | 36.843 | 103.594 | 0.33 | 12.035 | 330201.476 | 0.018 | 238.314 | 2.531 | 126.484 | 0.005 | 41.856 | 41.759 | 58.331 | 179.108 | 5.172 | 310.415 |
| 1 | train | 18.086 | 19.877 | 17.97 | 0.012 | 32.873 | 103.573 | 0.284 | 12.684 | 255562.006 | 0.02 | 315.967 | 2.538 | 187.871 | 0.006 | 40.407 | 39.978 | 56.582 | 173.965 | 5.2 | 307.738 |
| 1 | train | 13.631 | 13.422 | 13.624 | 0.007 | 41.491 | 76.125 | 0.98 | 5.443 | 458713.273 | 0.008 | 283.464 | 2.579 | 1070.879 | 0.003 | 36.863 | 37.643 | 51.359 | 165.462 | 5.007 | 285.024 |
| 0 | test | 22.171 | 20.823 | 22.028 | 0.012 | 36.911 | 112.176 | 0.185 | 18.913 | 287118.494 | 0.022 | 215.987 | 2.492 | 88.118 | 0.006 | 46.393 | 46.195 | 64.188 | 191.322 | 5.223 | 311.064 |
| 1 | test | 23.151 | 21.044 | 23.004 | 0.013 | 36.265 | 122.117 | 0.17 | 19.534 | 208383.518 | 0.021 | 188.457 | 2.394 | 167.12 | 0.006 | 47.876 | 47.457 | 66.111 | 194.737 | 5.281 | 319.086 |
| 1 | train | 18.895 | 19.764 | 18.772 | 0.012 | 34.16 | 106.711 | 0.289 | 12.617 | 293371.818 | 0.022 | 286.07 | 2.592 | 174.428 | 0.006 | 41.198 | 40.913 | 57.623 | 176.715 | 5.164 | 311.322 |
| 0 | test | 20.524 | 19.914 | 20.396 | 0.012 | 27.845 | 112.732 | 0.206 | 18.009 | 238074.412 | 0.018 | 177.683 | 2.501 | 388.368 | 0.007 | 44.353 | 44.144 | 61.54 | 185.692 | 5.133 | 309.134 |
| 0 | train | 20.606 | 20.218 | 20.478 | 0.012 | 31.627 | 106.419 | 0.301 | 12.487 | 315208.822 | 0.021 | 271.033 | 2.588 | 213.206 | 0.006 | 44.402 | 44.245 | 61.761 | 185.535 | 5.317 | 319.527 |
| 0 | test | 22.372 | 21.021 | 22.234 | 0.013 | 44.188 | 115.457 | 0.164 | 19.693 | 309481.409 | 0.023 | 367.86 | 2.417 | 223.828 | 0.007 | 46.785 | 46.282 | 64.81 | 192.575 | 5.147 | 312.249 |
| 1 | train | 13.682 | 13.365 | 13.723 | 0.007 | 42.147 | 61.777 | 1.04 | 5.441 | 254532.671 | 0.004 | 193.268 | 2.697 | 805.293 | 0.003 | 39.028 | 39.874 | 54.041 | 170.855 | 4.931 | 256.765 |
| 1 | test | 19.927 | 20.634 | 19.799 | 0.013 | 30.881 | 112.314 | 0.167 | 19.544 | 252020.37 | 0.024 | 257.499 | 2.514 | 95.655 | 0.007 | 42.787 | 42.322 | 59.634 | 180.734 | 5.075 | 313.748 |
| 0 | train | 21.539 | 20.35 | 21.399 | 0.012 | 33.288 | 111.197 | 0.307 | 12.338 | 263038.981 | 0.018 | 231.625 | 2.358 | 237.259 | 0.006 | 45.514 | 45.471 | 63.165 | 189.342 | 5.166 | 319.459 |
| 1 | test | 20.612 | 19.996 | 20.482 | 0.012 | 27.223 | 110.267 | 0.197 | 18.376 | 241280.612 | 0.017 | 193.06 | 2.557 | 151.777 | 0.006 | 44.323 | 44.049 | 61.603 | 185.594 | 5.225 | 310.169 |
| 1 | train | 23.264 | 21.084 | 23.116 | 0.013 | 20.475 | 116.721 | 0.171 | 19.528 | 261160.085 | 0.028 | 200.934 | 2.424 | 218.64 | 0.006 | 48.342 | 47.858 | 66.879 | 196.357 | 5.345 | 314.494 |
| 0 | test | 23.868 | 20.915 | 23.72 | 0.014 | 34.153 | 117.201 | 0.153 | 20.297 | 420006.223 | 0.027 | 390.686 | 2.403 | 124.458 | 0.006 | 48.987 | 48.343 | 67.619 | 198.447 | 5.214 | 319.274 |
| 1 | test | 15.011 | 13.808 | 15.009 | 0.007 | 37.933 | 78.454 | 0.971 | 5.5 | 299936.385 | 0.004 | 225.693 | 2.725 | 829.212 | 0.002 | 39.249 | 40.063 | 54.366 | 171.886 | 4.808 | 270.67 |
| 0 | train | 15.203 | 13.505 | 15.2 | 0.007 | 45.415 | 70.873 | 0.587 | 9.099 | 315188.856 | 0.004 | 171.934 | 2.696 | 900.852 | 0.003 | 40.872 | 41.784 | 56.571 | 176.391 | 5.125 | 287.263 |
| 1 | train | 12.487 | 12.458 | 12.44 | 0.008 | 42.226 | 76.739 | 0.924 | 5.595 | 340782.153 | 0.007 | 306.464 | 2.647 | 913.953 | 0.004 | 33.068 | 33.759 | 46.189 | 153.678 | 4.979 | 264.309 |
| 1 | train | 14.142 | 13.556 | 14.153 | 0.006 | 49.311 | 65.126 | 1.002 | 5.466 | 384214.696 | 0.004 | 270.629 | 2.805 | 995.848 | 0.002 | 38.78 | 39.604 | 53.742 | 170.984 | 4.82 | 274.729 |
| 1 | test | 15.513 | 14.86 | 15.5 | 0.007 | 37.974 | 78.274 | 0.999 | 5.484 | 393257.189 | 0.006 | 211.909 | 2.603 | 1003.715 | 0.003 | 40.887 | 41.763 | 56.597 | 176.878 | 5.098 | 285.711 |
| 0 | test | 15.777 | 15.035 | 15.759 | 0.007 | 39.746 | 70.57 | 0.582 | 9.002 | 340577.011 | 0.005 | 207.394 | 2.591 | 874.161 | 0.003 | 41.511 | 42.485 | 57.425 | 178.532 | 4.97 | 295.108 |
| 1 | test | 14.874 | 13.898 | 14.868 | 0.007 | 40.768 | 74.484 | 0.955 | 5.522 | 306054.526 | 0.005 | 252.582 | 2.693 | 771.66 | 0.003 | 38.395 | 39.261 | 53.402 | 170.318 | 4.832 | 274.703 |
| 1 | test | 14.13 | 13.262 | 14.149 | 0.007 | 37.324 | 70.752 | 0.371 | 13.606 | 397649.772 | 0.004 | 240.88 | 2.793 | 1020.983 | 0.002 | 38.225 | 39.113 | 52.953 | 169.248 | 4.767 | 287.957 |
| 1 | test | 13.071 | 13.263 | 13.117 | 0.006 | 50.181 | 60.701 | 1.073 | 5.487 | 333246.409 | 0.005 | 213.682 | 2.816 | 774.473 | 0.002 | 37.977 | 38.942 | 52.635 | 167.98 | 4.878 | 288.401 |
| 1 | train | 14.715 | 13.824 | 14.719 | 0.007 | 40.573 | 68.401 | 1.025 | 5.497 | 431831.092 | 0.005 | 238.173 | 2.736 | 932.865 | 0.002 | 40.055 | 40.951 | 55.484 | 174.151 | 5.018 | 286.914 |
| 1 | train | 14.109 | 14.003 | 14.138 | 0.006 | 48.016 | 71.978 | 1.005 | 5.432 | 416256.404 | 0.005 | 333.696 | 2.705 | 1048.953 | 0.002 | 38.964 | 39.891 | 54.014 | 171.822 | 4.791 | 260.975 |
| 1 | train | 16.338 | 16.2 | 16.31 | 0.007 | 36.581 | 86.871 | 0.955 | 5.503 | 521178.846 | 0.007 | 332.109 | 2.606 | 1105.46 | 0.003 | 40.824 | 41.768 | 56.51 | 177.469 | 5.089 | 286.427 |
| 2 | train | 10.526 | 10.189 | 10.455 | 0.007 | 34.575 | 66.721 | 0.947 | 5.477 | 539879.06 | 0.003 | 408.861 | 2.705 | 1078.06 | 0.003 | 30.255 | 31.025 | 42.556 | 146.061 | 4.895 | 265.339 |
| 2 | train | 10.79 | 10.989 | 10.709 | 0.007 | 36.74 | 74.162 | 0.909 | 5.52 | 471132.348 | 0.007 | 407.138 | 2.778 | 1002.607 | 0.003 | 29.511 | 30.177 | 41.583 | 143.697 | 4.898 | 263.679 |
| 1 | test | 14.638 | 14.35 | 14.656 | 0.007 | 41.919 | 59.099 | 1.055 | 5.433 | 293469.102 | 0.003 | 181.017 | 2.661 | 840.268 | 0.002 | 40.444 | 41.505 | 55.942 | 175.411 | 4.941 | 286.051 |
| 1 | train | 15.659 | 15.694 | 15.587 | 0.009 | 32.025 | 88.351 | 0.452 | 10.165 | 577014.366 | 0.01 | 377.363 | 2.588 | 617.124 | 0.004 | 37.498 | 37.982 | 52.385 | 167.76 | 5.077 | 297.904 |
| 1 | train | 14.822 | 13.885 | 14.795 | 0.008 | 36.826 | 89.553 | 0.518 | 9.392 | 372512.85 | 0.006 | 206.252 | 2.615 | 901.117 | 0.003 | 37.378 | 38.054 | 51.996 | 167.027 | 4.837 | 291.697 |
| 1 | train | 15.865 | 15.41 | 15.847 | 0.007 | 48.705 | 72.172 | 0.976 | 5.524 | 469250.806 | 0.005 | 302.21 | 2.768 | 1002.76 | 0.003 | 40.846 | 41.719 | 56.55 | 177.173 | 4.946 | 273.839 |
| 0 | train | 15.169 | 14.175 | 15.177 | 0.008 | 45.753 | 76.358 | 0.962 | 5.501 | 269633.764 | 0.003 | 191.295 | 2.594 | 861.321 | 0.003 | 39.771 | 40.621 | 54.969 | 173.167 | 4.837 | 278.058 |
| 0 | train | 21.568 | 20.079 | 21.429 | 0.011 | 47.988 | 106.217 | 0.334 | 11.868 | 297557.274 | 0.021 | 223.79 | 2.455 | 314.118 | 0.006 | 45.415 | 45.463 | 62.701 | 188.649 | 5.132 | 311.234 |
| 0 | test | 21.655 | 20.289 | 21.519 | 0.012 | 31.116 | 107.946 | 0.306 | 12.292 | 244948.799 | 0.021 | 185.942 | 2.489 | 394.578 | 0.006 | 45.329 | 45.31 | 62.6 | 188.058 | 5.205 | 307.465 |
| 0 | test | 15.204 | 14.436 | 15.175 | 0.007 | 39.019 | 84.308 | 0.523 | 9.273 | 494362.449 | 0.008 | 317.828 | 2.637 | 923.477 | 0.002 | 37.782 | 38.486 | 52.517 | 168.505 | 4.764 | 289.46 |
| 1 | test | 15.101 | 14.085 | 15.101 | 0.007 | 45.975 | 67.591 | 0.981 | 5.556 | 373545.711 | 0.006 | 251.59 | 2.742 | 884.865 | 0.003 | 39.348 | 40.167 | 54.361 | 171.739 | 4.925 | 281.377 |
| 1 | train | 15.776 | 14.1 | 15.761 | 0.008 | 31.95 | 79.033 | 0.54 | 9.233 | 294739.84 | 0.004 | 200.024 | 2.581 | 848.896 | 0.003 | 39.913 | 40.656 | 55.333 | 173.643 | 5.034 | 280.543 |
| 2 | train | 10.726 | 10.733 | 10.637 | 0.008 | 33.209 | 68.57 | 0.55 | 9.105 | 422454.447 | 0.004 | 306.97 | 2.849 | 643.44 | 0.004 | 30.015 | 30.705 | 42.083 | 144.492 | 4.963 | 293.645 |
| 1 | test | 14.593 | 13.742 | 14.587 | 0.007 | 47.415 | 80.773 | 0.539 | 9.178 | 336261.06 | 0.006 | 261.383 | 2.718 | 937.808 | 0.002 | 37.735 | 38.635 | 52.404 | 167.724 | 4.811 | 289.329 |
| 2 | train | 9.12 | 9.696 | 9.042 | 0.007 | 43.3 | 70.313 | 0.544 | 9.049 | 536994.547 | 0.007 | 596.281 | 2.87 | 1047.28 | 0.003 | 26.508 | 27.182 | 37.522 | 133.925 | 4.692 | 264.644 |
| 1 | train | 13.891 | 13.442 | 13.878 | 0.007 | 39.382 | 78.283 | 0.551 | 9.17 | 473963.76 | 0.008 | 344.083 | 2.698 | 1128.666 | 0.003 | 36.446 | 37.281 | 50.659 | 164.518 | 4.896 | 286.717 |
| 0 | test | 16.58 | 15.921 | 16.539 | 0.008 | 44.739 | 85.99 | 0.534 | 9.24 | 387329.404 | 0.006 | 294.783 | 2.524 | 1052.376 | 0.003 | 41.183 | 41.881 | 57.031 | 177.798 | 5.223 | 281.596 |
| 0 | test | 15.005 | 14.455 | 15.013 | 0.007 | 41.932 | 69.794 | 0.557 | 9.038 | 520051.815 | 0.005 | 288.455 | 2.645 | 1168.263 | 0.002 | 39.831 | 40.794 | 55.153 | 174.438 | 4.83 | 293.21 |
| 0 | train | 23.091 | 21.136 | 22.943 | 0.012 | 21.036 | 109.326 | 0.35 | 11.831 | 382990.661 | 0.017 | 233.932 | 2.422 | 153.841 | 0.006 | 48.295 | 48.067 | 66.794 | 196.794 | 5.44 | 314.912 |
| 0 | train | 15.719 | 16.144 | 15.706 | 0.006 | 48.066 | 64.469 | 1.075 | 5.443 | 397042.615 | 0.004 | 286.871 | 2.679 | 918.842 | 0.003 | 42.398 | 43.457 | 58.523 | 180.913 | 4.996 | 259.713 |
| 0 | train | 18.639 | 18.04 | 18.55 | 0.008 | 39.586 | 86.929 | 0.566 | 9.12 | 369827.443 | 0.005 | 219.222 | 2.526 | 949.482 | 0.004 | 45.665 | 46.633 | 62.797 | 190.42 | 5.034 | 287.925 |
| 0 | train | 15.525 | 15.256 | 15.525 | 0.007 | 46.601 | 71.57 | 1.047 | 5.462 | 357978.864 | 0.004 | 200.507 | 2.804 | 760.686 | 0.003 | 41.61 | 42.549 | 57.546 | 178.45 | 5.032 | 275.305 |
| 0 | train | 16.081 | 15.075 | 16.05 | 0.008 | 38.674 | 87.046 | 0.521 | 9.344 | 406136.568 | 0.005 | 264.327 | 2.632 | 1031.469 | 0.003 | 39.595 | 40.277 | 54.919 | 173.079 | 4.99 | 294.333 |
| 1 | test | 15.102 | 14.373 | 15.079 | 0.007 | 45.925 | 76.234 | 0.53 | 9.135 | 377744.917 | 0.006 | 301.934 | 2.539 | 992.556 | 0.003 | 38.421 | 39.168 | 53.496 | 170.004 | 4.874 | 290.077 |
| 2 | test | 10.586 | 11.046 | 10.502 | 0.007 | 38.387 | 80.136 | 0.892 | 5.612 | 413784.433 | 0.008 | 388.863 | 2.721 | 870.713 | 0.003 | 28.736 | 29.314 | 40.483 | 140.779 | 4.784 | 258.213 |
| 1 | test | 14.676 | 14.069 | 14.694 | 0.007 | 42.128 | 72.181 | 1.03 | 5.39 | 334135.278 | 0.003 | 290.478 | 2.537 | 967.528 | 0.002 | 40.193 | 41.155 | 55.571 | 174.978 | 4.925 | 257.523 |
| 0 | train | 15.321 | 14.708 | 15.322 | 0.006 | 53.136 | 67.754 | 1.004 | 5.448 | 372234.531 | 0.003 | 263.131 | 2.601 | 965.005 | 0.002 | 41.012 | 41.965 | 56.675 | 177.636 | 4.983 | 277.562 |
| 0 | train | 21.511 | 20.219 | 21.376 | 0.01 | 44.819 | 106.034 | 0.358 | 11.503 | 300731.632 | 0.016 | 185.691 | 2.545 | 369.788 | 0.006 | 45.728 | 45.774 | 63.244 | 189.27 | 4.983 | 308.813 |
| 1 | test | 16.513 | 15.693 | 16.479 | 0.008 | 38.349 | 81.829 | 0.544 | 9.133 | 424958.829 | 0.005 | 235.691 | 2.572 | 1067.445 | 0.003 | 40.523 | 41.356 | 56.091 | 176.141 | 5.079 | 301.884 |
| 1 | train | 13.523 | 13.028 | 13.511 | 0.007 | 36.764 | 80.643 | 0.546 | 9.138 | 483463.737 | 0.004 | 327.65 | 2.72 | 1151.525 | 0.003 | 35.621 | 36.434 | 49.578 | 162.283 | 4.814 | 273.45 |
| 0 | test | 15.077 | 14.473 | 15.06 | 0.007 | 40.664 | 81.092 | 0.956 | 5.527 | 405097.753 | 0.006 | 294.248 | 2.644 | 965.16 | 0.002 | 38.535 | 39.368 | 53.484 | 169.946 | 5.07 | 271.274 |
| 2 | train | 9.136 | 9.54 | 9.056 | 0.006 | 36.369 | 67.736 | 0.924 | 5.475 | 614751.337 | 0.008 | 679.812 | 2.883 | 471.277 | 0.002 | 26.337 | 27.003 | 37.286 | 134.152 | 4.633 | 252.664 |
| 1 | test | 14.272 | 13.04 | 14.295 | 0.007 | 42.572 | 71.532 | 0.981 | 5.508 | 318119.588 | 0.006 | 286.818 | 2.455 | 928.667 | 0.003 | 38.457 | 39.308 | 53.264 | 170.225 | 4.705 | 258.527 |
| 0 | train | 16.601 | 15.773 | 16.554 | 0.008 | 50.315 | 91.635 | 0.899 | 5.668 | 448859.731 | 0.006 | 304.32 | 2.6 | 754.488 | 0.003 | 40.003 | 40.802 | 55.547 | 174.89 | 4.941 | 293.264 |
| 1 | test | 15.765 | 14.526 | 15.752 | 0.008 | 40.802 | 71.093 | 0.946 | 5.551 | 310551.706 | 0.004 | 235.402 | 2.506 | 877.069 | 0.003 | 39.786 | 40.622 | 55.174 | 173.612 | 4.998 | 286.787 |
| 1 | train | 14.004 | 13.815 | 13.95 | 0.008 | 37.637 | 83.911 | 0.5 | 9.543 | 652124.999 | 0.009 | 579.182 | 2.727 | 1163.862 | 0.004 | 35.187 | 35.678 | 49.258 | 161.145 | 4.943 | 292.194 |
| 1 | test | 13.016 | 12.433 | 13.024 | 0.007 | 23.329 | 62.222 | 1.003 | 5.522 | 316860.333 | 0.006 | 173.098 | 2.763 | 907.485 | 0.003 | 36.201 | 36.951 | 50.364 | 163.26 | 4.887 | 272.038 |
| 0 | test | 13.772 | 13.626 | 13.803 | 0.007 | 32.303 | 68.427 | 1.003 | 5.443 | 373872.247 | 0.004 | 219.445 | 2.67 | 925.049 | 0.002 | 38.013 | 38.971 | 52.627 | 168.594 | 4.725 | 284.494 |
| 1 | train | 14.872 | 13.877 | 14.872 | 0.007 | 52.516 | 76.034 | 0.543 | 9.087 | 339208.725 | 0.008 | 283.369 | 2.602 | 874.776 | 0.002 | 38.496 | 39.379 | 53.467 | 169.76 | 4.761 | 282.871 |
| 1 | train | 15.2 | 13.866 | 15.179 | 0.007 | 33.224 | 91.362 | 0.901 | 5.632 | 324224.356 | 0.008 | 220.334 | 2.717 | 833.978 | 0.002 | 37.991 | 38.645 | 52.717 | 168.549 | 4.765 | 281.088 |
| 1 | test | 13.971 | 13.163 | 13.984 | 0.007 | 46.905 | 75.259 | 0.558 | 9.045 | 407979.561 | 0.005 | 369.455 | 2.477 | 1086.508 | 0.002 | 37.539 | 38.33 | 52.036 | 167.066 | 4.698 | 265.388 |
| 1 | train | 14.279 | 14.608 | 14.31 | 0.007 | 45.164 | 64.948 | 1.064 | 5.437 | 349033.589 | 0.003 | 233.392 | 2.802 | 907.68 | 0.002 | 39.81 | 40.815 | 54.961 | 173.318 | 5.031 | 284.411 |
| 0 | train | 15.237 | 14.272 | 15.228 | 0.007 | 51.619 | 66.156 | 0.593 | 8.919 | 291554.06 | 0.003 | 178.541 | 2.529 | 821.728 | 0.003 | 41.622 | 42.556 | 57.606 | 178.631 | 4.981 | 298.821 |
| 0 | test | 21.479 | 20.471 | 21.342 | 0.011 | 37.868 | 109.363 | 0.29 | 12.744 | 363614.994 | 0.024 | 313.006 | 2.439 | 124.935 | 0.006 | 45.878 | 45.577 | 63.675 | 190.718 | 5.291 | 312.112 |
| 1 | test | 15.365 | 14.559 | 15.355 | 0.007 | 35.591 | 75.847 | 0.57 | 9.121 | 376373.454 | 0.003 | 199.106 | 2.738 | 935.748 | 0.003 | 40.042 | 40.896 | 55.498 | 174.434 | 4.875 | 298.299 |
| 1 | train | 15.624 | 14.556 | 15.6 | 0.007 | 43.151 | 79.574 | 0.344 | 13.905 | 366855.53 | 0.006 | 298.048 | 2.604 | 794.567 | 0.002 | 39.143 | 39.853 | 54.4 | 171.812 | 4.893 | 280.904 |
| 1 | train | 14.004 | 13.46 | 13.996 | 0.006 | 47.024 | 79.683 | 0.551 | 9.157 | 314680.852 | 0.004 | 205.848 | 2.708 | 609.378 | 0.002 | 37.222 | 37.999 | 51.909 | 166.558 | 4.896 | 277.989 |
| 1 | train | 14.534 | 13.197 | 14.544 | 0.008 | 43.954 | 72.641 | 0.991 | 5.484 | 330628.885 | 0.003 | 227.048 | 2.709 | 887.289 | 0.003 | 39.673 | 40.539 | 54.961 | 172.96 | 5.014 | 261.451 |
| 1 | test | 14.376 | 13.839 | 14.397 | 0.007 | 39.45 | 63.684 | 0.983 | 5.431 | 491045.557 | 0.004 | 283.219 | 2.649 | 1130.014 | 0.002 | 39.049 | 39.926 | 54.056 | 171.742 | 4.859 | 264.719 |
| 1 | test | 15.291 | 15.247 | 15.212 | 0.009 | 44.036 | 98.401 | 0.438 | 10.274 | 594410.746 | 0.01 | 458.404 | 2.569 | 687.05 | 0.004 | 35.927 | 36.377 | 50.356 | 163.17 | 5.039 | 302.739 |
| 0 | train | 16.178 | 16.084 | 16.154 | 0.007 | 35.267 | 75.365 | 0.948 | 5.5 | 389733.289 | 0.004 | 304.21 | 2.606 | 1052.588 | 0.003 | 41.04 | 41.88 | 56.665 | 177.307 | 5.083 | 278.108 |
| 0 | test | 16.115 | 15.4 | 16.091 | 0.008 | 29.932 | 78.62 | 0.966 | 5.535 | 333974.889 | 0.004 | 202.99 | 2.709 | 929.786 | 0.003 | 41.525 | 42.313 | 57.516 | 178.077 | 5.208 | 268.993 |
| 0 | train | 16.708 | 16.014 | 16.672 | 0.008 | 43.118 | 78.252 | 0.542 | 9.253 | 452175.311 | 0.004 | 280.905 | 2.585 | 1119.405 | 0.004 | 41.666 | 42.493 | 57.552 | 179.238 | 5.18 | 293.164 |
| 1 | train | 16.599 | 15.553 | 16.564 | 0.008 | 42.734 | 82.128 | 0.557 | 9.102 | 288351.426 | 0.008 | 183.057 | 2.618 | 786.333 | 0.003 | 41.873 | 42.686 | 57.946 | 179.31 | 5.192 | 290.275 |
| 1 | train | 14.546 | 13.936 | 14.523 | 0.007 | 34.838 | 82.721 | 0.53 | 9.302 | 477196.542 | 0.007 | 380.441 | 2.651 | 1085.591 | 0.002 | 36.964 | 37.632 | 51.501 | 165.703 | 4.885 | 266.94 |
| 1 | test | 12.942 | 12.488 | 12.946 | 0.007 | 46.417 | 66.687 | 0.571 | 9.055 | 270511.663 | 0.007 | 206.973 | 2.837 | 806.616 | 0.003 | 35.655 | 36.441 | 49.67 | 161.494 | 4.732 | 273.377 |
| 0 | test | 16.201 | 15.778 | 16.185 | 0.007 | 48.072 | 81.937 | 0.979 | 5.499 | 284798.523 | 0.005 | 242.917 | 2.648 | 820.825 | 0.003 | 41.761 | 42.702 | 57.607 | 179.075 | 5.101 | 282.433 |
| 1 | test | 15.42 | 14.28 | 15.405 | 0.007 | 34.612 | 81.536 | 0.928 | 5.542 | 587611.05 | 0.008 | 415.069 | 2.662 | 1155.351 | 0.002 | 39.103 | 39.884 | 54.289 | 172.345 | 4.853 | 290.412 |
| 0 | train | 14.028 | 14.232 | 14.061 | 0.006 | 49.403 | 55.901 | 1.057 | 5.44 | 338256.821 | 0.002 | 224.858 | 2.629 | 902.858 | 0.002 | 39.548 | 40.525 | 54.736 | 172.989 | 4.928 | 272.247 |
| 0 | train | 22.005 | 20.739 | 21.867 | 0.011 | 31.594 | 109.101 | 0.335 | 12.055 | 550376.455 | 0.018 | 340.784 | 2.536 | 124.549 | 0.006 | 46.566 | 46.34 | 64.536 | 192.508 | 5.285 | 319.897 |
| 0 | train | 17.063 | 17.016 | 17.016 | 0.007 | 40.055 | 69.587 | 1.086 | 5.311 | 436758.329 | 0.003 | 233.71 | 2.55 | 1062.112 | 0.003 | 43.81 | 44.761 | 60.422 | 185.244 | 5.296 | 266.589 |
| 1 | test | 13.806 | 13.396 | 13.835 | 0.006 | 50.192 | 64.738 | 1.003 | 5.488 | 369235.166 | 0.003 | 249.807 | 2.459 | 855.543 | 0.002 | 39.227 | 40.116 | 54.454 | 172.012 | 4.834 | 260.955 |
| 0 | train | 16.285 | 16.086 | 16.265 | 0.008 | 47.657 | 72.114 | 0.567 | 9.082 | 410069.593 | 0.003 | 270.239 | 2.555 | 1039.595 | 0.004 | 41.491 | 42.308 | 57.311 | 178.155 | 5.113 | 284.369 |
| 0 | train | 24.568 | 20.722 | 24.417 | 0.014 | 40.631 | 131.238 | 0.147 | 20.611 | 285278.544 | 0.031 | 265.648 | 2.353 | 60.622 | 0.006 | 50.487 | 49.676 | 69.75 | 201.964 | 5.308 | 327.106 |
| 0 | train | 13.379 | 13.02 | 13.405 | 0.007 | 40.112 | 64.309 | 1.006 | 5.461 | 293411.359 | 0.004 | 205.985 | 2.674 | 830.893 | 0.003 | 37.97 | 38.858 | 52.766 | 168.068 | 5.035 | 269.135 |
| 1 | test | 13.126 | 12.977 | 13.104 | 0.008 | 33.747 | 73.568 | 0.952 | 5.492 | 413561.484 | 0.004 | 250.759 | 2.582 | 998.447 | 0.003 | 34.938 | 35.769 | 48.68 | 159.775 | 4.987 | 282.3 |
| 2 | train | 9.742 | 10.239 | 9.66 | 0.007 | 37.955 | 73.024 | 0.906 | 5.537 | 661593.876 | 0.008 | 635.444 | 2.933 | 1070.091 | 0.003 | 27.549 | 28.214 | 38.925 | 137.509 | 4.684 | 281.298 |
| 1 | train | 13.561 | 13.297 | 13.516 | 0.008 | 45.196 | 83.308 | 0.342 | 14.088 | 344301.936 | 0.006 | 307.104 | 2.552 | 743.744 | 0.004 | 34.668 | 35.243 | 48.532 | 158.87 | 4.948 | 283.261 |
| 1 | test | 16.545 | 15.907 | 16.485 | 0.008 | 30.352 | 88.99 | 0.495 | 9.679 | 507721.472 | 0.01 | 335.802 | 2.646 | 956.534 | 0.003 | 39.69 | 40.274 | 55.094 | 173.512 | 5.17 | 294.628 |
| 2 | test | 8.876 | 8.96 | 8.8 | 0.007 | 31.98 | 65.836 | 0.576 | 8.925 | 428795.06 | 0.005 | 488.805 | 2.693 | 984.189 | 0.002 | 27.392 | 28.186 | 38.504 | 136.527 | 4.666 | 252.25 |
| 1 | test | 14.276 | 13.543 | 14.281 | 0.006 | 42.3 | 71.928 | 0.978 | 5.493 | 398931.959 | 0.004 | 320.353 | 2.651 | 925.452 | 0.002 | 38.021 | 38.832 | 52.895 | 168.748 | 4.771 | 270.915 |
| 1 | train | 19.879 | 20.113 | 19.755 | 0.012 | 29.635 | 102.038 | 0.193 | 18.528 | 301199.742 | 0.018 | 272.146 | 2.574 | 322.388 | 0.007 | 43.167 | 42.804 | 60.015 | 182.188 | 5.081 | 305.425 |
| 2 | train | 8.867 | 9.567 | 8.784 | 0.007 | 42.625 | 79.123 | 0.962 | 5.428 | 512147.127 | 0.007 | 463.576 | 2.923 | 894.384 | 0.003 | 26.48 | 27.194 | 37.663 | 134.121 | 5.163 | 287.874 |
| 0 | train | 16.198 | 15.721 | 16.178 | 0.008 | 40.236 | 74.245 | 0.57 | 9.143 | 322728.27 | 0.005 | 237.111 | 2.706 | 920.425 | 0.004 | 41.071 | 41.882 | 56.794 | 176.707 | 5.268 | 291.729 |
| 0 | train | 15.725 | 14.235 | 15.703 | 0.009 | 35.944 | 77.605 | 0.528 | 9.342 | 280866.643 | 0.005 | 259.283 | 2.696 | 815.253 | 0.003 | 39.401 | 40.053 | 54.65 | 171.943 | 4.988 | 283.691 |
| 1 | test | 14.488 | 13.421 | 14.504 | 0.007 | 49.932 | 77.382 | 1.006 | 5.478 | 310165.776 | 0.003 | 193.7 | 2.792 | 858.63 | 0.002 | 38.735 | 39.548 | 53.779 | 170.084 | 4.893 | 294.445 |
| 1 | train | 12.903 | 13.138 | 12.883 | 0.007 | 39.641 | 79.492 | 0.546 | 9.092 | 513878.351 | 0.005 | 360.196 | 2.682 | 1204.778 | 0.003 | 34.954 | 35.757 | 48.814 | 160.567 | 4.91 | 266.652 |
| 0 | test | 15.521 | 15.415 | 15.51 | 0.006 | 49.973 | 71.998 | 0.969 | 5.456 | 563135.615 | 0.004 | 390.866 | 2.621 | 1102.237 | 0.002 | 39.901 | 40.819 | 55.299 | 175.041 | 4.982 | 276.586 |
| 1 | train | 13.99 | 13.849 | 14.011 | 0.006 | 43.645 | 62.998 | 1.03 | 5.448 | 366168.12 | 0.005 | 226.904 | 2.668 | 849.394 | 0.002 | 39.025 | 39.866 | 54.194 | 171.417 | 4.92 | 256.581 |
| 2 | train | 10.804 | 10.518 | 10.742 | 0.007 | 42.38 | 65.215 | 0.557 | 8.969 | 367732.583 | 0.008 | 220.352 | 2.806 | 936.498 | 0.003 | 30.863 | 31.71 | 43.386 | 147.76 | 4.998 | 308.217 |
| 1 | train | 14.147 | 14.055 | 14.142 | 0.007 | 39.735 | 80.808 | 0.954 | 5.475 | 458326.657 | 0.002 | 358.057 | 2.668 | 1086.831 | 0.002 | 37.102 | 38.028 | 51.575 | 166.927 | 4.838 | 276.307 |
| 1 | train | 12.633 | 13.383 | 12.581 | 0.007 | 31.659 | 74.639 | 0.534 | 9.106 | 593222.696 | 0.004 | 314.083 | 2.768 | 880.69 | 0.004 | 33.431 | 34.171 | 46.756 | 155.959 | 4.936 | 298.812 |
| 1 | test | 12.826 | 13.266 | 12.846 | 0.006 | 48.102 | 74.478 | 0.992 | 5.388 | 364030.889 | 0.004 | 388.638 | 2.635 | 987.717 | 0.002 | 36.048 | 36.935 | 50.201 | 163.336 | 4.743 | 248.556 |
| 1 | train | 13.509 | 13.312 | 13.504 | 0.007 | 49.084 | 75.934 | 0.99 | 5.48 | 313750.519 | 0.003 | 208.483 | 2.639 | 895.392 | 0.003 | 36.824 | 37.64 | 51.26 | 165.113 | 5.049 | 286.263 |
| 1 | train | 14.232 | 13.142 | 14.25 | 0.007 | 41.712 | 77.129 | 0.566 | 9.064 | 338722.987 | 0.004 | 256.541 | 2.574 | 983.93 | 0.002 | 38.102 | 38.933 | 52.857 | 168.731 | 4.736 | 269.751 |
| 1 | test | 13.896 | 12.849 | 13.886 | 0.008 | 42.568 | 73.039 | 0.956 | 5.489 | 349652.092 | 0.004 | 289.558 | 2.631 | 1014.201 | 0.003 | 36.398 | 37.172 | 50.559 | 164.13 | 4.867 | 287.008 |
| 1 | train | 13.09 | 13.024 | 13.104 | 0.007 | 44.788 | 68.355 | 0.981 | 5.452 | 375744.315 | 0.005 | 355.316 | 2.65 | 961.858 | 0.002 | 36.484 | 37.295 | 50.777 | 163.858 | 4.737 | 249.403 |
| 1 | test | 14.631 | 14.173 | 14.595 | 0.008 | 42.499 | 85.128 | 0.523 | 9.405 | 353298.221 | 0.005 | 203.09 | 2.624 | 804.926 | 0.003 | 36.952 | 37.694 | 51.623 | 166.092 | 4.924 | 297.951 |
| 0 | test | 18.07 | 16.713 | 17.988 | 0.009 | 43.694 | 89.481 | 0.534 | 9.428 | 430168.457 | 0.004 | 268.618 | 2.509 | 1037.777 | 0.004 | 42.925 | 43.702 | 59.394 | 182.608 | 5.342 | 308.069 |
| 2 | train | 10.098 | 9.838 | 10.003 | 0.007 | 43.77 | 68.8 | 0.95 | 5.428 | 471058.076 | 0.004 | 576.044 | 2.301 | 1056.621 | 0.003 | 29.201 | 29.929 | 41.012 | 142.356 | 4.793 | 245.412 |
| 1 | test | 13.668 | 13.269 | 13.671 | 0.006 | 44.4 | 77.711 | 1.014 | 5.508 | 419584.911 | 0.004 | 305.192 | 2.692 | 1054.033 | 0.002 | 36.758 | 37.577 | 51.181 | 164.798 | 4.825 | 278.325 |
| 1 | train | 14.781 | 13.862 | 14.764 | 0.007 | 43.02 | 76.332 | 0.569 | 9.112 | 514093.878 | 0.008 | 279.655 | 2.662 | 1038.008 | 0.002 | 38.075 | 38.826 | 52.964 | 169.101 | 5.011 | 277.291 |
| 1 | train | 13.45 | 13.369 | 13.435 | 0.007 | 42.35 | 83.789 | 0.556 | 9.079 | 516322.987 | 0.006 | 395.303 | 2.781 | 1219.793 | 0.003 | 36.338 | 37.113 | 50.653 | 164.329 | 5.044 | 274.601 |
| 0 | train | 14.774 | 14.679 | 14.785 | 0.007 | 46.904 | 72.588 | 1.018 | 5.504 | 355350.005 | 0.004 | 205.978 | 2.752 | 974.126 | 0.003 | 40.258 | 41.163 | 55.727 | 174.816 | 5.116 | 306.342 |
| 1 | test | 14.362 | 13.059 | 14.375 | 0.007 | 39.702 | 67.179 | 0.994 | 5.506 | 338228.867 | 0.004 | 267.711 | 2.615 | 925.512 | 0.002 | 39.049 | 39.917 | 54.168 | 171.64 | 4.922 | 280.38 |
| 1 | train | 14.834 | 14.218 | 14.824 | 0.006 | 47.883 | 72.027 | 0.382 | 13.397 | 405493.549 | 0.005 | 242.303 | 2.728 | 905.387 | 0.002 | 39.373 | 40.3 | 54.889 | 172.777 | 4.883 | 301.696 |
| 2 | test | 13.863 | 13.424 | 13.827 | 0.008 | 42.909 | 77.894 | 0.518 | 9.272 | 437258.339 | 0.01 | 434.054 | 2.552 | 980.909 | 0.003 | 35.284 | 35.991 | 49.24 | 160.828 | 4.895 | 278.612 |
| 1 | train | 15.033 | 14.044 | 15.029 | 0.007 | 44.378 | 76.79 | 0.972 | 5.468 | 359328.328 | 0.008 | 260.219 | 2.663 | 899.361 | 0.002 | 39.015 | 39.818 | 54.111 | 171.44 | 4.859 | 273.64 |
| 0 | test | 21.56 | 19.883 | 21.428 | 0.01 | 36.673 | 109.399 | 0.363 | 11.452 | 305836.403 | 0.017 | 223.444 | 2.424 | 436.744 | 0.006 | 46.009 | 46.119 | 63.673 | 190.709 | 5.175 | 308.7 |
| 0 | test | 14.556 | 13.541 | 14.58 | 0.007 | 47.86 | 64.211 | 1.01 | 5.442 | 245370.901 | 0.002 | 197.746 | 2.553 | 840.988 | 0.003 | 40.002 | 40.855 | 55.35 | 174.062 | 4.906 | 272.753 |
| 1 | test | 14.386 | 14.239 | 14.331 | 0.008 | 36.435 | 81.603 | 0.325 | 14.315 | 524900.808 | 0.011 | 344.207 | 2.646 | 822.556 | 0.004 | 35.41 | 35.963 | 49.473 | 161.657 | 4.856 | 288.389 |
| 0 | test | 16.492 | 16.244 | 16.457 | 0.008 | 40.606 | 71.928 | 0.376 | 13.82 | 203618.511 | 0.005 | 131.678 | 2.648 | 688.521 | 0.004 | 41.832 | 42.549 | 57.807 | 178.438 | 5.286 | 297.75 |
| 0 | test | 15.785 | 14.363 | 15.759 | 0.008 | 49.721 | 78.354 | 0.963 | 5.546 | 345488.087 | 0.005 | 284.69 | 2.463 | 1030.502 | 0.003 | 39.494 | 40.299 | 54.724 | 172.688 | 5.075 | 285.191 |
| 1 | test | 12.939 | 13.143 | 12.971 | 0.006 | 37.701 | 80.552 | 1 | 5.428 | 422561.728 | 0.003 | 313.808 | 2.319 | 1058.41 | 0.002 | 36.854 | 37.758 | 51.188 | 165.698 | 4.776 | 252.283 |
| 1 | test | 14.521 | 13.856 | 14.505 | 0.007 | 39.501 | 82.523 | 0.949 | 5.504 | 459192.881 | 0.006 | 278.234 | 2.696 | 1116.183 | 0.002 | 37.49 | 38.309 | 52.038 | 167.24 | 4.838 | 285.659 |
| 2 | train | 9.661 | 10.535 | 9.58 | 0.007 | 43.436 | 96.118 | 0.527 | 9.223 | 665111.692 | 0.007 | 453.484 | 2.799 | 955.4 | 0.003 | 27.339 | 27.978 | 38.893 | 137.206 | 4.964 | 305.346 |
| 1 | train | 13.92 | 13.01 | 13.952 | 0.007 | 44.901 | 64.829 | 1.05 | 5.487 | 238561.297 | 0.004 | 191.186 | 2.592 | 763.435 | 0.003 | 39.332 | 40.176 | 54.44 | 171.738 | 4.874 | 254.876 |
| 1 | train | 14.329 | 14.071 | 14.264 | 0.009 | 36.277 | 90.689 | 0.462 | 9.893 | 392363.608 | 0.011 | 362.47 | 2.754 | 1002.767 | 0.005 | 34.695 | 35.156 | 48.545 | 158.957 | 4.858 | 289.055 |
| 0 | test | 20.667 | 19.148 | 20.538 | 0.01 | 42.122 | 123.063 | 0.377 | 11.248 | 336656.779 | 0.013 | 245.878 | 2.549 | 323.593 | 0.006 | 44.644 | 44.794 | 61.789 | 186.819 | 5.065 | 309.147 |
| 1 | train | 13.951 | 13.348 | 13.972 | 0.006 | 50.051 | 67.235 | 1.019 | 5.485 | 421774.26 | 0.003 | 270.138 | 2.89 | 880.987 | 0.002 | 38.373 | 39.332 | 53.141 | 169.843 | 4.73 | 261.007 |
| 0 | train | 14.15 | 15.271 | 14.179 | 0.006 | 49.021 | 72.9 | 0.587 | 9.012 | 408980.205 | 0.004 | 227.589 | 2.825 | 954.214 | 0.002 | 39.542 | 40.433 | 54.779 | 173.28 | 4.91 | 276.063 |
| 1 | test | 21.726 | 21.003 | 21.585 | 0.013 | 30.367 | 117.163 | 0.161 | 19.872 | 239665.772 | 0.025 | 240.569 | 2.458 | 119.507 | 0.007 | 45.822 | 45.27 | 63.492 | 189.072 | 5.141 | 312.724 |
| 0 | train | 21.772 | 20.03 | 21.634 | 0.011 | 26.601 | 107.349 | 0.362 | 11.494 | 241482.755 | 0.018 | 157.236 | 2.481 | 385.108 | 0.005 | 46.452 | 46.506 | 64.229 | 191.375 | 5.259 | 303.784 |
| 2 | test | 16.165 | 20.549 | 16.052 | 0.014 | 24.242 | 104.262 | 0.153 | 19.94 | 209715.243 | 0.029 | 461.007 | 2.555 | 47.331 | 0.007 | 36.798 | 35.966 | 51.862 | 160.569 | 5.05 | 309.939 |
| 3 | train | 7.308 | 8.692 | 7.214 | 0.007 | 38.623 | 67.524 | 0.908 | 5.567 | 324007.375 | 0.004 | 474.038 | 2.685 | 52.16 | 0.003 | 22.215 | 22.788 | 32.051 | 119.38 | 4.811 | 257.37 |
| 3 | test | 10.979 | 16.262 | 10.881 | 0.011 | 33.572 | 107.298 | 0.309 | 12.045 | 222498.736 | 0.013 | 462.601 | 2.804 | 11.021 | 0.005 | 27.536 | 27.374 | 39.438 | 133.026 | 4.677 | 298.681 |
| 3 | train | 10.013 | 15.033 | 9.915 | 0.011 | 25.579 | 87.058 | 0.327 | 11.675 | 187157.173 | 0.012 | 437.178 | 2.789 | 38.067 | 0.006 | 25.998 | 25.847 | 37.373 | 127.68 | 4.565 | 304.118 |
| 3 | test | 10.143 | 15.52 | 10.043 | 0.011 | 25.931 | 86.452 | 0.316 | 11.843 | 174308.572 | 0.014 | 556.793 | 2.738 | 41.846 | 0.006 | 26.086 | 25.905 | 37.406 | 127.35 | 4.645 | 299.911 |
| 3 | train | 11.105 | 16.554 | 11.005 | 0.012 | 37.906 | 91.641 | 0.297 | 12.144 | 186208.87 | 0.022 | 496.706 | 2.716 | 36.75 | 0.006 | 27.905 | 27.576 | 39.905 | 133.197 | 4.648 | 309.392 |
| 3 | test | 9.731 | 16.731 | 9.633 | 0.012 | 35.398 | 90.377 | 0.288 | 12.277 | 114229.312 | 0.021 | 439.342 | 2.958 | 11.1 | 0.006 | 25.338 | 24.956 | 36.451 | 122.242 | 4.667 | 300.859 |
| 3 | train | 9.157 | 16.695 | 9.057 | 0.012 | 25.198 | 84.992 | 0.188 | 18.272 | 178628.38 | 0.02 | 691.385 | 2.854 | 7.436 | 0.006 | 24.241 | 23.895 | 35.005 | 118.121 | 4.715 | 307.31 |
| 2 | train | 12.739 | 15.892 | 12.645 | 0.01 | 30.627 | 97.533 | 0.352 | 11.491 | 371661.168 | 0.015 | 496.558 | 2.775 | 57.291 | 0.005 | 31.067 | 31.061 | 44.117 | 146.846 | 4.971 | 302.155 |
| 3 | train | 8.084 | 16.654 | 7.988 | 0.014 | 21.855 | 88.504 | 0.266 | 12.62 | 127676.061 | 0.021 | 581.45 | 3.016 | 6.476 | 0.008 | 22.261 | 21.679 | 32.026 | 106.539 | 4.853 | 295.512 |
| 3 | train | 9.775 | 16.001 | 9.675 | 0.012 | 37.642 | 97.88 | 0.198 | 17.975 | 195849.059 | 0.02 | 638.104 | 2.831 | 17.863 | 0.006 | 25.633 | 25.325 | 36.84 | 124.483 | 4.795 | 310.557 |
| 3 | test | 9.828 | 15.992 | 9.73 | 0.011 | 23.687 | 87.398 | 0.197 | 18.016 | 169922.964 | 0.019 | 499.996 | 2.947 | 8.391 | 0.006 | 25.55 | 25.231 | 36.672 | 124.231 | 4.691 | 294.45 |
| 3 | train | 8.585 | 15.274 | 8.489 | 0.011 | 25.503 | 92.148 | 0.311 | 11.899 | 122334.228 | 0.018 | 461.074 | 2.878 | 9.789 | 0.005 | 23.231 | 22.986 | 33.504 | 115.569 | 4.673 | 306.942 |
| 3 | test | 7.829 | 9.303 | 7.73 | 0.008 | 22.621 | 73.127 | 0.649 | 8.338 | 524711.058 | 0.008 | 546.921 | 2.963 | 19.364 | 0.004 | 22.496 | 22.86 | 32.318 | 119.27 | 4.885 | 280.47 |
| 3 | test | 8.523 | 12.279 | 8.427 | 0.009 | 27.814 | 83.126 | 0.486 | 9.859 | 331211.159 | 0.013 | 900.446 | 2.769 | 22.594 | 0.004 | 23.705 | 23.75 | 34.36 | 121.327 | 4.939 | 288.765 |
| 3 | test | 12.399 | 19.414 | 12.294 | 0.015 | 36.485 | 98.039 | 0.154 | 19.668 | 168181.734 | 0.031 | 529.156 | 2.685 | 14.654 | 0.007 | 30.422 | 29.535 | 43.395 | 137.012 | 4.859 | 310.345 |
| 3 | train | 7.054 | 10.296 | 6.957 | 0.008 | 24.17 | 72.509 | 0.443 | 10.084 | 261572.296 | 0.011 | 475.514 | 2.987 | 102.298 | 0.003 | 20.891 | 21.13 | 30.313 | 112.581 | 4.574 | 267.688 |
| 3 | test | 5.495 | 11.207 | 5.412 | 0.01 | 27.954 | 72.326 | 0.722 | 6.635 | 164851.425 | 0.011 | 550.189 | 2.957 | 14.906 | 0.005 | 17.538 | 17.457 | 25.682 | 94.594 | 4.893 | 304.387 |
| 2 | train | 9.831 | 11.221 | 9.739 | 0.008 | 35.546 | 77.557 | 0.478 | 9.696 | 502267.085 | 0.01 | 671.406 | 2.935 | 236.7 | 0.005 | 26.515 | 26.926 | 37.865 | 133.216 | 4.85 | 274.686 |
| 3 | test | 7.843 | 11.709 | 7.746 | 0.009 | 22.311 | 76.755 | 0.552 | 9.334 | 540132.079 | 0.01 | 1155.51 | 2.896 | 50.516 | 0.004 | 22.246 | 22.378 | 32.275 | 116.192 | 4.885 | 291.009 |
| 3 | test | 9.568 | 12.063 | 9.477 | 0.009 | 32.165 | 79.739 | 0.305 | 14.815 | 546508.948 | 0.011 | 707.579 | 2.856 | 197.052 | 0.005 | 25.609 | 25.891 | 36.779 | 129.453 | 4.858 | 300.381 |
| 3 | test | 8.08 | 15.702 | 7.981 | 0.012 | 33.262 | 82.836 | 0.194 | 18.058 | 115309.409 | 0.019 | 536.958 | 2.966 | 9.105 | 0.006 | 22.268 | 21.972 | 32.059 | 110.858 | 4.746 | 290.283 |
| 3 | test | 9.667 | 17.089 | 9.569 | 0.017 | 11.169 | 82.064 | 0.213 | 18.096 | 284337.039 | 0.02 | 1116.24 | 2.941 | 10.095 | 0.009 | 25.534 | 24.623 | 36.492 | 116.343 | 5.269 | 315.532 |
| 3 | train | 9.875 | 13.639 | 9.781 | 0.01 | 24.854 | 81.017 | 0.44 | 10.423 | 430314.679 | 0.014 | 888.832 | 2.882 | 19.531 | 0.005 | 26.001 | 25.923 | 37.428 | 128.928 | 5.044 | 305.601 |
| 3 | test | 9.624 | 15.4 | 9.527 | 0.011 | 34.429 | 87.909 | 0.207 | 17.585 | 128805.105 | 0.016 | 400.517 | 2.851 | 42.289 | 0.006 | 25.166 | 24.982 | 36.202 | 123.262 | 4.754 | 292.21 |
| 3 | train | 9.42 | 13.913 | 9.324 | 0.01 | 31.155 | 81.491 | 0.252 | 16.328 | 194619.674 | 0.017 | 368.113 | 2.979 | 15.03 | 0.005 | 25.013 | 24.944 | 35.985 | 124.885 | 4.861 | 283.059 |
| 3 | test | 8.774 | 17.254 | 8.676 | 0.015 | 30.336 | 88.559 | 0.165 | 19.041 | 137302.005 | 0.024 | 617.409 | 2.789 | 9.8 | 0.008 | 23.527 | 22.871 | 33.703 | 111.823 | 4.927 | 304.25 |
| 3 | test | 8.003 | 14.411 | 7.905 | 0.011 | 27.704 | 87.796 | 0.219 | 17.237 | 138566.91 | 0.012 | 497.736 | 2.865 | 18.107 | 0.005 | 22.207 | 22.059 | 32.178 | 112.908 | 4.636 | 303.365 |
| 3 | train | 10.246 | 16.01 | 10.149 | 0.011 | 27.266 | 90.428 | 0.314 | 11.925 | 209008.109 | 0.017 | 511.825 | 2.789 | 27.432 | 0.006 | 26.6 | 26.35 | 38.241 | 129.181 | 4.683 | 315.514 |
| 3 | test | 11.711 | 17.258 | 11.612 | 0.012 | 31.214 | 97.174 | 0.187 | 18.472 | 205539.186 | 0.019 | 556.942 | 2.718 | 26.192 | 0.006 | 28.964 | 28.571 | 41.381 | 136.622 | 4.716 | 302.101 |
| 2 | test | 9.532 | 14.659 | 9.432 | 0.01 | 29.734 | 84.41 | 0.348 | 11.447 | 168843.571 | 0.015 | 404.849 | 2.954 | 28.857 | 0.005 | 25.145 | 25.105 | 36.203 | 125.048 | 4.662 | 299.246 |
| 3 | test | 9.978 | 16.021 | 9.879 | 0.012 | 33.384 | 85.966 | 0.296 | 12.106 | 152677.366 | 0.016 | 619.327 | 2.871 | 49.575 | 0.006 | 25.898 | 25.536 | 37.076 | 125.143 | 4.575 | 301.763 |
| 3 | train | 9.379 | 17.849 | 9.28 | 0.016 | 25.288 | 99.159 | 0.151 | 19.568 | 152949.94 | 0.026 | 652.206 | 2.861 | 7.43 | 0.008 | 24.848 | 23.923 | 35.343 | 112.963 | 4.892 | 307.479 |
| 3 | train | 12.826 | 20.071 | 12.722 | 0.015 | 29.171 | 97.898 | 0.152 | 19.815 | 151700.596 | 0.027 | 455.336 | 2.742 | 15.074 | 0.008 | 31.01 | 30.105 | 44.299 | 138.357 | 4.85 | 321.223 |
| 3 | test | 6.126 | 9.475 | 6.032 | 0.007 | 35.605 | 75.212 | 0.489 | 9.686 | 308355.483 | 0.008 | 612.581 | 3.047 | 21.897 | 0.002 | 19.04 | 19.455 | 27.799 | 106.511 | 4.49 | 267.333 |
| 2 | test | 11.159 | 14.463 | 11.065 | 0.009 | 23.914 | 94.573 | 0.372 | 11.14 | 384484.059 | 0.014 | 484.424 | 2.845 | 41.6 | 0.004 | 28.425 | 28.513 | 40.594 | 138.425 | 4.891 | 297.477 |
| 2 | test | 10.65 | 14.806 | 10.554 | 0.01 | 27.231 | 84.226 | 0.347 | 11.409 | 239235.077 | 0.015 | 452.391 | 2.975 | 49.831 | 0.005 | 27.358 | 27.272 | 39.183 | 133.53 | 4.755 | 297.361 |
| 3 | test | 7.704 | 14.866 | 7.608 | 0.012 | 38.041 | 83.562 | 0.204 | 17.689 | 113216.834 | 0.016 | 480.695 | 2.91 | 39.72 | 0.006 | 21.439 | 21.214 | 30.962 | 107.8 | 4.784 | 285.198 |
| 3 | train | 10.827 | 18.63 | 10.725 | 0.014 | 26.719 | 92.434 | 0.159 | 19.359 | 145737.323 | 0.023 | 575.401 | 2.879 | 9.352 | 0.008 | 27.374 | 26.649 | 39.18 | 126.337 | 4.739 | 309.409 |
| 3 | train | 8.599 | 17.201 | 8.502 | 0.016 | 27.992 | 90.909 | 0.173 | 18.854 | 143909.659 | 0.018 | 689.915 | 2.881 | 10.409 | 0.008 | 23.366 | 22.624 | 33.595 | 109.56 | 4.906 | 316.771 |
| 3 | test | 10.621 | 17.679 | 10.52 | 0.013 | 27.384 | 90.654 | 0.267 | 12.621 | 157246.734 | 0.024 | 471.304 | 2.838 | 12.958 | 0.006 | 26.932 | 26.408 | 38.518 | 127.199 | 4.622 | 302.166 |
| 3 | train | 11.085 | 18.607 | 10.984 | 0.015 | 27.799 | 93.888 | 0.161 | 19.322 | 159373.122 | 0.023 | 536.339 | 2.817 | 15.549 | 0.008 | 27.98 | 27.216 | 39.906 | 128.605 | 4.819 | 309.277 |
| 3 | train | 8.857 | 14.667 | 8.758 | 0.011 | 22.872 | 90.088 | 0.327 | 11.633 | 175508.86 | 0.019 | 596.564 | 3.041 | 38.153 | 0.005 | 23.85 | 23.664 | 34.453 | 119.258 | 4.515 | 292.867 |
| 3 | test | 11.232 | 16.707 | 11.131 | 0.012 | 30.391 | 95.712 | 0.195 | 18.137 | 213658.749 | 0.02 | 490.303 | 2.797 | 22.102 | 0.006 | 28.232 | 27.968 | 40.45 | 134.344 | 4.665 | 303.839 |
| 3 | test | 6.319 | 11.606 | 6.23 | 0.01 | 22.918 | 75.903 | 0.409 | 10.719 | 210109.316 | 0.014 | 559.55 | 2.891 | 18.98 | 0.005 | 19.294 | 19.376 | 28.223 | 103.19 | 4.793 | 294.196 |
| 3 | train | 7.425 | 12.65 | 7.324 | 0.009 | 31.107 | 73.992 | 0.378 | 10.967 | 272425.749 | 0.013 | 833.204 | 2.892 | 21.313 | 0.004 | 21.341 | 21.356 | 30.826 | 111.906 | 4.617 | 296.601 |
| 2 | train | 11.444 | 15.804 | 11.347 | 0.011 | 30.517 | 89.696 | 0.328 | 11.754 | 176597.106 | 0.018 | 385.709 | 2.766 | 31.498 | 0.006 | 28.703 | 28.519 | 41.056 | 137.48 | 4.797 | 300.246 |
| 3 | train | 10.24 | 18.887 | 10.139 | 0.018 | 30.268 | 98.126 | 0.107 | 27.38 | 119878.877 | 0.02 | 453.517 | 2.855 | 11.626 | 0.01 | 26.364 | 25.357 | 37.558 | 118.063 | 4.992 | 303.479 |
| 2 | train | 11.737 | 15.284 | 11.64 | 0.01 | 47.05 | 91.005 | 0.351 | 11.5 | 250085.813 | 0.014 | 465.059 | 2.754 | 109.841 | 0.005 | 29.417 | 29.336 | 42.103 | 140.976 | 4.963 | 302.068 |
| 3 | train | 11.259 | 19.208 | 11.157 | 0.015 | 25.052 | 88.564 | 0.156 | 19.468 | 133738.697 | 0.023 | 491.664 | 2.834 | 13.883 | 0.008 | 28.305 | 27.496 | 40.334 | 129.145 | 4.686 | 300.048 |
| 2 | train | 9.838 | 11.94 | 9.746 | 0.009 | 22.826 | 77.87 | 0.549 | 9.198 | 648787.197 | 0.012 | 618.598 | 2.958 | 66.06 | 0.005 | 26.367 | 26.613 | 37.777 | 132.392 | 4.954 | 295.102 |
| 3 | train | 10.334 | 14.468 | 10.238 | 0.011 | 27.635 | 90.434 | 0.381 | 10.942 | 208448.842 | 0.016 | 508.764 | 2.741 | 45.036 | 0.006 | 26.798 | 26.684 | 38.526 | 131.373 | 4.646 | 290.578 |
| 3 | train | 7.709 | 14.179 | 7.612 | 0.011 | 35.567 | 77.75 | 0.221 | 17.217 | 143567.975 | 0.012 | 573.793 | 2.963 | 26.496 | 0.005 | 21.805 | 21.688 | 31.582 | 111.034 | 4.691 | 285.669 |
| 2 | train | 11.65 | 15.49 | 11.553 | 0.011 | 25.329 | 98.224 | 0.361 | 11.288 | 296713.41 | 0.014 | 433.069 | 2.766 | 62.343 | 0.005 | 29.138 | 29.014 | 41.633 | 139.206 | 4.918 | 296.412 |
| 3 | train | 8.18 | 10.275 | 8.089 | 0.007 | 34.731 | 74.479 | 0.567 | 8.922 | 441391.721 | 0.008 | 455.898 | 2.989 | 77.026 | 0.003 | 23.563 | 23.987 | 33.949 | 122.943 | 5.033 | 289.865 |
| 2 | train | 9.158 | 10.65 | 9.063 | 0.008 | 37.28 | 76.691 | 0.491 | 9.584 | 481130.495 | 0.011 | 612.487 | 2.82 | 167.075 | 0.005 | 25.196 | 25.523 | 35.988 | 129.076 | 4.696 | 275.379 |
| 3 | train | 7.824 | 10.023 | 7.724 | 0.008 | 25.821 | 76.356 | 0.524 | 9.256 | 540939.199 | 0.01 | 762.359 | 2.955 | 208.929 | 0.005 | 22.57 | 22.905 | 32.398 | 119.76 | 4.672 | 290.363 |
| 3 | test | 9.825 | 16.04 | 9.729 | 0.012 | 21.823 | 87.676 | 0.302 | 11.989 | 118662.308 | 0.018 | 362.11 | 2.852 | 33.523 | 0.006 | 25.694 | 25.33 | 36.895 | 123.832 | 4.692 | 303.595 |
| 2 | test | 11.49 | 13.035 | 11.401 | 0.008 | 33.942 | 78.866 | 0.515 | 9.461 | 640018.219 | 0.012 | 644.057 | 2.784 | 244.477 | 0.004 | 29.289 | 29.642 | 41.456 | 142.818 | 5.162 | 294.492 |
| 3 | train | 8.375 | 10.394 | 8.277 | 0.009 | 28.489 | 72.652 | 0.511 | 9.445 | 391658.534 | 0.01 | 677.446 | 2.911 | 123.028 | 0.006 | 23.499 | 23.697 | 33.675 | 121.943 | 4.48 | 281.251 |
| 3 | train | 9.604 | 16.284 | 9.505 | 0.012 | 24.931 | 83.663 | 0.291 | 12.171 | 143481.417 | 0.02 | 448.178 | 2.876 | 20.771 | 0.006 | 25.186 | 24.827 | 36.171 | 122.394 | 4.57 | 302.037 |
| 2 | test | 13.129 | 17.642 | 13.027 | 0.012 | 35.662 | 99.135 | 0.296 | 12.314 | 250725.836 | 0.017 | 578.688 | 2.687 | 39.66 | 0.006 | 31.319 | 31.088 | 44.536 | 145.942 | 4.831 | 313.216 |
| 2 | test | 13.62 | 17.465 | 13.521 | 0.012 | 27.394 | 95.144 | 0.197 | 18.165 | 176206.497 | 0.022 | 317.48 | 2.668 | 79.965 | 0.006 | 32.569 | 32.252 | 46.216 | 149.04 | 5.072 | 310.023 |
| 2 | test | 12.979 | 17.635 | 12.881 | 0.012 | 25.625 | 91.132 | 0.295 | 12.39 | 233359.639 | 0.022 | 487.889 | 2.694 | 55.377 | 0.006 | 31.314 | 31.023 | 44.432 | 145.01 | 4.885 | 307.298 |
| 2 | test | 14.397 | 18.193 | 14.296 | 0.012 | 24.445 | 100.162 | 0.194 | 18.369 | 233373.982 | 0.019 | 327.937 | 2.617 | 39.36 | 0.006 | 33.774 | 33.442 | 47.802 | 153.704 | 4.965 | 320.376 |
| 2 | train | 10.961 | 16.421 | 10.864 | 0.011 | 32.443 | 86.867 | 0.197 | 18.05 | 212013.992 | 0.017 | 498.48 | 2.767 | 30.339 | 0.006 | 27.718 | 27.424 | 39.74 | 133.27 | 4.588 | 303.327 |
| 2 | test | 10.091 | 12.107 | 9.998 | 0.008 | 30.918 | 78.588 | 0.472 | 9.791 | 504383.033 | 0.008 | 452.149 | 2.908 | 72.041 | 0.004 | 26.797 | 27.148 | 38.155 | 134.166 | 4.844 | 283.028 |
| 3 | test | 6.272 | 11.968 | 6.181 | 0.011 | 22.312 | 71.525 | 0.458 | 10.497 | 209257.73 | 0.012 | 668.649 | 3.06 | 9.98 | 0.005 | 19.059 | 18.988 | 27.807 | 100.595 | 4.847 | 300.115 |
| 3 | train | 8.909 | 10.976 | 8.81 | 0.009 | 27.687 | 78.25 | 0.437 | 10.16 | 342240.471 | 0.012 | 583.285 | 2.89 | 28.344 | 0.005 | 24.363 | 24.671 | 34.849 | 125.562 | 4.539 | 285.53 |
| 3 | train | 9.615 | 17.387 | 9.514 | 0.013 | 35.41 | 95.164 | 0.269 | 12.483 | 119785.106 | 0.021 | 477.786 | 2.774 | 17.658 | 0.007 | 25.216 | 24.639 | 36.199 | 119.773 | 4.661 | 305.641 |
| 2 | test | 11.793 | 17.911 | 11.691 | 0.013 | 32.578 | 93.732 | 0.268 | 12.653 | 174885.733 | 0.024 | 544.236 | 2.806 | 27.405 | 0.007 | 29.415 | 28.804 | 41.971 | 136.469 | 4.641 | 299.055 |
| 3 | train | 9.182 | 15.359 | 9.084 | 0.011 | 33.963 | 85.535 | 0.309 | 11.878 | 138106.576 | 0.018 | 573.756 | 2.851 | 38.625 | 0.006 | 24.451 | 24.134 | 35.194 | 120.6 | 4.493 | 303.18 |
| 2 | train | 9.527 | 15.211 | 9.43 | 0.011 | 32.103 | 93.541 | 0.325 | 11.664 | 152329.249 | 0.016 | 514.265 | 2.812 | 22.502 | 0.006 | 25.206 | 24.964 | 36.358 | 123.788 | 4.659 | 312.451 |
| 3 | train | 12.642 | 17.632 | 12.544 | 0.012 | 23.042 | 94.816 | 0.302 | 12.283 | 237591.079 | 0.018 | 423.803 | 2.819 | 61.768 | 0.006 | 31.055 | 30.712 | 44.136 | 143.915 | 4.916 | 304.326 |
| 2 | train | 12.721 | 17.002 | 12.622 | 0.012 | 27.738 | 90.76 | 0.345 | 11.559 | 310108.933 | 0.017 | 563.012 | 2.855 | 126.75 | 0.006 | 31.115 | 30.753 | 44.414 | 144.815 | 4.972 | 308.887 |
| 2 | train | 13.861 | 18.861 | 13.755 | 0.013 | 33.144 | 103.875 | 0.167 | 19.336 | 169765.821 | 0.022 | 391.828 | 2.67 | 30.145 | 0.007 | 32.786 | 32.225 | 46.583 | 148.539 | 4.899 | 319.93 |
| 3 | train | 9.446 | 17.662 | 9.346 | 0.016 | 17.239 | 89.57 | 0.174 | 18.876 | 173759.902 | 0.022 | 722.182 | 2.826 | 11.443 | 0.008 | 25.039 | 24.207 | 35.877 | 115.339 | 4.957 | 302.811 |
| 3 | test | 5.473 | 10.695 | 5.39 | 0.009 | 27.445 | 75.589 | 0.268 | 16.065 | 162952.251 | 0.011 | 530.215 | 3.105 | 7.748 | 0.004 | 17.696 | 17.833 | 26.046 | 97.358 | 4.728 | 294.113 |
| 3 | train | 8.61 | 9.758 | 8.518 | 0.008 | 38.985 | 72.891 | 0.502 | 9.458 | 391376.072 | 0.008 | 492.514 | 2.955 | 127.471 | 0.004 | 24.711 | 25.161 | 35.347 | 127.398 | 4.895 | 261.013 |
| 2 | train | 10.092 | 13.128 | 9.995 | 0.009 | 31.439 | 83.471 | 0.403 | 10.65 | 303258.922 | 0.011 | 479.846 | 2.767 | 68.443 | 0.005 | 26.344 | 26.485 | 37.786 | 131.673 | 4.706 | 291.773 |
| 2 | train | 10.459 | 10.735 | 10.372 | 0.007 | 25.267 | 68.671 | 0.885 | 5.624 | 370863.46 | 0.007 | 262.826 | 2.753 | 431.851 | 0.003 | 28.892 | 29.586 | 40.855 | 141.492 | 4.844 | 253.924 |
| 3 | test | 8.693 | 10.63 | 8.596 | 0.009 | 34.653 | 74.603 | 0.851 | 5.851 | 425879.455 | 0.009 | 620.176 | 3.086 | 310.778 | 0.005 | 24.269 | 24.514 | 34.676 | 124.73 | 4.738 | 293.011 |
| 2 | train | 10.183 | 12.5 | 10.088 | 0.009 | 32.393 | 83.194 | 0.411 | 10.565 | 380902.475 | 0.018 | 557.458 | 2.863 | 50.765 | 0.005 | 26.574 | 26.793 | 37.956 | 133.207 | 4.739 | 301.843 |
| 3 | train | 10.086 | 11.599 | 9.998 | 0.009 | 28.632 | 76.068 | 0.534 | 9.208 | 459026.333 | 0.011 | 451.227 | 2.823 | 419.153 | 0.004 | 26.837 | 27.235 | 38.199 | 134.279 | 4.943 | 274.423 |
| 2 | train | 9.793 | 11.887 | 9.699 | 0.009 | 33.672 | 77.725 | 0.439 | 10.205 | 476115.972 | 0.01 | 504.244 | 2.805 | 76.914 | 0.004 | 26.26 | 26.555 | 37.569 | 132.309 | 4.767 | 293.594 |
| 2 | test | 13.23 | 17.684 | 13.127 | 0.012 | 29.005 | 94.814 | 0.291 | 12.365 | 160634.912 | 0.02 | 354.153 | 2.749 | 48.636 | 0.006 | 31.873 | 31.452 | 45.448 | 146.234 | 4.932 | 308.862 |
| 2 | train | 13.175 | 16.873 | 13.078 | 0.011 | 37.371 | 95.751 | 0.204 | 17.957 | 253405.248 | 0.02 | 438.413 | 2.745 | 66.365 | 0.006 | 31.746 | 31.494 | 45.099 | 147.949 | 4.85 | 305.652 |
| 2 | train | 14.969 | 18.858 | 14.865 | 0.012 | 26.361 | 99.295 | 0.182 | 18.92 | 233251.34 | 0.018 | 351.743 | 2.666 | 52.388 | 0.006 | 34.687 | 34.264 | 49.087 | 156.098 | 5.011 | 311.773 |
| 3 | train | 10.046 | 18.514 | 9.948 | 0.016 | 26.763 | 95.102 | 0.234 | 13.086 | 93605.306 | 0.024 | 355.435 | 3.189 | 7.977 | 0.008 | 26.096 | 25.065 | 37.152 | 117.27 | 4.914 | 298.153 |
| 2 | train | 12.815 | 16.647 | 12.719 | 0.011 | 30.948 | 99.465 | 0.325 | 11.877 | 251329.184 | 0.015 | 492.032 | 2.818 | 58.043 | 0.006 | 31.325 | 31.161 | 44.673 | 146.306 | 4.995 | 306.633 |
| 2 | test | 11.479 | 12.421 | 11.398 | 0.008 | 40.966 | 76.179 | 0.832 | 5.862 | 485644.382 | 0.011 | 416.023 | 2.819 | 141.024 | 0.003 | 29.548 | 30.057 | 41.746 | 144.057 | 5.009 | 285.905 |
| 2 | test | 12.812 | 17.372 | 12.711 | 0.012 | 30.305 | 91.609 | 0.193 | 18.303 | 204692.734 | 0.022 | 438.537 | 2.79 | 84.641 | 0.006 | 30.88 | 30.609 | 43.795 | 143.692 | 4.834 | 300.232 |
| 2 | test | 13.036 | 18.869 | 12.933 | 0.014 | 36.788 | 95.23 | 0.165 | 19.314 | 159616.466 | 0.027 | 467.379 | 2.691 | 30.845 | 0.007 | 31.415 | 30.786 | 44.657 | 143.072 | 4.699 | 306.747 |
| 3 | train | 11.058 | 16.584 | 10.959 | 0.012 | 24.512 | 90.273 | 0.295 | 12.177 | 168708.139 | 0.022 | 516.908 | 2.808 | 31.314 | 0.006 | 27.996 | 27.625 | 40.131 | 133.1 | 4.686 | 292.756 |
| 3 | train | 9.505 | 16.871 | 9.406 | 0.013 | 31.103 | 95.559 | 0.282 | 12.336 | 121795.138 | 0.021 | 475.813 | 2.807 | 12.962 | 0.007 | 25.011 | 24.531 | 35.97 | 120.264 | 4.773 | 302.003 |
| 3 | train | 10.786 | 16.223 | 10.688 | 0.012 | 32.165 | 89.49 | 0.309 | 11.967 | 201118.835 | 0.019 | 530.966 | 2.78 | 57.914 | 0.006 | 27.435 | 27.144 | 39.268 | 131.748 | 4.649 | 313.472 |
| 2 | test | 10.988 | 16.655 | 10.887 | 0.011 | 30.857 | 96.625 | 0.2 | 17.977 | 204128.964 | 0.018 | 511.287 | 2.754 | 36.593 | 0.006 | 27.769 | 27.468 | 39.86 | 133.061 | 4.88 | 302.486 |
| 2 | train | 11.163 | 12.078 | 11.081 | 0.008 | 28.95 | 92.557 | 0.534 | 9.23 | 634630.564 | 0.009 | 427.543 | 2.828 | 436.871 | 0.003 | 29.148 | 29.657 | 41.158 | 142.611 | 5.108 | 294.247 |
| 2 | train | 9.249 | 10.644 | 9.158 | 0.008 | 30.841 | 73.914 | 0.535 | 9.161 | 578218.967 | 0.01 | 560.311 | 3.083 | 323.549 | 0.004 | 25.498 | 26.03 | 36.63 | 130.408 | 5.134 | 291.482 |
| 2 | train | 10.075 | 13.618 | 9.978 | 0.01 | 43.669 | 85.49 | 0.242 | 16.6 | 214044.548 | 0.017 | 419.447 | 2.849 | 21.422 | 0.005 | 26.347 | 26.316 | 37.843 | 130.701 | 4.658 | 290.581 |
| 3 | train | 9.045 | 13.278 | 8.944 | 0.01 | 33.295 | 80.367 | 0.357 | 11.204 | 232249.169 | 0.015 | 681.434 | 2.815 | 55.708 | 0.006 | 24.275 | 24.239 | 34.929 | 123.019 | 4.361 | 301.07 |
| 3 | train | 8.795 | 12.337 | 8.7 | 0.009 | 21.991 | 74.789 | 0.332 | 14.434 | 554295.258 | 0.012 | 922.767 | 3.056 | 14.292 | 0.005 | 24.094 | 24.151 | 34.782 | 123.128 | 4.766 | 291.652 |
| 3 | train | 10.853 | 17.823 | 10.752 | 0.013 | 35.918 | 102.263 | 0.267 | 12.636 | 142861.706 | 0.022 | 438.976 | 2.774 | 18.118 | 0.007 | 27.453 | 26.904 | 39.319 | 128.996 | 4.67 | 300.957 |
| 3 | train | 12.154 | 17.418 | 12.056 | 0.012 | 32.843 | 87.013 | 0.294 | 12.345 | 184771.708 | 0.024 | 449.704 | 2.893 | 28.168 | 0.006 | 30 | 29.613 | 42.881 | 139.96 | 4.841 | 308.486 |
| 3 | test | 11.337 | 17.853 | 11.233 | 0.013 | 25.012 | 93.707 | 0.17 | 18.952 | 148247.338 | 0.024 | 408.144 | 2.88 | 23.675 | 0.007 | 28.403 | 27.784 | 40.544 | 132.44 | 4.628 | 313.399 |
| 2 | train | 13.458 | 18.665 | 13.354 | 0.013 | 18.568 | 92.917 | 0.315 | 12.189 | 347171.319 | 0.019 | 716.839 | 2.669 | 10.782 | 0.007 | 32.26 | 31.599 | 46.069 | 146.985 | 4.98 | 307.048 |
| 3 | train | 10.949 | 17.427 | 10.852 | 0.013 | 34.192 | 99.691 | 0.176 | 18.747 | 115711.714 | 0.024 | 445.982 | 2.809 | 24.595 | 0.007 | 27.837 | 27.198 | 39.901 | 129.66 | 4.912 | 301.57 |
| 2 | test | 12.504 | 16.926 | 12.404 | 0.011 | 24.661 | 93.336 | 0.301 | 12.238 | 291807.678 | 0.021 | 479.049 | 2.776 | 25.661 | 0.006 | 30.523 | 30.25 | 43.454 | 143.658 | 4.774 | 316.717 |
| 2 | test | 11.62 | 15.626 | 11.526 | 0.01 | 26.116 | 91.644 | 0.346 | 11.55 | 253250.033 | 0.013 | 398.636 | 2.823 | 62.121 | 0.005 | 29.029 | 28.933 | 41.408 | 139.403 | 4.909 | 298.909 |
| 2 | train | 13.072 | 18.787 | 12.967 | 0.013 | 28.814 | 97.315 | 0.166 | 19.261 | 162511.695 | 0.023 | 424.002 | 2.709 | 35.372 | 0.007 | 31.437 | 30.831 | 44.794 | 143.272 | 4.735 | 315.353 |
| 2 | test | 12.786 | 17.65 | 12.683 | 0.013 | 17.228 | 93.746 | 0.363 | 11.547 | 376853.111 | 0.017 | 700.083 | 2.772 | 20.788 | 0.007 | 31.114 | 30.606 | 44.425 | 143.566 | 5.019 | 319.534 |
| 2 | train | 11.625 | 16.673 | 11.53 | 0.011 | 35.333 | 89.092 | 0.311 | 12.046 | 220436.854 | 0.021 | 574.135 | 2.779 | 42.537 | 0.006 | 29.136 | 28.824 | 41.562 | 137.787 | 4.898 | 302.363 |
| 3 | train | 9.303 | 17.006 | 9.207 | 0.013 | 32.205 | 87.949 | 0.174 | 18.814 | 170028.69 | 0.022 | 683.377 | 2.9 | 7.164 | 0.007 | 24.657 | 24.083 | 35.413 | 117.446 | 4.852 | 301.828 |
| 3 | train | 10.234 | 16.259 | 10.134 | 0.012 | 35.678 | 87.838 | 0.3 | 12.092 | 165824.704 | 0.022 | 476.885 | 2.821 | 27.714 | 0.006 | 26.324 | 26.015 | 37.91 | 127.555 | 4.6 | 302.486 |
| 3 | train | 9.449 | 16.924 | 9.351 | 0.013 | 37.483 | 87.504 | 0.283 | 12.309 | 126543.812 | 0.018 | 587.25 | 2.919 | 9.279 | 0.007 | 24.918 | 24.456 | 35.877 | 119.95 | 4.701 | 306.714 |
| 2 | train | 11.765 | 13.425 | 11.68 | 0.008 | 32.94 | 83.196 | 0.467 | 9.939 | 643742.612 | 0.012 | 505.446 | 2.755 | 213.896 | 0.004 | 29.918 | 30.254 | 42.381 | 144.836 | 5.021 | 286.787 |
| 3 | test | 7.574 | 8.806 | 7.486 | 0.007 | 36.082 | 69.535 | 0.36 | 13.468 | 660387.639 | 0.006 | 515.543 | 2.84 | 308.29 | 0.003 | 22.916 | 23.597 | 32.992 | 122.148 | 5.038 | 285.987 |
| 3 | test | 9.856 | 17.329 | 9.758 | 0.014 | 22.987 | 101.661 | 0.269 | 12.552 | 174657.324 | 0.02 | 597.755 | 2.86 | 10.478 | 0.007 | 25.714 | 25.11 | 36.935 | 121.153 | 4.887 | 306.023 |
| 2 | train | 10.76 | 12.769 | 10.669 | 0.008 | 41.331 | 82.754 | 0.437 | 10.19 | 521520.653 | 0.011 | 647.874 | 2.77 | 162.52 | 0.003 | 28.077 | 28.367 | 39.887 | 138.724 | 4.908 | 290.03 |
| 3 | test | 10.65 | 17.819 | 10.551 | 0.013 | 28.56 | 93.994 | 0.169 | 18.986 | 125888.806 | 0.023 | 495.54 | 2.857 | 12.334 | 0.007 | 26.894 | 26.367 | 38.58 | 126.757 | 4.527 | 300.453 |
| 2 | train | 12.451 | 16.103 | 12.354 | 0.011 | 31.168 | 89.93 | 0.367 | 11.302 | 290044.489 | 0.015 | 429.822 | 2.763 | 33.959 | 0.006 | 30.559 | 30.446 | 43.647 | 144.421 | 5.014 | 318.37 |
| 3 | train | 8.756 | 17.31 | 8.66 | 0.017 | 29.537 | 85.409 | 0.245 | 12.949 | 97194.523 | 0.025 | 379.911 | 3.114 | 4.556 | 0.009 | 23.502 | 22.647 | 33.5 | 107.494 | 4.907 | 297.52 |
| 3 | train | 10.91 | 16.218 | 10.81 | 0.012 | 23.772 | 90.13 | 0.216 | 17.524 | 212665.887 | 0.016 | 458.505 | 2.801 | 46.646 | 0.006 | 27.733 | 27.394 | 39.841 | 132.982 | 4.689 | 299.911 |
| 3 | train | 8.036 | 16.615 | 7.939 | 0.015 | 26.3 | 84.9 | 0.163 | 19.152 | 136810.036 | 0.022 | 564.497 | 2.957 | 5.517 | 0.008 | 22.119 | 21.485 | 31.71 | 104.918 | 4.816 | 293.85 |
| 3 | train | 11.207 | 16.811 | 11.108 | 0.012 | 33.197 | 87.119 | 0.314 | 11.977 | 205439.647 | 0.023 | 658.431 | 2.762 | 20.32 | 0.006 | 27.99 | 27.635 | 40.069 | 133.126 | 4.708 | 300.303 |
| 2 | test | 13.424 | 19.408 | 13.318 | 0.013 | 34.701 | 106.265 | 0.166 | 19.356 | 274701.055 | 0.024 | 700.764 | 2.744 | 25.533 | 0.007 | 32.042 | 31.312 | 45.598 | 145.172 | 4.84 | 310.506 |
| 2 | test | 12.466 | 18.99 | 12.363 | 0.013 | 26.338 | 103.797 | 0.166 | 19.274 | 208794.72 | 0.024 | 633.732 | 2.774 | 11.919 | 0.006 | 30.25 | 29.677 | 43.098 | 138.889 | 4.787 | 304.655 |
| 3 | test | 12.757 | 19.848 | 12.651 | 0.015 | 31.094 | 100.395 | 0.152 | 19.799 | 158349.392 | 0.025 | 498.779 | 2.735 | 9.402 | 0.007 | 30.75 | 29.937 | 43.794 | 138.078 | 4.963 | 310.305 |
| 2 | test | 12.018 | 15.647 | 11.924 | 0.01 | 28.579 | 87.914 | 0.347 | 11.477 | 258717.285 | 0.015 | 396.738 | 2.671 | 93.957 | 0.005 | 29.693 | 29.704 | 42.354 | 141.991 | 4.867 | 303.409 |
| 2 | train | 11.546 | 14.703 | 11.451 | 0.01 | 27.776 | 100.054 | 0.373 | 11.128 | 274887.884 | 0.012 | 348.458 | 2.78 | 77.428 | 0.005 | 29.098 | 29.119 | 41.508 | 140.753 | 4.951 | 296.89 |
| 3 | train | 8.068 | 11.289 | 7.972 | 0.009 | 26.331 | 67.09 | 0.581 | 9.145 | 559511.981 | 0.011 | 1299.703 | 2.892 | 40.773 | 0.005 | 22.85 | 22.905 | 32.978 | 118.434 | 4.802 | 282.503 |
| 3 | train | 11.95 | 18.328 | 11.847 | 0.014 | 27.969 | 88.274 | 0.314 | 12.142 | 267357.504 | 0.021 | 840.728 | 2.773 | 28.494 | 0.007 | 29.565 | 28.838 | 42.176 | 135.59 | 4.873 | 319.155 |
| 3 | test | 8.31 | 11.004 | 8.211 | 0.009 | 27.67 | 75.489 | 0.492 | 9.653 | 358078.478 | 0.011 | 493.699 | 2.954 | 30.718 | 0.005 | 23.216 | 23.478 | 33.601 | 121.059 | 4.664 | 284.162 |
| 3 | train | 9.181 | 17.072 | 9.084 | 0.017 | 26.548 | 79.771 | 0.319 | 12.079 | 193229.715 | 0.021 | 760.161 | 2.816 | 13.5 | 0.009 | 24.448 | 23.66 | 35.097 | 113.17 | 5.128 | 311.208 |
| 3 | train | 12.418 | 19.566 | 12.315 | 0.014 | 24.201 | 100.728 | 0.156 | 19.611 | 202834.96 | 0.024 | 511.237 | 2.787 | 26.682 | 0.007 | 30.152 | 29.422 | 42.95 | 137.257 | 4.693 | 311.503 |
| 3 | train | 10.487 | 18.336 | 10.386 | 0.014 | 30.66 | 90.71 | 0.163 | 19.177 | 150852.384 | 0.023 | 571.633 | 2.771 | 10.065 | 0.007 | 26.805 | 26.092 | 38.373 | 124.95 | 4.656 | 305.111 |
| 2 | train | 12.214 | 17.914 | 12.114 | 0.013 | 30.309 | 89.131 | 0.337 | 11.812 | 299431.37 | 0.016 | 730.407 | 2.705 | 17.423 | 0.007 | 29.881 | 29.397 | 42.752 | 139.043 | 4.965 | 306.44 |
| 3 | train | 10.553 | 16.423 | 10.454 | 0.012 | 20.665 | 84.188 | 0.37 | 11.298 | 235474.292 | 0.016 | 680.948 | 2.76 | 22.473 | 0.006 | 27.136 | 26.68 | 39.086 | 129.532 | 4.881 | 310.2 |
| 2 | train | 14.394 | 17.228 | 14.295 | 0.012 | 22.583 | 94.157 | 0.418 | 10.836 | 542881.417 | 0.014 | 636.808 | 2.78 | 34.599 | 0.006 | 34.025 | 33.754 | 48.283 | 155.458 | 5.328 | 316.653 |
| 2 | train | 12.801 | 17.991 | 12.7 | 0.013 | 26.299 | 97.108 | 0.28 | 12.585 | 152720.591 | 0.022 | 263.898 | 2.714 | 39.699 | 0.007 | 30.917 | 30.426 | 44.014 | 142.667 | 4.839 | 302.646 |
| 3 | train | 9.212 | 14.224 | 9.114 | 0.01 | 28.52 | 83.597 | 0.362 | 11.217 | 204377.197 | 0.017 | 542.635 | 2.891 | 40.884 | 0.005 | 24.476 | 24.402 | 35.227 | 122.737 | 4.656 | 304.661 |
| 3 | test | 8.417 | 13.314 | 8.32 | 0.011 | 26.133 | 89.47 | 0.4 | 10.874 | 206186.363 | 0.014 | 450.981 | 3.08 | 68.345 | 0.005 | 23.433 | 23.236 | 33.81 | 117.013 | 4.921 | 303.95 |
| 2 | test | 11.73 | 17.726 | 11.628 | 0.013 | 29.726 | 94.06 | 0.176 | 18.816 | 179154.873 | 0.022 | 582.917 | 2.697 | 37.858 | 0.007 | 29.096 | 28.576 | 41.588 | 135.836 | 4.722 | 305.085 |
| 3 | train | 9.513 | 17.096 | 9.413 | 0.013 | 26.559 | 89.091 | 0.178 | 18.61 | 155529.961 | 0.02 | 523.606 | 2.948 | 22.757 | 0.007 | 24.964 | 24.423 | 35.863 | 119.267 | 4.818 | 315.795 |
| 2 | test | 10.709 | 12.598 | 10.62 | 0.008 | 35.824 | 83.233 | 0.301 | 14.874 | 580854.471 | 0.009 | 560.792 | 2.905 | 603.02 | 0.003 | 28.007 | 28.389 | 39.68 | 138.518 | 4.878 | 291.76 |
| 3 | train | 10.249 | 14.555 | 10.151 | 0.011 | 29.518 | 83.968 | 0.363 | 11.264 | 223440.613 | 0.017 | 436.215 | 2.859 | 31.387 | 0.005 | 26.462 | 26.366 | 38.194 | 130.321 | 4.749 | 296.95 |
| 3 | train | 10.103 | 18.776 | 10.004 | 0.016 | 22.578 | 100.941 | 0.15 | 19.6 | 134197.97 | 0.024 | 547.472 | 2.85 | 5.245 | 0.009 | 26.168 | 25.273 | 37.47 | 118.976 | 4.927 | 315.468 |
| 3 | train | 10.219 | 18.79 | 10.118 | 0.016 | 27.163 | 97.572 | 0.149 | 19.695 | 150484.414 | 0.023 | 590.854 | 2.858 | 8.55 | 0.009 | 26.246 | 25.353 | 37.448 | 119.776 | 4.885 | 323.984 |
| 2 | test | 11.088 | 16.39 | 10.988 | 0.011 | 28.77 | 95.969 | 0.317 | 11.91 | 213825.151 | 0.016 | 519.31 | 2.765 | 13.723 | 0.006 | 27.922 | 27.626 | 40.052 | 133.698 | 4.652 | 317.69 |
| 3 | test | 11.613 | 17.223 | 11.51 | 0.012 | 28.192 | 97.692 | 0.313 | 12.035 | 272135.608 | 0.02 | 723.324 | 2.735 | 16.196 | 0.006 | 28.833 | 28.448 | 41.188 | 136.432 | 4.739 | 303.912 |
| 2 | test | 13.517 | 16.635 | 13.422 | 0.01 | 31.64 | 89.436 | 0.338 | 11.748 | 330196.713 | 0.016 | 441.899 | 2.754 | 91.93 | 0.005 | 32.255 | 32.215 | 45.652 | 150.641 | 4.889 | 307.5 |
| 2 | train | 11.691 | 17.328 | 11.591 | 0.012 | 34.351 | 93.677 | 0.283 | 12.436 | 185341.176 | 0.018 | 473.294 | 2.756 | 20.483 | 0.006 | 28.997 | 28.571 | 41.504 | 136.627 | 4.652 | 314.801 |
| 3 | train | 8.959 | 16.546 | 8.862 | 0.013 | 33.08 | 87.125 | 0.277 | 12.351 | 136645.533 | 0.022 | 615.27 | 2.9 | 36.821 | 0.007 | 23.959 | 23.439 | 34.33 | 115.484 | 4.721 | 299.99 |
| 3 | train | 10.786 | 17.134 | 10.687 | 0.013 | 34.173 | 95.37 | 0.187 | 18.404 | 200295.689 | 0.02 | 577.597 | 2.829 | 41.797 | 0.007 | 27.483 | 27.025 | 39.429 | 130.308 | 4.792 | 301.301 |
| 3 | train | 9.315 | 18.187 | 9.215 | 0.017 | 30.842 | 91.244 | 0.149 | 19.638 | 96619.496 | 0.023 | 410.514 | 2.904 | 6.825 | 0.009 | 24.793 | 23.791 | 35.333 | 112.37 | 4.871 | 306.635 |
| 3 | train | 10.914 | 15.896 | 10.814 | 0.011 | 27.678 | 87.481 | 0.343 | 11.549 | 258768.261 | 0.019 | 639.922 | 2.755 | 23.119 | 0.006 | 27.55 | 27.3 | 39.51 | 132.72 | 4.673 | 304.363 |
| 2 | train | 12.935 | 17.787 | 12.833 | 0.012 | 25.122 | 92.048 | 0.183 | 18.758 | 262447.827 | 0.018 | 487.441 | 2.822 | 15.214 | 0.007 | 31.288 | 30.854 | 44.461 | 144.832 | 4.794 | 307.161 |
| 3 | train | 9.809 | 13.999 | 9.711 | 0.011 | 29.694 | 84.912 | 0.385 | 10.948 | 200840.833 | 0.016 | 407.065 | 2.831 | 28.558 | 0.006 | 25.724 | 25.63 | 37.032 | 127.346 | 4.544 | 298.228 |
| 2 | test | 12.594 | 16.695 | 12.496 | 0.012 | 30.849 | 88.444 | 0.311 | 12.05 | 261472.631 | 0.019 | 530.822 | 2.85 | 92.409 | 0.006 | 30.654 | 30.401 | 43.573 | 143.818 | 4.823 | 298.082 |
| 2 | train | 13.274 | 17.256 | 13.173 | 0.012 | 26.512 | 86.739 | 0.372 | 11.32 | 362232.106 | 0.019 | 588.396 | 2.706 | 69.863 | 0.007 | 31.897 | 31.552 | 45.459 | 147.716 | 5.111 | 305.55 |
| 3 | train | 11.686 | 18.918 | 11.584 | 0.014 | 23.383 | 94.892 | 0.178 | 18.849 | 267396.58 | 0.018 | 876.17 | 2.844 | 6.972 | 0.007 | 29.056 | 28.27 | 41.583 | 132.999 | 4.83 | 303.076 |
| 2 | train | 13.38 | 17.982 | 13.277 | 0.013 | 32.433 | 92.606 | 0.21 | 17.797 | 211175.224 | 0.025 | 441.948 | 2.705 | 25.869 | 0.007 | 32.188 | 31.647 | 45.902 | 147.063 | 4.939 | 307.29 |
| 2 | test | 12.611 | 16.406 | 12.515 | 0.011 | 27.161 | 89.417 | 0.368 | 11.309 | 394687.936 | 0.019 | 618.529 | 2.772 | 49.434 | 0.006 | 30.93 | 30.713 | 44.075 | 145.368 | 5.074 | 309.007 |
| 3 | train | 10.94 | 14.626 | 10.843 | 0.01 | 24.467 | 91.532 | 0.351 | 11.42 | 288490.686 | 0.014 | 441.611 | 2.873 | 26.56 | 0.006 | 28 | 27.91 | 40.114 | 136.503 | 4.739 | 289.396 |
| 2 | train | 12.524 | 13.783 | 12.439 | 0.009 | 25.017 | 82.438 | 0.518 | 9.458 | 820385.501 | 0.012 | 576.824 | 2.931 | 54.724 | 0.004 | 31.43 | 31.7 | 44.395 | 149.606 | 5.058 | 309.008 |
| 2 | train | 9.947 | 11.913 | 9.856 | 0.008 | 21.928 | 78.932 | 0.487 | 9.629 | 531539.496 | 0.01 | 474.431 | 2.874 | 97.462 | 0.004 | 26.544 | 26.875 | 37.873 | 133.947 | 4.837 | 290.028 |
| 2 | test | 10.952 | 13.136 | 10.864 | 0.009 | 32.219 | 82.197 | 0.429 | 10.382 | 457294.101 | 0.011 | 612.663 | 2.888 | 172.095 | 0.004 | 28.419 | 28.685 | 40.432 | 139.157 | 5.017 | 288.997 |
| 2 | train | 16.13 | 19.156 | 16.019 | 0.014 | 16.563 | 89.162 | 0.375 | 11.636 | 335393.705 | 0.015 | 415.625 | 2.562 | 39.711 | 0.007 | 36.882 | 36.216 | 52.283 | 161.422 | 5.438 | 308.31 |
| 2 | train | 12.111 | 12.306 | 12.044 | 0.008 | 30.816 | 78.025 | 1.102 | 5.053 | 886120.269 | 0.011 | 599.836 | 2.797 | 406.246 | 0.004 | 31.502 | 32.017 | 44.477 | 150.048 | 5.381 | 269.654 |
| 2 | train | 14.503 | 19.387 | 14.396 | 0.014 | 25.884 | 100.894 | 0.182 | 18.891 | 279244.433 | 0.018 | 589.368 | 2.632 | 38.875 | 0.007 | 34.004 | 33.335 | 48.18 | 152.219 | 5.003 | 315.646 |
| 3 | train | 10.728 | 16.887 | 10.632 | 0.015 | 17.583 | 87.245 | 0.244 | 17.204 | 194367.173 | 0.016 | 590.666 | 2.839 | 9.513 | 0.008 | 27.674 | 26.993 | 39.699 | 126.844 | 5.323 | 308.407 |
| 2 | train | 12.04 | 16.016 | 11.943 | 0.011 | 34.876 | 91.073 | 0.327 | 11.776 | 256687.582 | 0.016 | 522.115 | 2.77 | 75.026 | 0.006 | 29.886 | 29.704 | 42.761 | 141.55 | 4.889 | 301.287 |
| 2 | train | 10.681 | 15.062 | 10.585 | 0.011 | 25.85 | 83.987 | 0.335 | 11.612 | 211040.724 | 0.015 | 393.645 | 2.783 | 25.853 | 0.006 | 27.212 | 27.115 | 38.855 | 132.89 | 4.688 | 311.386 |
| 3 | train | 6.964 | 14.308 | 6.877 | 0.014 | 27.136 | 83.43 | 0.177 | 18.961 | 76934.977 | 0.02 | 443.362 | 3.201 | 6.468 | 0.006 | 20.401 | 19.716 | 29.359 | 96.496 | 4.848 | 291.22 |
| 3 | train | 9.783 | 15.387 | 9.685 | 0.011 | 39.477 | 99.332 | 0.21 | 17.54 | 163165.939 | 0.013 | 565.94 | 2.813 | 63.827 | 0.006 | 25.823 | 25.541 | 37.32 | 126.167 | 4.7 | 292.135 |
| 2 | test | 12.532 | 14.891 | 12.44 | 0.009 | 34.881 | 86.833 | 0.39 | 10.934 | 343195.536 | 0.011 | 453.676 | 2.857 | 217.008 | 0.005 | 31.062 | 31.188 | 44.029 | 147.384 | 4.88 | 286.297 |
| 2 | train | 14.93 | 19.978 | 14.819 | 0.014 | 26.001 | 102.787 | 0.159 | 19.835 | 273263.088 | 0.026 | 514.713 | 2.694 | 18.315 | 0.007 | 34.697 | 33.936 | 49.171 | 153.453 | 4.993 | 311.183 |
| 3 | test | 7.803 | 11.534 | 7.712 | 0.009 | 27.487 | 81.184 | 0.491 | 9.825 | 361299.628 | 0.013 | 827.264 | 3.015 | 23.986 | 0.004 | 22.302 | 22.498 | 32.562 | 116.779 | 4.981 | 290.98 |
| 3 | test | 8.313 | 10.761 | 8.222 | 0.009 | 19.554 | 66.292 | 1.112 | 5.241 | 552728.306 | 0.013 | 716.681 | 3.006 | 23.749 | 0.004 | 23.485 | 23.691 | 34.146 | 121.755 | 5.302 | 280.145 |
| 2 | test | 11.506 | 13.301 | 11.417 | 0.008 | 32.257 | 75.735 | 0.35 | 13.911 | 542159.909 | 0.01 | 435.023 | 2.738 | 82.336 | 0.003 | 29.356 | 29.706 | 41.7 | 142.694 | 5.249 | 291.193 |
| 3 | test | 8.932 | 10.343 | 8.834 | 0.009 | 30.577 | 74.461 | 0.343 | 13.849 | 376170.245 | 0.012 | 438.536 | 2.854 | 115.923 | 0.005 | 24.53 | 24.898 | 35.112 | 126.218 | 4.662 | 271.324 |
| 3 | test | 8.632 | 15.917 | 8.535 | 0.012 | 27.492 | 93.575 | 0.308 | 11.944 | 167792.161 | 0.019 | 663.429 | 2.909 | 25.369 | 0.006 | 23.213 | 22.97 | 33.619 | 115.261 | 4.798 | 302.713 |
| 2 | train | 10.751 | 14.507 | 10.654 | 0.01 | 24.353 | 86.121 | 0.352 | 11.37 | 213175.272 | 0.016 | 384.359 | 2.807 | 23.525 | 0.006 | 27.599 | 27.48 | 39.546 | 134.705 | 4.735 | 296.358 |
| 2 | train | 13.609 | 17.476 | 13.506 | 0.012 | 28.422 | 95.033 | 0.199 | 18.107 | 224126.124 | 0.016 | 392.931 | 2.791 | 144.002 | 0.006 | 32.522 | 32.25 | 46.207 | 149.895 | 5.02 | 317.989 |
| 2 | train | 12.137 | 15.837 | 12.042 | 0.011 | 26.732 | 91.472 | 0.338 | 11.65 | 231933.553 | 0.017 | 490.639 | 2.797 | 79.78 | 0.006 | 29.983 | 29.829 | 42.778 | 142.619 | 4.878 | 304.293 |
| 2 | train | 13.089 | 15.41 | 12.998 | 0.011 | 26.475 | 89.891 | 0.359 | 11.359 | 248726.799 | 0.017 | 288.546 | 2.671 | 84.482 | 0.005 | 31.782 | 31.782 | 44.949 | 149.112 | 4.901 | 302.243 |
| 3 | test | 8.735 | 17.408 | 8.639 | 0.017 | 27.415 | 93.898 | 0.151 | 19.567 | 106490.468 | 0.021 | 477.001 | 2.946 | 10.352 | 0.009 | 23.798 | 22.794 | 33.938 | 108.569 | 4.871 | 298.067 |
| 2 | train | 10.143 | 12.193 | 10.05 | 0.008 | 25.699 | 82.862 | 0.449 | 10.109 | 565991.536 | 0.01 | 572.2 | 2.841 | 209.496 | 0.004 | 26.97 | 27.314 | 38.39 | 135.306 | 4.84 | 290.36 |
| 2 | train | 12.168 | 15.513 | 12.076 | 0.011 | 24.704 | 82.175 | 0.359 | 11.336 | 275833.449 | 0.01 | 417.234 | 2.707 | 46.896 | 0.005 | 30.323 | 30.21 | 43.056 | 144.013 | 4.954 | 298.672 |
| 3 | train | 8.982 | 14.683 | 8.883 | 0.011 | 31.524 | 83.705 | 0.329 | 11.661 | 177487.186 | 0.016 | 539.977 | 2.849 | 9.724 | 0.005 | 24.223 | 24.024 | 34.999 | 121.021 | 4.638 | 300.956 |
| 2 | train | 12.501 | 16.463 | 12.402 | 0.011 | 24.213 | 86.45 | 0.313 | 12.004 | 213896.383 | 0.02 | 302.61 | 2.803 | 70.421 | 0.006 | 30.666 | 30.398 | 43.625 | 143.895 | 4.914 | 308.448 |
| 3 | test | 9.186 | 13.946 | 9.087 | 0.01 | 27.982 | 81.699 | 0.349 | 11.378 | 204267.799 | 0.015 | 522.194 | 2.87 | 24.684 | 0.006 | 24.652 | 24.595 | 35.442 | 123.588 | 4.481 | 296.824 |
| 3 | train | 9.533 | 16.786 | 9.435 | 0.013 | 25.595 | 101.476 | 0.296 | 12.161 | 201467.398 | 0.021 | 787.246 | 2.833 | 33.447 | 0.007 | 25.139 | 24.572 | 36.072 | 119.972 | 4.777 | 298.299 |
| 2 | train | 10.788 | 13.981 | 10.691 | 0.01 | 32.66 | 89.772 | 0.236 | 16.758 | 225975.616 | 0.013 | 303.009 | 2.775 | 60.604 | 0.005 | 27.612 | 27.619 | 39.449 | 135.12 | 4.783 | 292.446 |
| 2 | train | 12.907 | 20.004 | 12.8 | 0.016 | 35.125 | 93.798 | 0.105 | 22.031 | 57539.863 | 0.03 | 268.952 | 2.737 | 19.556 | 0.008 | 31.228 | 30.217 | 44.386 | 137.491 | 4.806 | 309.236 |
| 2 | train | 12.474 | 15.197 | 12.38 | 0.011 | 24.986 | 82.851 | 0.401 | 10.816 | 333869.592 | 0.016 | 474.989 | 2.759 | 133.236 | 0.005 | 30.791 | 30.728 | 43.846 | 145.81 | 5.086 | 302.153 |
| 2 | test | 13.456 | 16.428 | 13.36 | 0.011 | 24.243 | 96.831 | 0.217 | 17.547 | 235359.734 | 0.017 | 301.455 | 2.715 | 98.431 | 0.006 | 32.559 | 32.337 | 46.102 | 150.863 | 4.961 | 301.564 |
| 3 | test | 9.849 | 16.8 | 9.748 | 0.012 | 24.79 | 87.42 | 0.184 | 18.455 | 117547.016 | 0.019 | 402.261 | 2.825 | 10.191 | 0.006 | 25.622 | 25.239 | 36.936 | 122.973 | 4.694 | 302.901 |
| 3 | train | 6.227 | 13.396 | 6.142 | 0.014 | 29.596 | 80.552 | 0.286 | 12.436 | 111420.361 | 0.016 | 545.512 | 3.103 | 10.283 | 0.006 | 18.795 | 18.344 | 27.078 | 92.711 | 4.643 | 288.831 |
| 2 | test | 14.072 | 17.165 | 13.975 | 0.011 | 40.404 | 92.469 | 0.317 | 12.087 | 235037.097 | 0.017 | 325.357 | 2.774 | 29.594 | 0.006 | 33.142 | 33.032 | 46.871 | 153.063 | 4.922 | 302.455 |
| 2 | train | 15.76 | 18.636 | 15.656 | 0.012 | 35.815 | 101.317 | 0.186 | 18.748 | 222683.101 | 0.02 | 365.174 | 2.567 | 108.784 | 0.006 | 36.119 | 35.757 | 50.925 | 161.294 | 4.991 | 308.234 |
| 2 | train | 13.476 | 16.665 | 13.381 | 0.011 | 22.797 | 92.726 | 0.327 | 11.87 | 229442.444 | 0.017 | 316.941 | 2.812 | 103.08 | 0.006 | 32.45 | 32.284 | 46.008 | 150.766 | 4.862 | 316.344 |
| 2 | test | 12.415 | 18.336 | 12.313 | 0.013 | 35.06 | 91.673 | 0.17 | 19.087 | 181445.264 | 0.02 | 481.313 | 2.716 | 50.767 | 0.007 | 30.287 | 29.723 | 43.199 | 139.888 | 4.703 | 311.309 |
| 2 | test | 10.333 | 11.934 | 10.242 | 0.009 | 40.154 | 87.007 | 0.446 | 10.026 | 399989.086 | 0.009 | 424.099 | 2.779 | 166.801 | 0.004 | 27.076 | 27.493 | 38.555 | 135.453 | 4.908 | 299.351 |
| 2 | test | 10.052 | 12.001 | 9.961 | 0.008 | 34.201 | 81.756 | 0.464 | 9.912 | 593974.562 | 0.013 | 829.376 | 2.866 | 241.428 | 0.004 | 26.833 | 27.166 | 38.335 | 134.781 | 4.928 | 294.851 |
| 3 | train | 10.038 | 16.61 | 9.94 | 0.013 | 26.002 | 87.005 | 0.185 | 18.409 | 152147.641 | 0.016 | 451.397 | 2.914 | 23.703 | 0.006 | 26.017 | 25.547 | 37.418 | 124.451 | 4.829 | 298.505 |
| 3 | test | 8.54 | 16.73 | 8.441 | 0.014 | 29.464 | 88.255 | 0.265 | 12.576 | 146794.299 | 0.019 | 545.556 | 2.983 | 6.807 | 0.007 | 23.033 | 22.495 | 33.025 | 110.443 | 4.853 | 300.941 |
| 2 | train | 10.406 | 13.204 | 10.309 | 0.009 | 29.165 | 78.934 | 0.389 | 10.834 | 249851.924 | 0.016 | 413.123 | 2.849 | 31.559 | 0.005 | 27.086 | 27.231 | 38.653 | 134.407 | 4.748 | 298.703 |
| 2 | test | 12.359 | 16.019 | 12.262 | 0.011 | 23.427 | 92.387 | 0.328 | 11.848 | 313044.641 | 0.018 | 405.887 | 2.901 | 19.596 | 0.005 | 30.222 | 30.152 | 43.076 | 143.599 | 4.952 | 309.638 |
| 2 | train | 10.959 | 14.638 | 10.863 | 0.01 | 35.687 | 86.651 | 0.227 | 17.103 | 258586.708 | 0.017 | 491.112 | 2.914 | 59.754 | 0.005 | 27.813 | 27.776 | 39.796 | 135.543 | 4.768 | 295.82 |
| 2 | train | 10.972 | 15.038 | 10.873 | 0.01 | 38.486 | 85.705 | 0.339 | 11.617 | 303440.164 | 0.017 | 614.269 | 2.875 | 17.692 | 0.005 | 27.932 | 27.825 | 40.022 | 135.869 | 4.687 | 306.57 |
| 2 | test | 10.818 | 14.227 | 10.72 | 0.01 | 24.357 | 83.602 | 0.363 | 11.261 | 293940.099 | 0.016 | 472.752 | 2.766 | 59.159 | 0.005 | 27.428 | 27.497 | 39.12 | 134.967 | 4.767 | 297.768 |
| 2 | test | 11.664 | 15.579 | 11.567 | 0.011 | 34.966 | 88.564 | 0.327 | 11.779 | 251607.384 | 0.019 | 542.23 | 2.76 | 41.479 | 0.005 | 29.132 | 29.026 | 41.517 | 139.369 | 4.891 | 293.497 |
| 2 | train | 12.806 | 16.743 | 12.707 | 0.012 | 30.529 | 92.418 | 0.198 | 18.153 | 198557.543 | 0.019 | 398.255 | 2.803 | 50.029 | 0.006 | 31.12 | 30.878 | 44.233 | 144.956 | 4.962 | 303.238 |
| 2 | train | 11.639 | 14.658 | 11.547 | 0.01 | 37.368 | 87.421 | 0.363 | 11.289 | 300149.144 | 0.014 | 504.6 | 2.784 | 78.347 | 0.005 | 29.253 | 29.307 | 41.671 | 141 | 4.907 | 289.867 |
| 3 | train | 10.017 | 14.896 | 9.918 | 0.011 | 36.866 | 87.045 | 0.326 | 11.722 | 270847.284 | 0.015 | 843.379 | 2.878 | 79.595 | 0.006 | 26.088 | 25.867 | 37.521 | 128.576 | 4.507 | 313.967 |
| 2 | test | 12.189 | 15.782 | 12.094 | 0.011 | 39.231 | 91.021 | 0.328 | 11.742 | 224258.475 | 0.016 | 372.516 | 2.796 | 99.855 | 0.005 | 30.111 | 29.985 | 42.89 | 142.689 | 4.86 | 310.981 |
| 3 | train | 8.625 | 10.043 | 8.53 | 0.008 | 31.09 | 77.611 | 0.503 | 9.414 | 495586.914 | 0.009 | 553.896 | 2.914 | 366.721 | 0.004 | 24.473 | 24.942 | 34.961 | 126.93 | 4.843 | 267.468 |
| 2 | train | 11.344 | 14.382 | 11.251 | 0.01 | 42.757 | 81.781 | 0.368 | 11.106 | 274047.776 | 0.015 | 483.398 | 2.779 | 91.026 | 0.004 | 28.858 | 28.905 | 41.176 | 139.696 | 4.896 | 297.56 |
| 3 | train | 8.564 | 12.821 | 8.473 | 0.009 | 29.186 | 88.267 | 0.407 | 10.693 | 322589.161 | 0.014 | 687.064 | 2.934 | 30.109 | 0.004 | 23.779 | 23.886 | 34.5 | 121.569 | 4.892 | 297.112 |
| 3 | test | 7.995 | 9.883 | 7.899 | 0.007 | 34.123 | 79.909 | 0.481 | 9.59 | 431068.826 | 0.008 | 542.231 | 2.928 | 16.214 | 0.004 | 22.867 | 23.412 | 33.032 | 121.588 | 4.867 | 284.259 |
| 3 | test | 6.381 | 9.135 | 6.288 | 0.007 | 37.795 | 67.314 | 0.54 | 9.186 | 348595.021 | 0.008 | 591.67 | 2.95 | 45.696 | 0.002 | 19.653 | 20.062 | 28.728 | 108.575 | 4.54 | 269.349 |
| 3 | train | 9.007 | 10.821 | 8.911 | 0.009 | 22.675 | 69.897 | 0.567 | 8.964 | 592787.701 | 0.012 | 676.326 | 2.894 | 27.568 | 0.006 | 24.689 | 24.934 | 35.381 | 126.559 | 4.759 | 287.495 |
| 3 | train | 8.314 | 14.244 | 8.216 | 0.011 | 22.263 | 80.497 | 0.155 | 24.107 | 181915.495 | 0.018 | 605.123 | 2.968 | 49.872 | 0.005 | 23.002 | 22.853 | 33.209 | 116.408 | 4.671 | 290.299 |
| 3 | train | 9.509 | 14.507 | 9.41 | 0.01 | 38.801 | 79.503 | 0.217 | 17.287 | 187751.346 | 0.015 | 637.226 | 2.941 | 45.295 | 0.005 | 25.112 | 24.993 | 36.166 | 124.802 | 4.574 | 296.751 |
| 3 | test | 7.369 | 15.292 | 7.276 | 0.013 | 34.031 | 87.66 | 0.187 | 18.361 | 109422.503 | 0.019 | 553.812 | 2.844 | 6.59 | 0.007 | 20.745 | 20.355 | 29.926 | 102.961 | 4.796 | 295.203 |
| 3 | test | 7.896 | 9.098 | 7.803 | 0.007 | 35.303 | 74.395 | 0.527 | 9.154 | 739210.223 | 0.005 | 753.388 | 2.899 | 178.412 | 0.003 | 23.53 | 24.145 | 33.415 | 124.06 | 4.846 | 280.956 |
| 3 | train | 10.421 | 16.47 | 10.321 | 0.012 | 32.2 | 93.837 | 0.186 | 18.427 | 185953.152 | 0.018 | 543.89 | 2.935 | 10.563 | 0.006 | 26.733 | 26.351 | 38.295 | 128.544 | 4.476 | 306.276 |
| 3 | train | 8.313 | 11.215 | 8.214 | 0.009 | 26.339 | 75.356 | 0.492 | 9.617 | 501837.964 | 0.01 | 850.591 | 2.969 | 21.982 | 0.004 | 23.228 | 23.42 | 33.583 | 121.227 | 4.645 | 278.459 |
| 3 | train | 8.151 | 10.387 | 8.055 | 0.008 | 25.39 | 77.742 | 0.998 | 5.365 | 637115.362 | 0.01 | 805.233 | 3.034 | 104.297 | 0.004 | 23.27 | 23.534 | 33.576 | 121.767 | 4.929 | 285.693 |
| 2 | test | 9.443 | 11.258 | 9.349 | 0.009 | 39.149 | 75.684 | 0.448 | 10.082 | 300712.469 | 0.009 | 435.019 | 2.863 | 108.129 | 0.005 | 25.438 | 25.886 | 36.37 | 129.32 | 4.767 | 271.989 |
| 3 | test | 7.528 | 9.398 | 7.431 | 0.008 | 33.562 | 77.153 | 0.527 | 9.255 | 506428.028 | 0.01 | 527.613 | 3.001 | 58.059 | 0.004 | 22.063 | 22.405 | 31.83 | 117.997 | 4.695 | 275.092 |
| 3 | train | 8.875 | 10.177 | 8.783 | 0.008 | 37.029 | 73.798 | 0.495 | 9.544 | 430460.473 | 0.008 | 497.889 | 3.03 | 170.546 | 0.003 | 25.115 | 25.603 | 36.077 | 128.962 | 4.961 | 268.412 |
| 3 | train | 8.077 | 14.11 | 7.978 | 0.011 | 36.213 | 81.949 | 0.342 | 11.404 | 190580.816 | 0.014 | 800.03 | 2.89 | 33.541 | 0.005 | 22.384 | 22.306 | 32.31 | 114.272 | 4.721 | 293.615 |
| 3 | train | 7.29 | 14.881 | 7.196 | 0.012 | 26.768 | 80.6 | 0.304 | 12.027 | 112815.134 | 0.022 | 540.732 | 2.958 | 8.006 | 0.006 | 20.732 | 20.442 | 29.943 | 104.173 | 4.736 | 290.338 |
| 3 | train | 8.074 | 16.242 | 7.978 | 0.014 | 26.733 | 91.436 | 0.174 | 18.775 | 161482.526 | 0.018 | 586.883 | 2.995 | 6.165 | 0.007 | 22.365 | 21.793 | 32.167 | 107.526 | 4.846 | 294.902 |
| 3 | test | 10.501 | 18.119 | 10.402 | 0.014 | 23.205 | 92.692 | 0.165 | 19.111 | 206905.511 | 0.021 | 671.17 | 2.838 | 13.801 | 0.007 | 26.932 | 26.261 | 38.513 | 125.628 | 4.684 | 303.85 |
| 2 | test | 12.931 | 16.434 | 12.836 | 0.011 | 22.985 | 108.403 | 0.371 | 11.285 | 524204.834 | 0.012 | 657.892 | 2.747 | 13.42 | 0.005 | 31.326 | 31.217 | 44.575 | 147.55 | 5.066 | 297.456 |
| 3 | train | 11.23 | 16.627 | 11.131 | 0.012 | 23.489 | 93.065 | 0.207 | 17.774 | 204080.65 | 0.019 | 509.611 | 2.743 | 17.959 | 0.006 | 28.214 | 27.86 | 40.4 | 133.881 | 4.766 | 298.995 |
| 3 | train | 7.054 | 9.619 | 6.954 | 0.008 | 26.007 | 67.009 | 0.372 | 13.369 | 475929.801 | 0.008 | 623.186 | 2.884 | 42.839 | 0.003 | 21.124 | 21.444 | 30.599 | 113.683 | 4.678 | 294.902 |
| 3 | test | 7.184 | 11.124 | 7.085 | 0.008 | 34.159 | 76.074 | 0.47 | 9.917 | 307203.474 | 0.008 | 733.064 | 2.955 | 26.815 | 0.003 | 20.937 | 21.136 | 30.282 | 111.666 | 4.603 | 278.912 |
| 3 | train | 9.383 | 10.907 | 9.288 | 0.008 | 33.395 | 78.324 | 0.475 | 9.785 | 492680.116 | 0.012 | 577.658 | 2.944 | 158.466 | 0.004 | 25.706 | 26.06 | 36.741 | 130.907 | 4.73 | 277.74 |
| 3 | test | 8.511 | 10.539 | 8.415 | 0.008 | 35.146 | 75.791 | 0.532 | 9.176 | 550484.382 | 0.008 | 838.816 | 2.853 | 69.104 | 0.004 | 23.79 | 24.187 | 34.158 | 124.026 | 4.938 | 278.205 |
| 3 | train | 9.348 | 14.179 | 9.25 | 0.01 | 25.823 | 96.541 | 0.427 | 10.604 | 549694.961 | 0.013 | 1076.355 | 2.94 | 12.095 | 0.005 | 24.825 | 24.736 | 35.763 | 124.344 | 4.607 | 298.65 |
| 3 | train | 9.382 | 16.833 | 9.284 | 0.014 | 32.782 | 87.751 | 0.266 | 12.546 | 103193.087 | 0.022 | 468.094 | 2.616 | 11.039 | 0.007 | 25.063 | 24.421 | 35.979 | 118.507 | 4.92 | 313.472 |
| 3 | test | 7.46 | 10.846 | 7.363 | 0.008 | 35.483 | 87.221 | 0.467 | 9.915 | 459142.465 | 0.01 | 1078.328 | 2.921 | 84.224 | 0.003 | 21.687 | 21.9 | 31.525 | 115.629 | 4.674 | 286.184 |
| 3 | test | 8.157 | 10.171 | 8.058 | 0.008 | 34.179 | 78.228 | 0.837 | 5.815 | 581400.145 | 0.01 | 781.041 | 2.894 | 65.627 | 0.005 | 22.915 | 23.232 | 32.911 | 120.953 | 4.579 | 284.543 |
| 3 | train | 6.088 | 11.109 | 5.994 | 0.008 | 32.237 | 70.671 | 0.416 | 10.497 | 234653.318 | 0.011 | 745.026 | 2.973 | 66.723 | 0.004 | 18.765 | 18.934 | 27.336 | 103.069 | 4.486 | 277.284 |
| 2 | train | 9.405 | 10.927 | 9.311 | 0.008 | 36.398 | 76.61 | 0.339 | 13.88 | 647007.311 | 0.007 | 668.216 | 2.893 | 167.933 | 0.004 | 25.666 | 26.077 | 36.652 | 130.673 | 4.911 | 278.622 |
| 3 | train | 8.313 | 11.534 | 8.212 | 0.009 | 32.871 | 85.437 | 0.405 | 10.562 | 215016.57 | 0.013 | 455.87 | 2.956 | 30.972 | 0.004 | 23.029 | 23.18 | 33.208 | 119.595 | 4.478 | 288.426 |
| 3 | train | 9.522 | 14.424 | 9.424 | 0.01 | 21.402 | 87.593 | 0.335 | 11.556 | 186065.538 | 0.017 | 408.673 | 2.901 | 30.927 | 0.005 | 25.175 | 25.027 | 36.264 | 124.993 | 4.583 | 300.312 |
| 2 | train | 8.482 | 9.459 | 8.393 | 0.007 | 32.056 | 68.458 | 0.918 | 5.507 | 564945.944 | 0.005 | 628.699 | 2.721 | 546.338 | 0.003 | 24.961 | 25.61 | 35.555 | 129.175 | 4.856 | 274.305 |
| 2 | train | 8.963 | 9.841 | 8.877 | 0.007 | 36.206 | 68.532 | 0.53 | 9.243 | 541513.637 | 0.008 | 469.351 | 2.801 | 736.061 | 0.003 | 25.877 | 26.459 | 36.811 | 131.939 | 4.837 | 253.598 |
| 3 | train | 8.081 | 8.981 | 8.005 | 0.006 | 35.624 | 65.839 | 0.956 | 5.399 | 593029.389 | 0.007 | 667.346 | 2.543 | 869.121 | 0.003 | 24.623 | 25.369 | 34.899 | 127.764 | 4.747 | 246.275 |
| 3 | test | 5.021 | 9.387 | 4.941 | 0.008 | 36.323 | 66.327 | 0.472 | 10.084 | 293272.591 | 0.008 | 967.074 | 2.812 | 74.28 | 0.004 | 16.92 | 17.268 | 24.963 | 96.891 | 4.645 | 258.958 |
| 3 | test | 6.28 | 9.154 | 6.187 | 0.007 | 29.425 | 66.755 | 0.589 | 8.876 | 370693.301 | 0.009 | 618.089 | 3.076 | 33.966 | 0.003 | 19.536 | 19.911 | 28.53 | 108.179 | 4.735 | 278.99 |
| 2 | train | 8.583 | 9.455 | 8.499 | 0.007 | 38.868 | 71.386 | 0.952 | 5.362 | 687497.54 | 0.007 | 658.015 | 2.661 | 459.352 | 0.003 | 25.077 | 25.702 | 35.497 | 129.66 | 4.694 | 250.961 |
| 3 | train | 9.024 | 15.141 | 8.925 | 0.012 | 20.885 | 85.671 | 0.308 | 11.885 | 203936.354 | 0.019 | 708.508 | 2.921 | 22.592 | 0.006 | 24.082 | 23.794 | 34.611 | 119.229 | 4.547 | 294.255 |
| 3 | test | 10.138 | 14.384 | 10.039 | 0.011 | 35.581 | 84.779 | 0.34 | 11.467 | 226018.546 | 0.017 | 528.763 | 2.795 | 60.079 | 0.006 | 26.403 | 26.257 | 37.863 | 130.134 | 4.487 | 295.328 |
| 3 | test | 8.288 | 16.018 | 8.19 | 0.013 | 31.285 | 86.399 | 0.289 | 12.199 | 166236.974 | 0.019 | 552.214 | 2.835 | 9.395 | 0.007 | 22.664 | 22.305 | 32.653 | 111.606 | 4.812 | 303.294 |

A,original_glrlm_ShortRunHighGrayLevelEmphasis.B,original_glszm_SmallAreaHighGrayLevelEmphasis C,additivegaussiannoise_glrlm_ShortRunHighGrayLevelEmphasis.D,binomialblurimage_gldm_SmallDependenceEmphasis.E.curvatureflow_firstorder_Minimum.F,log_firstorder_log-sigma-0-5-mm-3D-Maximum.G,log_glrlm_log-sigma-1-0-mm-3D-LongRunLowGrayLevelEmphasis H,log_glrlm_log-sigma-1-0-mm-3D-ShortRunHighGrayLevelEmphasis.I,log_gldm_log-sigma-4-0-mm-3D-DependenceNonUniformity.J,wavelet_ngtdm_wavelet-LHL-Contrast.K,wavelet_ngtdm_wavelet-LLL-Busyness.L,normalize_firstorder_Range.M,normalize_glrlm_RunVariance.N,boxmean_glszm_ZonePercentage.O,specklenoise_glcm_DifferenceVariance.P,specklenoise_glrlm_GrayLevelVariance.Q,specklenoise_glszm_GrayLevelVariance.R,specklenoise_glszm_SmallAreaHighGrayLevelEmphasis.S,recursivegaussian_glszm_ZoneEntropy.T,shotnoise_firstorder_Maximum.
