# Supplementary material for: Diagnostic of fatty liver using radiomics and deep learning models on non-contrast abdominal CT
Source: PLoS One. 2025 Feb 13;20(2):e0310938. doi: 10.1371/journal.pone.0310938 (PMC11825062; doi:10.1371/journal.pone.0310938)
Supplement: S3 Table — (DOCX) [file pone.0310938.s005.docx]

**S3 Table.** **The mean of 5 - fold cross - validation.**

| Method(2d) | auc_test | auc_95%CI_lower_test | auc_95%CI_upper_test | sensitivity_test | specificity_test | accuracy_test | precision_test | f1Score_test |
| --- | --- | --- | --- | --- | --- | --- | --- | --- |
| Z_score_scaler>>AdaBoost | 0.955 | 0.944 | 0.987 | 0.849 | 0.942 | 0.838 | 0.853 | 0.849 |
| Z_score_scaler>>BDT | 0.961 | 0.954 | 0.99 | 0.868 | 0.951 | 0.862 | 0.874 | 0.87 |
| Z_score_scaler>>DT | 0.942 | 0.921 | 0.984 | 0.85 | 0.944 | 0.843 | 0.855 | 0.851 |
| Z_score_scaler>>GP | 0.96 | 0.952 | 0.991 | 0.876 | 0.952 | 0.867 | 0.881 | 0.878 |
| Z_score_scaler>>GBDT | 0.956 | 0.943 | 0.989 | 0.858 | 0.946 | 0.849 | 0.86 | 0.858 |
| Z_score_scaler>>KNN | 0.939 | 0.918 | 0.98 | 0.83 | 0.936 | 0.819 | 0.834 | 0.831 |
| Z_score_scaler>>LR | 0.938 | 0.917 | 0.979 | 0.782 | 0.922 | 0.78 | 0.796 | 0.785 |
| Z_score_scaler>>QDA | 0.947 | 0.934 | 0.982 | 0.818 | 0.932 | 0.811 | 0.831 | 0.823 |
| Z_score_scaler>>RF | 0.959 | 0.95 | 0.989 | 0.863 | 0.949 | 0.856 | 0.869 | 0.864 |
| Z_score_scaler>>SGD | 0.863 | 0.816 | 0.929 | 0.773 | 0.915 | 0.763 | 0.792 | 0.775 |
| Z_score_scaler>>SVM | 0.96 | 0.954 | 0.989 | 0.873 | 0.95 | 0.859 | 0.873 | 0.873 |
| Z_score_scaler>>XGBOOST | 0.956 | 0.943 | 0.99 | 0.855 | 0.945 | 0.845 | 0.857 | 0.855 |
| Method(3D) | auc_test | auc_95%CI_lower_test | auc_95%CI_upper_test | sensitivity_test | specificity_test | accuracy_test | precision_test | f1Score_test |
| Z_score_scaler>>AdaBoost | 0.954 | 0.944 | 0.986 | 0.851 | 0.942 | 0.837 | 0.856 | 0.852 |
| Z_score_scaler>>BDT | 0.949 | 0.936 | 0.984 | 0.833 | 0.936 | 0.821 | 0.839 | 0.832 |
| Z_score_scaler>>DT | 0.927 | 0.9 | 0.974 | 0.815 | 0.929 | 0.8 | 0.818 | 0.813 |
| Z_score_scaler>>GP | 0.954 | 0.944 | 0.985 | 0.856 | 0.944 | 0.844 | 0.861 | 0.858 |
| Z_score_scaler>>GBDT | 0.953 | 0.941 | 0.986 | 0.85 | 0.943 | 0.838 | 0.853 | 0.849 |
| Z_score_scaler>>KNN | 0.938 | 0.915 | 0.979 | 0.832 | 0.936 | 0.821 | 0.839 | 0.833 |
| Z_score_scaler>>LR | 0.938 | 0.922 | 0.975 | 0.777 | 0.919 | 0.772 | 0.787 | 0.779 |
| Z_score_scaler>>QDA | 0.93 | 0.912 | 0.97 | 0.75 | 0.909 | 0.75 | 0.783 | 0.756 |
| Z_score_scaler>>RF | 0.936 | 0.918 | 0.974 | 0.8 | 0.926 | 0.79 | 0.81 | 0.802 |
| Z_score_scaler>>SGD | 0.887 | 0.849 | 0.945 | 0.814 | 0.926 | 0.791 | 0.814 | 0.81 |
| Z_score_scaler>>SVM | 0.956 | 0.948 | 0.986 | 0.85 | 0.943 | 0.839 | 0.854 | 0.85 |
| Z_score_scaler>>XGBOOST | 0.955 | 0.944 | 0.987 | 0.848 | 0.942 | 0.836 | 0.853 | 0.849 |
