# Supplementary material for: Diagnostic of fatty liver using radiomics and deep learning models on non-contrast abdominal CT
Source: PLoS One. 2025 Feb 13;20(2):e0310938. doi: 10.1371/journal.pone.0310938 (PMC11825062; doi:10.1371/journal.pone.0310938)
Supplement: S4 Table — If there is still a need for explanation during the operation process, you can contact us at 13837141925@163.com. (DOCX) [file pone.0310938.s006.docx]

**S4 Table. Clinical data**

| Label | LFC | age | gender | Glu | ALT | AST | GGT | ALP | TP | ALB | GLB | TBIL | DBIL | IBIL | TC | TG | HDL | LDL |
| --- | --- | --- | --- | --- | --- | --- | --- | --- | --- | --- | --- | --- | --- | --- | --- | --- | --- | --- |
| 0 | 3.363155087 | 44 | 0 | 6.06 | 26 | 21 | 45 | 62 | 70.7 | 47.4 | 23.3 | 13.61 | 6.34 | 7.3 | 4.29 | 0.53 | 1.66 | 2.4 |
| 0 | 2.815299749 | 59 | 0 | 6.56 | 16 | 18 | 18 | 70 | 65 | 45.5 | 19.5 | 8.29 | 3.46 | 4.8 | 4.53 | 1.92 | 0.94 | 2.72 |
| 1 | 6.047471523 | 57 | 1 | 4.92 | 18 | 21 | 10 | 67 | 69 | 47.5 | 21.5 | 20.3 | 8.5 | 11.8 | 4.18 | 0.75 | 1.82 | 2.13 |
| 0 | 0.355419199 | 53 | 1 | 5 | 16 | 25 | 15 | 55 | 71.8 | 46.4 | 25.4 | 20.4 | 7.1 | 13.3 | 4.29 | 0.69 | 1.68 | 2.39 |
| 0 | 3.211826642 | 53 | 0 | 5.02 | 11 | 13 | 15 | 88 | 66.1 | 44.4 | 21.7 | 15.9 | 5.3 | 10.6 | 4.38 | 1.77 | 1.27 | 2.72 |
| 1 | 6.848315239 | 48 | 1 | 4.9 | 16 | 18 | 16 | 69 | 71.4 | 44.5 | 26.9 | 3.6 | 1.9 | 1.7 | 5.28 | 0.85 | 1.5 | 3.65 |
| 1 | 8.920066198 | 57 | 1 | 5.55 | 42 | 31 | 141 | 74 | 67.5 | 47.6 | 19.9 | 7.82 | 3.71 | 4.1 | 5.35 | 0.79 | 1.98 | 2.98 |
| 2 | 20.91312536 | 57 | 0 | 5.04 | 12 | 11 | 15 | 91 | 67 | 44.8 | 22.2 | 7.71 | 3.5 | 4.2 | 4.79 | 2.07 | 0.87 | 3.27 |
| 2 | 22.54013189 | 58 | 1 | 6.77 | 57 | 36 | 56 | 105 | 73.4 | 47 | 26.4 | 8.24 | 3.45 | 4.8 | 6.28 | 1.54 | 1.39 | 4.3 |
| 0 | 2.997053464 | 43 | 1 | 4.48 | 13 | 18 | 8 | 56 | 70.2 | 49.1 | 21.1 | 6.01 | 3.19 | 2.8 | 3.08 | 0.34 | 1.93 | 1.13 |
| 1 | 7.633492788 | 59 | 0 | 5.15 | 70 | 41 | 175 | 117 | 72.8 | 48.6 | 24.2 | 11.47 | 5.01 | 6.5 | 5.56 | 3.97 | 1.02 | 3.21 |
| 2 | 24.64467303 | 49 | 0 | 5.66 | 64 | 37 | 32 | 102 | 75.5 | 48 | 27.5 | 15.17 | 6.21 | 9 | 5.06 | 2.01 | 1.09 | 3.26 |
| 1 | 8.812322934 | 48 | 0 | 5.21 | 14 | 18 | 17 | 103 | 70.6 | 43.3 | 27.3 | 17.84 | 8.04 | 9.8 | 2.83 | 0.76 | 1.02 | 1.6 |
| 0 | 1.61249129 | 52 | 1 | 5.09 | 15 | 19 | 9 | 54 | 69.5 | 43.3 | 26.2 | 7 | 3.5 | 3.5 | 5.01 | 0.79 | 1.87 | 3.07 |
| 1 | 6.691961924 | 53 | 0 | 7.99 | 23 | 20 | 26 | 68 | 74 | 49.2 | 24.8 | 15.95 | 6.37 | 9.6 | 4.13 | 2.72 | 1 | 2.58 |
| 0 | 4.753998478 | 60 | 0 | 5.35 | 22 | 20 | 53 | 85 | 70.1 | 43.2 | 26.9 | 19.5 | 7.9 | 11.6 | 4 | 1.19 | 1.35 | 2.39 |
| 0 | 0.98216486 | 50 | 1 | 4.71 | 9 | 13 | 11 | 39 | 66.9 | 42.3 | 24.6 | 5.52 | 2.56 | 3 | 4.09 | 0.67 | 1.68 | 2.26 |
| 1 | 8.328226566 | 50 | 0 | 5.86 | 16 | 25 | 44 | 96 | 71.1 | 50.6 | 20.5 | 7.91 | 3.76 | 4.2 | 5.18 | 0.77 | 1.28 | 3.31 |
| 0 | 1.784952164 | 53 | 1 | 4.78 | 23 | 24 | 42 | 77 | 66.4 | 47.8 | 18.6 | 12.8 | 5.5 | 7.3 | 4.74 | 1.19 | 1.54 | 2.88 |
| 1 | 8.71088473 | 58 | 1 | 6.98 | 20 | 21 | 100 | 85 | 75 | 49.9 | 25.1 | 19.2 | 7.1 | 12.1 | 5.72 | 2.09 | 1.13 | 3.65 |
| 0 | 4.901552598 | 55 | 0 | 5.84 | 10 | 14 | 18 | 75 | 78 | 48.1 | 29.9 | 11.82 | 5.55 | 6.3 | 4.84 | 1.28 | 1.15 | 3.31 |
| 0 | 2.475127737 | 58 | 1 | 5.24 | 12 | 19 | 10 | 72 | 70.2 | 47.4 | 22.8 | 9.12 | 3.62 | 5.5 | 5.47 | 1.46 | 1.41 | 3.34 |
| 1 | 12.664958 | 66 | 1 | 5.71 | 329 | 204 | 87 | 130 | 71.4 | 46.6 | 24.8 | 19.71 | 10.12 | 9.6 | 5.03 | 0.96 | 1.92 | 2.5 |
| 2 | 14.82020728 | 79 | 1 | 7.49 | 30 | 46 | 95 | 124 | 75.4 | 44.7 | 30.7 | 14.1 | 4.7 | 9.4 | 3.56 | 1.66 | 1.2 | 1.7 |
| 0 | 3.191734751 | 51 | 1 | 4.82 | 12 | 16 | 10 | 72 | 70 | 43.4 | 26.6 | 10.29 | 4.66 | 5.6 | 3.95 | 0.61 | 0.96 | 2.75 |
| 0 | 2.028309087 | 59 | 0 | 5.15 | 18 | 21 | 40 | 57 | 64.3 | 44.2 | 20.1 | 9.18 | 4.57 | 4.6 | 3.74 | 0.54 | 1.58 | 1.67 |
| 0 | 1.947618802 | 60 | 0 | 5.39 | 26 | 23 | 38 | 64 | 66.1 | 50.3 | 15.8 | 6.17 | 2.82 | 3.3 | 4.73 | 2.94 | 1.1 | 2.55 |
| 0 | 3.095777273 | 59 | 0 | 5.86 | 14 | 22 | 22 | 87 | 71.2 | 40.8 | 30.4 | 6.81 | 3.06 | 3.8 | 4.64 | 2.39 | 1.21 | 2.56 |
| 1 | 7.71478947 | 44 | 0 | 6.17 | 20 | 16 | 21 | 78 | 73.9 | 48.8 | 25.1 | 12.44 | 4.08 | 8.4 | 5.45 | 2.2 | 1.23 | 3.61 |
| 0 | 4.314027468 | 42 | 0 | 5.07 | 38 | 25 | 85 | 79 | 66.3 | 46.1 | 20.2 | 10.7 | 3.8 | 6.9 | 5.91 | 5.09 | 1.06 | 3.59 |
| 1 | 11.52117459 | 66 | 0 | 6.87 | 15 | 25 | 18 | 94 | 73.4 | 44.9 | 28.5 | 19.88 | 8.57 | 11.3 | 3.59 | 2.1 | 1.17 | 1.57 |
| 0 | 3.905575454 | 76 | 0 | 9.37 | 20 | 20 | 34 | 72 | 72.4 | 50.8 | 21.6 | 12.24 | 4.7 | 7.5 | 4.42 | 1.05 | 1.03 | 3.12 |
| 0 | 3.718303124 | 43 | 0 | 5.21 | 17 | 19 | 56 | 86 | 75.6 | 48.9 | 26.7 | 13.9 | 4.3 | 9.6 | 6.22 | 1.68 | 1.34 | 4.24 |
| 0 | 4.82617871 | 59 | 0 | 5.11 | 7 | 24 | 25 | 52 | 68.8 | 47 | 21.8 | 16.4 | 6.5 | 9.9 | 4.35 | 0.78 | 1.69 | 2.52 |
| 0 | 4.966504812 | 59 | 0 | 4.78 | 24 | 21 | 20 | 67 | 65.6 | 48.8 | 16.8 | 11.93 | 4.37 | 7.6 | 3.84 | 0.98 | 0.97 | 2.51 |
| 0 | 0.231475194 | 63 | 1 | 6.35 | 17 | 23 | 16 | 71 | 77.8 | 43.5 | 34.3 | 8.74 | 3.48 | 5.3 | 6.47 | 1.62 | 1.23 | 4.43 |
| 0 | 3.206389149 | 42 | 0 | 4.7 | 25 | 20 | 15 | 67 | 71.1 | 50.4 | 20.7 | 21.12 | 8.56 | 12.6 | 3.7 | 2.14 | 1.05 | 1.9 |
| 0 | 1.953143756 | 58 | 0 | 5.68 | 29 | 29 | 16 | 83 | 72.6 | 46.1 | 26.5 | 13.83 | 5.98 | 7.8 | 4.45 | 1.22 | 1.1 | 2.84 |
| 0 | 1.153784394 | 58 | 1 | 4.77 | 10 | 16 | 15 | 102 | 69.9 | 47 | 22.9 | 9.3 | 3.1 | 6.2 | 6.21 | 1.7 | 1.48 | 4.09 |
| 0 | 4.075454315 | 47 | 1 | 5.23 | 9 | 15 | 11 | 68 | 67.3 | 43.2 | 24.1 | 8.25 | 3.17 | 5.1 | 6.19 | 1.72 | 1.22 | 3.81 |
| 0 | 4.479239146 | 51 | 0 | 5.64 | 15 | 15 | 39 | 55 | 76.5 | 46.9 | 29.6 | 13.43 | 4.35 | 9.1 | 5.31 | 2.59 | 1.34 | 3.26 |
| 2 | 22.86995061 | 45 | 0 | 8.1 | 30 | 22 | 29 | 71 | 67.6 | 45 | 22.6 | 8.11 | 3.15 | 5 | 4.73 | 4.36 | 1.01 | 2.02 |
| 1 | 5.980994701 | 47 | 1 | 4.46 | 11 | 23 | 15 | 56 | 67.4 | 49.8 | 17.6 | 19.35 | 6.78 | 12.6 | 4.71 | 0.74 | 1.78 | 2.79 |
| 0 | 2.532223145 | 58 | 0 | 4.25 | 17 | 21 | 10 | 64 | 72 | 41.8 | 30.2 | 26.42 | 10.65 | 15.8 | 5.46 | 1.11 | 1.51 | 3.48 |
| 1 | 12.47642899 | 59 | 0 | 8.41 | 31 | 22 | 38 | 73 | 74.2 | 48 | 26.2 | 8.4 | 3.7 | 4.7 | 5.81 | 2.56 | 1.16 | 3.93 |
| 1 | 13.25754706 | 44 | 0 | 5.83 | 22 | 19 | 25 | 77 | 71.8 | 46.6 | 23.2 | 8.59 | 3.92 | 4.7 | 6.23 | 2.08 | 1.22 | 4.19 |
| 1 | 5.14105924 | 57 | 0 | 4.64 | 10 | 16 | 12 | 83 | 72 | 46.1 | 25.9 | 11.47 | 5.03 | 6.4 | 4.9 | 0.74 | 1.38 | 3.15 |
| 1 | 6.496323427 | 62 | 1 | 6.23 | 16 | 19 | 20 | 86 | 78.2 | 47.9 | 30.3 | 9.34 | 4.1 | 5.2 | 7.05 | 0.94 | 1.68 | 4.56 |
| 0 | 0.400099913 | 53 | 1 | 5.25 | 19 | 24 | 36 | 88 | 64.9 | 43.9 | 21 | 20.2 | 7.7 | 12.5 | 4.14 | 1.09 | 1.87 | 2.11 |
| 1 | 5.121684631 | 57 | 1 | 6.54 | 54 | 40 | 271 | 146 | 78 | 46 | 32 | 7.1 | 3.1 | 4 | 4.68 | 4.06 | 1.02 | 2.33 |
| 1 | 9.517853578 | 56 | 0 | 5.06 | 13 | 15 | 18 | 65 | 69.3 | 44 | 25.3 | 8.77 | 4.22 | 4.6 | 4.12 | 1.27 | 1.18 | 2.23 |
| 0 | 1.923678795 | 58 | 0 | 4.68 | 14 | 20 | 17 | 99 | 69.5 | 45.2 | 24.3 | 6.8 | 3.5 | 3.3 | 4.24 | 0.76 | 1.42 | 2.53 |
| 1 | 6.510238806 | 49 | 1 | 4.72 | 16 | 21 | 11 | 80 | 65.1 | 45.2 | 19.9 | 7.11 | 3.9 | 3.2 | 5.35 | 1.24 | 1.75 | 3.22 |
| 0 | 1.692995628 | 59 | 1 | 5.57 | 21 | 31 | 27 | 89 | 73.8 | 46.9 | 26.9 | 13.6 | 3.28 | 10.3 | 7.65 | 2.04 | 1.69 | 4.93 |
| 0 | 2.292546749 | 48 | 1 | 4.69 | 7 | 14 | 12 | 58 | 63.5 | 44 | 19.5 | 8.16 | 3.98 | 4.2 | 4.48 | 0.76 | 1.46 | 2.62 |
| 1 | 6.269600868 | 48 | 0 | 5.06 | 7 | 20 | 16 | 74 | 70.5 | 48.1 | 22.4 | 10.9 | 4.24 | 6.7 | 5.55 | 1.5 | 1.51 | 3.42 |
| 1 | 7.912436644 | 46 | 0 | 5.64 | 52 | 47 | 131 | 83 | 71.1 | 47.5 | 23.6 | 13.22 | 4.42 | 8.8 | 4.11 | 2.04 | 1.12 | 2.31 |
| 1 | 7.044618448 | 58 | 0 | 5.12 | 40 | 18 | 18 | 65 | 70 | 46.1 | 23.9 | 14.86 | 6.23 | 8.6 | 5.62 | 0.97 | 1.21 | 4.06 |
| 1 | 8.186085701 | 52 | 1 | 5.82 | 24 | 22 | 17 | 113 | 70 | 47.7 | 22.3 | 16.98 | 6.68 | 10.3 | 5.04 | 1.62 | 1.11 | 3.38 |
| 1 | 5.002114296 | 58 | 1 | 7.18 | 10 | 17 | 8 | 102 | 75.4 | 43.1 | 32.3 | 16.42 | 6.15 | 10.3 | 5.2 | 1.01 | 1.54 | 3.4 |
| 0 | 4.490056992 | 80 | 0 | 5.46 | 21 | 24 | 44 | 63 | 72.4 | 47.2 | 25.2 | 9.23 | 3.51 | 5.7 | 6.47 | 1.96 | 1.19 | 4.58 |
| 1 | 10.07017215 | 82 | 0 | 7.34 | 17 | 14 | 11 | 57 | 62.5 | 42.1 | 20.4 | 8.81 | 4.1 | 4.7 | 3.19 | 0.85 | 1.32 | 1.64 |
| 0 | 2.447263837 | 65 | 0 | 5.32 | 81 | 46 | 38 | 80 | 63.4 | 44.4 | 19 | 10.2 | 3.8 | 6.4 | 4.07 | 2.33 | 1.14 | 2.09 |
| 1 | 12.12834994 | 41 | 1 | 4.92 | 8 | 12 | 9 | 79 | 73.6 | 45.2 | 28.4 | 7.66 | 3.42 | 4.2 | 4.53 | 1.58 | 1.71 | 2.36 |
| 1 | 5.81152614 | 73 | 0 | 6.46 | 15 | 15 | 12 | 56 | 69.2 | 43.1 | 26.1 | 10 | 4.18 | 5.8 | 2.17 | 0.82 | 0.94 | 1 |
| 0 | 4.282108386 | 47 | 0 | 5.2 | 22 | 21 | 119 | 86 | 69.6 | 46.4 | 23.2 | 12.16 | 4.44 | 7.7 | 5.5 | 5.64 | 1.15 | 2.35 |
| 1 | 7.004493237 | 82 | 0 | 9.17 | 21 | 26 | 78 | 91 | 74.8 | 46.5 | 28.3 | 10.03 | 4.74 | 5.3 | 3.58 | 1.56 | 1.12 | 1.81 |
| 0 | 4.718606472 | 56 | 0 | 4.97 | 10 | 15 | 12 | 89 | 70.1 | 44 | 26.1 | 16.42 | 5.15 | 11.3 | 4.66 | 1.47 | 1.26 | 3.08 |
| 0 | 1.752087514 | 50 | 0 | 5.11 | 17 | 16 | 68 | 73 | 74.7 | 51.4 | 23.3 | 10.21 | 2.95 | 7.3 | 8.34 | 3.18 | 1.38 | 5.8 |
| 1 | 10.96579488 | 51 | 0 | 5.23 | 31 | 25 | 67 | 47 | 64.2 | 49.2 | 15 | 11.4 | 4.32 | 7.1 | 4.4 | 1.57 | 1.05 | 2.86 |
| 0 | 1.240041415 | 54 | 0 | 6.43 | 15 | 17 | 36 | 93 | 68.2 | 47.8 | 20.4 | 14.96 | 6.45 | 8.5 | 4.95 | 1.3 | 1.53 | 3 |
| 0 | 3.051914374 | 60 | 0 | 4.95 | 14 | 19 | 35 | 91 | 69.2 | 46 | 23.2 | 9.81 | 4.96 | 4.8 | 3.79 | 1.14 | 1.53 | 1.72 |
| 1 | 6.752807776 | 39 | 1 | 4.88 | 25 | 17 | 41 | 72 | 77.4 | 47.1 | 30.3 | 9 | 4.2 | 4.8 | 3.39 | 0.68 | 1.48 | 1.76 |
| 0 | 2.227725824 | 49 | 1 | 5.12 | 13 | 19 | 13 | 53 | 68.4 | 48.6 | 19.8 | 6.58 | 3.2 | 3.4 | 4.35 | 0.65 | 1.51 | 2.65 |
| 1 | 6.481934468 | 42 | 0 | 5.72 | 47 | 29 | 39 | 72 | 74.9 | 45.7 | 29.2 | 4 | 1.85 | 2.2 | 6.49 | 4.13 | 1.17 | 3.75 |
| 0 | 2.731816729 | 58 | 1 | 4.8 | 23 | 32 | 15 | 88 | 79.5 | 50.4 | 29.1 | 12.93 | 4.8 | 8.1 | 4.14 | 1.15 | 1.42 | 2.37 |
| 0 | 2.782203595 | 51 | 1 | 5.93 | 12 | 21 | 26 | 69 | 78 | 52.7 | 25.3 | 10.82 | 4.5 | 6.3 | 4.32 | 1.08 | 2.11 | 1.64 |
| 0 | 4.780040582 | 55 | 0 | 5.55 | 11 | 16 | 20 | 69 | 71.5 | 46.3 | 25.2 | 12.18 | 4.06 | 8.1 | 5.06 | 1.23 | 1.2 | 3.31 |
| 1 | 5.51453193 | 56 | 0 | 6.6 | 11 | 17 | 20 | 63 | 70.8 | 48.9 | 21.9 | 20.88 | 7.39 | 13.5 | 4.31 | 1.17 | 0.91 | 2.99 |
| 2 | 20.61038685 | 69 | 1 | 6.63 | 25 | 23 | 25 | 105 | 70 | 44.7 | 25.3 | 7.32 | 3.14 | 4.2 | 3.43 | 0.92 | 1.43 | 1.81 |
| 2 | 17.6113685 | 57 | 1 | 6.17 | 14 | 16 | 24 | 24 | 77.1 | 48.8 | 28.3 | 15.35 | 6.9 | 8.4 | 4.78 | 1.63 | 1.67 | 2.64 |
| 0 | 4.711031159 | 58 | 1 | 5.16 | 44 | 47 | 23 | 60 | 66.1 | 44.7 | 21.4 | 7.5 | 3.3 | 4.2 | 4.53 | 1.85 | 0.99 | 2.92 |
| 1 | 9.761855761 | 56 | 0 | 6.09 | 13 | 17 | 47 | 75 | 68.8 | 44.2 | 24.6 | 10.24 | 4.71 | 5.5 | 4.64 | 3.14 | 0.85 | 2.46 |
| 0 | 1.612586558 | 62 | 0 | 4.99 | 16 | 23 | 29 | 41 | 68.6 | 47 | 21.6 | 21.77 | 7.97 | 13.8 | 5.72 | 1.14 | 1.85 | 3.17 |
| 1 | 9.616509597 | 49 | 1 | 4.7 | 43 | 30 | 33 | 79 | 73.8 | 50 | 23.8 | 7.18 | 3.47 | 3.7 | 5.29 | 1.78 | 1.01 | 3.74 |
| 0 | 4.476401329 | 51 | 1 | 4.88 | 9 | 15 | 13 | 65 | 70.5 | 45 | 25.5 | 7.6 | 3.01 | 4.6 | 5.18 | 1.34 | 1.47 | 3 |
| 1 | 7.101814429 | 60 | 1 | 4.94 | 32 | 24 | 86 | 99 | 67.4 | 45.4 | 22 | 8.1 | 4.2 | 3.9 | 4.09 | 1.23 | 1.92 | 1.98 |
| 1 | 5.423511028 | 45 | 1 | 4.77 | 13 | 19 | 17 | 90 | 67.6 | 44.9 | 22.7 | 9 | 4.7 | 4.3 | 3.7 | 0.69 | 1.35 | 2.17 |
| 2 | 17.76542536 | 80 | 0 | 6.73 | 19 | 20 | 29 | 60 | 73.5 | 45.9 | 27.6 | 9.89 | 4.46 | 5.4 | 4.18 | 3.17 | 1.03 | 2.1 |
| 0 | 3.999335607 | 72 | 0 | 7.81 | 12 | 15 | 8 | 109 | 70.9 | 47.9 | 23 | 6.27 | 3.65 | 2.6 | 2.7 | 1.74 | 0.86 | 1.29 |
| 1 | 7.869740168 | 59 | 0 | 4.85 | 26 | 24 | 15 | 56 | 74.4 | 49.7 | 24.7 | 10.15 | 4.08 | 6.1 | 6.77 | 1.21 | 1.61 | 4.42 |
| 0 | 3.879587253 | 58 | 0 | 5.44 | 20 | 29 | 22 | 61 | 71.3 | 50.4 | 20.9 | 11.27 | 4.31 | 7 | 3.73 | 1.05 | 1.5 | 1.74 |
| 2 | 23.75662549 | 46 | 0 | 9.28 | 30 | 27 | 41 | 66 | 71.7 | 46.4 | 25.3 | 24.61 | 8.02 | 16.6 | 4.72 | 1.21 | 1.32 | 3.08 |
| 0 | 2.94725434 | 42 | 1 | 4.94 | 7 | 11 | 9 | 48 | 69.7 | 45.4 | 24.3 | 7.8 | 3.2 | 4.6 | 5.17 | 0.66 | 1.76 | 3.27 |
| 1 | 7.57193319 | 57 | 0 | 5.35 | 21 | 17 | 52 | 42 | 69.3 | 47.9 | 21.4 | 13.09 | 3.98 | 9.1 | 4.89 | 1.64 | 0.91 | 3 |
| 2 | 26.65988032 | 53 | 1 | 4.82 | 18 | 21 | 24 | 76 | 76 | 47.7 | 28.3 | 8.9 | 3.87 | 5 | 5.38 | 2.52 | 1.33 | 3.41 |
| 1 | 9.203583241 | 67 | 0 | 7.72 | 9 | 17 | 18 | 39 | 76.1 | 50.5 | 25.6 | 15.96 | 4.15 | 11.8 | 4.9 | 2.52 | 1.08 | 2.94 |
| 0 | 4.510001024 | 56 | 1 | 4.96 | 12 | 16 | 13 | 82 | 73.2 | 46.9 | 26.3 | 21.4 | 7.1 | 14.3 | 4.65 | 2.85 | 1.21 | 2.59 |
| 1 | 6.181274335 | 57 | 1 | 5.17 | 12 | 17 | 14 | 85 | 76.7 | 48.9 | 27.8 | 10.7 | 5.3 | 5.4 | 5.5 | 1.2 | 1.59 | 3.62 |
| 0 | 4.944284757 | 48 | 1 | 5.19 | 25 | 24 | 32 | 47 | 66 | 46.1 | 19.9 | 5.6 | 2.8 | 2.8 | 6.73 | 0.97 | 2.1 | 4.49 |
| 2 | 15.93123531 | 47 | 0 | 5.74 | 21 | 19 | 41 | 106 | 71.3 | 49.8 | 21.5 | 7.33 | 2.81 | 4.5 | 5.24 | 2.48 | 0.88 | 3.42 |
| 0 | 4.465339184 | 58 | 1 | 4.66 | 32 | 35 | 15 | 73 | 74.8 | 46.3 | 28.5 | 10.3 | 1.3 | 9 | 3.8 | 0.94 | 1.45 | 1.99 |
| 0 | 3.42833813 | 45 | 1 | 5.41 | 21 | 37 | 12 | 63 | 69.7 | 46 | 23.7 | 12 | 4.7 | 7.3 | 4.9 | 1.9 | 1.25 | 3.01 |
| 1 | 5.706238747 | 52 | 0 | 9.55 | 26 | 22 | 36 | 66 | 64.2 | 42.9 | 21.3 | 11.5 | 5 | 6.5 | 3.76 | 0.99 | 1.61 | 1.83 |
| 1 | 6.216986895 | 65 | 0 | 6.07 | 15 | 21 | 13 | 56 | 70.2 | 44.4 | 25.8 | 13.3 | 5 | 8.3 | 3.8 | 1.03 | 1.17 | 2.24 |
| 0 | 1.653458913 | 48 | 0 | 4.86 | 15 | 11 | 31 | 62 | 73.3 | 48.9 | 24.4 | 12.7 | 4.4 | 8.3 | 6.09 | 2.33 | 1.47 | 3.86 |
| 0 | 0.227357745 | 47 | 1 | 5.96 | 18 | 18 | 13 | 60 | 73.5 | 44.4 | 29.1 | 4.88 | 2.31 | 2.6 | 4.57 | 0.95 | 1.06 | 2.97 |
| 0 | 0.741832276 | 67 | 0 | 5.44 | 16 | 22 | 20 | 84 | 75.3 | 43.6 | 31.7 | 9.95 | 5.15 | 4.8 | 4.68 | 0.77 | 1.78 | 2.44 |
| 0 | 1.669004738 | 79 | 1 | 5.14 | 14 | 21 | 25 | 102 | 74.4 | 45.7 | 28.7 | 21 | 8.4 | 12.6 | 4.65 | 1.76 | 1.46 | 2.75 |
| 1 | 5.281525771 | 44 | 0 | 4.77 | 21 | 19 | 33 | 54 | 75.9 | 48.4 | 27.5 | 18.4 | 6.1 | 12.3 | 6.28 | 2.04 | 1.18 | 4.24 |
| 1 | 5.0597229 | 49 | 0 | 4.74 | 32 | 19 | 36 | 98 | 76.9 | 51.6 | 25.3 | 23.4 | 8.2 | 15.2 | 5.18 | 1.66 | 1.61 | 3.17 |
| 0 | 4.968880296 | 57 | 0 | 5.39 | 24 | 20 | 25 | 135 | 70.8 | 45.3 | 25.5 | 8.24 | 4.29 | 4 | 3.92 | 1.52 | 0.8 | 2.26 |
| 0 | 0.059240103 | 52 | 1 | 4.94 | 15 | 22 | 24 | 68 | 68.6 | 46.2 | 22.4 | 11.42 | 4.58 | 6.8 | 5.61 | 1.48 | 1.53 | 3.46 |
| 1 | 8.598901272 | 50 | 0 | 4.75 | 8 | 19 | 145 | 66 | 67.5 | 43.1 | 24.4 | 26.34 | 7.75 | 18.6 | 5.79 | 1.07 | 1.71 | 3.47 |
| 1 | 6.644749641 | 51 | 0 | 4.81 | 24 | 21 | 29 | 51 | 73 | 46.9 | 26.1 | 13.66 | 3.49 | 10.2 | 5.33 | 1.73 | 0.97 | 3.29 |
| 0 | 3.515452464 | 31 | 0 | 5.27 | 24 | 19 | 30 | 53 | 75.9 | 55 | 20.9 | 15.11 | 5.22 | 9.9 | 5.44 | 1.92 | 1.28 | 3.41 |
| 0 | 0.379788409 | 49 | 1 | 5.55 | 19 | 17 | 16 | 43 | 69 | 44.7 | 24.3 | 7.99 | 3.28 | 4.7 | 4.03 | 0.59 | 1.33 | 2.3 |
| 0 | 2.682808359 | 50 | 0 | 4.85 | 22 | 21 | 38 | 56 | 75.2 | 49.5 | 25.7 | 13.4 | 3.55 | 9.9 | 4.49 | 0.61 | 1.26 | 2.75 |
| 0 | 1.677968661 | 41 | 0 | 4.53 | 30 | 27 | 19 | 53 | 66.2 | 49.7 | 16.5 | 20.85 | 7.99 | 12.9 | 4.66 | 0.87 | 1.18 | 2.86 |
| 1 | 5.659305255 | 73 | 0 | 8.35 | 23 | 36 | 19 | 104 | 72.2 | 48.9 | 23.3 | 29.7 | 11.4 | 18.3 | 3.73 | 1.57 | 1.11 | 2.13 |
| 1 | 5.899393479 | 83 | 0 | 6.28 | 57 | 36 | 26 | 68 | 73 | 44.8 | 28.2 | 13.27 | 5.95 | 7.3 | 3.71 | 2.22 | 1.3 | 1.57 |
| 1 | 10.06175423 | 53 | 0 | 4.49 | 20 | 23 | 34 | 84 | 72.1 | 46.7 | 25.4 | 14.7 | 6.3 | 8.4 | 4.52 | 1.87 | 1.22 | 2.7 |
| 1 | 5.266175747 | 59 | 0 | 5.05 | 11 | 18 | 21 | 50 | 70.1 | 45 | 25.1 | 12.83 | 5.17 | 7.7 | 4.58 | 1.23 | 1.07 | 2.84 |
| 2 | 22.31052748 | 81 | 0 | 7.98 | 18 | 21 | 28 | 51 | 73.5 | 47.9 | 25.6 | 10.95 | 5.29 | 5.7 | 4.69 | 1.32 | 1.33 | 2.75 |
| 2 | 18.04167239 | 37 | 0 | 5.13 | 32 | 24 | 30 | 63 | 73.3 | 46.1 | 27.2 | 16.27 | 7.34 | 8.9 | 4.54 | 2.1 | 1 | 2.72 |
| 0 | 2.686857065 | 68 | 0 | 5.35 | 22 | 25 | 33 | 96 | 70.1 | 49.1 | 21 | 15.09 | 6.57 | 8.5 | 4.23 | 0.93 | 1.56 | 2.26 |
| 1 | 6.83155187 | 68 | 1 | 5.27 | 14 | 16 | 11 | 70 | 70.8 | 45.5 | 25.3 | 7.14 | 3.64 | 3.5 | 4.98 | 0.79 | 1.54 | 2.72 |
| 1 | 9.322991292 | 62 | 0 | 6.22 | 14 | 18 | 27 | 83 | 73.4 | 45 | 28.4 | 7.93 | 3.63 | 4.3 | 3.47 | 1.18 | 1.13 | 1.8 |
| 1 | 11.08651718 | 86 | 0 | 5.51 | 13 | 17 | 14 | 80 | 75.2 | 46.2 | 29 | 11.43 | 4.88 | 6.6 | 5 | 1.19 | 1.12 | 3.39 |
| 0 | 3.911310037 | 66 | 0 | 4.39 | 21 | 23 | 17 | 74 | 69.3 | 43.4 | 25.9 | 12.66 | 4.83 | 7.8 | 5.35 | 1.33 | 1.66 | 3.1 |
| 1 | 6.11339426 | 60 | 0 | 5.56 | 29 | 27 | 22 | 80 | 77.2 | 48.5 | 28.7 | 12.8 | 5.6 | 7.2 | 3.86 | 1.82 | 1.38 | 1.91 |
| 0 | 3.705401103 | 60 | 1 | 5.33 | 12 | 19 | 27 | 59 | 68.5 | 46.4 | 22.1 | 6.6 | 2.84 | 3.8 | 3.81 | 1.52 | 1.22 | 1.97 |
| 1 | 6.218686263 | 62 | 1 | 5.04 | 25 | 31 | 11 | 101 | 68.2 | 45 | 23.2 | 6.9 | 3.2 | 3.7 | 4.64 | 1.85 | 1.14 | 2.65 |
| 0 | 2.301876386 | 49 | 1 | 6.65 | 16 | 22 | 16 | 70 | 76.1 | 50.5 | 25.6 | 12.91 | 4.36 | 8.6 | 6.55 | 1.76 | 1.56 | 4.35 |
| 1 | 10.89183935 | 56 | 0 | 5.34 | 16 | 24 | 45 | 81 | 72.4 | 47.9 | 24.5 | 10.95 | 4.05 | 6.9 | 4.77 | 2.46 | 1.17 | 2.77 |
| 0 | 3.094614466 | 49 | 0 | 5.3 | 25 | 18 | 15 | 83 | 64.9 | 46.9 | 18 | 11.1 | 4.7 | 6.4 | 2.6 | 0.56 | 1.16 | 1.35 |
| 0 | 1.892006059 | 50 | 1 | 4.63 | 14 | 21 | 15 | 69 | 76.5 | 42.4 | 34.1 | 4.58 | 2.31 | 2.3 | 4.71 | 0.79 | 1.4 | 2.77 |
| 0 | 1.321269155 | 65 | 0 | 5.5 | 14 | 19 | 22 | 76 | 77.1 | 46.8 | 30.3 | 9.97 | 3.89 | 6.1 | 3.96 | 1.46 | 1.11 | 2.14 |
| 0 | 2.528006991 | 49 | 1 | 5.22 | 10 | 14 | 14 | 64 | 69.9 | 45.3 | 24.6 | 3.07 | 2.06 | 1 | 3.78 | 1.79 | 1.09 | 1.92 |
| 1 | 7.862721284 | 54 | 1 | 5.55 | 24 | 22 | 17 | 68 | 75 | 50 | 25 | 9.26 | 4.11 | 5.2 | 4.22 | 1.32 | 1.51 | 2.04 |
| 1 | 12.11367257 | 48 | 1 | 5.67 | 14 | 16 | 24 | 103 | 71.2 | 46.8 | 24.4 | 13.81 | 6.38 | 7.4 | 4.07 | 1.44 | 1.57 | 1.79 |
| 1 | 7.16873312 | 64 | 1 | 6.66 | 16 | 17 | 15 | 88 | 73.5 | 46.2 | 27.3 | 12.8 | 4.7 | 8.1 | 5.36 | 0.76 | 1.42 | 3.85 |
| 0 | 0.155029535 | 51 | 1 | 4.65 | 13 | 18 | 13 | 73 | 78.5 | 47.7 | 30.8 | 14.9 | 5.46 | 9.4 | 5.7 | 1.37 | 1.4 | 3.57 |
| 1 | 9.750513077 | 45 | 1 | 5.4 | 14 | 20 | 11 | 103 | 66 | 46.8 | 19.2 | 7.23 | 3.83 | 3.4 | 4.18 | 0.98 | 1.27 | 2.65 |
| 1 | 12.21442238 | 58 | 0 | 5.19 | 21 | 18 | 27 | 49 | 71.6 | 45.9 | 25.7 | 14.9 | 6.5 | 8.4 | 4.11 | 0.45 | 1.7 | 2.26 |
| 0 | 2.416895747 | 18 | 1 | 3.97 | 9 | 13 | 9 | 51 | 66.8 | 45.1 | 21.7 | 3.95 | 1.71 | 2.2 | 4.23 | 0.48 | 1.57 | 2.3 |
| 0 | 2.359345754 | 43 | 0 | 5.05 | 17 | 21 | 21 | 88 | 76.1 | 50.4 | 25.7 | 6.8 | 3.4 | 3.4 | 5.16 | 1.18 | 1.43 | 3.3 |
| 0 | 1.148979406 | 54 | 0 | 5.04 | 16 | 24 | 18 | 80 | 68.8 | 46.2 | 22.6 | 10.5 | 3.7 | 6.8 | 4.7 | 1.99 | 1.02 | 3 |
| 0 | 0.572544058 | 56 | 0 | 5.61 | 30 | 28 | 24 | 74 | 70.1 | 48.6 | 21.5 | 12.98 | 4.88 | 8.1 | 4.75 | 0.94 | 1.27 | 2.86 |
| 0 | 4.932506879 | 53 | 0 | 5.14 | 19 | 22 | 37 | 69 | 65.3 | 45.1 | 20.2 | 18.04 | 5.97 | 12.1 | 4.81 | 2.63 | 1.37 | 2.74 |
| 1 | 7.235535463 | 68 | 0 | 5.79 | 18 | 25 | 14 | 91 | 66.2 | 44.9 | 21.3 | 11.82 | 6.01 | 5.8 | 4.38 | 1.48 | 1.08 | 2.72 |
| 0 | 1.207288802 | 49 | 1 | 4.4 | 13 | 12 | 16 | 63 | 71 | 47.5 | 23.5 | 6.2 | 3.3 | 2.9 | 5.03 | 0.99 | 1.84 | 2.98 |
| 0 | 0.335966349 | 64 | 0 | 6.2 | 12 | 20 | 24 | 73 | 65.6 | 44.4 | 21.2 | 10.93 | 4.84 | 6.1 | 4.25 | 1.35 | 1.27 | 2.6 |
| 1 | 13.48987166 | 57 | 0 | 5 | 16 | 14 | 30 | 90 | 67.3 | 47.7 | 19.6 | 10.39 | 4.35 | 6 | 4.68 | 2.54 | 1.15 | 2.68 |
| 2 | 19.21871249 | 57 | 1 | 8 | 26 | 18 | 66 | 88 | 75.9 | 49.2 | 26.7 | 5.84 | 2.73 | 3.1 | 4.81 | 2.42 | 1.59 | 2.58 |
| 0 | 4.438340505 | 50 | 1 | 4.56 | 18 | 22 | 29 | 106 | 65.1 | 47.8 | 17.3 | 12.35 | 5.22 | 7.1 | 4.76 | 0.81 | 1.02 | 3.23 |
| 1 | 5.504247824 | 52 | 1 | 5.06 | 17 | 28 | 40 | 90 | 69.5 | 46.2 | 23.3 | 5.12 | 2.67 | 2.5 | 4.95 | 1.45 | 1.46 | 2.93 |
| 1 | 9.396439234 | 46 | 0 | 5.29 | 26 | 23 | 60 | 58 | 67.6 | 47.3 | 20.3 | 14.1 | 4.6 | 9.5 | 4.03 | 3.25 | 0.92 | 2.53 |
| 0 | 3.172393084 | 65 | 0 | 5.5 | 28 | 20 | 16 | 85 | 68 | 45.2 | 22.8 | 5.68 | 2.61 | 3.1 | 4.54 | 0.99 | 1.48 | 2.37 |
| 1 | 6.36419185 | 49 | 0 | 4.93 | 21 | 20 | 26 | 58 | 64.9 | 45.2 | 19.7 | 10.51 | 4.61 | 5.9 | 5.99 | 2.14 | 1.56 | 3.64 |
| 1 | 5.78484265 | 45 | 0 | 4.78 | 11 | 23 | 20 | 71 | 67.8 | 46.8 | 21 | 16.3 | 5.8 | 10.5 | 3.63 | 0.38 | 1.57 | 2.03 |
| 1 | 13.58608182 | 43 | 0 | 5.25 | 21 | 17 | 33 | 94 | 71 | 48.3 | 22.7 | 16.86 | 6.07 | 10.8 | 6.05 | 2.38 | 1.06 | 3.92 |
| 1 | 7.423626741 | 51 | 1 | 5.21 | 21 | 21 | 22 | 62 | 72.2 | 48 | 24.2 | 9.41 | 3.59 | 5.8 | 4.89 | 0.73 | 2.26 | 2.44 |
| 1 | 12.86817487 | 57 | 0 | 5.77 | 13 | 20 | 25 | 56 | 68.5 | 49.4 | 19.1 | 11.08 | 4.83 | 6.3 | 3.55 | 1.68 | 1.13 | 2.05 |
| 1 | 10.03575325 | 48 | 0 | 4.88 | 22 | 21 | 23 | 53 | 67 | 47.5 | 19.5 | 12.07 | 5.48 | 6.6 | 4.24 | 1.16 | 1.08 | 2.58 |
| 1 | 10.49778525 | 58 | 0 | 5 | 17 | 26 | 25 | 60 | 70.1 | 42.4 | 27.7 | 9.47 | 4.67 | 4.8 | 4.74 | 1.22 | 1.37 | 2.75 |
| 0 | 4.601626515 | 49 | 0 | 4.93 | 25 | 20 | 18 | 78 | 75.3 | 46.4 | 28.9 | 12.16 | 5.65 | 6.5 | 4.99 | 1.26 | 1.35 | 3.13 |
| 0 | 2.374013662 | 46 | 1 | 5.3 | 11 | 15 | 10 | 55 | 66.7 | 43.8 | 22.9 | 11.6 | 4.6 | 7 | 3.47 | 0.92 | 1.29 | 2.05 |
| 1 | 6.162198385 | 56 | 0 | 4.79 | 18 | 21 | 20 | 106 | 76.7 | 49.1 | 27.6 | 9.3 | 4.11 | 5.2 | 4.24 | 1.55 | 1.37 | 2.16 |
| 1 | 6.406167348 | 59 | 1 | 5.2 | 16 | 20 | 13 | 95 | 76.8 | 46.8 | 30 | 3.73 | 1.61 | 2.1 | 5.02 | 1.88 | 1.3 | 2.66 |
| 0 | 3.597071966 | 37 | 0 | 4.63 | 25 | 23 | 73 | 85 | 79.5 | 50.4 | 29.1 | 24.99 | 7.72 | 17.3 | 5.28 | 0.87 | 1.21 | 3.37 |
| 0 | 4.952127616 | 57 | 0 | 5.9 | 26 | 27 | 47 | 111 | 69.7 | 48.5 | 21.2 | 15.45 | 3.83 | 11.6 | 5 | 3.51 | 0.9 | 2.94 |
| 2 | 20.34919103 | 37 | 1 | 4.33 | 59 | 30 | 23 | 69 | 74 | 47.6 | 26.4 | 10.07 | 4.8 | 5.3 | 4.81 | 1.69 | 1.63 | 2.67 |
| 1 | 12.02917035 | 42 | 0 | 5.09 | 31 | 26 | 96 | 94 | 68.9 | 49.5 | 19.4 | 19.01 | 6.88 | 12.1 | 5.23 | 2.43 | 1.35 | 3.37 |
| 0 | 0.391767104 | 42 | 0 | 4.91 | 15 | 19 | 24 | 74 | 70 | 46.4 | 23.6 | 14 | 5.1 | 8.9 | 5.15 | 1.58 | 1.08 | 3.55 |
| 1 | 7.495031039 | 41 | 0 | 5.07 | 15 | 14 | 15 | 99 | 73.6 | 48.3 | 25.3 | 12.3 | 4.6 | 7.7 | 5.25 | 2.5 | 1.09 | 3.23 |
| 1 | 10.6777366 | 52 | 0 | 6.73 | 39 | 22 | 43 | 90 | 72.5 | 47.2 | 25.3 | 15.6 | 5.8 | 9.8 | 3.55 | 1.77 | 0.97 | 1.88 |
| 0 | 4.53240331 | 58 | 1 | 4.75 | 15 | 21 | 13 | 81 | 70.7 | 47.1 | 23.6 | 13.14 | 5.33 | 7.8 | 5.02 | 1 | 1.68 | 3.05 |
| 1 | 6.849548658 | 57 | 0 | 4.74 | 16 | 13 | 12 | 68 | 68 | 46 | 22 | 11.4 | 5.5 | 5.9 | 3.17 | 1.58 | 1.04 | 1.5 |
| 0 | 3.914865017 | 40 | 1 | 4.63 | 10 | 13 | 13 | 56 | 70.7 | 47.2 | 23.5 | 6.04 | 3.19 | 2.8 | 4.54 | 0.53 | 1.84 | 2.22 |
| 1 | 7.903291225 | 58 | 1 | 5.1 | 24 | 21 | 28 | 71 | 69.3 | 46.5 | 22.8 | 11.71 | 4.31 | 7.4 | 5.04 | 2.2 | 1.17 | 3.18 |
| 1 | 10.1486578 | 54 | 0 | 4.84 | 23 | 34 | 6 | 44 | 60.1 | 42 | 18.1 | 13.77 | 5.14 | 8.6 | 4.66 | 1.27 | 1.35 | 2.62 |
| 2 | 24.68734042 | 59 | 0 | 4.78 | 19 | 18 | 21 | 61 | 72.4 | 50.1 | 22.3 | 8.34 | 4.4 | 3.9 | 3.98 | 1.28 | 1.68 | 1.88 |
| 1 | 5.148152669 | 65 | 1 | 5.37 | 13 | 22 | 18 | 113 | 75.5 | 49 | 26.5 | 11.01 | 5.58 | 5.4 | 4.88 | 1.69 | 1.37 | 2.75 |
| 1 | 6.744579474 | 54 | 1 | 4.29 | 108 | 51 | 142 | 149 | 74.8 | 44.2 | 30.6 | 5.74 | 3.95 | 1.8 | 3.07 | 1 | 1.76 | 0.84 |
| 1 | 5.207342307 | 54 | 0 | 5.78 | 22 | 27 | 333 | 83 | 67.3 | 46.7 | 20.6 | 10.43 | 5.16 | 5.3 | 3.5 | 2.53 | 1.56 | 1.2 |
| 0 | 4.714439948 | 56 | 1 | 4.59 | 13 | 18 | 5 | 91 | 67.9 | 47.1 | 20.8 | 11.49 | 4.79 | 6.7 | 4.7 | 0.83 | 1.45 | 2.9 |
| 0 | 2.517602364 | 57 | 1 | 5.02 | 30 | 23 | 76 | 109 | 74.1 | 46.3 | 27.8 | 7.8 | 3.4 | 4.4 | 6.02 | 0.99 | 1.58 | 4.27 |
| 0 | 4.787453334 | 51 | 0 | 5.01 | 13 | 16 | 33 | 57 | 72.9 | 49.4 | 23.5 | 18.86 | 8.56 | 10.3 | 5.71 | 1.41 | 1.6 | 3.44 |
| 0 | 2.584696333 | 52 | 1 | 4.55 | 25 | 18 | 75 | 98 | 73.6 | 46.6 | 27 | 6.9 | 3.6 | 3.3 | 4.57 | 0.89 | 1.45 | 2.63 |
| 1 | 6.839022477 | 58 | 1 | 5.36 | 18 | 23 | 13 | 89 | 66.6 | 43.6 | 23 | 11.3 | 4.1 | 7.2 | 4.76 | 1.25 | 1.13 | 3.05 |
| 0 | 0.459649523 | 56 | 1 | 5.76 | 17 | 24 | 22 | 34 | 78.4 | 51.3 | 27.1 | 11.31 | 4.15 | 7.2 | 4.62 | 1.64 | 1.41 | 2.49 |
| 1 | 5.887323538 | 57 | 0 | 6.98 | 17 | 17 | 13 | 81 | 77.2 | 50.4 | 26.8 | 16.8 | 6.5 | 10.3 | 5.26 | 1.59 | 1.72 | 3 |
| 1 | 6.210587978 | 46 | 0 | 5.2 | 16 | 22 | 18 | 55 | 77.6 | 46.5 | 31.1 | 17.3 | 6.1 | 11.2 | 4.72 | 1.62 | 1.11 | 2.92 |
| 1 | 7.14339002 | 46 | 0 | 5.04 | 46 | 26 | 31 | 52 | 67.3 | 47.8 | 19.5 | 11.22 | 4.35 | 6.9 | 4.07 | 1.5 | 1.13 | 2.53 |
| 0 | 1.982840459 | 49 | 0 | 5.96 | 27 | 23 | 128 | 59 | 69 | 47.7 | 21.3 | 16.78 | 4.89 | 11.9 | 4.73 | 2.14 | 1.21 | 2.77 |
| 0 | 4.827436527 | 43 | 0 | 5.32 | 16 | 18 | 26 | 70 | 67.6 | 44.3 | 23.3 | 9.56 | 3.08 | 6.5 | 4.03 | 1.36 | 0.99 | 2.53 |
| 1 | 10.49408531 | 44 | 0 | 5.2 | 21 | 23 | 22 | 97 | 80.1 | 51 | 29.1 | 31 | 7.9 | 23.1 | 5.79 | 1.66 | 1.75 | 3.79 |
| 0 | 3.976362228 | 63 | 0 | 15.44 | 21 | 17 | 19 | 97 | 72.5 | 43.2 | 29.3 | 16.96 | 6.47 | 10.5 | 3.87 | 1.7 | 0.91 | 2.37 |
| 1 | 5.699429035 | 60 | 0 | 5.85 | 8 | 13 | 13 | 60 | 65.5 | 46.2 | 19.3 | 11.95 | 5.71 | 6.2 | 3.92 | 0.77 | 1.3 | 2.37 |
| 0 | 4.183408578 | 55 | 0 | 4.72 | 17 | 20 | 23 | 69 | 67.4 | 49.1 | 18.3 | 7.4 | 3.51 | 3.9 | 4.87 | 1.97 | 1.22 | 3.05 |
| 0 | 3.3312428 | 42 | 0 | 5.73 | 21 | 19 | 13 | 77 | 73.2 | 50.3 | 22.9 | 15.88 | 5.85 | 10 | 4.8 | 1.23 | 1.44 | 2.63 |
| 0 | 3.394112905 | 46 | 0 | 5.07 | 9 | 20 | 23 | 50 | 68.9 | 41.5 | 27.4 | 10.17 | 9.28 | 0.9 | 5.21 | 19.23 | 0.54 | 1.15 |
| 1 | 5.941295624 | 72 | 1 | 4.26 | 11 | 17 | 12 | 91 | 73.7 | 46 | 27.7 | 7.4 | 3.5 | 3.9 | 5.03 | 1.3 | 1.26 | 3.22 |
| 0 | 4.717128595 | 71 | 1 | 5.45 | 24 | 39 | 12 | 88 | 75 | 48 | 27 | 15.6 | 6 | 9.6 | 3.8 | 1.02 | 1.89 | 1.7 |
| 1 | 6.766001383 | 67 | 1 | 5.47 | 27 | 19 | 43 | 108 | 69.9 | 45.7 | 24.2 | 7.81 | 2.69 | 5.1 | 4.35 | 1.23 | 1.62 | 2.16 |
| 1 | 10.61747535 | 68 | 0 | 4.8 | 13 | 19 | 12 | 83 | 68.2 | 42.9 | 25.3 | 14.53 | 5.14 | 9.4 | 3.44 | 0.83 | 1.14 | 1.98 |
| 1 | 11.49756241 | 57 | 0 | 7.54 | 16 | 17 | 41 | 82 | 71.7 | 48.2 | 23.5 | 20.97 | 7.92 | 13.1 | 5.89 | 2.48 | 1.55 | 3.08 |
| 1 | 8.860997041 | 50 | 1 | 5.24 | 15 | 16 | 12 | 58 | 69.3 | 42.5 | 26.8 | 12.78 | 6.28 | 6.5 | 3.5 | 0.78 | 1.35 | 1.7 |
| 0 | 2.692865849 | 53 | 1 | 8.12 | 35 | 22 | 14 | 38 | 77.1 | 46 | 31.1 | 9.6 | 4.7 | 4.9 | 2.91 | 1.32 | 1.25 | 1.32 |
| 1 | 5.434562047 | 69 | 1 | 4.92 | 21 | 21 | 31 | 108 | 75.9 | 44.9 | 31 | 5.65 | 2.99 | 2.7 | 4.6 | 1.34 | 1.69 | 2.2 |
| 2 | 20.83978971 | 58 | 0 | 5.83 | 48 | 34 | 56 | 79 | 72.6 | 43.8 | 28.8 | 8.69 | 3.71 | 5 | 6.82 | 4.59 | 1 | 4.45 |
| 0 | 4.09642148 | 45 | 1 | 4.77 | 19 | 22 | 8 | 66 | 71.7 | 47.1 | 24.6 | 16.67 | 6.5 | 10.2 | 4.18 | 1.58 | 1.45 | 2.13 |
| 1 | 7.378588041 | 52 | 0 | 4.61 | 26 | 20 | 21 | 92 | 67.5 | 45.5 | 22 | 9 | 4.5 | 4.5 | 4.05 | 1.94 | 1.21 | 2.31 |
| 1 | 7.224553585 | 50 | 0 | 5.96 | 24 | 21 | 23 | 83 | 66.9 | 50.6 | 16.3 | 11.84 | 5.62 | 6.2 | 3.91 | 2.01 | 0.9 | 2.22 |
| 0 | 3.391345064 | 57 | 1 | 5.76 | 21 | 23 | 12 | 79 | 79.2 | 50.8 | 28.4 | 8.13 | 4.35 | 3.8 | 4.34 | 1.13 | 1.5 | 2.16 |
| 1 | 5.389237324 | 47 | 1 | 4.58 | 17 | 15 | 17 | 49 | 67.7 | 49.4 | 18.3 | 8.29 | 4.04 | 4.3 | 5.15 | 0.98 | 1.28 | 3.42 |
| 0 | 2.742022276 | 51 | 1 | 4.83 | 23 | 20 | 14 | 90 | 72.6 | 44.3 | 28.3 | 5.18 | 2.22 | 3 | 4.46 | 2.28 | 1.35 | 2.05 |
| 1 | 5.625828743 | 50 | 0 | 4.95 | 30 | 30 | 32 | 43 | 73 | 48.4 | 24.6 | 12.12 | 5.02 | 7.1 | 5.26 | 2.79 | 1.25 | 2.52 |
| 0 | 0.210721811 | 54 | 0 | 5.07 | 15 | 20 | 44 | 77 | 71.7 | 46.5 | 25.2 | 18.7 | 6.3 | 12.4 | 4.44 | 1.95 | 1.25 | 2.3 |
| 0 | 3.968425711 | 59 | 0 | 4.86 | 12 | 17 | 23 | 79 | 63.2 | 46.7 | 16.5 | 14.81 | 5.35 | 9.5 | 4.73 | 1.35 | 1.73 | 2.71 |
| 0 | 4.135274887 | 49 | 1 | 5.28 | 11 | 19 | 10 | 87 | 76.2 | 45.4 | 30.8 | 10.5 | 4.5 | 6 | 4.04 | 0.8 | 1.48 | 2.19 |
| 0 | 3.289269686 | 43 | 1 | 5.21 | 13 | 18 | 9 | 55 | 68.7 | 49.3 | 19.4 | 6.93 | 3.41 | 3.5 | 4.71 | 0.55 | 1.97 | 2.5 |
| 1 | 8.06683445 | 43 | 0 | 4.28 | 15 | 14 | 25 | 73 | 72.9 | 46.3 | 26.6 | 7.41 | 3.99 | 3.4 | 4.95 | 4.3 | 1.34 | 2.26 |
| 1 | 6.129131317 | 49 | 1 | 5.17 | 23 | 25 | 11 | 90 | 79.5 | 48.3 | 31.2 | 6.98 | 2.88 | 4.1 | 7.24 | 2.68 | 1.92 | 3.98 |
| 0 | 0.384463906 | 50 | 1 | 5.18 | 11 | 16 | 19 | 49 | 73.4 | 50 | 23.4 | 10.42 | 4.87 | 5.6 | 4.45 | 1.78 | 1.23 | 2.55 |
| 1 | 5.102526665 | 51 | 0 | 4.84 | 12 | 14 | 9 | 61 | 70.4 | 49.7 | 20.7 | 15.77 | 6.34 | 9.4 | 3.85 | 0.98 | 1.15 | 2.2 |
| 1 | 12.66221333 | 53 | 0 | 4.55 | 38 | 28 | 53 | 56 | 74.6 | 51.3 | 23.3 | 15.7 | 5.51 | 10.2 | 4.5 | 2.15 | 1.05 | 2.69 |
| 1 | 8.567094008 | 57 | 0 | 4.54 | 21 | 24 | 21 | 55 | 67.9 | 45.1 | 22.8 | 26.61 | 9.28 | 17.3 | 4.3 | 0.94 | 1.16 | 2.74 |
| 0 | 4.010157824 | 60 | 0 | 5.14 | 12 | 17 | 12 | 52 | 71.9 | 45.2 | 26.7 | 8.9 | 2.8 | 6.1 | 4.04 | 0.78 | 1.12 | 2.69 |
| 0 | 4.161939025 | 61 | 1 | 4.74 | 11 | 17 | 9 | 108 | 74.9 | 43.9 | 31 | 8.8 | 3.7 | 5.1 | 3.94 | 1.55 | 1.11 | 2.19 |
| 0 | 3.142346263 | 57 | 1 | 5.31 | 17 | 16 | 12 | 84 | 75.6 | 46.3 | 29.3 | 6.78 | 2.84 | 3.9 | 5.46 | 1.92 | 1.27 | 3.64 |
| 1 | 6.684445937 | 57 | 0 | 4.91 | 9 | 14 | 20 | 64 | 66.1 | 45.5 | 20.6 | 14.49 | 5.3 | 9.2 | 5.18 | 1.25 | 1.71 | 2.93 |
| 0 | 3.569185734 | 51 | 1 | 5.39 | 11 | 24 | 11 | 51 | 64.7 | 41.4 | 23.3 | 6.8 | 3.62 | 3.2 | 5.82 | 1.31 | 1.55 | 3.5 |
| 1 | 6.688079675 | 64 | 1 | 6.26 | 20 | 22 | 10 | 106 | 62.3 | 38.9 | 23.4 | 6.13 | 3.5 | 2.6 | 2.74 | 0.79 | 1.11 | 1.27 |
| 2 | 26.71591949 | 53 | 0 | 6.96 | 68 | 43 | 63 | 67 | 69.8 | 45.2 | 24.6 | 11.24 | 4.73 | 6.5 | 4.16 | 2.1 | 1.02 | 2.6 |
| 1 | 6.290417671 | 55 | 1 | 5.49 | 17 | 19 | 17 | 89 | 74.4 | 51.1 | 23.3 | 10.31 | 4.24 | 6.1 | 3.64 | 0.79 | 1.35 | 1.71 |
| 0 | 4.740383546 | 48 | 1 | 5.07 | 20 | 17 | 15 | 84 | 76.2 | 43.5 | 32.7 | 5.39 | 2.34 | 3 | 5.45 | 2.13 | 1.25 | 3.36 |
| 0 | 3.241105795 | 57 | 0 | 5.02 | 18 | 22 | 16 | 64 | 72.1 | 46.1 | 26 | 8.58 | 3.17 | 5.4 | 4.17 | 0.73 | 1.3 | 2.38 |
| 1 | 6.193889141 | 43 | 0 | 4.16 | 23 | 16 | 31 | 104 | 70.5 | 45.8 | 24.7 | 14.41 | 5.15 | 9.3 | 4.79 | 1.41 | 1.10 | 3.28 |
| 1 | 6.849728743 | 58 | 0 | 5.84 | 10 | 12 | 15 | 70 | 72 | 41.5 | 30.5 | 4.6 | 3 | 1.6 | 3.7 | 0.81 | 0.78 | 2.43 |
| 0 | 2.197762529 | 54 | 1 | 4.76 | 17 | 20 | 13 | 68 | 76.4 | 46.6 | 29.8 | 16.79 | 6.06 | 10.7 | 5.15 | 1.12 | 1.29 | 3.29 |
| 1 | 5.226157665 | 54 | 0 | 5.78 | 10 | 23 | 40 | 92 | 69.4 | 50.8 | 18.6 | 7.88 | 2.9 | 5 | 5.81 | 2.39 | 1.27 | 3.53 |
| 1 | 5.209303379 | 53 | 0 | 4.97 | 29 | 30 | 20 | 79 | 75.8 | 46.4 | 29.4 | 20.9 | 6.3 | 14.6 | 4.63 | 1.41 | 1.26 | 2.83 |
| 1 | 12.56420771 | 59 | 1 | 6.19 | 23 | 21 | 23 | 65 | 74.5 | 48.8 | 25.7 | 13.93 | 5.63 | 8.3 | 4.97 | 3.83 | 1.16 | 2.03 |
| 1 | 7.82010301 | 46 | 0 | 5.91 | 41 | 33 | 58 | 50 | 73.4 | 46.1 | 27.3 | 22.5 | 6.5 | 16 | 6.32 | 2.94 | 1.27 | 3.97 |
| 1 | 6.650156816 | 47 | 0 | 5.48 | 31 | 32 | 42 | 65 | 76.1 | 49.4 | 26.7 | 22.97 | 5.85 | 17.1 | 6.42 | 1.8 | 1.29 | 4.09 |
| 0 | 3.438663801 | 50 | 1 | 4.38 | 11 | 19 | 11 | 50 | 67.3 | 45.8 | 21.5 | 14.19 | 5.11 | 9.1 | 4.88 | 0.83 | 1.85 | 2.56 |
| 1 | 12.28628508 | 75 | 1 | 5.64 | 38 | 25 | 35 | 63 | 77.5 | 45.2 | 32.3 | 9.95 | 3.95 | 6 | 5.35 | 1.32 | 1.09 | 3.68 |
| 0 | 4.183917046 | 63 | 0 | 5.98 | 15 | 15 | 40 | 71 | 67 | 45.8 | 21.2 | 7.76 | 3.48 | 4.3 | 4.5 | 1.26 | 1.3 | 2.5 |
| 1 | 11.58941062 | 61 | 0 | 4.96 | 31 | 29 | 34 | 76 | 70.7 | 50.5 | 20.2 | 16.21 | 6.93 | 9.3 | 5.06 | 1.96 | 0.83 | 3.25 |
| 0 | 2.824347814 | 47 | 1 | 5.31 | 10 | 16 | 21 | 64 | 65 | 46.2 | 18.8 | 7.92 | 4.12 | 3.8 | 4.75 | 1.34 | 1.14 | 3.18 |
| 0 | 2.111408552 | 49 | 1 | 6.17 | 15 | 22 | 11 | 96 | 76.9 | 49.7 | 27.2 | 11.85 | 4.66 | 7.2 | 6.12 | 1.12 | 2.01 | 3.38 |
| 1 | 10.29993153 | 55 | 0 | 5.77 | 27 | 19 | 57 | 48 | 63.6 | 47.1 | 16.5 | 10.02 | 3.54 | 6.5 | 5.67 | 1.43 | 1.19 | 3.91 |
| 1 | 10.43374983 | 43 | 0 | 5.04 | 20 | 22 | 22 | 95 | 70.9 | 47.3 | 23.6 | 12.38 | 5.19 | 7.2 | 2.76 | 0.96 | 0.97 | 1.25 |
| 1 | 10.75618013 | 52 | 1 | 5.03 | 37 | 25 | 73 | 92 | 69.4 | 45.8 | 23.6 | 7.58 | 4.16 | 3.4 | 4.54 | 1.12 | 1.18 | 2.72 |
| 0 | 2.272898833 | 42 | 1 | 5.5 | 12 | 15 | 12 | 74 | 70.5 | 49.7 | 20.8 | 10.23 | 3.74 | 6.5 | 4.34 | 1.11 | 1.17 | 2.7 |
| 1 | 10.87075075 | 54 | 0 | 4.7 | 24 | 25 | 31 | 78 | 74.4 | 47.8 | 26.6 | 11.9 | 5.1 | 6.8 | 5.73 | 1.31 | 1.28 | 3.86 |
| 0 | 3.834909757 | 48 | 1 | 5.76 | 14 | 22 | 15 | 72 | 72.2 | 43.9 | 28.3 | 4.06 | 1.67 | 2.4 | 5.47 | 1.75 | 1.59 | 3.16 |
| 1 | 6.679949601 | 45 | 0 | 5 | 14 | 19 | 19 | 59 | 75.2 | 48.5 | 26.7 | 10.99 | 5.03 | 6 | 4.67 | 0.67 | 1.17 | 2.98 |
| 1 | 10.27979072 | 41 | 0 | 4.68 | 22 | 20 | 26 | 76 | 76 | 49.5 | 26.5 | 8.7 | 3.7 | 5 | 5.01 | 1.72 | 1.24 | 3.32 |
| 1 | 6.819578807 | 60 | 1 | 5.04 | 15 | 22 | 15 | 108 | 74.9 | 45.5 | 29.4 | 10.1 | 3.7 | 6.4 | 4.22 | 1.7 | 1.31 | 2.36 |
| 0 | 4.674002488 | 58 | 1 | 4.83 | 19 | 20 | 10 | 100 | 66.3 | 49.9 | 16.4 | 7.09 | 3.06 | 4 | 5.43 | 1.23 | 1.29 | 3.27 |
| 1 | 13.02150536 | 58 | 1 | 5.44 | 33 | 23 | 21 | 101 | 72.2 | 44.8 | 27.4 | 15.8 | 7.8 | 8 | 3.52 | 1.14 | 1.17 | 1.88 |
| 1 | 7.085288525 | 54 | 0 | 4.81 | 9 | 19 | 28 | 84 | 80.9 | 49.4 | 31.5 | 12.71 | 4.81 | 7.9 | 5.06 | 1.1 | 1.33 | 2.94 |
| 0 | 1.127272129 | 43 | 0 | 5.37 | 24 | 17 | 37 | 84 | 74.8 | 45.9 | 28.9 | 12.31 | 4 | 8.3 | 4.63 | 2.96 | 0.89 | 2.86 |
| 1 | 6.824831645 | 42 | 1 | 5.07 | 12 | 17 | 13 | 76 | 75.5 | 48.6 | 26.9 | 12.3 | 4.7 | 7.6 | 4.31 | 2.8 | 1.1 | 2.28 |
| 0 | 2.972357909 | 51 | 0 | 5.25 | 17 | 20 | 9 | 86 | 70.2 | 49.8 | 20.4 | 17.35 | 6.11 | 11.2 | 5.46 | 1.18 | 1.78 | 3.09 |
| 1 | 12.93972921 | 59 | 0 | 5.32 | 31 | 26 | 25 | 96 | 68 | 46.6 | 21.4 | 11.88 | 5.35 | 6.5 | 3.18 | 1.89 | 0.93 | 1.5 |
| 0 | 3.394400279 | 50 | 1 | 5.3 | 22 | 21 | 43 | 68 | 69.6 | 45.8 | 23.8 | 11.94 | 4.5 | 7.4 | 5.22 | 1.35 | 1.74 | 2.85 |
| 1 | 13.79787922 | 48 | 0 | 4.88 | 35 | 24 | 18 | 63 | 65.5 | 46.9 | 18.6 | 12.32 | 4.98 | 7.3 | 3.33 | 0.77 | 0.82 | 2.08 |
| 1 | 10.81932068 | 54 | 0 | 5.22 | 29 | 22 | 23 | 50 | 68.3 | 48.7 | 19.6 | 7.82 | 3.79 | 4 | 4.99 | 0.97 | 0.97 | 3.45 |
| 2 | 20.38812383 | 66 | 0 | 5.06 | 22 | 18 | 18 | 77 | 67.5 | 43 | 24.5 | 7.23 | 4.21 | 3 | 3.86 | 1.66 | 0.84 | 2.39 |
| 0 | 3.396620989 | 67 | 0 | 6.2 | 42 | 37 | 27 | 30 | 73.6 | 47.1 | 26.5 | 14.95 | 7.13 | 7.8 | 2.53 | 0.86 | 0.9 | 1.16 |
| 1 | 7.039475759 | 51 | 0 | 8.86 | 27 | 23 | 37 | 76 | 69.7 | 44.1 | 25.6 | 11.1 | 4.5 | 6.6 | 4.04 | 0.96 | 1.21 | 2.44 |
| 0 | 3.139982303 | 61 | 0 | 5.22 | 11 | 12 | 17 | 70 | 69.6 | 46.7 | 22.9 | 12.72 | 6.43 | 6.3 | 3.12 | 0.67 | 1.07 | 1.69 |
| 1 | 6.009962559 | 58 | 1 | 5.64 | 14 | 22 | 54 | 121 | 68.6 | 50.1 | 18.5 | 13.26 | 5.53 | 7.7 | 3.68 | 2.61 | 1.13 | 1.61 |
| 0 | 4.764298598 | 45 | 1 | 4.89 | 9 | 13 | 8 | 46 | 71.1 | 47.3 | 23.8 | 12.37 | 5.08 | 7.3 | 5.29 | 0.65 | 2.46 | 2.3 |
| 2 | 18.02643967 | 49 | 1 | 5.57 | 24 | 19 | 27 | 77 | 65.9 | 43.7 | 22.2 | 6.09 | 3.43 | 2.7 | 4.62 | 1.73 | 0.92 | 3.05 |
| 1 | 5.536179701 | 58 | 0 | 5.41 | 36 | 26 | 23 | 67 | 70.7 | 47.7 | 23 | 18.98 | 6.49 | 12.5 | 4.2 | 0.8 | 1.33 | 2.5 |
| 0 | 3.940660755 | 58 | 0 | 5.78 | 10 | 16 | 20 | 58 | 64 | 45.1 | 18.9 | 11.09 | 5.03 | 6.1 | 4.35 | 1.14 | 1.23 | 2.65 |
| 0 | 2.343726198 | 60 | 0 | 5.29 | 16 | 19 | 20 | 46 | 71.9 | 51.3 | 20.6 | 12.1 | 5 | 7.1 | 4.43 | 1 | 1.87 | 2.24 |
| 1 | 13.65261793 | 63 | 0 | 7.3 | 24 | 22 | 21 | 52 | 76.5 | 51.2 | 25.3 | 10.58 | 5.14 | 5.4 | 4.1 | 1.65 | 0.95 | 2.41 |
| 1 | 6.76482598 | 67 | 0 | 8.43 | 15 | 21 | 14 | 59 | 73.9 | 44.7 | 29.2 | 6.4 | 2.9 | 3.5 | 5.19 | 0.75 | 1.2 | 3.84 |
| 0 | 1.508384585 | 52 | 0 | 5.24 | 17 | 17 | 38 | 97 | 76.1 | 50.7 | 25.4 | 15.1 | 5.5 | 9.6 | 4.91 | 2.34 | 1.01 | 3.19 |
| 1 | 8.207102776 | 58 | 0 | 6.05 | 10 | 10 | 35 | 105 | 73.6 | 42.6 | 31 | 6.09 | 3.49 | 2.6 | 4.19 | 1.84 | 1.33 | 2.41 |
| 1 | 5.59644413 | 57 | 0 | 6.2 | 36 | 26 | 13 | 82 | 66.3 | 45.3 | 21 | 14.37 | 7.3 | 7.1 | 2.79 | 1.31 | 1.25 | 1.12 |
| 1 | 8.406290372 | 55 | 0 | 7.71 | 25 | 24 | 32 | 70 | 75.7 | 49.2 | 26.5 | 15.3 | 6.6 | 8.7 | 2.92 | 1.33 | 1.38 | 1.22 |
| 1 | 7.595511278 | 67 | 0 | 6.62 | 19 | 21 | 21 | 72 | 74.8 | 47.1 | 27.7 | 9.79 | 4.86 | 4.9 | 3.13 | 1.93 | 0.93 | 1.34 |
| 1 | 5.871830146 | 65 | 0 | 7.83 | 16 | 24 | 49 | 124 | 77.2 | 46.8 | 30.4 | 16.9 | 5.6 | 11.3 | 4.25 | 0.73 | 1.29 | 2.69 |
| 1 | 7.987728437 | 52 | 0 | 5.17 | 10 | 15 | 14 | 51 | 66.9 | 50.2 | 16.7 | 16.6 | 7.9 | 8.7 | 5.84 | 0.57 | 3.34 | 2.5 |
| 1 | 9.852950255 | 44 | 0 | 6.14 | 19 | 15 | 25 | 93 | 72.5 | 51 | 21.5 | 20.28 | 6.82 | 13.5 | 3.7 | 1.13 | 1.18 | 2.02 |
| 0 | 4.123457233 | 55 | 0 | 4.81 | 23 | 20 | 20 | 75 | 67.5 | 46.6 | 20.9 | 6.34 | 2.73 | 3.6 | 4.88 | 0.86 | 0.97 | 3.35 |
| 0 | 2.007832368 | 52 | 0 | 5.19 | 15 | 17 | 17 | 79 | 74 | 50.5 | 23.5 | 11.02 | 4.74 | 6.3 | 4.7 | 1.65 | 0.94 | 3.03 |
| 1 | 9.027086894 | 52 | 0 | 5.27 | 14 | 20 | 14 | 61 | 72.1 | 49.6 | 22.5 | 8.88 | 4.11 | 4.8 | 4.92 | 0.81 | 1.5 | 3.07 |
| 1 | 11.95548471 | 53 | 0 | 5.41 | 19 | 17 | 35 | 50 | 71.3 | 44.5 | 26.8 | 15.93 | 5.94 | 10 | 4.25 | 2 | 1.28 | 2.27 |
| 1 | 6.641546885 | 60 | 0 | 4.97 | 25 | 19 | 25 | 83 | 73 | 45.6 | 27.4 | 12.09 | 4.71 | 7.4 | 3.75 | 1.25 | 1.17 | 2.06 |
| 2 | 18.55737623 | 74 | 0 | 5.84 | 14 | 18 | 21 | 76 | 76.7 | 49.9 | 26.8 | 6.4 | 2.7 | 3.7 | 4.69 | 4.46 | 1.21 | 2.08 |
| 0 | 3.37129883 | 46 | 1 | 4.71 | 13 | 19 | 14 | 56 | 71.9 | 50.2 | 21.7 | 13.44 | 5.43 | 8 | 3.86 | 1.14 | 1.29 | 2.06 |
| 1 | 8.14836216 | 54 | 1 | 4.66 | 30 | 22 | 16 | 114 | 76.4 | 42.1 | 34.3 | 6.03 | 3.1 | 2.9 | 5.04 | 1.27 | 1.22 | 3.39 |
| 0 | 4.385585467 | 56 | 0 | 5.44 | 24 | 20 | 16 | 63 | 72.2 | 46 | 26.2 | 14.4 | 4.9 | 9.5 | 4.5 | 1.91 | 0.93 | 2.75 |
| 0 | 3.598231951 | 60 | 0 | 5.6 | 33 | 34 | 42 | 68 | 69.9 | 47.2 | 22.7 | 9.28 | 4.11 | 5.2 | 3.08 | 0.87 | 1.29 | 1.37 |
| 1 | 5.090425332 | 65 | 0 | 5.76 | 13 | 22 | 15 | 49 | 66.8 | 47.4 | 19.4 | 23.82 | 8.96 | 14.9 | 3.67 | 1.66 | 0.92 | 2.07 |
| 1 | 9.00857989 | 65 | 0 | 4.69 | 14 | 16 | 30 | 76 | 78.4 | 49.6 | 28.8 | 12.39 | 3.98 | 8.4 | 6.29 | 2.24 | 1.13 | 3.83 |
| 0 | 2.83412536 | 57 | 0 | 5.35 | 16 | 19 | 15 | 86 | 76.5 | 53.9 | 22.6 | 10.7 | 4.18 | 6.5 | 4.77 | 1.64 | 1.15 | 2.83 |
| 1 | 9.721002897 | 48 | 1 | 4.7 | 14 | 18 | 4 | 42 | 66.4 | 44.7 | 21.7 | 7.34 | 3.09 | 4.3 | 4.76 | 1.07 | 1.97 | 2.37 |
| 1 | 7.107334932 | 43 | 0 | 4.7 | 26 | 30 | 25 | 49 | 67 | 47.1 | 19.9 | 12.88 | 4.65 | 8.2 | 5.02 | 0.94 | 1.45 | 3.08 |
| 0 | 2.148205241 | 58 | 0 | 5.46 | 14 | 16 | 17 | 61 | 67 | 49.2 | 17.8 | 12.65 | 5.07 | 7.6 | 5.61 | 1.36 | 1.1 | 3.73 |
| 0 | 1.003801982 | 59 | 0 | 4.5 | 29 | 23 | 13 | 75 | 65.7 | 41.5 | 24.2 | 10.2 | 4.68 | 5.5 | 5.11 | 1.08 | 1.45 | 3.26 |
| 1 | 8.679833571 | 58 | 0 | 6.06 | 22 | 19 | 25 | 111 | 67.5 | 44.4 | 23.1 | 12.5 | 4.7 | 7.8 | 3.53 | 1.45 | 1.02 | 2.09 |
| 1 | 7.215710958 | 65 | 0 | 7 | 9 | 15 | 13 | 44 | 58.2 | 41.1 | 17.1 | 9.6 | 3.94 | 5.7 | 4 | 0.91 | 1.25 | 2.3 |
| 1 | 7.013325373 | 58 | 1 | 4.89 | 25 | 27 | 27 | 106 | 83.4 | 50.9 | 32.5 | 21.5 | 8.3 | 13.2 | 5.6 | 1.12 | 1.81 | 3.45 |
| 1 | 8.649424871 | 43 | 0 | 4.85 | 24 | 19 | 29 | 83 | 70.3 | 46.1 | 24.2 | 13.7 | 4.41 | 9.3 | 4.47 | 2.18 | 1.07 | 2.44 |
| 1 | 6.539354881 | 47 | 1 | 4.39 | 10 | 15 | 10 | 65 | 72.2 | 44 | 28.2 | 15.32 | 4.95 | 10.4 | 4.58 | 0.68 | 1.28 | 3.05 |
| 1 | 10.83224932 | 38 | 0 | 4.94 | 12 | 17 | 14 | 52 | 76.8 | 56 | 20.8 | 9.24 | 3.73 | 5.5 | 3.74 | 0.77 | 1.28 | 2.03 |
| 0 | 3.172339241 | 55 | 0 | 5.88 | 23 | 15 | 33 | 77 | 71.9 | 47.5 | 24.4 | 9.05 | 3.7 | 5.3 | 5.54 | 1.52 | 1.25 | 3.46 |
| 1 | 7.018592358 | 51 | 0 | 4.83 | 13 | 14 | 25 | 62 | 68.3 | 45.6 | 22.7 | 9.81 | 3.99 | 5.8 | 4.24 | 0.7 | 1.13 | 2.56 |
| 0 | 4.690864086 | 58 | 0 | 5.18 | 11 | 11 | 12 | 74 | 67.6 | 48.6 | 19 | 15.97 | 7.81 | 8.2 | 3.24 | 0.88 | 1.15 | 2.03 |
| 1 | 6.288739204 | 48 | 1 | 4.76 | 10 | 13 | 14 | 75 | 73.2 | 45.8 | 27.4 | 5.6 | 3.3 | 2.3 | 4.1 | 0.79 | 1.63 | 2.17 |
| 0 | 2.195898175 | 56 | 0 | 6.42 | 13 | 16 | 18 | 68 | 72 | 46.4 | 25.6 | 10.07 | 3.84 | 6.2 | 3.52 | 0.81 | 1.31 | 1.88 |
| 1 | 8.210445404 | 56 | 0 | 5.31 | 24 | 25 | 16 | 64 | 68.7 | 45.5 | 23.2 | 22.19 | 7.65 | 14.5 | 3.52 | 1.32 | 1.19 | 1.95 |
| 1 | 11.06369019 | 54 | 0 | 6.53 | 20 | 17 | 29 | 63 | 69.5 | 49.6 | 19.9 | 12.89 | 5.34 | 7.6 | 3.69 | 0.79 | 1.32 | 1.86 |
| 1 | 6.68206358 | 45 | 0 | 4.63 | 13 | 14 | 20 | 68 | 73.4 | 51 | 22.4 | 9.2 | 3.9 | 5.3 | 4.39 | 2.1 | 0.99 | 2.2 |
| 0 | 4.192169944 | 58 | 0 | 7.67 | 23 | 28 | 38 | 130 | 76.2 | 51.4 | 24.8 | 15.2 | 7 | 8.2 | 4.12 | 0.96 | 1.23 | 2.51 |
| 1 | 7.96463871 | 56 | 0 | 5.33 | 17 | 22 | 30 | 51 | 68.8 | 47.3 | 21.5 | 6.8 | 3.84 | 3 | 3.41 | 0.6 | 1.5 | 1.84 |
| 0 | 1.334613204 | 65 | 0 | 5.8 | 10 | 16 | 12 | 76 | 75.3 | 49.2 | 26.1 | 10.35 | 3.77 | 6.6 | 5.59 | 1.45 | 1.54 | 3.47 |
| 1 | 7.116610209 | 63 | 0 | 5.72 | 8 | 19 | 31 | 51 | 68.8 | 45.9 | 22.9 | 12.84 | 5.66 | 7.2 | 3.62 | 0.79 | 1.63 | 1.82 |
| 2 | 18.06303151 | 58 | 0 | 7.11 | 45 | 29 | 211 | 99 | 66 | 45.3 | 20.7 | 11 | 4.2 | 6.8 | 6.57 | 1.81 | 1.91 | 4.08 |
| 1 | 5.00201726 | 58 | 0 | 4.48 | 27 | 21 | 13 | 65 | 67.9 | 44.4 | 23.5 | 11 | 3.5 | 7.5 | 3.98 | 0.97 | 1.21 | 2.4 |
| 1 | 8.093506336 | 54 | 0 | 5.32 | 18 | 20 | 17 | 57 | 72.1 | 45.6 | 26.5 | 7.28 | 2.72 | 4.6 | 4.35 | 1.34 | 0.99 | 2.87 |
| 1 | 5.340483109 | 59 | 0 | 5.78 | 18 | 19 | 57 | 71 | 74.8 | 46.8 | 28 | 8.1 | 3.21 | 4.9 | 5.43 | 2.68 | 1.2 | 3.27 |
| 1 | 8.587590853 | 58 | 0 | 5.37 | 31 | 37 | 129 | 68 | 73.4 | 44.6 | 28.8 | 17.44 | 8.76 | 8.7 | 5.19 | 2.05 | 1.51 | 3.22 |
| 1 | 12.53913164 | 57 | 0 | 7.32 | 11 | 14 | 19 | 87 | 68.8 | 48.5 | 20.3 | 14.15 | 6.5 | 7.7 | 4.03 | 1.41 | 1.05 | 2.25 |
| 1 | 8.156013807 | 53 | 1 | 5.5 | 26 | 20 | 12 | 80 | 70.1 | 47.1 | 23 | 6.32 | 2.46 | 3.9 | 6.76 | 4.93 | 0.93 | 3.4 |
| 1 | 11.65074539 | 57 | 0 | 5.85 | 30 | 30 | 19 | 72 | 74.5 | 48.4 | 26.1 | 10.7 | 4.9 | 5.8 | 3.12 | 2.2 | 1.22 | 1.12 |
| 1 | 11.81120872 | 68 | 0 | 6.02 | 13 | 16 | 17 | 71 | 67.1 | 50.2 | 16.9 | 12.17 | 5.38 | 6.8 | 4.88 | 0.69 | 1.3 | 2.95 |
| 0 | 4.41958348 | 44 | 0 | 5.57 | 22 | 22 | 28 | 99 | 69.4 | 47.3 | 22.1 | 7.7 | 3 | 4.7 | 4.83 | 1.95 | 1.21 | 2.76 |
| 1 | 6.140256246 | 41 | 0 | 6.05 | 17 | 16 | 31 | 99 | 72.2 | 50.6 | 21.6 | 8.18 | 3.32 | 4.9 | 3.52 | 1.37 | 1.4 | 1.73 |
| 0 | 2.959303856 | 60 | 0 | 5.7 | 6 | 13 | 22 | 64 | 72.5 | 50.2 | 22.3 | 4.4 | 1.96 | 2.4 | 4.85 | 3.87 | 1.11 | 1.95 |
| 0 | 3.375711004 | 50 | 1 | 5.33 | 27 | 24 | 12 | 66 | 72.9 | 48.3 | 24.6 | 10.3 | 3.9 | 6.4 | 3.89 | 2.46 | 1 | 2 |
| 1 | 5.120604912 | 38 | 0 | 5.54 | 25 | 17 | 13 | 52 | 80.2 | 49.5 | 30.7 | 12.57 | 3.98 | 8.6 | 5.96 | 1.85 | 1.59 | 4.05 |
| 0 | 3.122495413 | 54 | 1 | 4.96 | 18 | 21 | 10 | 77 | 69.9 | 43.6 | 26.3 | 9.36 | 4.4 | 5 | 3.35 | 0.82 | 1.58 | 1.72 |
| 1 | 6.020764987 | 51 | 0 | 5.09 | 16 | 16 | 11 | 62 | 66.5 | 46.3 | 20.2 | 15.16 | 5.23 | 9.9 | 5.21 | 1.3 | 1.22 | 3.22 |
| 2 | 19.67808787 | 48 | 0 | 6.77 | 30 | 21 | 61 | 55 | 68 | 44.9 | 23.1 | 9.49 | 5.34 | 4.2 | 3.35 | 1.45 | 1.02 | 1.88 |
| 2 | 24.23605919 | 52 | 0 | 5.03 | 41 | 31 | 46 | 83 | 69.1 | 48 | 21.1 | 16.94 | 6.12 | 10.8 | 4.92 | 1.77 | 1.1 | 2.89 |
| 1 | 8.300377925 | 59 | 0 | 8.57 | 19 | 19 | 35 | 57 | 75.2 | 51.2 | 24 | 16.2 | 5.5 | 10.7 | 4.27 | 3.57 | 1.01 | 2.21 |
| 1 | 6.105370363 | 57 | 0 | 4.99 | 15 | 19 | 19 | 59 | 66.8 | 48.2 | 18.6 | 20.79 | 6.09 | 14.7 | 4.99 | 1.33 | 1.15 | 3.16 |
| 0 | 2.856259108 | 54 | 1 | 5.77 | 12 | 20 | 13 | 109 | 75.3 | 48.2 | 27.1 | 10.4 | 4.5 | 5.9 | 5.09 | 1.36 | 1.52 | 3.06 |
| 1 | 6.49178497 | 55 | 1 | 5.48 | 19 | 25 | 54 | 59 | 73.3 | 49.4 | 23.9 | 10.14 | 3.95 | 6.2 | 5.03 | 1.08 | 1.85 | 2.53 |
| 1 | 11.23453299 | 53 | 1 | 6.81 | 46 | 41 | 36 | 74 | 69.3 | 50.5 | 18.8 | 13.6 | 4.83 | 8.8 | 5.76 | 1.66 | 1.5 | 3.48 |
| 1 | 13.18259525 | 52 | 0 | 7.27 | 31 | 18 | 63 | 71 | 67.6 | 44.3 | 23.3 | 3.5 | 2 | 1.5 | 5.72 | 6.98 | 0.83 | 2.62 |
| 0 | 1.580408891 | 56 | 1 | 4.89 | 15 | 23 | 11 | 75 | 71.7 | 45.5 | 26.2 | 8.29 | 4 | 4.3 | 4.95 | 0.87 | 1.76 | 3.19 |
| 0 | 1.52102534 | 52 | 1 | 4.39 | 13 | 20 | 11 | 87 | 74.2 | 48.1 | 26.1 | 5.83 | 2.29 | 3.5 | 7.08 | 1.05 | 2.4 | 4.51 |
| 2 | 19.01496696 | 53 | 0 | 7.08 | 18 | 15 | 53 | 83 | 74.2 | 47.9 | 26.3 | 8.92 | 4.11 | 4.8 | 2.87 | 2.3 | 1.14 | 1.09 |
| 0 | 2.119082093 | 55 | 1 | 5.24 | 29 | 27 | 18 | 81 | 70.4 | 49.4 | 21 | 14.01 | 5.45 | 8.6 | 5.37 | 1.52 | 1.52 | 3.52 |
| 1 | 9.695449511 | 58 | 1 | 4.83 | 21 | 23 | 12 | 70 | 70.8 | 43.6 | 27.2 | 5.7 | 2.8 | 2.9 | 5.23 | 1.58 | 1.11 | 3.6 |
| 0 | 4.826952934 | 56 | 1 | 5.12 | 24 | 26 | 11 | 93 | 79.8 | 47.9 | 31.9 | 13.3 | 6.8 | 6.5 | 7.08 | 1.73 | 1.61 | 4.74 |
| 1 | 7.33912913 | 42 | 1 | 4.82 | 19 | 16 | 19 | 56 | 81.6 | 49.1 | 32.5 | 7.2 | 2.8 | 4.4 | 4.16 | 1.35 | 1.47 | 2.25 |
| 2 | 21.43030103 | 44 | 0 | 5.38 | 49 | 25 | 37 | 93 | 72.8 | 48.6 | 24.2 | 9.15 | 3.6 | 5.6 | 4.27 | 2.31 | 1.12 | 2.24 |
| 1 | 7.239896456 | 44 | 1 | 5.8 | 18 | 18 | 28 | 48 | 71.6 | 49 | 22.6 | 7.45 | 2.7 | 4.8 | 5.57 | 2.49 | 1.2 | 3.18 |
| 1 | 10.59536918 | 35 | 0 | 4.73 | 50 | 21 | 146 | 95 | 74.9 | 49.7 | 25.2 | 13.44 | 5.16 | 8.3 | 4.89 | 1.19 | 0.94 | 3.07 |
| 2 | 18.79521402 | 43 | 0 | 4.58 | 31 | 28 | 36 | 56 | 71.4 | 48 | 23.4 | 10.08 | 5.09 | 5 | 5.04 | 6.07 | 1.49 | 2.5 |
| 2 | 20.35814222 | 48 | 1 | 7.11 | 14 | 16 | 54 | 66 | 68.1 | 46.5 | 21.6 | 7.68 | 3.34 | 4.3 | 5.17 | 2.19 | 1.3 | 3.3 |
| 0 | 1.355605761 | 55 | 1 | 5.52 | 25 | 29 | 87 | 134 | 75 | 45.6 | 29.4 | 15.5 | 6.8 | 8.7 | 4.45 | 1.05 | 1.19 | 2.99 |
| 1 | 11.3023119 | 65 | 1 | 9.63 | 18 | 15 | 16 | 75 | 70.8 | 44.9 | 25.9 | 7.07 | 3.19 | 3.9 | 3.63 | 3.31 | 0.92 | 1.79 |
| 0 | 4.241495371 | 51 | 0 | 5.26 | 21 | 19 | 49 | 77 | 71.1 | 44.8 | 26.3 | 13.02 | 5.57 | 7.4 | 4.97 | 3.37 | 1.2 | 2.61 |
| 1 | 7.212303321 | 55 | 1 | 5.49 | 18 | 20 | 18 | 90 | 72.9 | 45.7 | 27.2 | 15.16 | 5.58 | 9.6 | 3.06 | 1.66 | 1.37 | 1.24 |
| 0 | 1.416325132 | 56 | 1 | 5.02 | 10 | 15 | 9 | 71 | 75.2 | 44.2 | 31 | 8.47 | 3.99 | 4.5 | 5.01 | 1.44 | 1.58 | 3.05 |
| 2 | 25.66531245 | 55 | 0 | 6.07 | 55 | 28 | 56 | 59 | 64.3 | 45.5 | 18.8 | 5.72 | 2.32 | 3.4 | 4.29 | 1.45 | 1.35 | 2.49 |
| 1 | 7.605488459 | 55 | 1 | 5.41 | 33 | 23 | 43 | 111 | 67.5 | 45.4 | 22.1 | 15.29 | 4.46 | 10.8 | 6.44 | 2.91 | 1.43 | 4.11 |
| 1 | 6.161235968 | 53 | 1 | 5.14 | 16 | 21 | 10 | 101 | 79.7 | 51.7 | 28 | 6.7 | 2.3 | 4.4 | 7.08 | 1.84 | 1.83 | 4.45 |
| 0 | 4.502149343 | 59 | 0 | 4.69 | 18 | 22 | 32 | 104 | 72.8 | 47.9 | 24.9 | 6.52 | 2.59 | 3.9 | 6.45 | 2.83 | 1.06 | 4.38 |
| 0 | 1.631181637 | 40 | 1 | 4.93 | 12 | 17 | 11 | 43 | 71.8 | 45 | 26.8 | 8.02 | 3.2 | 4.8 | 5.13 | 1.21 | 1.58 | 2.87 |
| 0 | 2.315658212 | 57 | 1 | 4.71 | 15 | 17 | 17 | 72 | 76.2 | 49 | 27.2 | 9 | 4 | 5 | 6.27 | 1.32 | 1.61 | 4.26 |
| 1 | 5.617451191 | 57 | 1 | 5.58 | 18 | 18 | 14 | 75 | 73.9 | 48.4 | 25.5 | 35.88 | 9.86 | 26 | 5.26 | 0.98 | 1.36 | 3.37 |
| 1 | 6.75043424 | 60 | 0 | 6.36 | 16 | 18 | 22 | 44 | 74.2 | 45.8 | 28.4 | 14.69 | 5.51 | 9.2 | 4.58 | 2.03 | 0.99 | 2.87 |
| 0 | 4.577756166 | 43 | 0 | 5.21 | 20 | 25 | 159 | 67 | 69.6 | 50.9 | 18.7 | 10.94 | 4.02 | 6.9 | 5.74 | 1.36 | 1.54 | 3.55 |
| 1 | 10.66775846 | 49 | 0 | 9.03 | 22 | 17 | 90 | 64 | 71.9 | 49.9 | 22 | 8.98 | 3.5 | 5.5 | 4.88 | 3.2 | 1.61 | 1.6 |
| 0 | 3.097937425 | 55 | 0 | 5.81 | 15 | 15 | 45 | 77 | 66.5 | 44.9 | 21.6 | 10.32 | 4.7 | 5.6 | 4.57 | 1.27 | 1.98 | 2.19 |
| 1 | 6.38653485 | 56 | 0 | 6.8 | 10 | 17 | 20 | 66 | 74.6 | 47.8 | 26.8 | 14.4 | 6.8 | 7.6 | 3.38 | 0.97 | 1.61 | 1.6 |
| 0 | 4.562403202 | 47 | 1 | 4.52 | 11 | 17 | 14 | 62 | 72.2 | 45.7 | 26.5 | 8 | 3.8 | 4.2 | 4.68 | 0.96 | 1.52 | 2.97 |
| 0 | 4.169025024 | 59 | 0 | 4.91 | 15 | 18 | 18 | 54 | 72.8 | 47 | 25.8 | 11.62 | 4.03 | 7.6 | 6.08 | 1.64 | 0.95 | 4.11 |
| 1 | 7.576126099 | 58 | 1 | 4.74 | 21 | 26 | 12 | 90 | 80.2 | 48.3 | 31.9 | 12.34 | 4.26 | 8.1 | 5.42 | 1.17 | 1.34 | 3.41 |
| 0 | 2.106735508 | 62 | 1 | 4.78 | 19 | 28 | 12 | 81 | 71.4 | 45.7 | 25.7 | 16.01 | 7.42 | 8.6 | 4.11 | 0.7 | 1.74 | 2.23 |
| 0 | 3.34377861 | 54 | 0 | 5 | 7 | 13 | 20 | 52 | 72.3 | 47.3 | 25 | 11.2 | 5.6 | 5.6 | 4.14 | 0.94 | 1.31 | 2.59 |
| 1 | 6.306643168 | 59 | 0 | 10.73 | 44 | 28 | 23 | 55 | 74.4 | 46 | 28.4 | 7.38 | 3.96 | 3.4 | 3.39 | 1.09 | 1.08 | 1.89 |
| 2 | 27.19657453 | 44 | 0 | 5.6 | 46 | 26 | 47 | 72 | 70.9 | 44.5 | 26.4 | 13.2 | 5.2 | 8 | 4.17 | 2.45 | 0.92 | 2.6 |
| 0 | 1.652965585 | 52 | 0 | 5.82 | 9 | 16 | 11 | 59 | 68.1 | 48.7 | 19.4 | 8.83 | 4.8 | 4 | 2.9 | 0.8 | 0.95 | 1.78 |
| 0 | 1.221473237 | 57 | 0 | 4.62 | 17 | 19 | 21 | 41 | 70.6 | 45.2 | 25.4 | 8.76 | 3.87 | 4.9 | 4.34 | 0.85 | 1.27 | 2.71 |
| 1 | 12.76407782 | 56 | 0 | 6 | 26 | 20 | 19 | 81 | 76.6 | 52.2 | 24.4 | 8.22 | 3.36 | 4.9 | 4.56 | 1.81 | 1.05 | 2.68 |
| 1 | 5.607927402 | 59 | 0 | 4.69 | 24 | 26 | 22 | 63 | 69.5 | 49.4 | 20.1 | 7.6 | 3.51 | 4.1 | 3.97 | 1.28 | 1.85 | 1.66 |
| 1 | 7.715447585 | 56 | 0 | 6.3 | 34 | 31 | 28 | 74 | 77.7 | 50.2 | 27.5 | 22.07 | 7.78 | 14.3 | 4.44 | 1.52 | 1.17 | 2.62 |
| 0 | 1.61639901 | 50 | 1 | 5.36 | 23 | 25 | 16 | 84 | 78.2 | 50.1 | 28.1 | 10.91 | 4.52 | 6.4 | 6.58 | 0.84 | 2 | 4.28 |
| 1 | 10.9362793 | 50 | 1 | 4.95 | 23 | 27 | 14 | 48 | 66.4 | 44.1 | 22.3 | 5.58 | 2.57 | 3 | 3.93 | 0.84 | 1.54 | 2.03 |
| 0 | 0.704980175 | 46 | 1 | 4.71 | 9 | 19 | 18 | 56 | 74.2 | 46.2 | 28 | 17.16 | 6.32 | 10.8 | 5.15 | 1.24 | 1.34 | 3.17 |
| 1 | 7.596725464 | 56 | 0 | 4.76 | 25 | 23 | 20 | 63 | 74 | 45.4 | 28.6 | 18.3 | 6.5 | 11.8 | 4.01 | 2.69 | 0.9 | 2.34 |
| 1 | 9.51314799 | 55 | 1 | 5.26 | 18 | 18 | 22 | 78 | 76.6 | 45.2 | 31.4 | 6.8 | 3.3 | 3.5 | 5.15 | 2.04 | 1 | 3.5 |
| 2 | 19.85657692 | 54 | 1 | 7.7 | 27 | 23 | 28 | 95 | 74.3 | 47.8 | 26.5 | 10.31 | 5.03 | 5.3 | 4.47 | 3.6 | 1.04 | 2.19 |
| 1 | 6.130313396 | 51 | 0 | 5.09 | 27 | 36 | 62 | 86 | 80.9 | 51 | 29.9 | 7.9 | 0.81 | 7.1 | 4.42 | 2.75 | 1.13 | 2.22 |
| 1 | 8.924859206 | 46 | 0 | 5.79 | 19 | 22 | 12 | 79 | 76.5 | 47.8 | 28.7 | 10.1 | 4.4 | 5.7 | 3.8 | 1.37 | 1.08 | 2.31 |
| 1 | 6.803826491 | 53 | 0 | 5.05 | 14 | 19 | 15 | 80 | 69.7 | 45.2 | 24.5 | 7.26 | 3.07 | 4.2 | 4.08 | 0.93 | 1.21 | 2.3 |
| 2 | 19.21141624 | 50 | 0 | 5.23 | 37 | 21 | 50 | 74 | 69.5 | 50.7 | 18.8 | 13.58 | 5.22 | 8.4 | 5.12 | 3.1 | 1.2 | 2.93 |
| 1 | 12.39757983 | 57 | 1 | 5.05 | 160 | 114 | 54 | 118 | 74.3 | 44 | 30.3 | 7.44 | 3.29 | 4.2 | 6.6 | 1.13 | 1.74 | 4.15 |
| 2 | 17.99226824 | 51 | 0 | 5.85 | 22 | 21 | 19 | 123 | 73.2 | 44.9 | 28.3 | 13.05 | 4.05 | 9 | 3.37 | 1.38 | 1.08 | 1.72 |
| 1 | 5.627495925 | 57 | 1 | 4.78 | 15 | 18 | 28 | 84 | 67.6 | 45.4 | 22.2 | 11.87 | 4.17 | 7.7 | 4.42 | 2.22 | 1.23 | 2.29 |
| 1 | 5.854144573 | 46 | 1 | 4.91 | 13 | 16 | 9 | 49 | 68.7 | 46.1 | 22.6 | 15.2 | 7.3 | 7.9 | 4.01 | 0.63 | 1.7 | 2.27 |
| 0 | 0.947789431 | 33 | 0 | 4.78 | 29 | 26 | 132 | 69 | 78.5 | 49.6 | 28.9 | 9.53 | 4.43 | 5.1 | 5.02 | 1.71 | 1.49 | 3.08 |
| 1 | 9.228204409 | 47 | 1 | 4.52 | 17 | 18 | 12 | 56 | 68.9 | 47.6 | 21.3 | 5.62 | 2.81 | 2.8 | 4.37 | 0.97 | 1.57 | 2.36 |
| 0 | 1.124600887 | 50 | 0 | 5.33 | 12 | 15 | 77 | 32 | 62.9 | 48.5 | 14.4 | 15.2 | 5.41 | 9.8 | 5.02 | 1.84 | 1.23 | 3.06 |
| 0 | 2.745414615 | 44 | 0 | 4.9 | 24 | 16 | 24 | 89 | 71.5 | 46.4 | 25.1 | 7 | 3.4 | 3.6 | 2.68 | 1 | 1.24 | 1.18 |
| 2 | 18.39798164 | 62 | 0 | 8.34 | 35 | 26 | 62 | 45 | 67.1 | 47.6 | 19.5 | 9.42 | 5.79 | 3.6 | 2.86 | 1.24 | 1.11 | 1.27 |
| 0 | 3.945651293 | 60 | 0 | 5.67 | 16 | 20 | 16 | 60 | 71.1 | 44.1 | 27 | 11.39 | 5.92 | 5.5 | 2.67 | 0.83 | 1.23 | 1.11 |
| 1 | 8.813873132 | 56 | 0 | 5.63 | 17 | 23 | 28 | 49 | 72.7 | 46.1 | 26.6 | 13.79 | 4.62 | 9.2 | 6.7 | 4.51 | 0.98 | 3.18 |
| 0 | 0.121753375 | 46 | 1 | 6.23 | 13 | 17 | 21 | 80 | 76.9 | 49.6 | 27.3 | 10.81 | 4.03 | 6.8 | 5.13 | 1.89 | 1.19 | 3.32 |
| 0 | 3.445402781 | 42 | 1 | 4.94 | 14 | 17 | 10 | 56 | 66.8 | 43 | 23.8 | 4.46 | 2.17 | 2.3 | 4.69 | 0.83 | 1.5 | 2.68 |
| 0 | 2.949585279 | 40 | 1 | 5.35 | 10 | 17 | 6 | 57 | 74 | 48.4 | 25.6 | 7.1 | 4.36 | 2.7 | 3.42 | 0.97 | 1.36 | 1.69 |
| 1 | 9.119846026 | 43 | 0 | 5.35 | 27 | 22 | 37 | 98 | 69 | 47.8 | 21.2 | 7.8 | 3.3 | 4.5 | 3.8 | 4.7 | 0.85 | 1.66 |
| 1 | 7.950265249 | 58 | 1 | 5.31 | 226 | 138 | 26 | 92 | 72.7 | 49.6 | 23.1 | 11.12 | 4.5 | 6.6 | 4.29 | 0.69 | 1.89 | 2.23 |
| 1 | 11.04773426 | 42 | 0 | 5.39 | 11 | 12 | 12 | 88 | 73.1 | 44 | 29.1 | 7.06 | 2.53 | 4.5 | 3.92 | 1.2 | 1.02 | 2.35 |
| 1 | 9.099876086 | 85 | 0 | 4.95 | 15 | 17 | 183 | 133 | 70 | 41.5 | 28.5 | 36.03 | 13.46 | 22.6 | 4.32 | 0.92 | 1.29 | 2.59 |
| 1 | 6.037306786 | 58 | 0 | 6.14 | 26 | 26 | 17 | 116 | 69.9 | 46.3 | 23.6 | 6.71 | 2.65 | 4.1 | 4.71 | 2.04 | 1.04 | 3.01 |
| 0 | 4.908561865 | 52 | 0 | 3.98 | 13 | 20 | 18 | 84 | 78 | 49.9 | 28.1 | 16.4 | 7.35 | 9.1 | 4.94 | 0.88 | 1.46 | 3.14 |
| 1 | 9.088149548 | 60 | 0 | 6.92 | 25 | 24 | 21 | 42 | 70.2 | 49.2 | 21 | 20 | 6.35 | 13.6 | 4.12 | 1.46 | 1.13 | 2.47 |
| 1 | 8.438828627 | 48 | 0 | 7.56 | 17 | 13 | 15 | 71 | 66.3 | 47 | 19.3 | 9.67 | 4.5 | 5.2 | 5.22 | 3.5 | 1.01 | 2.68 |
| 1 | 13.50095463 | 52 | 0 | 4.74 | 28 | 27 | 22 | 74 | 79.1 | 52.6 | 26.5 | 21 | 7.4 | 13.6 | 4.7 | 1.5 | 0.9 | 3.24 |
| 0 | 4.66830047 | 54 | 0 | 5.89 | 18 | 16 | 30 | 87 | 63.8 | 45.4 | 18.4 | 12 | 5 | 7 | 3.96 | 2.13 | 1.05 | 2.26 |
| 0 | 1.322387497 | 58 | 0 | 5.14 | 19 | 22 | 19 | 70 | 76.3 | 47.4 | 28.9 | 13.36 | 6.01 | 7.3 | 3.91 | 0.68 | 1.15 | 2.44 |
| 0 | 3.742568851 | 59 | 1 | 5.06 | 12 | 15 | 9 | 91 | 76.1 | 46 | 30.1 | 10.34 | 3.68 | 6.7 | 5.47 | 2.1 | 1.32 | 3.28 |
| 1 | 6.84814469 | 45 | 0 | 6.19 | 21 | 16 | 42 | 50 | 70.4 | 46.9 | 23.5 | 10.99 | 3.94 | 7.1 | 6.43 | 1.64 | 1.29 | 4.48 |
| 1 | 7.837669849 | 65 | 1 | 5.23 | 9 | 15 | 11 | 52 | 69.7 | 45.6 | 24.1 | 11.67 | 4.89 | 6.8 | 3.99 | 0.85 | 1.73 | 1.95 |
| 0 | 3.935236295 | 63 | 1 | 6.37 | 12 | 16 | 14 | 85 | 70.3 | 45.4 | 24.9 | 8.28 | 3.44 | 4.8 | 3.96 | 1.46 | 1.06 | 2.44 |
| 1 | 11.05056826 | 59 | 1 | 5.15 | 36 | 23 | 45 | 85 | 71.1 | 49.6 | 21.5 | 7.2 | 3.52 | 3.7 | 5.99 | 1.46 | 1.83 | 3.56 |
| 1 | 13.03295104 | 57 | 0 | 6.05 | 36 | 35 | 76 | 77 | 71.9 | 48.6 | 23.3 | 13.3 | 4.9 | 8.4 | 5.55 | 3.22 | 1.6 | 2.93 |
| 1 | 7.002667427 | 46 | 0 | 5.38 | 31 | 24 | 24 | 70 | 71.8 | 46.4 | 25.4 | 13.1 | 5.1 | 8 | 3.6 | 1.46 | 1.04 | 2.04 |
| 1 | 8.604034265 | 46 | 1 | 5.43 | 12 | 13 | 14 | 47 | 73.3 | 48.7 | 24.6 | 9.7 | 4.25 | 5.4 | 4.23 | 1.06 | 1.5 | 2.46 |
| 1 | 13.21637026 | 51 | 1 | 5.03 | 32 | 22 | 16 | 80 | 77.2 | 48.5 | 28.7 | 11.32 | 4.27 | 7.1 | 4.97 | 2.99 | 1.08 | 2.85 |
| 0 | 3.341058413 | 46 | 0 | 5.08 | 19 | 14 | 47 | 74 | 69.9 | 49.1 | 20.8 | 13.15 | 5.35 | 7.8 | 4.66 | 1.98 | 1.06 | 2.88 |
| 1 | 5.538266341 | 45 | 1 | 5.13 | 17 | 13 | 15 | 46 | 72.4 | 48 | 24.4 | 13.86 | 4.07 | 9.8 | 6.02 | 1.17 | 1.26 | 4.07 |
| 1 | 5.086784005 | 49 | 0 | 5.65 | 36 | 22 | 43 | 77 | 75.6 | 54 | 21.6 | 11.3 | 4.6 | 6.7 | 4.63 | 2.21 | 0.86 | 2.95 |
| 2 | 23.58496539 | 48 | 1 | 5.46 | 107 | 65 | 22 | 62 | 74.4 | 46.6 | 27.8 | 5.71 | 2.79 | 2.9 | 5.16 | 1.92 | 0.96 | 3.35 |
| 1 | 8.026299636 | 36 | 0 | 5.55 | 20 | 16 | 23 | 95 | 73.7 | 52 | 21.7 | 14.49 | 5 | 9.5 | 6.14 | 1.45 | 1.45 | 4.23 |
| 1 | 11.89558427 | 52 | 0 | 6.89 | 18 | 17 | 25 | 70 | 77.6 | 49.6 | 28 | 11.6 | 4.6 | 7 | 4.63 | 1.97 | 1.24 | 2.83 |
| 1 | 10.74482695 | 56 | 0 | 6.75 | 17 | 16 | 21 | 88 | 62.4 | 46 | 16.4 | 12.82 | 6.19 | 6.6 | 3.95 | 1.74 | 1.12 | 2.36 |
| 0 | 2.163961053 | 34 | 0 | 4.99 | 28 | 27 | 30 | 83 | 80.9 | 54.4 | 26.5 | 8.5 | 3.1 | 5.4 | 5.57 | 3.59 | 0.93 | 3.56 |
| 1 | 5.471386433 | 59 | 1 | 5.25 | 13 | 16 | 18 | 76 | 68.3 | 48.6 | 19.7 | 4.1 | 2.5 | 1.6 | 4.23 | 0.59 | 1.77 | 2.17 |
| 1 | 10.98812866 | 51 | 1 | 9.93 | 24 | 18 | 27 | 101 | 73.6 | 46.3 | 27.3 | 5.5 | 2.6 | 2.9 | 4.96 | 1.3 | 1.33 | 3.16 |
| 0 | 1.444567124 | 51 | 0 | 5.21 | 22 | 22 | 33 | 73 | 71.9 | 53.9 | 18 | 13.7 | 5.7 | 8 | 4.25 | 2 | 1.17 | 2.29 |
| 0 | 4.237050136 | 60 | 0 | 9.16 | 177 | 78 | 75 | 82 | 76.5 | 51.2 | 25.3 | 13.23 | 6.86 | 6.4 | 3.92 | 0.97 | 1.61 | 1.96 |
| 0 | 3.571582158 | 53 | 1 | 5.66 | 13 | 16 | 13 | 86 | 73.8 | 47.3 | 26.5 | 20.77 | 6.7 | 14.1 | 6.32 | 1.92 | 1.24 | 4.13 |
| 1 | 5.999420563 | 65 | 0 | 5.76 | 16 | 20 | 9 | 60 | 73.5 | 45.2 | 28.3 | 12.3 | 5.9 | 6.4 | 2.58 | 0.52 | 1.31 | 1.16 |
| 1 | 10.40036058 | 48 | 1 | 8.15 | 18 | 21 | 12 | 112 | 76.4 | 50.1 | 26.3 | 8.3 | 3.3 | 5 | 5.96 | 1.39 | 1.45 | 3.98 |
| 0 | 3.83369875 | 52 | 1 | 5.13 | 20 | 24 | 13 | 70 | 73.2 | 49.9 | 23.3 | 6.58 | 3.32 | 3.3 | 5.28 | 0.57 | 2.08 | 2.9 |
| 1 | 9.814539115 | 64 | 0 | 4.54 | 12 | 17 | 16 | 74 | 72 | 45.9 | 26.1 | 6.8 | 3.53 | 3.3 | 5.54 | 1.03 | 1.16 | 3.88 |
| 1 | 6.544877172 | 56 | 1 | 5.88 | 15 | 18 | 16 | 76 | 76.2 | 47.3 | 28.9 | 5.3 | 1.9 | 3.4 | 4.73 | 1.23 | 1.19 | 2.95 |
| 0 | 4.44584771 | 58 | 0 | 5.47 | 23 | 16 | 70 | 78 | 72.6 | 47.9 | 24.7 | 7.86 | 3.99 | 3.9 | 4.61 | 2.54 | 1.09 | 2.51 |
| 1 | 6.678083897 | 54 | 1 | 6.12 | 6 | 17 | 14 | 67 | 71.6 | 43.2 | 28.4 | 4.04 | 1.92 | 2.1 | 6.14 | 3.22 | 1.19 | 3.58 |
| 0 | 4.194999615 | 49 | 1 | 4.44 | 10 | 14 | 15 | 54 | 64.6 | 42.5 | 22.1 | 5.21 | 2.86 | 2.3 | 4.71 | 1.58 | 1.05 | 2.92 |
| 1 | 11.85706615 | 57 | 0 | 4.81 | 20 | 21 | 23 | 93 | 72.9 | 45.7 | 27.2 | 6.6 | 2.9 | 3.7 | 3.91 | 1.65 | 0.9 | 2.37 |
| 1 | 8.19850572 | 59 | 0 | 5.91 | 32 | 25 | 17 | 127 | 70.5 | 47.7 | 22.8 | 9.88 | 4.33 | 5.6 | 2.37 | 0.95 | 0.85 | 0.95 |
| 1 | 7.186012745 | 55 | 0 | 5.31 | 17 | 18 | 23 | 81 | 75 | 47.5 | 27.5 | 23.6 | 8.2 | 15.4 | 4.05 | 1.21 | 0.96 | 2.68 |
| 0 | 2.096445441 | 56 | 0 | 5.24 | 9 | 14 | 10 | 61 | 67.8 | 41.3 | 26.5 | 22.41 | 7.1 | 15.3 | 4.17 | 1.38 | 1.48 | 2.1 |
| 1 | 7.466523488 | 50 | 1 | 5.57 | 19 | 21 | 22 | 106 | 66 | 45.2 | 20.8 | 9.92 | 3.55 | 6.4 | 5.11 | 2.1 | 1.06 | 3.44 |
| 1 | 6.691175779 | 54 | 1 | 5.48 | 34 | 41 | 75 | 91 | 70.6 | 45.8 | 24.8 | 13.44 | 4.55 | 8.9 | 6.22 | 1.76 | 1.34 | 4.36 |
| 1 | 6.624719938 | 48 | 1 | 5.08 | 20 | 18 | 12 | 59 | 74.7 | 50.8 | 23.9 | 12.2 | 5.36 | 6.8 | 4.11 | 0.54 | 1.75 | 2.16 |
| 1 | 6.544791063 | 50 | 0 | 5.14 | 12 | 18 | 12 | 70 | 69 | 46 | 23 | 13.49 | 3.92 | 9.6 | 3.51 | 0.65 | 1.3 | 1.93 |
| 1 | 8.06755956 | 42 | 1 | 5.32 | 10 | 13 | 6 | 60 | 73.7 | 48.6 | 25.1 | 12.5 | 4.6 | 7.9 | 4.65 | 2.28 | 1.17 | 2.88 |
| 1 | 5.086894433 | 39 | 0 | 5.22 | 18 | 18 | 28 | 74 | 71.4 | 47.6 | 23.8 | 14.32 | 5.88 | 8.4 | 3.87 | 3.53 | 0.84 | 2.07 |
| 2 | 19.67602921 | 51 | 0 | 5.92 | 39 | 27 | 37 | 88 | 77.4 | 48.7 | 28.7 | 7.73 | 3.29 | 4.4 | 5.48 | 8.43 | 0.98 | 1.83 |
| 2 | 18.11618233 | 54 | 1 | 6.17 | 44 | 29 | 43 | 103 | 85.2 | 54.3 | 30.9 | 9.28 | 4.1 | 5.2 | 6.17 | 0.88 | 1.98 | 3.61 |
| 1 | 5.526077747 | 43 | 1 | 5.02 | 12 | 16 | 11 | 59 | 71.2 | 45.9 | 25.3 | 6.81 | 3.28 | 3.5 | 3.71 | 1.01 | 1.74 | 1.82 |
| 1 | 12.77926127 | 66 | 0 | 8.24 | 24 | 20 | 27 | 78 | 69.4 | 45.9 | 23.5 | 7.44 | 2.65 | 4.8 | 4.47 | 2.65 | 0.84 | 2.54 |
| 1 | 10.23480097 | 54 | 0 | 4.98 | 52 | 29 | 29 | 86 | 67 | 45.5 | 21.5 | 15.12 | 7.9 | 7.2 | 4.2 | 0.64 | 1.38 | 2.67 |
| 1 | 9.649420738 | 54 | 0 | 5.53 | 31 | 25 | 54 | 61 | 76.9 | 48.2 | 28.7 | 8.1 | 3.8 | 4.3 | 4.11 | 1.46 | 1.09 | 2.43 |
| 0 | 3.121699651 | 49 | 1 | 4.45 | 43 | 36 | 23 | 77 | 72 | 44.6 | 27.4 | 9.29 | 2.8 | 6.5 | 5.09 | 1.52 | 1.26 | 3.15 |
| 0 | 3.836483796 | 58 | 0 | 4.83 | 15 | 17 | 24 | 54 | 69.1 | 48.7 | 20.4 | 17.24 | 7.37 | 9.9 | 3.14 | 0.86 | 1.39 | 1.3 |
| 0 | 4.873148918 | 56 | 0 | 4.94 | 27 | 18 | 41 | 53 | 74 | 48.8 | 25.2 | 10.87 | 3.68 | 7.2 | 4.5 | 1.68 | 1.25 | 2.81 |
| 0 | 4.737646381 | 57 | 0 | 5.98 | 11 | 12 | 25 | 70 | 64.5 | 41.3 | 23.2 | 13.57 | 5.32 | 8.3 | 3.98 | 2.03 | 0.8 | 2.28 |
| 1 | 6.315451781 | 42 | 0 | 5.13 | 21 | 20 | 15 | 75 | 63.4 | 43 | 20.4 | 11.99 | 4.84 | 7.2 | 4.13 | 1.46 | 1.26 | 2.43 |
| 1 | 8.205509345 | 58 | 1 | 6.21 | 29 | 24 | 37 | 58 | 83.7 | 54.1 | 29.6 | 23.79 | 9.54 | 14.3 | 3.73 | 1.78 | 1.06 | 2.1 |
| 2 | 18.68363762 | 60 | 1 | 4.85 | 18 | 23 | 15 | 44 | 78.2 | 50.6 | 27.6 | 6.2 | 3.6 | 2.6 | 4.05 | 1.35 | 1.31 | 2.1 |
| 1 | 7.858098666 | 59 | 0 | 8.31 | 33 | 23 | 29 | 76 | 71.4 | 50 | 21.4 | 13.46 | 5.75 | 7.7 | 4.09 | 1.17 | 1.26 | 2.38 |
| 2 | 22.71943029 | 53 | 0 | 5.5 | 40 | 25 | 82 | 77 | 69.4 | 50.8 | 18.6 | 10.44 | 5.01 | 5.4 | 4.15 | 3.76 | 0.92 | 2.15 |
| 1 | 6.726830165 | 56 | 0 | 6.14 | 14 | 14 | 22 | 34 | 68.1 | 50.4 | 17.7 | 10.52 | 4.18 | 6.3 | 4.59 | 2.84 | 0.88 | 2.5 |
| 0 | 0.63822337 | 60 | 0 | 5.25 | 19 | 13 | 20 | 76 | 66.3 | 44.3 | 22 | 11.1 | 4.85 | 6.3 | 3.63 | 1.16 | 0.97 | 2.22 |
| 0 | 4.531770468 | 40 | 0 | 4.95 | 27 | 22 | 23 | 73 | 73.2 | 48.2 | 25 | 12.42 | 4.99 | 7.4 | 4.87 | 3.21 | 1.1 | 2.65 |
| 0 | 2.956656178 | 46 | 1 | 5.02 | 8 | 15 | 12 | 66 | 76.7 | 48.6 | 28.1 | 10.11 | 4.98 | 5.1 | 4.35 | 1.38 | 1.62 | 2.42 |
| 0 | 1.549744884 | 32 | 1 | 5.27 | 13 | 15 | 13 | 46 | 71.7 | 47.9 | 23.8 | 8.75 | 3.93 | 4.8 | 2.92 | 1.1 | 1.23 | 1.16 |
| 0 | 0.611118237 | 53 | 1 | 4.6 | 21 | 17 | 16 | 89 | 72.8 | 48.1 | 24.7 | 6.7 | 3 | 3.7 | 4.45 | 0.96 | 1.45 | 2.61 |
| 0 | 3.577573061 | 44 | 0 | 5.14 | 13 | 20 | 13 | 103 | 72.8 | 49.4 | 23.4 | 35.33 | 11.82 | 23.5 | 4.28 | 1.91 | 1.19 | 2.43 |
| 0 | 2.737690488 | 41 | 0 | 4.37 | 27 | 28 | 41 | 79 | 78.4 | 50.6 | 27.8 | 10.74 | 3.79 | 6.9 | 5.2 | 2.07 | 1.26 | 3.09 |
| 1 | 9.574687799 | 43 | 0 | 5.66 | 22 | 21 | 27 | 59 | 74.3 | 48.3 | 26 | 10.81 | 4.11 | 6.7 | 4.18 | 2.87 | 1.21 | 2.04 |
| 2 | 22.11975098 | 54 | 0 | 5.51 | 22 | 20 | 71 | 54 | 71.7 | 46.7 | 25 | 13.69 | 6.47 | 7.2 | 5.12 | 1.02 | 1.37 | 3.2 |
| 1 | 6.528107961 | 45 | 1 | 5.62 | 10 | 17 | 17 | 52 | 77.6 | 48.6 | 29 | 11.4 | 3.8 | 7.6 | 6.18 | 0.99 | 1.59 | 4.45 |
| 0 | 1.562350472 | 34 | 1 | 5.1 | 7 | 13 | 11 | 47 | 70.2 | 48.3 | 21.9 | 9.91 | 4.63 | 5.3 | 3.77 | 1.28 | 1.23 | 1.89 |
| 0 | 2.08295091 | 49 | 1 | 4.98 | 11 | 16 | 31 | 96 | 73.5 | 46.3 | 27.2 | 20.06 | 6.6 | 13.5 | 4.98 | 1.05 | 1.62 | 2.84 |
| 1 | 6.381524404 | 45 | 0 | 4.94 | 33 | 22 | 45 | 85 | 69.8 | 48.2 | 21.6 | 10.58 | 4.15 | 6.4 | 5.1 | 3.1 | 1.07 | 2.7 |
| 1 | 9.926803589 | 57 | 0 | 8.31 | 17 | 15 | 15 | 85 | 72.9 | 44.8 | 28.1 | 10.71 | 3.25 | 7.5 | 5 | 5.36 | 0.89 | 1.96 |
| 0 | 4.823727926 | 33 | 0 | 4.8 | 18 | 17 | 16 | 66 | 80 | 50.9 | 29.1 | 17.5 | 6.3 | 11.2 | 4.09 | 0.94 | 1.86 | 2.01 |
| 2 | 22.19373194 | 59 | 0 | 6.56 | 30 | 31 | 8 | 61 | 73.4 | 46.9 | 26.5 | 7.63 | 3.89 | 3.7 | 5.76 | 2.09 | 0.86 | 4.2 |
| 1 | 7.392938137 | 66 | 1 | 5.4 | 13 | 19 | 22 | 75 | 75.5 | 44.8 | 30.7 | 14.86 | 6.14 | 8.7 | 3.35 | 1.35 | 1.47 | 1.55 |
| 0 | 4.402731816 | 61 | 0 | 5.47 | 13 | 21 | 28 | 40 | 70.5 | 43.9 | 26.6 | 8.6 | 3.3 | 5.3 | 6.29 | 1.19 | 1.68 | 4.09 |
| 1 | 5.313557307 | 50 | 1 | 5.32 | 30 | 24 | 17 | 105 | 75.2 | 48.3 | 26.9 | 7.35 | 2.37 | 5 | 6.91 | 1.93 | 1.2 | 5 |
| 1 | 11.44139417 | 58 | 0 | 7.86 | 22 | 17 | 55 | 72 | 72.5 | 48.5 | 24 | 10.41 | 4.15 | 6.3 | 7.53 | 5.76 | 1.18 | 4.3 |
| 1 | 9.67245547 | 90 | 0 | 5.99 | 20 | 21 | 24 | 45 | 69.9 | 46.6 | 23.3 | 11.9 | 5.52 | 6.4 | 4.03 | 1.85 | 1.15 | 2.25 |
| 0 | 4.68028605 | 44 | 1 | 4.83 | 5 | 17 | 11 | 61 | 73.2 | 45.6 | 27.6 | 6.51 | 3.44 | 3.1 | 4.75 | 0.89 | 1.76 | 2.72 |
| 1 | 5.327597618 | 42 | 0 | 4.46 | 42 | 30 | 98 | 60 | 70.2 | 45.6 | 24.6 | 12.59 | 4.18 | 8.4 | 6.89 | 3 | 1.06 | 4.39 |
| 1 | 6.676098982 | 59 | 1 | 5.71 | 23 | 19 | 16 | 78 | 70.8 | 44.7 | 26.1 | 7.6 | 4.5 | 3.1 | 2.92 | 1.21 | 1.15 | 1.26 |
| 1 | 10.10162894 | 49 | 0 | 4.66 | 26 | 24 | 19 | 101 | 65.3 | 42.7 | 22.6 | 10.8 | 4.8 | 6 | 4.54 | 1.49 | 1.47 | 2.47 |
| 1 | 6.168021043 | 41 | 1 | 5.16 | 20 | 18 | 14 | 55 | 71.8 | 45.9 | 25.9 | 5.5 | 2.24 | 3.3 | 6.79 | 2.87 | 1.49 | 4.32 |
| 0 | 3.924420357 | 53 | 0 | 5.42 | 16 | 22 | 24 | 75 | 70.6 | 46.9 | 23.7 | 10.29 | 3.83 | 6.5 | 4.82 | 2.13 | 0.99 | 2.96 |
| 0 | 4.829822063 | 45 | 0 | 5.57 | 18 | 22 | 41 | 63 | 72.5 | 48.5 | 24 | 10.78 | 4.01 | 6.8 | 5.45 | 2.66 | 1.43 | 3.33 |
| 1 | 7.422484875 | 46 | 1 | 4.54 | 7 | 13 | 11 | 58 | 71.1 | 46.1 | 25 | 11.5 | 5 | 6.5 | 4.83 | 1.08 | 1.34 | 2.97 |
| 1 | 8.358252843 | 49 | 0 | 5.14 | 22 | 22 | 25 | 69 | 71.6 | 47.6 | 24 | 9.46 | 3.16 | 6.3 | 4.88 | 2.31 | 1.14 | 3.02 |
| 1 | 5.444484393 | 47 | 1 | 5.07 | 25 | 26 | 9 | 44 | 68.9 | 45.6 | 23.3 | 8.43 | 4.11 | 4.3 | 3.17 | 0.53 | 1.47 | 1.47 |
| 1 | 6.552419662 | 62 | 0 | 5.53 | 14 | 17 | 27 | 73 | 67.6 | 45.1 | 22.5 | 9.7 | 4 | 5.7 | 5.25 | 0.7 | 1.04 | 3.85 |
| 1 | 8.409457843 | 51 | 0 | 5.63 | 11 | 15 | 22 | 75 | 69.6 | 47.3 | 22.3 | 7.98 | 3.46 | 4.5 | 3.64 | 1.63 | 0.99 | 2.07 |
| 1 | 11.50849533 | 52 | 0 | 5.13 | 56 | 43 | 45 | 63 | 69.2 | 46.8 | 22.4 | 18.57 | 8.24 | 10.3 | 5.06 | 2.46 | 1.03 | 3.16 |
| 0 | 0.903087099 | 43 | 0 | 5.09 | 43 | 53 | 29 | 64 | 70.7 | 44.3 | 26.4 | 7.26 | 3.59 | 3.7 | 4.21 | 0.72 | 1.04 | 2.86 |
| 0 | 4.859070778 | 49 | 0 | 4.97 | 24 | 22 | 29 | 76 | 72.6 | 49 | 23.6 | 10.02 | 3.32 | 6.7 | 4.24 | 2.16 | 1.26 | 1.99 |
| 0 | 1.773333947 | 54 | 0 | 5.19 | 25 | 26 | 82 | 48 | 72.5 | 51.7 | 20.8 | 20.5 | 5.9 | 14.6 | 6.08 | 2.6 | 1.38 | 3.71 |
| 1 | 5.946569284 | 49 | 1 | 4.51 | 13 | 21 | 12 | 73 | 74.9 | 47.4 | 27.5 | 7.41 | 2.15 | 5.3 | 5.23 | 1.49 | 1.25 | 3.09 |
| 1 | 7.305841128 | 53 | 0 | 5.63 | 17 | 19 | 57 | 78 | 69.3 | 46.1 | 23.2 | 8.77 | 3.41 | 5.4 | 3.17 | 1.33 | 0.9 | 1.66 |
| 1 | 9.694521904 | 66 | 1 | 4.85 | 18 | 26 | 14 | 104 | 75.2 | 45.5 | 29.7 | 7.54 | 2.77 | 4.8 | 5.21 | 1.29 | 1.62 | 3.13 |
| 0 | 1.072087367 | 59 | 1 | 5.05 | 14 | 19 | 18 | 111 | 71 | 47.8 | 23.2 | 9.1 | 3 | 6.1 | 5.72 | 1.48 | 1.26 | 3.75 |
| 1 | 8.262966792 | 43 | 0 | 6.02 | 22 | 16 | 50 | 104 | 71.6 | 48.8 | 22.8 | 9.95 | 4.74 | 5.2 | 3.78 | 4.68 | 0.94 | 1.69 |
| 0 | 2.406471888 | 48 | 1 | 4.5 | 17 | 20 | 14 | 76 | 69.1 | 47.5 | 21.6 | 12.59 | 5.34 | 7.3 | 4.32 | 0.99 | 1.81 | 2.07 |
| 0 | 3.719142318 | 38 | 0 | 4.91 | 12 | 15 | 16 | 60 | 68.9 | 45.9 | 23 | 8.88 | 3.57 | 5.3 | 4.23 | 0.8 | 1.03 | 2.9 |
| 0 | 0.04636538 | 57 | 0 | 6.2 | 7 | 16 | 16 | 44 | 69.7 | 48.2 | 21.5 | 9.5 | 4.3 | 5.2 | 4.67 | 1.19 | 1.28 | 2.75 |
| 1 | 6.222039541 | 50 | 0 | 5 | 14 | 16 | 10 | 48 | 72.8 | 45.7 | 27.1 | 8.58 | 3.36 | 5.2 | 4.14 | 0.66 | 1.36 | 2.45 |
| 0 | 2.846282005 | 39 | 0 | 5.79 | 18 | 17 | 36 | 93 | 71.3 | 48.4 | 22.9 | 5.16 | 2.2 | 3 | 4.66 | 1.07 | 1.24 | 2.92 |
| 0 | 2.053413232 | 64 | 1 | 5.5 | 11 | 17 | 8 | 77 | 69 | 43.6 | 25.4 | 7.1 | 2.97 | 4.1 | 4.05 | 2.38 | 1.08 | 2.29 |
| 0 | 3.494201183 | 53 | 0 | 5.21 | 13 | 18 | 18 | 74 | 70.2 | 45.4 | 24.8 | 8.6 | 4.4 | 4.2 | 3.72 | 0.95 | 1.14 | 2.16 |
| 1 | 13.34377448 | 59 | 0 | 5.5 | 23 | 26 | 36 | 92 | 74.3 | 47 | 27.3 | 9.09 | 3.62 | 5.5 | 5.79 | 1.62 | 1.29 | 3.65 |
| 2 | 23.69099681 | 48 | 0 | 11.39 | 102 | 86 | 232 | 105 | 73.8 | 50 | 23.8 | 9.49 | 3.79 | 5.7 | 7.47 | 4.43 | 1.06 | 4.75 |
| 1 | 11.9295187 | 65 | 0 | 5.86 | 9 | 12 | 22 | 56 | 69.1 | 47.8 | 21.3 | 14.53 | 6.05 | 8.5 | 4.63 | 1.35 | 1.24 | 2.92 |
| 1 | 5.057947 | 59 | 0 | 4.93 | 26 | 21 | 17 | 65 | 69.1 | 44.8 | 24.3 | 8.56 | 4.87 | 3.7 | 2.95 | 1.89 | 0.89 | 1.34 |
| 2 | 21.7283802 | 64 | 0 | 7.35 | 22 | 23 | 38 | 72 | 69 | 48.4 | 20.6 | 6.85 | 3.14 | 3.7 | 5.93 | 3.75 | 1.13 | 3.54 |
| 1 | 9.475715637 | 63 | 0 | 7.85 | 36 | 25 | 20 | 84 | 71.5 | 48.5 | 23 | 13.35 | 6.15 | 7.2 | 3.48 | 0.76 | 1.42 | 1.65 |
| 1 | 8.492530346 | 56 | 0 | 5.85 | 44 | 29 | 73 | 71 | 66.7 | 43.8 | 22.9 | 9.39 | 4.87 | 4.5 | 4.48 | 1.76 | 1.54 | 2.31 |
| 2 | 23.04695765 | 50 | 0 | 6.51 | 36 | 32 | 32 | 80 | 78 | 49.7 | 28.3 | 13 | 3.8 | 9.2 | 4.89 | 2.25 | 0.99 | 3.18 |
| 0 | 4.190734386 | 58 | 1 | 5.28 | 12 | 17 | 18 | 96 | 71.6 | 44.2 | 27.4 | 16.5 | 6.8 | 9.7 | 4.49 | 1.02 | 1.36 | 2.67 |
| 0 | 4.503939986 | 78 | 1 | 5.66 | 13 | 17 | 17 | 65 | 77.1 | 48.5 | 28.6 | 6.5 | 3.54 | 3 | 3.53 | 1 | 1.29 | 1.79 |
| 1 | 6.317945798 | 58 | 1 | 5.07 | 18 | 19 | 13 | 60 | 79.1 | 48.7 | 30.4 | 5.3 | 2.6 | 2.7 | 5.71 | 1.17 | 1.73 | 3.49 |
| 1 | 9.154461702 | 47 | 0 | 4.52 | 25 | 20 | 33 | 68 | 73.5 | 48.3 | 25.2 | 27.07 | 8.67 | 18.4 | 5.47 | 1.74 | 1.18 | 3.68 |
| 0 | 2.559565306 | 40 | 0 | 5.47 | 29 | 19 | 30 | 69 | 82.3 | 49.2 | 33.1 | 20.16 | 7.83 | 12.3 | 3.31 | 0.97 | 0.88 | 2.05 |
| 1 | 9.221777916 | 57 | 0 | 4.75 | 18 | 23 | 19 | 54 | 71.1 | 46.6 | 24.5 | 12.8 | 5.7 | 7.1 | 4.79 | 0.67 | 1.65 | 2.78 |
| 2 | 18.07543818 | 45 | 0 | 5.07 | 25 | 21 | 25 | 71 | 70.8 | 49.9 | 20.9 | 15 | 7.2 | 7.8 | 3.8 | 1.07 | 0.9 | 2.52 |
| 1 | 5.512999733 | 47 | 0 | 4.94 | 19 | 18 | 17 | 56 | 70.3 | 49.5 | 20.8 | 5.26 | 2.59 | 2.7 | 3.84 | 0.9 | 0.98 | 2.45 |
| 1 | 13.38779354 | 33 | 0 | 4.91 | 52 | 24 | 38 | 67 | 76.2 | 50 | 26.2 | 10.86 | 3.77 | 7.1 | 4.09 | 5.83 | 0.72 | 1.95 |
| 1 | 6.226036231 | 42 | 1 | 5.79 | 20 | 15 | 13 | 50 | 70.4 | 45.4 | 25 | 6.7 | 3.2 | 3.5 | 4.98 | 1.65 | 1.12 | 3.02 |
| 1 | 5.249459743 | 57 | 0 | 5.6 | 42 | 26 | 24 | 86 | 71.8 | 49.3 | 22.5 | 18.6 | 9.2 | 9.4 | 2.97 | 0.69 | 1.23 | 1.4 |
| 1 | 5.340164185 | 61 | 0 | 4.97 | 34 | 25 | 32 | 100 | 72.2 | 48.6 | 23.6 | 10.3 | 3.7 | 6.6 | 5.82 | 1.42 | 1.37 | 3.87 |
| 1 | 9.712973436 | 50 | 0 | 5.22 | 22 | 16 | 17 | 87 | 71.6 | 49.1 | 22.5 | 13.31 | 5.49 | 7.8 | 2.48 | 1.71 | 0.79 | 1.08 |
| 1 | 9.510139783 | 61 | 1 | 4.82 | 26 | 19 | 29 | 101 | 69.7 | 48.6 | 21.1 | 11.34 | 3.67 | 7.7 | 5.54 | 1.47 | 1.53 | 3.45 |
| 1 | 8.827004115 | 61 | 0 | 5.26 | 13 | 14 | 13 | 68 | 63.6 | 43.1 | 20.5 | 10.6 | 5 | 5.6 | 3.71 | 0.51 | 1.12 | 2.22 |
| 0 | 3.346351504 | 58 | 0 | 4.46 | 23 | 26 | 18 | 77 | 71.8 | 50 | 21.8 | 39.52 | 14.44 | 25.1 | 2.91 | 1.07 | 0.93 | 1.61 |
| 2 | 18.62678909 | 44 | 0 | 5.19 | 37 | 27 | 138 | 94 | 69.5 | 46.9 | 22.6 | 12.4 | 5.4 | 7 | 4.05 | 3.5 | 0.99 | 2.35 |
| 1 | 7.50812912 | 50 | 1 | 4.76 | 14 | 15 | 11 | 67 | 61.8 | 39.6 | 22.2 | 12.4 | 5.03 | 7.4 | 4.29 | 1.06 | 1.23 | 2.45 |
| 1 | 9.433544318 | 59 | 0 | 5.11 | 15 | 16 | 28 | 71 | 68.5 | 43.6 | 24.9 | 9.8 | 4.29 | 5.5 | 4.55 | 0.82 | 1.51 | 2.51 |
| 1 | 9.087505658 | 59 | 0 | 8.1 | 15 | 21 | 27 | 69 | 65.5 | 45.4 | 20.1 | 14.21 | 5.85 | 8.4 | 4.18 | 4.38 | 0.84 | 1.79 |
| 0 | 3.84762303 | 60 | 0 | 6.9 | 22 | 30 | 21 | 83 | 67.6 | 46.6 | 21 | 6.7 | 3.2 | 3.5 | 4.44 | 0.84 | 1.71 | 2.41 |
| 1 | 5.375981967 | 65 | 0 | 6.82 | 22 | 23 | 34 | 79 | 73.8 | 46.9 | 26.9 | 16.52 | 4.16 | 12.4 | 6.57 | 1.88 | 1.17 | 4.69 |
| 1 | 8.047365824 | 58 | 0 | 6.02 | 20 | 21 | 9 | 47 | 67.1 | 47.1 | 20 | 11.07 | 4.55 | 6.5 | 4.46 | 0.7 | 1.74 | 2.39 |
| 2 | 14.64449628 | 59 | 0 | 5.91 | 22 | 18 | 21 | 78 | 74.1 | 49.5 | 24.6 | 23.67 | 7.1 | 16.6 | 4.77 | 3.67 | 0.9 | 2.74 |
| 1 | 5.49162511 | 59 | 0 | 5.16 | 16 | 22 | 19 | 68 | 70.3 | 46.9 | 23.4 | 16.85 | 6.72 | 10.1 | 3.61 | 1.79 | 0.86 | 2.07 |
| 0 | 2.271998405 | 45 | 1 | 4.43 | 4 | 13 | 12 | 46 | 71.6 | 46.6 | 25 | 6.34 | 2.48 | 3.9 | 3.69 | 1.04 | 1.04 | 2.21 |
| 0 | 3.925493638 | 59 | 1 | 5.02 | 33 | 31 | 26 | 40 | 73.6 | 46.1 | 27.5 | 13.36 | 5.59 | 7.8 | 3.77 | 1.46 | 1.7 | 1.56 |
| 1 | 13.84265709 | 57 | 0 | 5.63 | 46 | 31 | 34 | 63 | 73.2 | 53.1 | 20.1 | 18.74 | 5.04 | 13.7 | 6.01 | 4.29 | 1.33 | 3.18 |
| 0 | 3.211136381 | 83 | 1 | 4.98 | 6 | 16 | 12 | 56 | 65.5 | 42 | 23.5 | 5.15 | 2.9 | 2.3 | 5.5 | 1.21 | 1.62 | 3.28 |
| 0 | 4.767298698 | 86 | 0 | 6.16 | 17 | 18 | 22 | 119 | 76 | 43 | 33 | 9 | 3.9 | 5.1 | 3.55 | 1.57 | 1.14 | 1.8 |
| 1 | 10.9380366 | 46 | 0 | 5.3 | 18 | 20 | 21 | 68 | 70.5 | 49.6 | 20.9 | 15 | 5.2 | 9.8 | 5.42 | 3.34 | 1 | 3.42 |
| 1 | 9.490380605 | 59 | 0 | 5.81 | 25 | 22 | 27 | 93 | 77.4 | 54.7 | 22.7 | 3.83 | 1.52 | 2.3 | 4.91 | 1.38 | 0.78 | 3.27 |
| 2 | 20.13864899 | 37 | 0 | 6.18 | 117 | 57 | 78 | 112 | 77.2 | 56.1 | 21.1 | 20.55 | 7.51 | 13 | 5.43 | 4.41 | 0.95 | 3.03 |
| 1 | 7.11248366 | 54 | 1 | 6.68 | 35 | 29 | 53 | 91 | 71.3 | 48.3 | 23 | 8.24 | 2.52 | 5.7 | 4.83 | 0.77 | 1.64 | 2.73 |
| 1 | 13.88020134 | 58 | 0 | 5.68 | 36 | 27 | 42 | 105 | 74.2 | 46.3 | 27.9 | 15.41 | 7.33 | 8.1 | 3.92 | 1.53 | 1.08 | 2.31 |
| 0 | 4.853276253 | 52 | 1 | 4.91 | 10 | 15 | 13 | 22 | 76.1 | 46.2 | 29.9 | 12.1 | 5.6 | 6.5 | 5.5 | 0.85 | 1.69 | 3.48 |
| 1 | 5.910669804 | 42 | 1 | 4.73 | 13 | 13 | 17 | 57 | 69.1 | 49.3 | 19.8 | 10.9 | 6.2 | 4.7 | 3.2 | 0.77 | 1.7 | 1.14 |
| 0 | 2.65220964 | 38 | 1 | 5.19 | 7 | 11 | 8 | 47 | 70 | 46.9 | 23.1 | 5.23 | 3.2 | 2 | 4.2 | 0.59 | 1.48 | 2.59 |
| 1 | 7.929882367 | 58 | 0 | 5.38 | 21 | 39 | 39 | 89 | 66.9 | 46.2 | 20.7 | 6.6 | 3.4 | 3.2 | 4.83 | 0.86 | 1.25 | 2.99 |
| 0 | 2.998244842 | 71 | 1 | 5.11 | 8 | 18 | 13 | 66 | 79.3 | 49.1 | 30.2 | 7.91 | 3.77 | 4.1 | 5.27 | 0.78 | 1.48 | 3.37 |
| 2 | 20.49971517 | 69 | 0 | 5.9 | 37 | 36 | 68 | 106 | 78.9 | 51.2 | 27.7 | 11.48 | 3.45 | 8 | 4.85 | 3.16 | 0.97 | 2.7 |
| 3 | 28.33428605 | 49 | 0 | 4.63 | 16 | 21 | 15 | 64 | 73.1 | 46 | 27.1 | 15.3 | 5.1 | 10.2 | 6.93 | 0.8 | 2.08 | 4.58 |
| 3 | 29.31374518 | 56 | 0 | 6.33 | 12 | 13 | 17 | 78 | 44.7 | 29.4 | 15.3 | 5.4 | 2.4 | 3 | 7.43 | 1.83 | 2.28 | 4.65 |
| 3 | 28.23402977 | 46 | 1 | 5.74 | 219 | 125 | 40 | 99 | 81 | 49.5 | 31.5 | 13.76 | 5.02 | 8.7 | 6.49 | 1.56 | 1.35 | 4.4 |
| 3 | 31.10363515 | 45 | 0 | 7.09 | 45 | 36 | 62 | 72 | 81.8 | 53.3 | 28.5 | 12.75 | 2.13 | 10.6 | 4.51 | 1.37 | 1.29 | 2.83 |
| 3 | 29.8385404 | 33 | 0 | 6.19 | 26 | 25 | 8 | 87 | 77.7 | 48.9 | 28.8 | 14.9 | 6.2 | 8.7 | 3.62 | 1.39 | 1.14 | 2.02 |
| 3 | 31.985569 | 48 | 1 | 6.2 | 36 | 29 | 26 | 68 | 77.8 | 48.4 | 29.4 | 7.6 | 3.5 | 4.1 | 4.99 | 1.7 | 1.53 | 2.89 |
| 3 | 34.59740321 | 28 | 0 | 4.79 | 111 | 42 | 110 | 75 | 74.4 | 49.2 | 25.2 | 13.6 | 5.2 | 9.4 | 5.38 | 3.75 | 0.85 | 3.14 |
| 2 | 20.76018079 | 35 | 0 | 6.33 | 46 | 26 | 38 | 80 | 78.2 | 50.3 | 27.9 | 4.74 | 2.73 | 2 | 5.40 | 1.56 | 1.09 | 3.92 |
| 3 | 40.28986231 | 28 | 0 | 5.06 | 23 | 20 | 15 | 56 | 72 | 49.5 | 22.5 | 13.5 | 4.6 | 8.9 | 4.04 | 1.05 | 1.26 | 2.58 |
| 3 | 33.9541556 | 31 | 0 | 5.49 | 37 | 26 | 40 | 148 | 47.7 | 30.3 | 17.4 | 3.6 | 1.5 | 2.1 | 7.49 | 2.2 | 2.63 | 4.56 |
| 3 | 35.12604396 | 35 | 1 | 6.82 | 33 | 23 | 60 | 71 | 76.9 | 45.8 | 31.1 | 4.4 | 2.6 | 1.8 | 5.09 | 1.97 | 1.03 | 3.65 |
| 3 | 36.12223519 | 40 | 1 | 5.98 | 40 | 26 | 31 | 62 | 74 | 44.1 | 29.9 | 7.1 | 3.78 | 3.3 | 2.87 | 1.35 | 1.49 | 1.09 |
| 3 | 30.30571175 | 40 | 1 | 5.01 | 57 | 34 | 26 | 49 | 72 | 46.4 | 25.6 | 6.08 | 2.51 | 3.6 | 5.82 | 1.96 | 1.35 | 3.75 |
| 3 | 31.65054576 | 32 | 1 | 5.75 | 30 | 25 | 30 | 103 | 70.5 | 42.8 | 27.7 | 8.8 | 3.0 | 5.8 | 3.71 | 0.81 | 1.46 | 2.15 |
| 3 | 29.00256666 | 36 | 0 | 5.2 | 18 | 18 | 14 | 90 | 72.7 | 46.2 | 26.5 | 19.83 | 6.19 | 13.6 | 5.98 | 0.83 | 1.59 | 3.76 |
| 3 | 35.77504603 | 36 | 0 | 5.04 | 72 | 33 | 46 | 82 | 76.9 | 49.5 | 27.4 | 8.31 | 3.83 | 4.5 | 7.17 | 1.85 | 1.31 | 5.16 |
| 3 | 38.91990662 | 28 | 0 | 4.97 | 87 | 33 | 68 | 82 | 74.1 | 49.9 | 24.2 | 10.23 | 4.25 | 6 | 4.5 | 6.43 | 0.7 | 2.26 |
| 2 | 27.61690331 | 35 | 0 | 4.99 | 70 | 32 | 41 | 62 | 70.6 | 48.2 | 22.4 | 9.86 | 4.28 | 5.6 | 4.78 | 3.16 | 0.88 | 3.0 |
| 3 | 32.93161265 | 33 | 0 | 4.8 | 56 | 24 | 49 | 67 | 80.6 | 52.6 | 28 | 10 | 3.9 | 6.1 | 5.18 | 2.77 | 1.10 | 3.14 |
| 3 | 30.55215136 | 36 | 0 | 5.43 | 34 | 24 | 55 | 75 | 74.7 | 50.5 | 24.2 | 8.35 | 2.88 | 5.5 | 4.71 | 4.09 | 0.97 | 2.36 |
| 3 | 35.63621394 | 38 | 1 | 4.6 | 59 | 46 | 44 | 62 | 65.6 | 41.1 | 24.5 | 5.55 | 2.46 | 3.1 | 6.05 | 3.21 | 1.17 | 3.33 |
| 3 | 38.00460943 | 31 | 1 | 11.51 | 69 | 39 | 99 | 99 | 76.4 | 47.8 | 28.6 | 11.31 | 5.47 | 5.8 | 5.18 | 2.46 | 0.87 | 3.69 |
| 3 | 28.09906832 | 33 | 0 | 6.28 | 53 | 23 | 32 | 98 | 68.7 | 50.4 | 18.3 | 13.69 | 4.78 | 8.9 | 4.62 | 1.64 | 0.98 | 2.55 |
| 3 | 31.95376396 | 31 | 0 | 4.94 | 39 | 34 | 42 | 94 | 74.9 | 51.1 | 23.8 | 12.1 | 5.6 | 6.5 | 4.8 | 1.55 | 1.05 | 2.92 |
| 3 | 35.36386172 | 52 | 1 | 5.55 | 13 | 15 | 16 | 93 | 73.7 | 46.6 | 27.1 | 5.4 | 2.45 | 3 | 4.18 | 1.54 | 1.04 | 2.59 |
| 3 | 36.1242218 | 32 | 0 | 3.74 | 7 | 13 | 11 | 77 | 63.6 | 36.4 | 27.2 | 6.77 | 1.52 | 5.3 | 2.23 | 0.91 | 0.91 | 0.94 |
| 3 | 33.23260244 | 34 | 0 | 4.65 | 61 | 38 | 54 | 65 | 76.9 | 53.5 | 23.4 | 17.4 | 4.8 | 12.6 | 6.83 | 2.72 | 1.12 | 4.5 |
| 3 | 34.05109596 | 25 | 0 | 4.43 | 64 | 30 | 66 | 80 | 71.3 | 51.4 | 19.9 | 11.61 | 5 | 6.6 | 4.38 | 1.56 | 0.92 | 3.19 |
| 3 | 28.34622002 | 49 | 0 | 7.75 | 79 | 50 | 214 | 80 | 79.5 | 51.9 | 27.6 | 23.1 | 7.8 | 15.3 | 6.43 | 5.58 | 1.23 | 3.67 |
| 2 | 27.590566 | 45 | 1 | 5.52 | 26 | 22 | 17 | 87 | 75.4 | 47.3 | 18.1 | 25.47 | 8.46 | 16.7 | 4.04 | 1.01 | 1 | 2.8 |
| 3 | 32.63002777 | 31 | 0 | 5.59 | 51 | 24 | 34 | 67 | 74.9 | 52.9 | 22 | 17.48 | 6.29 | 10.6 | 5.02 | 2.16 | 1.07 | 3.24 |
| 3 | 41.48886236 | 22 | 0 | 6.39 | 202 | 86 | 53 | 90 | 73.2 | 51.5 | 21.7 | 18.68 | 7.76 | 10.9 | 5.66 | 1.55 | 0.94 | 4.15 |
| 3 | 29.62797101 | 32 | 0 | 5.18 | 33 | 22 | 52 | 87 | 71.9 | 43.8 | 28.1 | 8.14 | 3.42 | 4.7 | 5.08 | 8.29 | 0.73 | 2.03 |
| 3 | 35.82895279 | 33 | 0 | 5.41 | 41 | 18 | 34 | 86 | 76 | 52.2 | 23.8 | 13.98 | 5.88 | 8.1 | 4.4 | 1.12 | 1.03 | 2.93 |
| 2 | 25.73415248 | 34 | 0 | 4.81 | 70 | 29 | 25 | 54 | 75 | 53.2 | 21.8 | 13.54 | 5.22 | 8.3 | 5.30 | 2.37 | 0.89 | 3.65 |
| 2 | 26.24021912 | 33 | 0 | 4.41 | 43 | 29 | 22 | 62 | 72.4 | 49.6 | 22.8 | 8.38 | 3.94 | 4.4 | 3.26 | 1.82 | 0.81 | 2.11 |
| 3 | 36.74491628 | 33 | 0 | 4.63 | 68 | 27 | 99 | 91 | 77 | 50.8 | 26.2 | 21.05 | 7.68 | 13.4 | 4.44 | 2.4 | 1 | 2.75 |
| 3 | 35.340964 | 37 | 0 | 5.11 | 112 | 52 | 115 | 79 | 80.2 | 54.3 | 25.9 | 12.6 | 5.3 | 7.3 | 6.29 | 2.48 | 0.91 | 4.39 |
| 3 | 39.86642583 | 46 | 0 | 4.85 | 99 | 46 | 50 | 55 | 68.5 | 48.1 | 20.4 | 12.37 | 6.14 | 6.2 | 5.92 | 3.7 | 0.95 | 3.44 |
| 3 | 33.53623962 | 29 | 0 | 9.21 | 77 | 31 | 37 | 87 | 67.5 | 45.1 | 22.4 | 12.34 | 4.51 | 7.8 | 4.88 | 1.58 | 0.9 | 3.23 |
| 3 | 33.97972933 | 36 | 0 | 6.66 | 125 | 67 | 289 | 96 | 72.6 | 50.1 | 22.5 | 8.57 | 3.54 | 5.0 | 6.75 | 5.23 | 1.16 | 4.02 |
| 3 | 33.70912806 | 51 | 0 | 6.59 | 40 | 27 | 66 | 83 | 74.1 | 45.7 | 28.4 | 7.61 | 3.90 | 3.7 | 6.21 | 7.46 | 0.84 | 2.57 |
| 3 | 31.51135254 | 41 | 1 | 5.79 | 105 | 81 | 24 | 65 | 66.8 | 49.1 | 17.7 | 11 | 4.4 | 6.6 | 5.54 | 4.5 | 1 | 3.12 |
| 3 | 39.35691833 | 41 | 0 | 5.14 | 30 | 30 | 45 | 87 | 73.4 | 50.8 | 22.6 | 13.1 | 4.5 | 8.6 | 4.34 | 3.41 | 0.96 | 2.31 |
| 3 | 34.41584714 | 20 | 0 | 4.67 | 52 | 26 | 44 | 65 | 71.6 | 48.5 | 23.1 | 8.08 | 3.20 | 4.9 | 5.58 | 2.09 | 1.10 | 3.34 |
| 2 | 27.7703406 | 34 | 0 | 5.15 | 19 | 21 | 28 | 69 | 77.4 | 46.9 | 30.5 | 8.5 | 3.5 | 5 | 4.76 | 1.38 | 0.96 | 3.37 |
| 3 | 37.62236532 | 38 | 0 | 5.17 | 104 | 49 | 46 | 95 | 80 | 57.6 | 22.4 | 8.7 | 3.47 | 5.2 | 5.63 | 1.55 | 1.21 | 3.72 |
| 2 | 19.2716643 | 34 | 0 | 4.69 | 43 | 25 | 34 | 105 | 70.4 | 47.2 | 23.2 | 7.13 | 2.64 | 4.5 | 6.63 | 1.65 | 1.18 | 4.49 |
| 3 | 34.36236445 | 23 | 0 | 4.74 | 97 | 40 | 51 | 69 | 74 | 49.2 | 24.8 | 14 | 5 | 9 | 4.94 | 2.03 | 1.04 | 3.33 |
| 2 | 26.64935684 | 51 | 1 | 6.31 | 36 | 23 | 22 | 78 | 80.4 | 46.7 | 33.7 | 7.88 | 2.91 | 5 | 4.37 | 2.46 | 1.05 | 2.35 |
| 3 | 28.20843887 | 45 | 0 | 7.87 | 64 | 36 | 54 | 57 | 74.2 | 46.1 | 28.1 | 8.69 | 5.02 | 3.7 | 5.24 | 2.21 | 1.16 | 3.01 |
| 3 | 38.82820129 | 33 | 0 | 5.67 | 20 | 18 | 16 | 54 | 74.2 | 49.2 | 25 | 18.1 | 7.6 | 10.5 | 5.47 | 1.15 | 1.39 | 4.06 |
| 2 | 23.01475461 | 31 | 0 | 4.29 | 71 | 34 | 36 | 71 | 69.7 | 49.5 | 20.2 | 10.89 | 4.53 | 6.4 | 4.82 | 1.66 | 1.11 | 3.34 |
| 3 | 31.62513097 | 44 | 1 | 5.82 | 18 | 20 | 15 | 66 | 70.7 | 43.6 | 27.1 | 9.95 | 3.69 | 6.3 | 3.94 | 3.52 | 0.88 | 2.29 |
| 2 | 25.17696253 | 46 | 0 | 5.8 | 44 | 30 | 34 | 55 | 71.5 | 49.6 | 21.9 | 7.78 | 2.73 | 5.1 | 5.94 | 2.27 | 1.68 | 3.58 |
| 3 | 32.96958542 | 43 | 0 | 16.88 | 26 | 20 | 51 | 57 | 70.8 | 48 | 22.8 | 16.15 | 6.65 | 9.5 | 5.52 | 2.87 | 0.97 | 3.10 |
| 3 | 32.3782355 | 45 | 1 | 4.86 | 57 | 33 | 23 | 89 | 70 | 42.6 | 27.4 | 9.11 | 4.30 | 4.8 | 5.56 | 1.31 | 1.24 | 4 |
| 2 | 21.09722837 | 44 | 0 | 8.23 | 31 | 26 | 51 | 155 | 68.8 | 49.6 | 19.2 | 13.74 | 6.18 | 7.6 | 5.19 | 2.04 | 1.15 | 3.33 |
| 3 | 31.2699337 | 57 | 0 | 4.59 | 42 | 31 | 63 | 77 | 74.8 | 49.7 | 25.1 | 8.63 | 4.03 | 4.6 | 6.57 | 3.51 | 1.29 | 4.22 |
| 3 | 34.20841853 | 42 | 1 | 6.59 | 48 | 30 | 42 | 81 | 77.3 | 47 | 30.3 | 5.11 | 2.62 | 2.5 | 5.93 | 2.03 | 1.47 | 3.64 |
| 2 | 21.57284609 | 27 | 0 | 5 | 22 | 19 | 26 | 92 | 74.6 | 48.8 | 25.8 | 9.1 | 3.7 | 5.4 | 4.34 | 3.99 | 0.64 | 2.14 |
| 2 | 19.63019191 | 60 | 0 | 9.4 | 36 | 38 | 31 | 87 | 72.9 | 49 | 23.9 | 21.13 | 3.85 | 17.3 | 4.84 | 1.61 | 1.31 | 2.73 |
| 2 | 25.20562077 | 33 | 0 | 5.48 | 104 | 61 | 53 | 82 | 75.6 | 51.1 | 24.5 | 6.7 | 3.42 | 3.3 | 5.67 | 3.4 | 1.26 | 2.98 |
| 2 | 18.06442006 | 33 | 0 | 5.03 | 23 | 17 | 27 | 77 | 74.2 | 49.8 | 24.4 | 15.56 | 5.55 | 10 | 4.58 | 1.21 | 0.91 | 2.76 |
| 2 | 27.87336604 | 34 | 0 | 5.89 | 28 | 33 | 37 | 81 | 77.1 | 52.1 | 25 | 6.6 | 2.8 | 3.8 | 5.66 | 1.6 | 0.96 | 4.18 |
| 2 | 25.79714648 | 33 | 0 | 10.5 | 177 | 73 | 27 | 62 | 70.1 | 44.5 | 25.9 | 17.7 | 7.4 | 10.3 | 3.75 | 1.2 | 1.1 | 2.26 |
| 3 | 40.71565882 | 31 | 1 | 6.49 | 34 | 23 | 31 | 78 | 66.5 | 42.3 | 24.2 | 4.94 | 2.29 | 2.7 | 4.65 | 1.14 | 1.05 | 2.75 |
| 3 | 28.67969386 | 39 | 0 | 5.43 | 54 | 42 | 55 | 82 | 71.9 | 48.6 | 23.3 | 17.81 | 5.4 | 12.4 | 9.92 | 15.22 | 0.6 | 1.02 |
| 3 | 34.18495242 | 46 | 0 | 7.85 | 44 | 40 | 65 | 80 | 77.5 | 47.2 | 30.3 | 24.9 | 7.8 | 17.1 | 6.02 | 2.20 | 1.18 | 4.25 |
| 2 | 26.35419401 | 32 | 0 | 4.88 | 96 | 57 | 30 | 65 | 76 | 53.7 | 22.3 | 14.34 | 6.12 | 8.2 | 5.4 | 1.51 | 1.11 | 3.39 |
| 3 | 33.53734779 | 39 | 0 | 7.66 | 35 | 24 | 27 | 98 | 74.5 | 46.8 | 27.2 | 9.14 | 4.27 | 4.9 | 5 | 1.19 | 1.18 | 2.91 |
| 2 | 27.66401164 | 40 | 0 | 5.05 | 56 | 35 | 48 | 85 | 67.8 | 43.4 | 24.4 | 8.1 | 3.2 | 4.9 | 4.5 | 2.33 | 1.16 | 2.8 |
| 3 | 29.56408755 | 53 | 0 | 7.02 | 39 | 23 | 314 | 73 | 68.2 | 47.1 | 21.1 | 10.61 | 4.33 | 6.3 | 5.46 | 4.57 | 0.93 | 2.45 |
| 2 | 25.52331479 | 44 | 0 | 6.04 | 21 | 29 | 23 | 67 | 71.3 | 46.6 | 24.7 | 8.86 | 3.73 | 5.1 | 5.28 | 2.11 | 0.9 | 3.49 |
| 2 | 22.15525627 | 42 | 0 | 4.66 | 46 | 24 | 43 | 79 | 73.1 | 47.4 | 25.7 | 11.64 | 4.48 | 7.2 | 4.6 | 2.16 | 0.89 | 2.98 |
| 3 | 37.619325 | 33 | 0 | 4.51 | 126 | 50 | 81 | 71 | 73.7 | 53 | 20.7 | 10.08 | 3.8 | 6.3 | 6.02 | 2.56 | 0.87 | 3.8 |
| 3 | 42.95984523 | 31 | 0 | 5.68 | 98 | 41 | 82 | 68 | 79.7 | 50.8 | 28.9 | 11.6 | 6 | 5.6 | 4.49 | 2.01 | 1.2 | 2.52 |
| 3 | 30.68830554 | 35 | 1 | 4.34 | 20 | 17 | 22 | 103 | 75.7 | 49.7 | 26 | 10.28 | 3.38 | 6.9 | 6.8 | 1.8 | 1.66 | 4.47 |
| 2 | 26.96688906 | 33 | 0 | 5.24 | 34 | 25 | 24 | 49 | 73.5 | 51 | 22.5 | 22.4 | 8.73 | 13.7 | 3.85 | 0.73 | 1.23 | 2.25 |
| 2 | 23.05158488 | 39 | 0 | 4.54 | 30 | 24 | 41 | 60 | 71.7 | 48 | 23.7 | 15.69 | 4.98 | 10.7 | 6.03 | 1.64 | 1.03 | 4.01 |
| 3 | 29.97748439 | 57 | 0 | 5.81 | 48 | 75 | 189 | 81 | 72.2 | 49.4 | 22.8 | 17.2 | 8.1 | 9.1 | 6.61 | 1.38 | 1.89 | 4.62 |
| 2 | 26.86263847 | 23 | 0 | 5.42 | 31 | 25 | 44 | 64 | 68.5 | 45.5 | 23 | 10.03 | 3.67 | 6.4 | 5.01 | 2.71 | 0.96 | 3.44 |
| 3 | 28.13651403 | 54 | 0 | 5.8 | 25 | 29 | 29 | 101 | 76.3 | 46.3 | 30 | 5.08 | 1.1 | 4.1 | 5.74 | 1.59 | 1.16 | 4.07 |
| 2 | 26.43804423 | 48 | 0 | 5.09 | 25 | 21 | 25 | 60 | 73 | 46.9 | 26.1 | 7.67 | 3.3 | 4.4 | 4.84 | 6.31 | 0.83 | 1.74 |
| 2 | 22.48863538 | 36 | 0 | 4.4 | 46 | 26 | 37 | 89 | 72.7 | 44.9 | 27.8 | 15.52 | 5.4 | 10.1 | 5.65 | 2.51 | 1.07 | 3.47 |
| 2 | 23.96949005 | 32 | 0 | 4.76 | 35 | 30 | 72 | 74 | 70.7 | 48.9 | 21.8 | 13.73 | 4.47 | 9.3 | 5.35 | 2.94 | 1.01 | 3.19 |
| 2 | 16.39729452 | 29 | 0 | 4.39 | 52 | 30 | 36 | 101 | 75.6 | 49.8 | 25.8 | 12.8 | 2.94 | 9.9 | 4.93 | 2.14 | 0.93 | 3.04 |
| 3 | 37.88605118 | 51 | 1 | 6.35 | 61 | 45 | 32 | 90 | 66.7 | 42 | 24.7 | 5.9 | 2.57 | 3.3 | 4.48 | 2.38 | 0.8 | 2.73 |
| 2 | 25.73213196 | 31 | 0 | 4.44 | 40 | 23 | 47 | 77 | 71.1 | 49.1 | 22 | 10.16 | 2.92 | 7.2 | 6.56 | 3.81 | 1.07 | 4.33 |
| 2 | 20.70897897 | 34 | 0 | 5.21 | 26 | 16 | 21 | 94 | 71.5 | 50.2 | 21.3 | 7.47 | 2.67 | 4.8 | 4.84 | 2.61 | 1.01 | 2.93 |
| 2 | 26.90286255 | 47 | 0 | 5.78 | 49 | 33 | 55 | 118 | 66.3 | 50.6 | 15.7 | 17.12 | 5.71 | 11.4 | 3.58 | 3.19 | 0.91 | 1.42 |
| 2 | 25.96799914 | 65 | 0 | 11.95 | 97 | 84 | 95 | 74 | 75.2 | 45.5 | 29.7 | 12.47 | 5.74 | 6.7 | 4.15 | 0.92 | 1.07 | 2.68 |
| 3 | 29.05785815 | 40 | 0 | 5.39 | 41 | 24 | 34 | 53 | 73.3 | 48.1 | 25.2 | 9.8 | 3.67 | 6.4 | 5.03 | 1.26 | 1.5 | 3.05 |
| 3 | 33.05910238 | 42 | 1 | 6.69 | 50 | 29 | 32 | 102 | 77.4 | 44.9 | 32.5 | 12.29 | 3.69 | 8.6 | 6.89 | 4.29 | 1.09 | 3.08 |
| 3 | 31.00682068 | 40 | 0 | 6.24 | 76 | 35 | 61 | 83 | 68.9 | 43.6 | 25.3 | 18.2 | 4.99 | 13.2 | 5.01 | 1.4 | 1 | 3.47 |
| 2 | 27.36107254 | 35 | 0 | 5.3 | 50 | 31 | 80 | 77 | 73.8 | 49 | 24.8 | 8.1 | 3.07 | 5 | 4.86 | 2.01 | 0.96 | 3.29 |
| 2 | 20.88791148 | 39 | 0 | 8.72 | 64 | 48 | 25 | 76 | 75.5 | 47.2 | 28.3 | 25.9 | 11.2 | 14.7 | 3.39 | 0.98 | 0.95 | 2.07 |
| 2 | 26.76054192 | 33 | 0 | 4.81 | 49 | 24 | 55 | 57 | 75.8 | 50.1 | 25.7 | 16.2 | 6.3 | 9.9 | 4.85 | 1.57 | 1.27 | 3.07 |
| 2 | 27.97030258 | 37 | 0 | 4.82 | 53 | 33 | 44 | 79 | 71.9 | 46.6 | 25.3 | 7.7 | 2.4 | 5.3 | 5.09 | 1.78 | 1.13 | 3.4 |
| 3 | 30.17985598 | 44 | 0 | 7.2 | 74 | 35 | 45 | 51 | 79 | 47.2 | 31.8 | 12.8 | 4.7 | 8.1 | 5.23 | 4.36 | 1.07 | 2.77 |
| 3 | 30.27204259 | 37 | 0 | 5.79 | 63 | 34 | 54 | 45 | 84.7 | 51.1 | 33.6 | 25.3 | 9.6 | 15.7 | 5.2 | 2.23 | 1.2 | 3.28 |
| 3 | 32.19312223 | 32 | 0 | 5.26 | 82 | 56 | 68 | 89 | 71.5 | 49 | 22.5 | 14.16 | 5.14 | 9 | 5.71 | 2.09 | 1.25 | 3.42 |
| 3 | 28.40746752 | 34 | 1 | 6.62 | 30 | 26 | 15 | 68 | 68 | 46 | 22 | 5.88 | 2.96 | 2.9 | 6.03 | 2.47 | 1.43 | 3.67 |
| 3 | 29.58721542 | 41 | 0 | 12.52 | 57 | 40 | 104 | 133 | 73.3 | 46.8 | 26.5 | 10.28 | 4.77 | 5.5 | 6.23 | 3.10 | 1.21 | 3.76 |
| 2 | 24.05233002 | 28 | 0 | 5.54 | 51 | 29 | 63 | 73 | 78.8 | 55.4 | 23.3 | 10.32 | 3.47 | 6.8 | 5.81 | 2.18 | 0.98 | 3.37 |
| 3 | 31.70922152 | 53 | 0 | 8.3 | 31 | 222 | 102 | 75 | 72.2 | 48.8 | 23.4 | 16.44 | 5.56 | 10.9 | 5.7 | 2.06 | 1.14 | 3.67 |
| 2 | 25.61070633 | 35 | 0 | 4.67 | 66 | 94 | 597 | 100 | 72.5 | 47.8 | 24.7 | 12.7 | 7.1 | 5.6 | 6.51 | 0.98 | 1.09 | 5.1 |
| 2 | 23.60297585 | 42 | 0 | 6.07 | 15 | 10 | 52 | 92 | 71.4 | 43.3 | 28.1 | 15.37 | 11.15 | 4.2 | 8.84 | 33.53 | 0.51 | 1.22 |
| 2 | 24.52884865 | 46 | 0 | 6.25 | 31 | 22 | 47 | 83 | 71.9 | 50.2 | 21.7 | 9.67 | 4.49 | 5.2 | 5.13 | 1.94 | 1.03 | 3.33 |
| 2 | 25.15083059 | 29 | 0 | 7.45 | 71 | 27 | 102 | 47 | 78 | 49 | 29 | 12 | 4.3 | 7.7 | 5.81 | 4.68 | 0.86 | 3.75 |
| 2 | 26.30738831 | 46 | 0 | 5.27 | 18 | 20 | 29 | 77 | 71.4 | 45.8 | 25.6 | 5.68 | 2.08 | 3.6 | 4.83 | 5.65 | 0.53 | 2.55 |
| 3 | 37.42296346 | 36 | 0 | 5.86 | 112 | 54 | 60 | 78 | 75.9 | 50.1 | 25.8 | 11.9 | 4.38 | 7.5 | 6.85 | 4.5 | 0.91 | 4.25 |
| 3 | 30.83383878 | 31 | 0 | 4.63 | 104 | 51 | 78 | 79 | 72 | 50.6 | 21.4 | 13.22 | 4.13 | 9.1 | 4.53 | 1.37 | 0.98 | 2.77 |
| 3 | 35.15335719 | 52 | 0 | 5.88 | 57 | 52 | 41 | 89 | 71.4 | 43.4 | 28 | 4.73 | 2.51 | 2.2 | 4.82 | 2.42 | 0.8 | 2.73 |
| 2 | 19.57148616 | 31 | 0 | 5.88 | 86 | 37 | 36 | 78 | 70.1 | 48.6 | 21.5 | 15.35 | 4.88 | 10.5 | 6.18 | 2.31 | 1.10 | 4.07 |
| 3 | 29.72709211 | 30 | 0 | 5.63 | 52 | 26 | 55 | 69 | 72.5 | 48.9 | 23.6 | 13.36 | 3.93 | 9.4 | 6.07 | 2.87 | 0.96 | 3.73 |
| 3 | 35.34616089 | 40 | 0 | 5.29 | 60 | 46 | 48 | 104 | 71.1 | 48 | 23.1 | 7.4 | 3.7 | 3.7 | 4.62 | 1.42 | 1.05 | 3.09 |
| 2 | 25.32974021 | 35 | 0 | 5.42 | 83 | 31 | 119 | 85 | 70.4 | 47 | 21.4 | 8.28 | 3.5 | 4.8 | 5.32 | 1.47 | 1.25 | 3.42 |
| 3 | 31.54695956 | 45 | 0 | 5.32 | 120 | 77 | 84 | 111 | 74.4 | 49.8 | 24.6 | 8.9 | 3.0 | 5.9 | 6.39 | 0.98 | 1.82 | 4.37 |
| 2 | 21.47920863 | 46 | 0 | 5.12 | 30 | 22 | 34 | 91 | 77 | 50.4 | 26.6 | 11.7 | 4.4 | 7.3 | 6.06 | 1.35 | 1.31 | 4.43 |
| 3 | 40.64473216 | 40 | 0 | 4.3 | 52 | 29 | 54 | 80 | 75.4 | 49.6 | 25.8 | 13.78 | 5.81 | 8 | 4.55 | 1.56 | 1.32 | 2.77 |
| 3 | 29.98101934 | 54 | 0 | 5.41 | 45 | 27 | 74 | 87 | 74.8 | 47.2 | 27.6 | 9.04 | 3.61 | 5.4 | 7.34 | 3.09 | 1.39 | 4.84 |
| 3 | 42.89150111 | 27 | 0 | 5.73 | 155 | 64 | 109 | 75 | 78.2 | 50.7 | 27.5 | 15.66 | 6.23 | 9.4 | 5.74 | 3.77 | 1.18 | 3.16 |
| 3 | 29.43951988 | 40 | 0 | 6.87 | 108 | 54 | 75 | 68 | 82.4 | 49.8 | 32.6 | 10.88 | 4.13 | 6.8 | 4.78 | 3.28 | 1.13 | 2.63 |
| 2 | 26.09472148 | 30 | 0 | 6.04 | 47 | 32 | 38 | 63 | 74.5 | 53.1 | 21.4 | 9.34 | 3.17 | 6.2 | 7.51 | 2.51 | 1.42 | 5.07 |
| 2 | 27.80531375 | 27 | 0 | 5.77 | 69 | 31 | 44 | 80 | 66.6 | 47 | 19.6 | 8.36 | 3.07 | 5.3 | 4.86 | 3.79 | 0.73 | 2.96 |
| 3 | 28.17339389 | 22 | 0 | 5.3 | 43 | 26 | 23 | 67 | 74.9 | 51 | 23.9 | 12.95 | 4.38 | 8.6 | 5.32 | 2.52 | 0.91 | 3.51 |
| 2 | 24.43564987 | 29 | 0 | 4.16 | 119 | 67 | 176 | 83 | 77.3 | 53.2 | 24.1 | 20.45 | 5.76 | 14.7 | 5.64 | 1.77 | 1.09 | 3.76 |
| 2 | 26.227911 | 36 | 0 | 5.04 | 58 | 23 | 34 | 92 | 71.6 | 47.8 | 23.8 | 8.86 | 3.74 | 5.1 | 4.83 | 1.03 | 1.09 | 3.25 |
| 3 | 33.0268987 | 50 | 0 | 5.47 | 40 | 29 | 38 | 57 | 69.5 | 46.5 | 23 | 10.15 | 3.28 | 6.9 | 3.96 | 1.89 | 1.2 | 2.03 |
| 3 | 29.61475436 | 33 | 0 | 8.69 | 148 | 75 | 60 | 100 | 79.3 | 50.2 | 29.1 | 6.89 | 2.48 | 4.4 | 6.47 | 2.81 | 1.13 | 4.18 |
| 3 | 31.21559906 | 46 | 1 | 6.09 | 77 | 42 | 46 | 75 | 73.3 | 45.5 | 27.8 | 5.96 | 3.67 | 2.3 | 5.53 | 1.74 | 1.15 | 3.33 |
| 3 | 38.90185674 | 31 | 0 | 4.67 | 190 | 62 | 49 | 84 | 83.7 | 54.5 | 29.2 | 25.5 | 10.2 | 15.2 | 4.69 | 1.5 | 1.15 | 2.95 |
| 3 | 28.97441546 | 37 | 0 | 13.45 | 192 | 89 | 68 | 76 | 69 | 49.1 | 19.9 | 11.49 | 5.33 | 6.2 | 6.17 | 2.29 | 1.02 | 4.39 |
| 3 | 33.38995616 | 42 | 0 | 6.12 | 55 | 29 | 57 | 90 | 74.3 | 50 | 24.3 | 11.4 | 5.3 | 6.1 | 3.37 | 2.39 | 0.77 | 1.98 |
| 2 | 24.48654048 | 29 | 0 | 5.17 | 22 | 19 | 18 | 93 | 70.6 | 46.6 | 24 | 11.42 | 4.63 | 6.8 | 5.55 | 1.9 | 1.04 | 3.73 |
| 3 | 32.03619321 | 34 | 0 | 4.56 | 61 | 35 | 79 | 71 | 74.9 | 51.6 | 23.3 | 23.8 | 8.2 | 15.6 | 5.8 | 2.3 | 1.35 | 3.99 |
| 2 | 21.36116536 | 27 | 0 | 5.14 | 48 | 17 | 37 | 81 | 78.5 | 52.2 | 26.3 | 11 | 4.4 | 6.6 | 4.76 | 1.75 | 1.24 | 2.94 |
| 2 | 27.12466685 | 40 | 0 | 5.06 | 33 | 34 | 60 | 75 | 67.5 | 46.1 | 21.4 | 4.7 | 2.22 | 2.5 | 4.87 | 3.56 | 0.81 | 2.77 |
| 3 | 32.02154795 | 45 | 0 | 5.62 | 34 | 23 | 69 | 85 | 70.9 | 45.9 | 25 | 5.9 | 3.25 | 2.7 | 4.09 | 2.16 | 1.01 | 2.36 |
| 3 | 32.21993383 | 34 | 0 | 5.03 | 46 | 25 | 38 | 93 | 75.4 | 47 | 28.4 | 4.37 | 2.69 | 1.7 | 6.13 | 2.28 | 1.12 | 4.09 |
| 2 | 26.33049965 | 48 | 0 | 6.7 | 51 | 27 | 88 | 86 | 74.1 | 48.4 | 25.7 | 11.2 | 5.3 | 5.9 | 5.9 | 2.83 | 1.21 | 3.84 |
| 3 | 35.78883616 | 38 | 0 | 4.76 | 26 | 19 | 30 | 78 | 75.5 | 47.4 | 28.1 | 12.41 | 4.69 | 7.7 | 7.49 | 0.82 | 1.3 | 5.39 |
| 2 | 22.98652077 | 48 | 0 | 8.38 | 103 | 78 | 67 | 77 | 74.7 | 51.4 | 23.3 | 15.24 | 7.07 | 8.2 | 5.84 | 3.29 | 1.10 | 3.30 |
| 3 | 30.60080528 | 27 | 1 | 5.14 | 24 | 18 | 18 | 97 | 70 | 46.5 | 23.5 | 4.77 | 2.66 | 2.1 | 4.48 | 0.75 | 1.2 | 2.86 |
| 3 | 36.4765714 | 34 | 0 | 5.11 | 35 | 18 | 20 | 52 | 72.2 | 47.2 | 25 | 14.9 | 5.9 | 9 | 4.65 | 1.8 | 1.17 | 2.81 |
| 3 | 36.4581515 | 38 | 0 | 13.55 | 30 | 23 | 130 | 82 | 69.9 | 48.5 | 21.4 | 14.58 | 7.19 | 7.4 | 6.41 | 4.13 | 0.89 | 3.64 |
| 2 | 27.94987488 | 38 | 1 | 5.43 | 38 | 25 | 26 | 91 | 72.6 | 43.8 | 25.8 | 3.9 | 2.4 | 1.5 | 4.26 | 1.7 | 1.17 | 2.52 |
| 3 | 28.05496089 | 33 | 0 | 10.42 | 52 | 26 | 40 | 100 | 75.2 | 48.2 | 27 | 10.52 | 4.8 | 5.7 | 5.08 | 3.61 | 0.76 | 3.24 |
| 2 | 20.10463015 | 47 | 0 | 5.59 | 26 | 25 | 74 | 69 | 68.9 | 46.8 | 22.1 | 9.18 | 1.66 | 7.5 | 4.71 | 3.59 | 1 | 2.66 |
| 2 | 27.54538727 | 41 | 0 | 7.29 | 40 | 27 | 34 | 58 | 73.7 | 48.4 | 25.3 | 9.2 | 2.8 | 6.4 | 6.00 | 5.12 | 1 | 3.21 |
| 3 | 34.52158864 | 47 | 0 | 5.43 | 23 | 14 | 36 | 66 | 72.3 | 48.1 | 24.2 | 7.95 | 3.17 | 4.8 | 6.62 | 0.77 | 1.76 | 4.38 |
| 3 | 29.96621641 | 35 | 0 | 5.99 | 50 | 29 | 51 | 63 | 77.2 | 47 | 30.2 | 8.27 | 3.89 | 4.3 | 4.81 | 4.03 | 0.87 | 2.61 |
| 3 | 37.54389954 | 73 | 1 | 6.86 | 41 | 25 | 42 | 110 | 78.2 | 48.3 | 29.9 | 6.5 | 3.2 | 3.3 | 5.39 | 2.38 | 1.03 | 3.63 |
| 3 | 28.40466626 | 29 | 0 | 6.89 | 99 | 41 | 74 | 74 | 76.4 | 52.8 | 23.6 | 10.5 | 4.5 | 6.0 | 4.65 | 1.72 | 1.11 | 3.09 |
| 2 | 24.27007484 | 36 | 0 | 5.78 | 22 | 19 | 70 | 78 | 79.6 | 50.4 | 29.2 | 13.73 | 4.25 | 9.5 | 5.63 | 4.91 | 1.17 | 2.78 |
| 3 | 30.09089343 | 43 | 0 | 5.31 | 26 | 18 | 27 | 56 | 72.7 | 48.1 | 24.6 | 10.33 | 4.57 | 5.8 | 4.85 | 0.95 | 1.31 | 3.01 |
| 2 | 23.46936099 | 39 | 0 | 4.73 | 33 | 34 | 74 | 119 | 79.2 | 51.3 | 27.9 | 12.23 | 3.39 | 8.8 | 6.39 | 1.86 | 1.25 | 4.21 |
| 2 | 21.82107226 | 33 | 0 | 5.39 | 62 | 37 | 63 | 129 | 71.3 | 49.3 | 22 | 7 | 3.6 | 3.4 | 5.48 | 3.2 | 1.04 | 3.57 |
| 3 | 28.0380586 | 28 | 0 | 5.34 | 55 | 34 | 168 | 90 | 75.1 | 47.8 | 27.3 | 8.62 | 5.1 | 3.5 | 6.62 | 7.66 | 1.01 | 2.81 |
| 2 | 22.8571078 | 56 | 0 | 6.85 | 60 | 36 | 29 | 91 | 75.7 | 48.9 | 26.8 | 7.78 | 3.45 | 4.3 | 5.52 | 1.26 | 1.09 | 3.43 |
| 2 | 22.26116371 | 33 | 0 | 4.46 | 137 | 56 | 89 | 63 | 80.6 | 54.2 | 26.4 | 8.55 | 2.89 | 5.7 | 8.81 | 3.61 | 1.28 | 5.6 |
| 3 | 28.05725861 | 25 | 0 | 4.99 | 58 | 35 | 55 | 75 | 81.5 | 52.7 | 28.8 | 12.32 | 3.79 | 8.5 | 5.7 | 1.23 | 1.02 | 3.62 |
| 2 | 18.12034734 | 26 | 0 | 5.04 | 88 | 40 | 33 | 71 | 71.9 | 50.8 | 21.1 | 8.2 | 4.9 | 3.3 | 5.22 | 2.78 | 1.14 | 3.48 |
| 2 | 26.71445974 | 40 | 0 | 5.36 | 80 | 33 | 37 | 72 | 76.1 | 52.1 | 24 | 12.39 | 4.31 | 8.1 | 4.66 | 2.31 | 0.85 | 3.29 |
| 2 | 22.29145813 | 45 | 0 | 4.95 | 44 | 21 | 31 | 62 | 64.8 | 45.2 | 19.6 | 9.14 | 4.60 | 4.5 | 3.68 | 1.12 | 0.93 | 2.62 |
| 2 | 16.38387171 | 34 | 0 | 4.78 | 20 | 34 | 37 | 62 | 75.2 | 49.1 | 26.1 | 5.49 | 2.6 | 2.9 | 6.47 | 2.19 | 1.04 | 5.04 |
| 2 | 20.42221769 | 34 | 1 | 4.99 | 19 | 19 | 17 | 46 | 69.5 | 47.5 | 22 | 12.6 | 5.5 | 7.1 | 4.54 | 1.78 | 1.14 | 2.99 |
| 2 | 20.14143085 | 27 | 0 | 4.91 | 17 | 11 | 40 | 85 | 75.6 | 45.7 | 29.9 | 8.2 | 3.42 | 4.8 | 4.6 | 1.17 | 1.18 | 2.76 |
| 3 | 37.55703863 | 38 | 1 | 3.9 | 94 | 37 | 121 | 56 | 73.3 | 44.2 | 29.1 | 7.34 | 3.97 | 3.4 | 5.28 | 3.25 | 1.51 | 3.04 |
| 2 | 25.91951307 | 35 | 0 | 10.42 | 51 | 46 | 34 | 112 | 70.7 | 49 | 21.7 | 14.29 | 6.12 | 8.2 | 4.94 | 2.16 | 0.82 | 3.29 |
| 2 | 27.02194341 | 34 | 1 | 4.33 | 65 | 24 | 57 | 56 | 74.4 | 43.3 | 31.1 | 3.7 | 2.7 | 1.0 | 4.07 | 2.42 | 1.1 | 2.15 |
| 3 | 43.88671494 | 33 | 0 | 5.77 | 60 | 22 | 22 | 64 | 68.8 | 48.2 | 20.6 | 7.04 | 3.83 | 3.2 | 3.84 | 0.67 | 1.35 | 2.21 |
| 3 | 30.50740687 | 32 | 0 | 5.21 | 56 | 32 | 52 | 70 | 76.7 | 51.3 | 25.4 | 14.98 | 6.9 | 8.1 | 5.76 | 2.46 | 1.01 | 3.54 |
| 2 | 20.15100988 | 38 | 0 | 5.57 | 35 | 20 | 71 | 113 | 74.6 | 49.7 | 24.9 | 6.42 | 3.21 | 3.2 | 6.32 | 2.49 | 1.35 | 4.23 |
| 2 | 23.47510529 | 27 | 0 | 7.4 | 23 | 17 | 50 | 98 | 71 | 47.4 | 23.6 | 14.34 | 6.68 | 7.7 | 4.48 | 2.11 | 1.11 | 3.05 |
| 3 | 35.23509343 | 36 | 0 | 5.01 | 54 | 34 | 71 | 68 | 78.5 | 50.1 | 28.4 | 10.5 | 3.6 | 6.9 | 6.24 | 4.63 | 1.17 | 3.95 |
| 3 | 30.31049983 | 52 | 1 | 5.39 | 43 | 26 | 29 | 124 | 84 | 51.7 | 32.3 | 5.92 | 2.54 | 3.4 | 7.05 | 2.12 | 1.66 | 4.52 |
| 2 | 23.49015935 | 44 | 0 | 3.89 | 74 | 36 | 57 | 98 | 68.1 | 45.5 | 22.6 | 8.76 | 2.95 | 5.8 | 5.46 | 2.11 | 0.86 | 4.19 |
| 3 | 28.79891205 | 40 | 0 | 5.1 | 65 | 29 | 20 | 110 | 74.2 | 50.8 | 23.4 | 10.6 | 6.1 | 4.5 | 3.73 | 0.82 | 1.03 | 2.42 |
| 3 | 36.9929587 | 32 | 0 | 5.44 | 55 | 37 | 39 | 128 | 79.2 | 48.9 | 30.3 | 14 | 5.1 | 8.9 | 4.72 | 2.13 | 0.93 | 3.39 |
| 2 | 27.49767939 | 49 | 1 | 5.7 | 98 | 32 | 136 | 150 | 80.9 | 30.9 | 50 | 7.6 | 3.9 | 3.7 | 3.78 | 4.3 | 0.85 | 1.75 |
| 2 | 21.27033106 | 53 | 0 | 12.83 | 43 | 29 | 36 | 59 | 72.9 | 48.6 | 24.3 | 7.52 | 3.59 | 3.9 | 4.93 | 1.39 | 1.45 | 3.3 |
| 2 | 22.97617531 | 40 | 0 | 8.38 | 24 | 13 | 131 | 103 | 70.2 | 46.5 | 23.7 | 5 | 4.3 | 0.7 | 6.94 | 23.62 | 0.63 | 2.46 |
| 2 | 21.68171628 | 52 | 1 | 7.12 | 23 | 23 | 18 | 77 | 77.7 | 46.5 | 31.2 | 12.68 | 3.68 | 9 | 4.9 | 1.37 | 1.14 | 3.05 |
| 3 | 39.09999084 | 44 | 0 | 7.3 | 48 | 122 | 299 | 139 | 72.8 | 44.6 | 28.2 | 14.2 | 9.2 | 5 | 4.53 | 2.25 | 1 | 3.05 |
| 2 | 22.88587888 | 25 | 0 | 4.79 | 37 | 32 | 35 | 85 | 73.3 | 47.2 | 26.1 | 13.88 | 6.08 | 7.8 | 3.55 | 2.29 | 1.11 | 1.68 |
| 2 | 24.99348005 | 58 | 1 | 6.71 | 41 | 27 | 33 | 91 | 76.2 | 46 | 30.2 | 15.34 | 4.17 | 11.2 | 7.59 | 2.94 | 1.31 | 4.72 |
| 3 | 32.39337413 | 31 | 0 | 4.86 | 117 | 54 | 93 | 94 | 77 | 50.3 | 26.7 | 10.6 | 3.9 | 6.7 | 4.98 | 2.38 | 1 | 3.47 |
| 2 | 22.23112233 | 47 | 1 | 5.44 | 26 | 19 | 32 | 70 | 68 | 42.5 | 25.5 | 3.4 | 1.8 | 1.6 | 4.61 | 2.01 | 1.17 | 2.82 |
| 3 | 29.75728671 | 43 | 0 | 6.76 | 52 | 33 | 41 | 81 | 76.4 | 51.6 | 24.8 | 10.12 | 4.15 | 6 | 6.45 | 9.85 | 0.93 | 2.94 |
| 3 | 33.53064092 | 45 | 0 | 11.63 | 39 | 31 | 77 | 91 | 71.6 | 50.5 | 21.1 | 6.95 | 4.02 | 2.9 | 4.83 | 4.88 | 1.1 | 2 |
| 2 | 25.79809634 | 39 | 0 | 5.11 | 36 | 24 | 31 | 74 | 73 | 48.8 | 24.2 | 13.72 | 5 | 8.7 | 5.92 | 1.91 | 1.04 | 4.04 |
| 2 | 27.5331707 | 35 | 0 | 5.42 | 32 | 22 | 28 | 54 | 73.7 | 47.2 | 26.5 | 10.62 | 2.59 | 8 | 4.91 | 1.16 | 1.11 | 3.08 |
| 2 | 23.12060674 | 35 | 0 | 4.64 | 35 | 31 | 29 | 50 | 74.6 | 49.4 | 25.2 | 8.1 | 3.5 | 4.6 | 4.42 | 1.72 | 0.89 | 3.19 |
| 2 | 21.87795448 | 84 | 1 | 6.51 | 19 | 26 | 33 | 81 | 78.3 | 49.5 | 28.8 | 16.93 | 5.14 | 11.8 | 3.64 | 4.95 | 0.95 | 1.29 |
| 3 | 32.58540916 | 37 | 0 | 4.98 | 44 | 36 | 58 | 57 | 77.1 | 51.4 | 25.7 | 7.85 | 3.13 | 4.7 | 6.32 | 5.62 | 1.12 | 3.46 |
| 3 | 42.88308462 | 37 | 0 | 9.28 | 58 | 33 | 57 | 82 | 72.8 | 49.3 | 23.5 | 8.94 | 3.53 | 5.4 | 4.16 | 1.3 | 1.03 | 2.27 |
| 2 | 18.74263986 | 25 | 0 | 5.07 | 14 | 21 | 14 | 83 | 71.8 | 47.5 | 24.3 | 6.4 | 3.5 | 2.9 | 3.92 | 2.15 | 0.79 | 2.53 |
| 2 | 20.09907977 | 51 | 0 | 5.39 | 39 | 25 | 35 | 81 | 74 | 51.2 | 22.8 | 11.2 | 4.4 | 6.8 | 5.93 | 2.39 | 1.32 | 4.02 |
| 2 | 24.20897039 | 43 | 1 | 5.17 | 15 | 16 | 13 | 68 | 74.7 | 48.9 | 25.8 | 6.98 | 2.78 | 4.2 | 5.62 | 1.72 | 1.29 | 3.82 |
| 2 | 25.46968714 | 52 | 0 | 5.89 | 62 | 39 | 89 | 93 | 78.9 | 52 | 26.9 | 11.33 | 5.2 | 6.1 | 4.5 | 2.85 | 0.82 | 2.61 |
| 2 | 23.12337812 | 36 | 0 | 4.95 | 26 | 27 | 32 | 57 | 78.5 | 50.5 | 28 | 19.79 | 7.23 | 12.6 | 4.25 | 1.23 | 1.12 | 2.71 |
| 2 | 26.43457031 | 45 | 0 | 5.63 | 30 | 25 | 57 | 59 | 72.2 | 47.8 | 24.4 | 8.13 | 3.26 | 4.9 | 5.41 | 5.68 | 0.87 | 2.65 |
| 3 | 32.28154055 | 34 | 0 | 4.25 | 50 | 29 | 63 | 76 | 74.8 | 48.4 | 26.4 | 8.1 | 3.1 | 5 | 5.47 | 1.85 | 1.05 | 3.99 |
| 3 | 37.30603091 | 30 | 0 | 4.53 | 161 | 78 | 121 | 59 | 75.7 | 51.1 | 24.6 | 8.49 | 3.59 | 4.9 | 5.7 | 1.95 | 1.32 | 3.49 |
| 2 | 26.76625633 | 45 | 0 | 5.24 | 34 | 25 | 23 | 103 | 75.4 | 45.6 | 29.8 | 5.59 | 2.29 | 3.3 | 5.49 | 3.56 | 0.79 | 3.51 |
| 2 | 22.79416911 | 42 | 0 | 5.04 | 25 | 18 | 48 | 69 | 78.1 | 51.9 | 26.2 | 9.96 | 3.72 | 6.2 | 4.28 | 2.63 | 0.91 | 2.4 |
| 2 | 26.42281914 | 54 | 0 | 9.29 | 23 | 23 | 24 | 92 | 77.9 | 52.7 | 25.2 | 10.3 | 4.78 | 5.5 | 5.5 | 1.91 | 1.25 | 3.51 |
| 2 | 27.76854324 | 27 | 0 | 4.78 | 43 | 30 | 37 | 75 | 84.7 | 54.6 | 30.1 | 17.54 | 6.1 | 11.4 | 5.13 | 9.17 | 0.74 | 1.86 |
| 2 | 27.37834485 | 45 | 0 | 5.58 | 61 | 30 | 64 | 72 | 77.9 | 52 | 25.9 | 21.6 | 10.1 | 11.5 | 4.45 | 2.52 | 0.9 | 2.43 |
| 2 | 24.03061231 | 33 | 0 | 4.94 | 78 | 36 | 31 | 47 | 71.2 | 48.2 | 23 | 16.54 | 6.52 | 10 | 4.71 | 1.91 | 0.98 | 2.91 |
| 2 | 24.61080647 | 31 | 0 | 4.4 | 763 | 691 | 58 | 76 | 68.2 | 44 | 24.2 | 11.08 | 6.43 | 4.7 | 4.38 | 2.04 | 0.88 | 2.16 |
| 2 | 24.64065043 | 34 | 0 | 5.10 | 51 | 30 | 29 | 70 | 77.5 | 46.8 | 30.7 | 10.33 | 3.93 | 6.7 | 4.48 | 1.88 | 0.85 | 2.79 |
| 3 | 29.68839836 | 40 | 0 | 7.1 | 93 | 45 | 70 | 68 | 83.3 | 47.7 | 35.6 | 10.3 | 4.5 | 5.8 | 6.22 | 5.3 | 1.01 | 3.76 |
| 2 | 26.09611193 | 49 | 0 | 5.64 | 79 | 42 | 34 | 84 | 71.2 | 47.1 | 24.1 | 10.07 | 5.23 | 4.8 | 4.62 | 1.29 | 1.29 | 2.79 |
| 3 | 28.27637736 | 43 | 1 | 6.39 | 38 | 26 | 22 | 62 | 77.7 | 49 | 28.7 | 8.71 | 3.71 | 5 | 3.44 | 1.44 | 0.87 | 2.17 |
| 2 | 26.12568792 | 44 | 0 | 7.18 | 79 | 49 | 58 | 79 | 78.9 | 50.4 | 28.5 | 15.65 | 6.47 | 9.2 | 4.98 | 2.67 | 0.9 | 3.2 |
| 3 | 35.50326411 | 26 | 0 | 5.48 | 109 | 40 | 50 | 65 | 71.3 | 45.6 | 25.7 | 11.18 | 6.09 | 5.1 | 3.7 | 1.38 | 1 | 2.23 |
| 3 | 33.1351757 | 26 | 0 | 5.99 | 99 | 53 | 77 | 93 | 84.7 | 53 | 31.7 | 12.66 | 4.79 | 7.9 | 4.73 | 1.77 | 0.91 | 3.28 |
| 3 | 37.51318105 | 30 | 0 | 5.98 | 31 | 17 | 35 | 84 | 75.1 | 52.7 | 22.4 | 13.1 | 4.5 | 8.6 | 5.26 | 2.46 | 1.35 | 3.49 |
| 3 | 28.61841011 | 37 | 1 | 5.25 | 81 | 44 | 61 | 60 | 76.3 | 45.4 | 30.9 | 7.71 | 3.65 | 4.1 | 6.04 | 2.61 | 0.99 | 3.73 |
| 3 | 36.96489716 | 28 | 1 | 5.27 | 48 | 28 | 27 | 62 | 62.9 | 43.2 | 19.7 | 6.49 | 3.53 | 3 | 5.9 | 3.02 | 1.11 | 3.92 |
| 3 | 34.05641747 | 42 | 0 | 5.45 | 39 | 28 | 53 | 78 | 70.8 | 47.2 | 23.6 | 8.06 | 3.96 | 4.1 | 5.47 | 2.83 | 1.08 | 3.7 |
| 3 | 38.02277883 | 47 | 0 | 6.8 | 69 | 38 | 57 | 54 | 71.6 | 45.7 | 25.9 | 13.71 | 6.53 | 7.2 | 5.62 | 1.5 | 1.16 | 3.68 |
| 3 | 28.460186 | 38 | 0 | 5.22 | 24 | 21 | 50 | 68 | 70.94 | 49.2 | 21.7 | 8.3 | 3.04 | 5.3 | 4.62 | 2.99 | 0.81 | 3.07 |
| 3 | 29.70507749 | 44 | 0 | 5.36 | 65 | 42 | 44 | 77 | 76.5 | 48.7 | 27.8 | 5.56 | 4.25 | 1.3 | 6.51 | 21.26 | 0.62 | 1.3 |
| 3 | 29.94938469 | 24 | 0 | 5.87 | 65 | 33 | 194 | 109 | 75.1 | 51.7 | 23.4 | 11.3 | 5.8 | 5.5 | 5.13 | 3.15 | 0.98 | 3.58 |
| 3 | 29.57612991 | 44 | 1 | 8.83 | 33 | 30 | 33 | 82 | 79.5 | 47.4 | 32.1 | 5.63 | 2.64 | 3 | 7.24 | 3.39 | 0.889 | 5.08 |
| 2 | 26.55136299 | 39 | 0 | 8.26 | 31 | 19 | 58 | 61 | 81 | 53.4 | 27.6 | 25 | 10.4 | 14.6 | 5.42 | 2.94 | 0.94 | 3.69 |
| 3 | 30.33661715 | 41 | 0 | 5.37 | 69 | 34 | 67 | 94 | 73.5 | 47 | 26.5 | 8.9 | 5 | 3.9 | 5.35 | 2.76 | 1.07 | 3.83 |
| 3 | 28.65724627 | 33 | 1 | 6.47 | 13 | 17 | 22 | 120 | 75.9 | 47.3 | 28.6 | 4.64 | 2.87 | 1.8 | 4.49 | 0.9 | 1.28 | 2.57 |
| 3 | 35.75775846 | 33 | 0 | 4.91 | 74 | 32 | 27 | 101 | 71.8 | 48.3 | 23.5 | 14.5 | 6.08 | 8.4 | 5.03 | 1.85 | 1.01 | 3.49 |
| 3 | 41.84658432 | 48 | 0 | 4.98 | 41 | 27 | 60 | 61 | 81 | 49 | 31.9 | 7.34 | 2.2 | 5.1 | 6.53 | 1.88 | 1.43 | 4.32 |
| 3 | 39.98623912 | 41 | 0 | 15.79 | 124 | 119 | 199 | 94 | 72.9 | 51.6 | 21.3 | 16.07 | 5.59 | 10.5 | 6.18 | 5.76 | 0.79 | 3.78 |
| 3 | 33.20464897 | 22 | 0 | 6.42 | 71 | 36 | 49 | 82 | 77.4 | 36.4 | 31 | 8 | 4.4 | 3.6 | 4.85 | 4.72 | 0.86 | 2.93 |
| 2 | 23.84787305 | 34 | 0 | 18.88 | 38 | 26 | 34 | 116 | 77.9 | 52.1 | 25.8 | 12.71 | 5.42 | 7.3 | 8.4 | 7.17 | 1.01 | 4.51 |
| 3 | 29.55764071 | 40 | 0 | 5.81 | 29 | 25 | 42 | 58 | 74.9 | 48.5 | 26.4 | 14.77 | 5.27 | 9.5 | 5.08 | 2.21 | 1.21 | 2.78 |
| 3 | 35.21075948 | 47 | 0 | 5.29 | 49 | 34 | 47 | 61 | 72.6 | 45.4 | 27.2 | 9.58 | 3.72 | 5.9 | 3.14 | 2.77 | 0.82 | 1.41 |
| 3 | 33.98788007 | 26 | 0 | 5.15 | 187 | 72 | 79 | 80 | 76.3 | 46.7 | 26.6 | 9.56 | 5.73 | 3.8 | 3.55 | 0.46 | 1.23 | 2.1 |
| 3 | 28.0680631 | 30 | 0 | 4.51 | 93 | 50 | 57 | 60 | 68.8 | 46.1 | 22.7 | 9 | 4.5 | 4.5 | 5.04 | 1.75 | 1.14 | 3.27 |
| 3 | 31.45445951 | 36 | 0 | 4.71 | 93 | 48 | 38 | 82 | 70.1 | 46.5 | 23.6 | 14.97 | 7.49 | 7.5 | 4.16 | 1.76 | 0.8 | 2.62 |
| 3 | 35.7068011 | 26 | 0 | 7.13 | 79 | 162 | 3263 | 157 | 72.4 | 48.6 | 23.8 | 18.42 | 11.97 | 6.4 | 7.02 | 7.46 | 1.14 | 2.55 |
| 3 | 31.30428378 | 38 | 0 | 5.7 | 56 | 26 | 24 | 82 | 74.3 | 50.5 | 23.8 | 10.3 | 3.83 | 6.5 | 4.77 | 1.48 | 1.06 | 3.28 |
| 3 | 34.22522672 | 33 | 0 | 5.31 | 64 | 36 | 64 | 73 | 76.3 | 48.2 | 28.1 | 10.25 | 1.81 | 8.4 | 5.01 | 3.03 | 0.76 | 3.01 |
| 3 | 30.32915815 | 50 | 0 | 5.52 | 57 | 43 | 48 | 70 | 78.4 | 48.9 | 29.5 | 9.99 | 4.63 | 5.4 | 5.73 | 3.88 | 1.08 | 3.45 |
| 3 | 37.98796336 | 40 | 0 | 5.8 | 64 | 36 | 97 | 77 | 74.3 | 48.1 | 26.2 | 10.37 | 4.42 | 5.9 | 6.56 | 1.68 | 1.32 | 4.43 |
| 2 | 25.64946047 | 38 | 0 | 7.79 | 45 | 19 | 43 | 80 | 72.1 | 50.5 | 21.6 | 16.1 | 6.6 | 9.5 | 3.84 | 1.22 | 1.17 | 2.46 |
| 3 | 28.83889771 | 37 | 0 | 4.54 | 77 | 29 | 36 | 85 | 73.6 | 48.7 | 24.9 | 15.3 | 5.6 | 9.7 | 4.82 | 1.62 | 1.42 | 3.12 |
| 3 | 28.45877266 | 47 | 1 | 5.43 | 23 | 25 | 63 | 80 | 75 | 46.3 | 28.7 | 17.12 | 6.26 | 10.9 | 5.95 | 1.45 | 1.67 | 4.15 |
| 2 | 26.75195885 | 35 | 0 | 5.42 | 33 | 17 | 32 | 53 | 75.1 | 49.2 | 25.9 | 8.88 | 3.49 | 5.4 | 5.59 | 1.54 | 0.9 | 3.72 |
| 2 | 26.6436952 | 53 | 0 | 8.7 | 32 | 29 | 70 | 90 | 71.8 | 45 | 26.8 | 8.53 | 3.42 | 5.1 | 5.47 | 2.8 | 1.08 | 3.23 |
| 3 | 28.34999784 | 36 | 0 | 6.25 | 69 | 40 | 52 | 72 | 81.3 | 49.2 | 32.1 | 26.6 | 6.8 | 19.8 | 6.74 | 3.24 | 0.89 | 4.53 |
| 3 | 37.66812007 | 39 | 0 | 5.61 | 97 | 42 | 66 | 74 | 71.1 | 49.7 | 21.4 | 15 | 7.08 | 7.9 | 5.04 | 2.87 | 0.96 | 3.05 |
| 3 | 38.19195811 | 44 | 1 | 5.4 | 38 | 30 | 88 | 85 | 75.6 | 49.1 | 26.5 | 11.9 | 4.19 | 7.7 | 6.17 | 4.15 | 0.97 | 3.52 |
| 2 | 25.50961113 | 36 | 0 | 4.71 | 50 | 28 | 90 | 89 | 74.8 | 50 | 24.8 | 11.1 | 4.2 | 6.9 | 5.68 | 3.22 | 1.09 | 3.27 |
| 3 | 35.46797434 | 30 | 0 | 6.00 | 39 | 25 | 25 | 120 | 69.9 | 45.3 | 24.6 | 12.24 | 4.17 | 8.1 | 3.17 | 1.97 | 0.98 | 1.38 |
| 3 | 28.77285322 | 41 | 0 | 5.29 | 73 | 30 | 46 | 81 | 74.3 | 46.5 | 27.8 | 9.02 | 4.42 | 4.6 | 5.55 | 2.19 | 1.10 | 3.51 |
| 3 | 35.5904878 | 36 | 0 | 7.34 | 241 | 131 | 251 | 80 | 71 | 47.9 | 23.1 | 13.06 | 5.4 | 7.7 | 6.63 | 5.77 | 1 | 3.78 |
