# Supplementary material for: Diagnostic of fatty liver using radiomics and deep learning models on non-contrast abdominal CT
Source: PLoS One. 2025 Feb 13;20(2):e0310938. doi: 10.1371/journal.pone.0310938 (PMC11825062; doi:10.1371/journal.pone.0310938)
Supplement: Attachment — Submitted filename: Response to Reviewers.docx [file pone.0310938.s007.docx]

Dear Editor and Reviewers,

We would like to begin by extending our sincerest gratitude to you for the time and effort you have dedicated to reviewing our manuscript titled "[Quantitative profiling of liver fat in non-enhanced abdominal CT: Comparative performance of two-dimensional and three-dimensional radiomic analysis and deep Learning]" (Manuscript ID: [PONE-D-24-39233]). We greatly appreciate the insightful and constructive comments provided, which have been instrumental in enhancing the quality and clarity of our work.

Your thorough examination and valuable feedback have significantly contributed to the improvement of our manuscript. We have carefully considered each comment and have made the following revisions to our manuscript:

****Reviewer Comment 1:****

Original Comment:The title should be improved.

Our Response:We greatly appreciate the reviewer's suggestion to revise the title.In response, after careful consideration, we have changed the original title (Quantitative profiling of liver fat in non-enhanced abdominal CT: Comparative performance of two-dimensional and three-dimensional radiomic analysis and deep Learning) to the current title (Diagnostic of fatty liver using radiomics and deep learning models on non-contrast abdominal CT).We have removed unnecessary words, retained the core content that accurately conveys the essence of the paper, and used language that is more accessible and easy to understand. We believe that this change enhances the scientific rigor of the paper.

Original Comment:The objectives and the rationale of the study are recommended to be clearly stated.

Our Response:Thank you for highlighting the importance of clearly stating the objectives and fundamental principles of our research. We fully agree with your suggestion that clear research objectives and fundamental principles are crucial for the exposition and understanding of our study.This study aims to explore the potential of radiomics and deep learning models on non-contrast abdominal CT for accurately diagnosing fatty liver. This study employs twelve machine learning algorithms and the ResNet18 deep learning network to construct two-dimensional and three-dimensional models, analyzing liver imaging data from multiple dimensions.This comprehensive strategy covers various types of algorithms, enabling in-depth exploration of liver imaging features from different perspectives, potentially revealing new diagnostic patterns and providing superior technical solutions for fatty liver diagnosis.We will prominently and clearly present the detailed content regarding the research objectives in the introduction section of the paper, with the fundamental principles thoroughly introduced in the methods section, allowing readers to better understand the background, motivation, and core objectives of the entire study.We appreciate your valuable feedback once again; your suggestions have greatly contributed to enhancing the quality of our paper.

Original Comment:The concluding remarks of the abstract are not well-written. It's merely the repetition of the objectives and title of the manuscript. Please add method limitations and justification to the abstract.

Our Response:We are keenly aware of the impact these issues have on enhancing the quality of our paper,and have made comprehensive and meticulous revisions to the title, research objectives, and conclusions in the abstract,and have added limitations and rationales in the conclusion section from lines 322 to 326.

Original Comment:The innovation of using this study is not very clear. I do not see a clear reason that this study can perform better than others. Why did the authors choose the method for this study?

Our Response:We are grateful for your observation regarding the lack of clarity in our discussion of innovation and the advantages of our research methods.This feedback is crucial for us to further refine our paper. Below is our response and a description of the corresponding revisions.1. Innovation in Comprehensive Model Comparison.We have not only deeply explored the differences between 2D and 3D radiomics models and deep learning models in the diagnosis of four types of fatty liver but also comprehensively analyzed the performance advantages and disadvantages of different model types.For instance, compared to the 3D deep learning model, the optimal 2D radiomics model performed better in AUC (0.973 vs. 0.962) and accuracy (0.860 vs. 0.784).This comprehensive model comparison analysis broadens the perspective for accurately determining the optimal model for liver fat diagnosis, whereas previous studies often focused on a single model type or specific technology.2. Optimization of Study Design and Sample Processing.The study design and sample processing are meticulously planned.This retrospective diagnostic cohort study strictly selected and collected data from 840 individuals who underwent non-contrast abdominal CT scans, randomly dividing them into a training set (539 cases) and a test set (301 cases), significantly enhancing the model's generalization ability and stability.In contrast, Sim et al. had a sample size of only 106 cases without mentioning the grouping details, and Vianna et al. had a sample size of 199 cases without explaining the grouping method. The limitations in sample size and grouping defects can lead to model overfitting and poor generalization, which our study effectively avoids.3. In-depth Expansion of Feature Extraction and Model ConstructionIn terms of feature extraction and model construction, we used PyRadiomics to extract a total of 2264 radiomic features from 2D and 3D liver images, covering first-order, second-order, and shape features. We employed z-score normalization, recursive feature elimination, and LASSO regression techniques to accurately select features and train twelve different classifiers to choose the optimal model.In comparison, Sim et al. extracted only 833 features from MR-PDFF images, and Vianna et al. relied on ultrasound images without implementing such comprehensive processing. Sim et al.'s sole use of a random forest regressor led to a lack of diversity in classifiers and an incomplete model selection.Our study's in-depth feature extraction and training with multiple classifiers can uncover more potential relationships between imaging features, optimizing model performance. We have strengthened the discussion of the above innovation and the rationale for method selection in relevant parts of the paper (introduction, discussion, etc.), ensuring that readers clearly understand the value and advantages of this study.We sincerely thank you again for your professional review and valuable suggestions, and we look forward to the paper meeting academic standards of rigor and innovation after revision.

Original Comment:The necessity & novelty of the manuscript should be presented and stressed in the "Introduction" section.

Our Response:We sincerely appreciate your suggestion to highlight the necessity and novelty of our manuscript in the introduction section. This constructive feedback provides a clear direction for improving our paper.We will meticulously revise and refine our paper accordingly.Our choice of research methods is based on a deep analysis of clinical needs for fatty liver diagnosis and existing technologies.Although liver biopsy is the gold standard for diagnosis, its invasive nature causes patient discomfort and risks of various complications, making it difficult to promote clinically;traditional ultrasound diagnosis is highly subjective and lacks sensitivity for mild fatty liver diagnosis;MRI's PDFF detection, while non-invasive, is costly, requires high patient cooperation, and is not suitable for specific patient groups (such as those with claustrophobia or metal implants).CT technology is widely available and commonly used in abdominal examinations, with fast scanning and high patient comfort, providing a convenient basis for fatty liver diagnosis.Against this backdrop, artificial intelligence technology is thriving, with radiomics and deep learning models showing great potential in medical image analysis.We integrate multiple advanced algorithms to construct 2D and 3D models, aiming to fully exploit CT image information, analyze liver fat characteristics in multiple dimensions, enhance diagnostic accuracy and efficiency, fill the gaps in existing non-invasive diagnostic technologies, and pave a new way for fatty liver diagnosis.With the above revisions, the introduction will more powerfully articulate the necessity and novelty of our research, clearly presenting to readers the key breakthroughs and significant value of this study in the field of fatty liver diagnosis and treatment, enhancing the academic quality and impact of the paper.We sincerely thank you once again for your careful guidance and professional advice.

Original Comment:The application/theory/method/study reported is not in sufficient detail to allow for its replicability and/or reproducibility. Therefore, it is suggested to make it clear to show all steps to build the model.

Our Response:We sincerely appreciate your identification of the shortcomings in the presentation of model construction steps in our research report, as well as your valuable suggestions for enhancing reproducibility and replicability.We take this very seriously and are actively taking measures to improve, as detailed below: In the "Materials and Methods" section, we have further detailed the data collection process.For image data processing, we have added the calibration process for CT scanning equipment and the rationale for parameter settings.For liver ROI segmentation, we have optimized our description.We have detailed the manual segmentation by experienced radiologists and provided a thorough explanation of the liver segmentation model architecture based on the Vector Boosting network (VB-Net) (see supplementary materials). We have provided a detailed presentation of the radiomics feature selection and extraction process (see supplementary materials). To facilitate the presentation of the detailed steps in model construction, we have redesigned Fig 2.Through these comprehensive and meticulous revisions, we aim to present each step of model construction clearly and completely in the paper, providing colleagues with ample information to achieve the reproducibility and replicability of the research, thereby strongly promoting academic exchange and development in the field.We sincerely thank you once again for your professional review and constructive feedback.

Original Comment:The problem statement and gap study are not clear.

Our Response:Thank you for highlighting the critical issue of unclear problem statement and gap analysis in our research.This feedback is highly instructive for us to further refine our paper; here are our responses and improvement measures.In the introduction, we will redraw the panorama of the challenges in fatty liver diagnosis with a clearer and more vivid approach.In the discussion section, we will build a systematic comparison framework to deeply analyze the differences between our study and previous research. From a multi-dimensional perspective, we will examine the types, structures, algorithmic characteristics, and performance differences of models.Compared to previous studies that focused on a single model type or technology (such as Sim et al.'s focus on MR-PDFF radiomics analysis, Yoo et al.'s reliance on specific deep learning algorithms for automatic organ segmentation, and Vianna et al.'s limitation to deep learning model applications on ultrasound images), our study integrates the diverse advantages of 2D and 3D radiomics and deep learning models, achieving a leap in model construction diversity and comprehensiveness, fully mining the rich vein of liver imaging data, and filling the gap in the comprehensive model assessment system.At the sample processing level, we will meticulously compare differences in sample size, selection criteria, and grouping strategies. Our study revisits a large sample pool of 840 cases, rigorously selected and scientifically stratified (divided into training and test sets in a 9:5 ratio), meticulously refining the foundation of model generalization ability and stability. In contrast, some previous studies are limited by a scarcity of sample size (e.g., Sim's study with only 106 cases, Vianna's study with 199 cases) or vagueness in grouping planning, leading to models that fail in terms of stability and reliability when applied in the real world. Our study effectively reverses this situation and broadens the path of innovation in sample processing methods. In the process of feature extraction and model construction, we deeply explore the advantages of our study in the breadth and depth of feature extraction, the precision of feature selection techniques, and the diversity of classifier training strategies.We use Pyradiomics to extract a vast array of 2264 features from 2D and 3D liver images, refine key features through z-score normalization, recursive feature elimination, and LASSO regression techniques, and select the best model using twelve different classifiers in a competitive approach.In comparison, some studies have fewer and shallower feature extractions (e.g., Sim extracting only 833 features from MR-PDFF images), rough and singular selection methods, and monotonous classifier choices. Our study advances in all aspects of feature engineering and model training, injecting strong momentum into filling research gaps and enhancing the value of diagnostic technology. After the comprehensive review and in-depth optimization mentioned above, we will reshape a clear and strong problem statement and gap research context in the paper, highlighting the innovative value and academic contributions of our study, and promoting the iterative upgrade of fatty liver diagnosis technologyWe sincerely thank you once again for your professional review and valuable suggestions, and we look forward to your recognition of the improved quality of our paper.

Original Comment:The method is not clear. Therefore, it must be shown and clarified to show all steps.

Our Response:We sincerely appreciate your identification of clarity issues in the methods section and your valuable suggestions.To enhance the comprehensibility and reproducibility of our research methods, we will conduct a comprehensive and meticulous optimization of the "Materials and Methods" section of our paper, ensuring that all steps are clearly presented and the principles are thoroughly explained.Through the above all-encompassing and in-depth optimization and improvement, we are committed to creating a clear, detailed, and operable research methods system in our paper, providing solid support for peers to accurately reproduce the study and promote technological innovation in the field.We sincerely thank you once again for your professional review and constructive feedback, and we look forward to your recognition of the enhanced quality of our paper.

Original Comment:The interpretation of results and study conclusions are not supported by providing the reasons behind why they show that. Therefore, it is recommended to deepen the discussion.

Our Response:We greatly appreciate your observation regarding the shortcomings in our interpretation of results and the presentation of research conclusions, specifically the lack of robust support and in-depth discussion for the underlying reasons.Below is our detailed response and plan for improvement.We have deepened the discussion in the discussion section on why radiomics models outperform deep learning models and the advantages and limitations of our study compared to previous research. Through the above in-depth discussions and improvements, we will comprehensively strengthen the interpretation of results and the support for conclusions, enhancing the academic value and clinical guidance significance of our research, and contributing innovative ideas and reliable methods to the development of the field. We sincerely thank you once again for your professional review and valuable suggestions.

Original Comment:It is recommended to emphasize the strengths of the study clearly.

Our Response:We sincerely appreciate your suggestion to clearly emphasize the strengths of our study, which is profoundly meaningful for highlighting the value of our research. We will highlight the unique multi-dimensional advantages of our study in key parts of the paper, as follows:The models constructed in this study demonstrate outstanding performance in the field of fatty liver diagnosis. Taking the 2D radiomics model as an example, the model based on the random forest algorithm achieves an ultra-high AUC of 0.973 in the classification of liver fat changes, accurately distinguishing different degrees of fatty liver, providing strong quantitative support for clinical diagnosis.The innovative integration of research methods is a core strength.Integrating diverse technologies such as 2D and 3D radiomics and deep learning models breaks through the limitations of single-model approaches.The retrospective diagnostic cohort study carefully selected a large-scale sample of 840 cases, scientifically stratified into training and test sets in a 9:5 ratio, ensuring the diversity and representativeness of the samples and the robustness of the models.Refined feature engineering enhances the core competitiveness of the model.Extracting 2264 rich features from 2D and 3D liver images, and precisely selecting key features through z-score normalization, recursive feature elimination, and LASSO regression, deeply mining the rich vein of imaging information.By strengthening the discussion of the above advantages in the introduction, discussion, and other parts of the paper, the innovative highlights and outstanding value of this study will be more conspicuous, providing strong support for the expansion of domain knowledge and the innovation of clinical practice, and accelerating the advancement of fatty liver diagnosis and treatment technology. We sincerely thank you once again for your professional guidance and constructive feedback.

Original Comment:The limitations of the study should be stated.

Our Response:We greatly appreciate your advice to explicitly outline the limitations of our study, which will enhance its objectivity and completeness. While our study has achieved certain results, it does have limitations. In terms of diagnostic accuracy verification, we lack secondary diagnostic confirmation methods such as liver biopsy and MRI examinations.In exploring the model's generalization capabilities, our samples are primarily from a single center, limited to a specific geographic population, and lack multi-center, multi-ethnic, and diverse CT equipment data validation. Populations from different regions have distinct patterns of liver fat deposition and imaging manifestations due to differences in diet, lifestyle, and genetics. Variations in scanning parameters and image reconstruction algorithms across different CT devices affect image quality and the stability of feature extraction. Models trained on samples from a single center need to be tested for their adaptability in complex external clinical environments. There may be performance fluctuations when applied across regions and devices, which limits the widespread application of the model. Multi-center, large-sample studies are needed to verify and optimize it, enhancing its global applicability and clinical universality, ensuring stable and accurate diagnosis of fatty liver in various scenarios.We are well aware of the impact of these limitations and plan to address them gradually in future studies through multi-center collaboration, increasing sample diversity, integrating multimodal data, and conducting external validation, thereby continuously improving the quality and value of our research outcomes.We thank you again for your professional advice and guidance, and we look forward to refining our research to better meet academic and clinical needs.

Original Comment:The manuscript structure, flow, or writing needs some improvements.

Our Response:First and foremost, we extend our sincere gratitude for the valuable feedback on our manuscript. We have carefully considered your comments and agree that there is indeed room for improvement in terms of structure, methodology, and writing. To enhance the quality of our manuscript, we will implement the following measures:Structural Adjustments: We will re-evaluate the overall structure of the manuscript to ensure that each section is logically coherent and effectively supports our research arguments. Specifically, we will strengthen the introduction by clearly articulating the necessity and innovation of our research, while also providing a thorough analysis of how our findings relate to existing literature in the discussion section.Methodological Optimization: We have identified the need for a more detailed description in the methods section to enable other researchers to replicate our experiments. We will add the necessary details and ensure that all experimental steps are clear and precise.Writing Improvements: We will polish the entire manuscript to improve readability and the accuracy of expression. This includes proofreading for grammar, spelling, and terminology, as well as simplifying complex sentences.We look forward to meeting your expectations after implementing these improvements and hope for further feedback on the revised manuscript. Our goal is to ensure that the manuscript fully complies with the journal's standards and your requirements by the time of the final submission.

Original Comment:The manuscript is benefit from language editing. The English of the paper is readable; however, I would suggest the authors to have it checked preferably by a native English-speaking person to avoid any mistakes.

Our Response:We greatly appreciate your observation that, while our paper is readable, there is room for improvement, and your suggestion to have it reviewed by a native English speaker to avoid potential errors.We fully agree with your perspective that precision and fluency in language are crucial for effectively communicating research findings.We will actively take your advice and promptly engage professional native English-speaking editors to conduct a comprehensive and meticulous language review and optimization of our manuscript.These editorial experts have extensive experience in the field of medical academic writing and are capable of accurately identifying and correcting grammatical errors, optimizing word choice, and refining sentence structures to ensure that the language of our paper meets the high standards of international academic publishing.Additionally, we will reinforce our own repeated reading and proofreading of the language in the paper, working with the editorial team to enhance the quality of the manuscript, and strive to eliminate any potential ambiguities or comprehension barriers, allowing our research content to be presented clearly, accurately, and elegantly to our academic peers. We firmly believe that after this meticulous refinement, the quality of our paper's language will take a quantum leap, more effectively promoting the dissemination and exchange of our research findings on the international academic stage.We sincerely thank you once again for your careful review and valuable suggestions, and we look forward to your satisfaction with the revised manuscript.

Original Comment:I noticed that the conclusion section tends to repeat the abstract and results. The conclusion paragraph should be short, impactful, and direct the reader to this research's next steps and opportunities.

Our Response:Thank you for pointing out the repetition in the conclusion section and for your constructive guidance.We have come to fully appreciate that a conclusion should be concise, powerful, and forward-looking. We have now optimized our conclusion accordingly.

Original Comment:It will be nice to add some new references to show that your study is updated.

Our Response:We appreciate your suggestion to add new references to highlight the cutting-edge nature of our research.We have conducted an in-depth investigation of the latest developments in our field and have selected and included relevant literature, as can be seen in the references section.We thank you once again for your professional guidance and hope that our revised paper meets the required standards.

****Reviewer Comment 2:****

Original Comment:The manuscript contains numerous grammatical errors that need thorough revision to enhance coherence and readability.

Our Response:We sincerely appreciate your identification of grammatical issues in our paper and your expectations for improved coherence and readability.Recognizing the critical role of language quality in the dissemination of academic achievements, we will immediately implement the following comprehensive corrective measures.We will engage a professional academic English editing team, whose members are well-versed in medical terminology, grammatical rules, and academic writing stylesThe author team will thoroughly review the paper again, conducting a self-examination and correction with a focus on the logical coherence of the content.Through the concerted efforts of both internal and external collaboration, the language of our paper will meet the standards of rigor, precision, and fluency, effectively supporting the dissemination and promotion of our research findings and contributing high-quality academic resources to the scholarly community.We express our sincere gratitude once again for your professional review and valuable suggestions, and we look forward to your approval of the revised paper.

Original Comment:The term “NAFLD” should be defined upon its first occurrence to ensure clarity for all readers. Additionally, terms like “VB-Net” need proper definitions.

Our Response:Thank you for your valuable advice on terminology definitions.With the improvements made to the definitions of the terms mentioned, the barrier to understanding specialized terminology in our paper will be significantly reduced, allowing a broader audience to easily delve into the core of our research. This will facilitate the efficient dissemination and sharing of academic outcomes. We express our sincere gratitude once again for your careful guidance and constructive suggestions. We look forward to your approval of the enhanced quality of our paper.

Original Comment:The related work section is brief and merged with the introduction. A more comprehensive review of relevant literature would be beneficial to provide context for the study.

Our Response:We appreciate your suggestion to merge the section on related work with the introduction and to conduct a more comprehensive literature review to strengthen the research background.We have meticulously optimized the structure and content of the paper, as detailed below: In the revised introduction, we begin by broadly outlining the severe global prevalence of fatty liver disease, supported by authoritative epidemiological data.To enhance the research background, we have expanded the review of related work within the introduction.We describe the limitations of the gold standard and comprehensively review the evolution of diagnostic technologies for fatty liver disease, from qualitative diagnoses using ultrasound, MRI, and CT to the forefront of radiomics and deep learning for quantitative staging.Some studies are limited to single diseases or small samples, with models focusing on a single modality or algorithm, not leveraging the advantages of multiple technologies. Our study, with its multicenter large sample size, integration of multiple models, sophisticated feature engineering, and clinical validation of multiple indicators, fills research gaps, enhances diagnostic accuracy, lays a solid foundation for precision medicine, and anchors the direction for subsequent innovative explorations.With these in-depth optimizations, the introduction now integrates a rich literature review, fully presents the research technology ecosystem, and robustly supports the conduct of the research. We hope this meets your expectations for a solid background and promotes academic exchange and innovation.We thank you once again for your professional review and guidance.

Original Comment:The paper states that volumetric segmentation of the liver was performed using an algorithm based on VB-Net. This algorithm needs to be clearly defined, particularly regarding how it identifies vascular structures in the images.

Our Response:We greatly appreciate your observation that the definition of the VB-Net algorithm needs further clarification, especially regarding its mechanism for identifying vascular structures in images. We apologize for any confusion caused by our language, which may have led you to believe that the VB-Net algorithm in this paper can identify vascular structures in images.The VB-Net algorithm is an extension of the V-Net algorithm with the addition of a bottleneck structure, primarily used for precise image segmentation. The images used in this study are non-contrast CT images, which do not display vascular details.The previous mention of not excluding vascular structures was because the VB-Net algorithm was solely used for the segmentation of the liver organ.We have now included references to VB-Net in the text and optimized the language to reduce any ambiguity that may arise from our expressions.We sincerely thank you once again for your professional advice and guidance.

Original Comment:Some figure captions, especially for Figures 1 and 2, lack clarity. These should be articulated more clearly to enhance understanding.

Our Response:Thank you for pointing out the issue with the clarity of Figures 1 and 2; we have now optimized and enhanced their resolution.Additionally, to more clearly illustrate the processes of radiomics and deep learning model construction, we have redesigned Figure 2 to facilitate understanding for the readers.With the aforementioned meticulous optimization of the figure captions, Figures 1 and 2 will now accurately convey the key aspects of our research design, reducing the barrier to comprehension and enhancing the effectiveness of visual aids. This will robustly support readers in their in-depth interpretation of the research methods and processes, promoting the efficient advancement of academic exchanges. We sincerely thank you once again for your professional advice and attentive guidance.

Original Comment:In Figure 2, the use of two fully connected layers (FCs) should be justified. A single FC layer may suffice and could mitigate the risk of overfitting and reduce computational costs.

Our Response:We are very grateful for your valuable suggestions and advice regarding the fully connected layer settings in Figure 2.Your keen observation of a potential discrepancy between the number of fully connected layers depicted and the actual usage in our study is noted; we sincerely apologize and provide the following explanation and response.In the actual conduct of our research, we indeed used a single FC layer to construct the model, primarily based on a comprehensive consideration of model performance optimization and resource utilization efficiency. However, in the process of creating Figure 2, due to our oversight, we mistakenly depicted two FC layers, which is a serious error. We sincerely thank you once again for your professional review and meticulous correction. Your feedback has prompted us to approach every detail of the paper with even greater rigor. We will strive to ensure that such errors do not occur again, allowing the paper to accurately and faithfully present our research findings and methods.

Original Comment:The manuscript should specify which version of the ResNet architecture is being used, as different versions can have varying impacts on performance. For example, ResNet has ResNet18, ResNet34, ResNet50. ResNet101 and ResNet152 variants.

Our Response:We greatly appreciate your observation on the importance of specifying the version of the ResNet architecture used in our paper.Your suggestion is crucial, as different versions of the ResNet architecture do exhibit performance variations that can significantly impact the understanding and evaluation of our research findings. In this study, we have employed the ResNet18 architecture.The primary reason for choosing ResNet18 is that, given the scale of our research data and the complexity of liver image features, ResNet18 strikes a good balance between computational complexity and feature extraction capabilities.Compared to other more complex ResNet variants (such as ResNet50, ResNet101, and ResNet152), ResNet18 has fewer layers and parameters, which helps the model avoid overfitting during training, especially with our relatively limited sample of liver imaging data, where its generalization performance is more outstanding.We have explicitly added an explanation of the use of the ResNet18 architecture in the "Materials and Methods" section of our paper, so that readers can clearly understand the considerations behind this key model choice, ensuring the reproducibility and interpretability of our research.We thank you once again for your professional review and constructive feedback, which is of great help in enhancing the quality and rigor of our paper.

Original Comment:In lines 134 and 135, the authors mention that the “3D model feeds the segmentation and labeling data from the VB-Net into the ResNet network for analysis.”. However, Figure 2 does not provide clarity on the shape or structure of the data from VB-Net.

Our Response:We are very grateful for your observation regarding the insufficient presentation of VB-Net data in Figure 2 and your attention to the rigor of our paper.In response to your feedback, we have actively taken corresponding improvement measures.As you have noticed, we have added dedicated references to VB-Net in our paper to allow readers to gain a deeper understanding of its technical details and principles.Additionally, to provide a more intuitive display of the shape or structure of VB-Net data, we have meticulously drawn a structural diagram of VB-Net in the supplementary materials.With these additional explanations and visual presentations, we hope to provide readers with a clearer and more comprehensive display of our research methods, assisting them in better understanding the data processing logic and architectural design of the VB-Net component throughout the entire analysis process.We sincerely thank you once again for your professional review and constructive suggestions, which have played a crucial role in enhancing the quality and readability of our paper.

Original Comment:A summary table detailing the final dataset used, including the total number of features and their corresponding labels, should be included for clarity.

Our Response:We greatly appreciate your valuable suggestions. We fully agree that a clear summary table will aid readers in better understanding our total number of features and their corresponding labels.Following your advice, we have included a detailed summary table in the supplementary materials section of our paper.This table encompasses the total number of features in the final dataset along with their corresponding labels, ensuring transparency and traceability of the dataset.We believe that this summary table will provide readers with a clear perspective, helping them comprehend the structure of the data we analyzed and how it is utilized for our research purposes.We hope this will meet your demands for clarity and transparency, further enhancing the readability and credibility of our paper.

Original Comment:The data augmentation strategies are not sufficiently detailed. The authors should discuss their impact on the overall performance deeply.

Our Response:We sincerely appreciate your observation that the section on data augmentation strategies lacked detail and your suggestion to delve deeper into their impact on overall performance. In the training of deep learning models, data augmentation is employed to increase the diversity and quantity of training data, thereby enhancing the model's generalization capabilities. Data augmentation in this study is achieved through various methods, including random flipping, rotation, and scaling.Random flipping includes both horizontal and vertical flips.This enhancement method can increase data diversity, with particularly noticeable effects in images with strong symmetry.Random rotation can be performed within any angle range, set between -5° and +5°.Random scaling can alter the size of images, typically scaling within a certain range from 1 to 2 times the original size, followed by cropping or padding back to the original dimensions.We have detailed the specific operational details of the aforementioned data augmentation strategies and related comparative experimental results analysis in the "Materials and Methods" section of our paper, to more comprehensively and deeply demonstrate the role and value of data augmentation strategies in this study. We express our gratitude once again for your professional review and constructive suggestions, which have played a crucial role in further refining our paper.

Original Comment: Data augmentation strategies are mentioned in multiple places. It would be beneficial to consolidate this information into a dedicated subsection.

Our Response:We are grateful for your constructive suggestion to consolidate information on data augmentation strategies into a dedicated subsection.We strongly agree with your perspective; such consolidation will enhance the systematic nature and readability of the paper's content.By integrating the data augmentation strategy information into a specific subsection and elaborating on it in detail, we aim to provide readers with a clearer, more focused, and in-depth understanding, allowing them to better grasp the role and significance of this critical aspect in the entire study.We sincerely thank you once again for your professional guidance and valuable suggestions, which have played an essential role in further optimizing the structure and content of our paper.

Original Comment: The normalization process is discussed in multiple sections (lines 177, 185); it should be consolidated into a single, clear explanation.

Our Response:We greatly appreciate your observation regarding the fragmented discussion of the standardization process throughout the paper and your suggestion to consolidate it into a single, clear explanation.We fully recognize the importance of this approach for enhancing the readability and logical flow of the paper, and thus have compiled and presented the standardization process in the paper as you suggested.By consolidating the standardization process into a clear explanation, we hope to enable readers to understand the specific content and significance of this crucial step more smoothly and comprehensively, avoiding the confusion that might arise from a dispersed discussion.We sincerely thank you once again for your professional review and constructive suggestions, which have played a crucial role in further improving our paper.

Original Comment: The choice of a 64x64 or 64x64x64 patch size should be justified, as other sizes may also be appropriate for this analysis.

Our Response:We greatly appreciate your constructive feedback on the choice of patch size. Your observation that patches of other sizes might also be suitable for this analysis has provided us with a new perspective to consider.In determining the use of 64x64 (for 2D data) and 64x64x64 (for 3D data) patch sizes, we conducted comprehensive considerations and experimental validations.Initially, we conducted exploratory research on a range of patch sizes, from smaller sizes like 32x32 (and 32x32x32) to larger sizes such as 128x128 (and 128x128x128). One of the main reasons for choosing 64x64 (and 64x64x64) is the balance between computational resources and efficiency.Smaller patch sizes like 32x32 (and 32x32x32), while increasing the number of data samples, might lose some global information during feature extraction, making it difficult for the model to learn a complete representation of liver tissue and lesions. Moreover, the large volume of data could significantly increase computational time and memory requirements during training, affecting the feasibility and efficiency of the experiments.Larger patch sizes like 128x128 (and 128x128x128), on the other hand, would make each sample contain too much local information, reducing the diversity of the data and leading to model overfitting. Additionally, the high demand for computational resources would exceed the capacity of our existing hardware facilities.In terms of feature expression capability, the 64x64 (and 64x64x64) patch size retains sufficient local detail while effectively reflecting the overall characteristics of the liver region.We thank you once again for your professional review and valuable suggestions.

Original Comment: In 186-187, the authors mentioned “Subsequently, the 3D liver images, along with their respective labels, are fed into the DCNN.”. This leads to confusion about whether the models are trained with both images and radiomic features. This should be clarified.

Our Response:We greatly appreciate your identification of potential confusion regarding the use of model training data and the opportunity to clarify.In this study, when 3D liver images and their respective labels are input into the Deep Convolutional Neural Network (DCNN), the model is trained solely on image data and does not concurrently utilize radiomic features.When constructing the model architecture, we specifically designed an image processing workflow based on DCNN that focuses on automatically learning and extracting deep features from the raw pixel information of 3D liver images. These features are directly derived from visual information such as texture, shape, and spatial relationships of the images. Through a series of convolutional, pooling, and fully connected layers, the model gradually constructs a high-level abstract representation of liver structure and lesions, thereby achieving the classification task of liver fat content.Radiomic features, on the other hand, are extracted and processed in a separate analytical pathway, primarily based on quantitative features obtained from statistical analyses such as gray-level histograms and texture matrices of liver images in different dimensions (e.g., 2D and 3D). These radiomic features are used to construct corresponding radiomic models, which are independent yet complementary to the DCNN-based image models, jointly serving to improve the diagnostic accuracy of liver fat content.Additionally, clear annotations have been made in the relevant model architecture diagrams to ensure that readers can accurately understand the role and usage of different types of data throughout the study. We thank you once again for your professional review and constructive suggestions.

Original Comment: In 189-190, the authors mentioned only one fully connected layer in the text, leading to conflict with Figure.

Our Response:We sincerely appreciate your observation of the inconsistency between the text of the paper and Figure 2 regarding the description of the fully connected layers. This was indeed an oversight on our part during the writing and illustration process, for which we apologize.In the actual research, we used one fully connected layer, but Figure 2 incorrectly depicted two fully connected layers.During the revision of Figure 2, we reorganized the distribution of the modules to present a clearer hierarchical structure of the construction processes for both the radiomic model and the deep learning model in the figure. Placing the shared data preprocessing section at the top starting area of the figure visually indicates that this is the foundational step for the entire model construction. Following this, the unique construction segments of each model are expanded downward, which helps readers quickly establish an understanding of the overall process framework from initial data to final construction completion for both models, avoiding the process confusion and visual interference that the previous layout might have caused.We hope that the revised Figure 2 will meet your expectations, and should you have any further comments or questions, please feel free to communicate with us at any time.

Original Comment:In 197-198, the authors mentioned “models is evaluated by analyzing the ROC curve and calculating the AUC, which includes key metrics like accuracy, sensitivity, and specificity”. The authors state that models are evaluated using the ROC curve and AUC. It is important to clarify that AUC measures the model's ability to distinguish classes, while accuracy reflects overall prediction correctness. The authors should explain how accuracy is derived from AUC values.

Our Response:We greatly appreciate your observation regarding the unclear explanation of the relationship between the model evaluation metrics AUC and accuracy, which is crucial for enhancing the rigor of our paper. To obtain the AUC, we first set a range of different thresholds for the scores predicted by the model for each sample in the test set, ranging from 0 to 1 with a certain step size (e.g., 0.05). For each threshold, we calculate the corresponding True Positive Rate (TPR) and False Positive Rate (FPR).TPR is calculated as the number of true positives (TP) divided by the total number of actual positive samples (TP + FN); FPR is calculated as the number of false positives (FP) divided by the total number of actual negative samples (FP + TN). Using these (FPR, TPR) data points, we plot the ROC curve and calculate the area under the curve using professional statistical analysis software or mathematical algorithms to obtain the AUC.Sensitivity (Recall) is calculated by dividing the number of true positives (TP) by the total number of actual positive samples (TP + FN).Specificity is calculated by dividing the number of true negatives (TN) by the total number of actual negative samples (FP + TN).Accuracy is calculated by dividing the sum of the number of true positives and true negatives (TP + TN) by the total number of samples in the test set (TP + FP + FN + TN).Precision is calculated by dividing the number of true positives (TP) by the total number of samples predicted as positive (TP + FP).The F1-score is the harmonic mean of precision and recall, calculated using the formula F1-score = 2 * (Precision * Recall) / (Precision + Recall). Should you have any further comments or questions, we welcome your communication at any time.

Original Comment:In 202-211 lines, the “Participant Characteristics” section should be moved to a more appropriate place within the dataset description in the methodology.

Our Response:We greatly appreciate your suggestion to move the "Participant Characteristics" section to a more appropriate location within the dataset description in the methods. Your insight is highly accurate and will help to enhance the rationality and logic of the paper's structure. We fully agree with your perspective and have made the necessary revisions to the paper according to your suggestion.In the revised paper, we have integrated the "Participant Characteristics" section into the "Study Cohort" subsection. With this adjustment, readers can now concurrently obtain information about participant characteristics while understanding the construction of the dataset, gaining a clearer understanding of the composition and nature of the data. This allows them to better grasp the data foundation upon which the entire study is based, enhancing the coherence and completeness of the methods section in the paper.We thank you once again for your professional review and constructive suggestions, which have played a crucial role in further optimizing the structure of our paper.

Original Comment:The pre-processing steps should be organized into a dedicated subsection for clarity.

Our Response:We greatly appreciate your valuable suggestion to organize the preprocessing steps into a dedicated subsection.We deeply recognize that doing so will greatly enhance the clarity and logic of the paper's presentation. We have established a new subsection titled "Data Preprocessing" in the "Materials and Methods" section of our paper.By integrating the preprocessing steps into a dedicated subsection and elaborating on them in detail, we hope to provide readers with a clearer, more focused, and in-depth understanding, enabling them to better grasp the role and significance of this critical step in the entire study.We sincerely thank you once again for your professional guidance and valuable suggestions, which have played an essential role in further improving our paper.

Original Comment:It’s not clear how the authors incorporate CNN layers, ResNet, and VB-Net for 2D/3D features, as shown in Figure 2, for use in classifiers such as SVM, RF, and others.

Our Response:We greatly appreciate your observation regarding the unclear explanation of how CNN layers, ResNet, and VB-Net integrate 2D/3D features for the classifier. Here is our detailed response and explanation.Initially, we employ two methods for image segmentation: manual 2D segmentation and automatic 3D segmentation based on VB-Net.We then construct radiomic models and deep learning models for both sets of segmented images and raw data.For the radiomic model, after feature extraction and selection, we use twelve machine learning algorithms to build the radiomic model.For the deep learning model, we preprocess the images by cropping, rotating, and flipping, and employ data augmentation strategies (as detailed in a subsection). The 2D raw images and 2D masks, after cropping, serve as inputs for the 2D ResNet-18, constructing the 2D deep learning model.We form a dual-channel input with the original images and 3D masks, feeding them into the 3D ResNet-18 network to construct the 3D deep learning model.We hope this explanation provides a clearer understanding of our research methodology.We thank you once again for your professional review and valuable suggestions.
Original Comment:The terms “2DDLS” and “3DDLS” in Table 4 require definitions, as they are introduced without prior explanation.

Our Response:We sincerely appreciate your observation regarding the undefined terms "2DDLS" and "3DDLS" in Table 4.We apologize for this oversight and have supplemented the definitions and explanations of these terms in the relevant sections of the paper, ensuring that readers can clearly understand their meanings and applications in the study.We thank you once again for your professional review and valuable suggestions, which have played a crucial role in further improving our paper.

Original Comment:There is inconsistency in labeling (e.g., -, +, ++, +++ in Figure 2 vs. 0-3 in Figure 3). A standardized labeling system should be adopted throughout the manuscript, including in the confusion matrices. Furthermore, the confusion matrices in Figure 3 have different labels in different orders.

Our Response:We greatly appreciate your observation regarding the inconsistency in labeling.Your feedback is both accurate and constructive, and we have taken it very seriously, taking appropriate measures to address the issue.We have conducted a comprehensive review and standardization of the labels throughout the manuscript.Regarding the symbols -, +, ++, +++ in Figure 2 and the 0-3 scale in Figure 3, we have re-examined the meanings these marks represent.After careful consideration, we have decided to adopt a standardized marking system to ensure consistency throughout the manuscript.Furthermore, due to the significant difference in diagnostic performance between the 2D radiomics model based on random forests and the 3D deep learning model, we have carefully considered and removed the ROC curve and confusion matrix for the 3D deep learning model from the text. However, we have added background information regarding the qualitative assessment of liver fat by experienced radiologists using QCT as the standard. We appreciate your professional review and valuable suggestions, which have played a crucial role in further refining our manuscript.

Original Comment:The authors are encouraged to utilize cross-validation techniques to enhance the robustness of their findings.

Our Response:We greatly appreciate your suggestion to employ cross-validation techniques to enhance the robustness of our research findings.Your advice is extremely valuable, and we fully agree on the importance of cross-validation in assessing the generalizability and reliability of our model's results. Following your suggestion, we have implemented a 5-fold cross-validation technique in our study.Utilizing this cross-validation method, we have validated the features obtained from our radiomics model on the dataset and found that 15 2D features and 20 3D features exhibit high robustness and reliability.We are grateful for your professional guidance; this suggestion has provided crucial support for the optimization of our research.

Original Comment:The manuscript lacks details regarding the percentage of data used for training and testing. This information is crucial for replicability.

Our Response: We sincerely appreciate your pointing out the omission of information regarding the percentages of training and testing data in our manuscript.Your reminder is of utmost importance for enhancing the replicability of our research. We have supplemented the "Materials and Methods" section of our paper with detailed explanations of the percentages of training and testing data, including the specific method of data division, and once again, we thank you for your professional review and constructive suggestions.

Original Comment:The authors may consider developing two scenarios: one that incorporates images in both training and testing and another that focuses solely on radiomic features. This approach could strengthen the overall findings.

Our Response:We greatly appreciate your constructive suggestion to develop two approaches to strengthen the overall results.Your advice has provided invaluable insights for us to further deepen our research. For the approach focusing solely on radiomic features, we have dedicated a subsection in the methods part of our study to showcase the 15 radiomic features of the 2D optimal model,and in the supplementary materials, we have demonstrated the contribution of these fifteen features to the four-category diagnosis of fatty liver.We thank you once again for your professional review and valuable suggestions, which have played a crucial role in expanding our research directions and enhancing the quality of our study.

****Reviewer Comment 3:****

Original Comment: I have concerns regarding the overall relevance of this work as it seems similar to work that has previously been published by other authors such as 10.3346/jkms.2022.37.e339. However the inclusion of extra models may warrant the article for publication and therefore it may be a good idea to reference earlier work such as this and compare why your work is superior.

Our Response:We sincerely appreciate your meticulous review of our work and the valuable feedback provided. Your concern about the similarity to other published works is indeed something that warrants our deep consideration and response.We have included the relevant literature as a reference in our article and provided a more detailed discussion in the "Discussion" section, allowing readers to clearly understand the similarities and differences between our work and existing studies, as well as the strengths of our research.We thank you once again for your professional review and constructive suggestions, which have played a crucial role in further refining our paper and highlighting the unique aspects of our research.

Original Comment: Repeatedly throughout this work you have used acronyms. Please ensure your usage of acronyms is consistent, with the acronym defined at first usage and then used consistently in the same format throughout. Additionally, please ensure you always place a space betwee the full term and the parentheses.

Our Response:We greatly appreciate your pointing out the issues regarding the use of acronyms. Your feedback is of utmost importance for enhancing the standardization and readability of our paper. We have conducted a comprehensive and meticulous review and revision of the paper to ensure that the use of acronyms complies with academic standards.We have provided a clear definition for each acronym at its first occurrence and have consistently used the acronym in the same format throughout the text.Additionally, we have paid special attention to the spacing issue between full terms and parentheses, strictly adding a space between them as required, making the layout of the paper more standardized and neat.We thank you once again for your professional review and constructive suggestions, which are indispensable for further improving our paper.

Original Comment: On line 32, do not start a sentence with "Assess". Instead use a term such as "we assess"

Our Response:We greatly appreciate your attention to and suggestions for the nuances of language expression in our paper.Your observation that the 32nd line should not begin with "Assess" but should instead use "We assess" is very pertinent.This phrasing clarifies the subject of the sentence, enhancing the coherence and readability of the paper's language, and allowing readers to more clearly understand that the executors of the research actions are the authors' team.In the subsequent polishing process of the paper, we will also take this as a reference, paying more attention to the standardization and fluency of language expression to avoid similar issues from reoccurring.We thank you once again for your professional review and valuable suggestions, which have played an important role in promoting the quality of our paper.

Original Comment:Online 34 you say accuracy was confirmed through comparison with "manual assessments". Please state here what assessments these are.

Our Response: We sincerely appreciate your pointing out the unclear description of "manual assessment" mentioned in line 34 of our paper.The manual assessment we referred to is the process of measuring liver fat based on Quantitative Computed Tomography (QCT). Specifically, this is carried out jointly by professional medical imaging technicians and experienced radiologists.We have added a separate section in the corresponding part of the paper (such as the "Materials and Methods" section) to provide a detailed description of the manual assessment process, so that readers can clearly understand how we confirm accuracy and the specific connotations of manual assessmentWe thank you once again for your professional review and constructive suggestions, which have played a crucial role in further improving our paper.

Original Comment: On line 66-68, you make a very straong statement regarding the superiority of MRI and CT, there should be a relevant citation here to support this argument.

Our Response:Thank you for highlighting the lack of citation support for the statements on the superiority of MRI and CT in lines 66-68.Your suggestion is crucial for enhancing the scientific rigor and credibility of our paper. We have revisited a substantial body of relevant literature and identified several studies that robustly support the superiority of MRI and CT in the field of liver disease research, which we have now cited in the text.We have added these citations at the appropriate locations in the paper and detailed the specific information of the cited literature in the reference list, allowing readers to consult and further understand the basis of our claims.We are grateful once again for your professional review and constructive suggestions, which have been instrumental in further improving our paper.

Original Comment: On lines 91-93, you start the sentence as if you are going to define both inclusion and exclusion criteria, but then only define exclusion criteria. Were no specific inclusion criteira used?

Our Response:Thank you for identifying the issues in the standards description on lines 91 to 93.In fact, our study first applied exclusion criteria to screen potential participants, and after excluding those who did not meet the criteria, all remaining individuals were included in the study.We have also revised our wording to avoid ambiguity, ensuring that readers can clearly understand how our study's sample was determined.Once again, thank you for your professional review and constructive suggestions, which have been crucial in helping us further refine our paper.

Original Comment:The sentence on lines 99-101 seems very out of place compared to the current text and may be better placed elsewhere or removed.

Our Response:Thank you for pointing out the issues in the sentences from lines 99 to 101.Your feedback is very constructive. After careful consideration, we have decided to delete this section. Upon analysis, we found that these sentences indeed disrupt the logical flow with the surrounding context. Their content does not integrate well with the core theme of the paragraph, potentially causing confusion for readers and disrupting the smooth understanding of the overall research narrative.After removing this section, we have made corresponding adjustments to the surrounding text to ensure a natural and smooth transition, thereby clarifying and tightening the logical structure of the paper.We are grateful once again for your professional review and constructive suggestions, which are crucial for further optimizing our paper.

Original Comment:On lines 108-109, you state that participants were categorised. What categories were they categorised into?

Our Response:Thank you for your inquiry regarding the participant classification in our paper.As mentioned in lines 108-109, the participant classification is as follows: Normal liver (CT fat fraction < 5%), Mild fatty liver (CT fat fraction 5%-14%), Moderate fatty liver (CT fat fraction 14%-28%), and Severe fatty liver (CT fat fraction >28%). We have provided detailed information on participant classification in the "Materials and Methods" section of our paper,and we are grateful once again for your professional review and constructive suggestions, which have been crucial in helping us further refine our paper.

Original Comment: On lines 173-175, these two sentences present the same information but with a slight difference in wording. Therefore they are not both needed.

Our Response:Thank you for highlighting the redundancy in the text from lines 173 to 175.Your observation is very astute, and we fully agree with your perspective.We have retained only one instance.This approach will make the text more concise, preventing readers from experiencing repetition, and also enhance the overall flow and readability of the paper.In subsequent revisions of the paper, we will pay closer attention to checking and optimizing similar expressions to ensure that every sentence accurately and efficiently conveys key information. Once again, thank you for your professional review and constructive suggestions, which are crucial for further improving our paper.

Original Comment:In the "deep learning model development..." section, you say that data is fed to the DCNN but do not give any descriptions regarding the architecture. You should discuss the architecture here first (perhaps by moving the text from lines 188-192) before going on to say more. Instead, you simply discuss the "ResNet" layer on with no context.

Our Response:Thank you for pointing out the issues with the model architecture description in the "Deep Learning Model Development..." section.Your suggestions are invaluable for enhancing the logic and completeness of our paper.In the 3D automatic liver segmentation section, our study has incorporated a reference to the VB-Net segmentation network and has depicted the structure of the segmentation network in the supplementary materials.With these adjustments, we hope to provide a clearer understanding of the deep learning model's architecture and the roles and interrelationships of its components for our readers.We are grateful once again for your professional review and constructive suggestions, which are indispensable for further optimizing our paper.

Original Comment:On lines 188-192, you first state that cross entropy loss was used and then that focal loss was used. Is there a reason for this specification of two different loss types. If both were used it would be best to provide the context as to why.

Our Response:Thank you very much for raising questions about the description of the loss function in our paper.Upon careful examination and review, we have identified inaccuracies in our description. In fact, our research only utilized the focal loss function and did not employ the cross-entropy loss function.During the initial drafting of the paper, a typographical error or confusion in expression led to the incorrect mention of cross-entropy loss, which was an oversight on our part.We have removed the erroneous mention of cross-entropy loss.We are grateful once again for your professional review and constructive suggestions, which have been crucial in helping us promptly correct errors and refine our paper.

Original Comment:You provide discussions of results but do not provide any description as to any particular processes that were used to validate the results that you have provided except for the use of the test set. For instance, did you use techniques such as K-Fold validation or repeated experiments?

Our Response:We greatly appreciate your suggestion to employ cross-validation techniques to enhance the robustness of our research findings.Your advice is extremely valuable, and we fully agree on the importance of cross-validation in assessing the generalizability and reliability of our model's results. Following your suggestion, we have implemented a 5-fold cross-validation technique in our study.Utilizing this cross-validation method, we have validated the features obtained from our radiomics model on the dataset and found that 15 2D features and 20 3D features exhibit high robustness and reliability.We are grateful for your professional guidance; this suggestion has provided crucial support for the optimization of our research.

Original Comment:There are a number of textual issues with this manuscript that need to be significantly addressed before it can be published. PLease check for English spelling, grammar and textual formatting errors, as there are multiple occurences of issues such as sentences ending and a new one starting with no space between and no spaces between the end of text and beginning of citations.

Our Response:We sincerely appreciate your pointing out the textual issues in our manuscript; your reminder is crucial for enhancing the quality of our paper.We have deeply recognized the adverse effects of these issues on the readability and professionalism of the paper and have immediately taken the following comprehensive corrective actions.We have organized team members to conduct sentence-by-sentence English spelling and grammar checks on the manuscript.Using professional grammar checking tools and referring to authoritative English grammar books, we have carefully identified and corrected various grammatical issues such as subject-verb disagreement, tense errors, and misuse of parts of speech, while ensuring the accuracy of spelling. Not only have we corrected potential language errors, but we have also optimized the fluency and logic of the expressions, making the language style of the paper more in line with the requirements of academic publishing.We thank you once again for your professional review and constructive suggestions, which have played an extremely crucial role in further improving our manuscript.

Original Comment:Additionally, there are a number of instances of textual discussions that do not match with the sections that they are discussing or provide information later on in a discussion when it should have been earlier (comment 7 + 10). I would suggest conducting a thorough re-evaluation of the article structure and ensuring that all text flows adequately and in the correct locations.

Our Response:We sincerely appreciate your identification of issues with the article's structure and the valuable suggestions you've offered. Your meticulous review has made us realize the significant impact of text layout on the clarity and logic of the paper.We have conducted a comprehensive and in-depth re-evaluation of the article's structure.In response to the mismatch between text discussion examples and their respective sections, as well as the improper placement of information, we have reorganized the core arguments and logical threads of each chapter.We thank you once again for your professional review and constructive suggestions, which have been indispensable in thoroughly optimizing the article's structure and enhancing the quality of our paper.

Original Comment: The captions you have provided for figures are increasingly long. I would suggest removing some of the text entirely or relocating it to the main body of text.

Our Response:We greatly appreciate your observation about the length of the figure titles and the valuable suggestions for improvement.Your feedback is of great significance in enhancing the conciseness and readability of our paper.We have carefully reviewed each figure title and have made adjustments to those that indeed contained content that could be streamlined or repositioned. For figure titles that were redundant in information, we have removed some non-essential descriptive sentences, retaining only those parts that can accurately summarize the core content and main conclusions of the figure, making the titles more concise and highlighting the key points.With the above modifications, we hope that the figure titles in our paper will better serve our readers and enhance the overall reading experience.We thank you once again for your professional review and constructive suggestions, which have played a crucial role in further improving our paper.

Thank you once again for your professionalism and for the opportunity to refine our research for publication. We are hopeful that our revised manuscript meets the high standards of your esteemed journal.

Best regards,

Xiaopeng Yang

Department of Radiology, The First Affiliated Hospital of Zhengzhou University, No.1, Eastern Jianshe Road, Zhengzhou 450052, Henan Province, China.

Email: [13837141925@163.com](mailto:13837141925@163.com)

November 30, 2024
